# Trajectories of random walks on Markov sources reveal the structure of complex sequences

**DOI:** 10.1371/journal.pone.0359402

**Published:** 2026-11-11

**Authors:** Ali Altunkaya, Arunabha Sen, Nicholas J. Schork

**Affiliations:** 1 Arizona State University, Tempe, Arizona, United States of America; 2 The Translational Genomics Research Institute, Phoenix, Arizona, United States of America; Tohoku Daigaku, JAPAN

## Abstract

We highlight a hierarchical property that is found in many sequences, the property of subunit structure, in which sequence symbols are joined together in tightly knit groups to create new subunits at higher levels of organization. Many sequences have strong connections both between the subunits and within the subunits. We propose an information-theoretic method that reveals hierarchical subunit structure in sequences. We use the probability flow of random walks on the Markov source encompassing transitions from subunit to subunit as a proxy for information flow in the sequence. We decompose the sequence into subunits by compressing the description of the information flow within the Markov source that generates the sequence. We illustrate the method by partitioning several real-world sequences whose subunit structure is already known and find that the method detects this known structure with high sensitivity and reliability. We show the relative complexity of different types of sequences in terms of bits per transition step. Finally, we show relative information flow between levels of the sequence within a revealed hierarchy.

## Introduction

Many objects in nature, from cells to computers to humans, store and communicate information using sequences of symbols and specific groupings of those symbols. These groupings (or ‘messages’) are organized and ordered using intermediate level subunits that envelope or connect a ‘microscopic’ order of symbols to the ‘microscopic’ panorama of a broader message. These messages also exhibit characteristics of complex systems, such as each message being composed of many subunits that are associated with each other in non-random ways. Additionally, they often exhibit multiscale or hierarchical architectures or structures; e.g., messages are composed of subunits, and those subunits may reflect relationships between 'sub-subunits' and so forth -- all reflecting patterns built off an atomic or basic set of symbols.

Complex systems need intermediate stable forms or subsystems to evolve rapidly, so subunits encompassing the atomic units within these subsystems act as new building blocks for further construction at higher level subsystems [1,2]. We can observe these phenomena in natural languages: basic atomic units, such as letters or sounds, combine and generate syllables, syllables combine and generate words, and so on with phrases, sentences, paragraphs, sections etc. Similarly, biological sequences are often generated from subunits composed of atomic elements such as nucleotides. For example, DNA sequences reflect subsystems exhibiting levels within a broader hierarchical organization encompassing amino acids, codons, exons, genes, regulatory networks, etc.

As another example, note that to write a good book or text, an author must organize the information in a structured way. Good books both reflect and emphasize important concepts and structures within a more broadly defined idea, concept or set of ideas and/or concepts. There is a trade-off between highlighting most of the ideas at the expense of long description with many subunits and omitting some aspects of the ideas to allow shorter description with fewer subunits. An author should achieve a fine balance between excluding important structures by oversimplification and hiding significant relationships in the sea of superfluous details. To understand this trade-off, assume that a book reflects ideas that can be captured in 10 chapters (however organized and defined): if the author decides on using fewer chapters then they will omit some important ones; whereas if they provide hundreds of chapters then significant ideas will get lost in the redundancy. The best books contain a great amount of information but also the ideal bandwidth or conveyance of the main ideas and concepts. In this way, the best books are also good compressions. By using an information-theoretic approach, it is possible to measure how efficiently a book or text represents the underlying ideas or information conveyed.

If one considers the same scenario but from a reader’s perspective, or from the perspective of an early language acquirer, then the issues take on a different light. For example, 8-month-old infants can segment continuous speech into word-like subunits after a few minutes of listening. However, interestingly, they use a recognition of the transition probabilities between syllables for segmentation, not just the frequency of those syllables. Similarly, as they get older, children can detect sentences from continuous speech using the same kind of statistical learning based on the transition probabilities between the subunits, not just their frequency [3–5]. The use of transition probabilities is also at the heart of large language models (LLMs) where patterns among consecutive subunits (e.g., tokens such as words) are exploited to anticipate adjacent tokens in a broader sequence of tokens. However, LLMs do not necessarily reveal the hierarchical nature of the different subunits and substructures.

We define the problem of detecting subunit structure of a sequence using a few analogies. Assume all punctuation marks and space from a big novel (e.g., War and Peace) are removed, so it will become a single very long sequence of letters. Can we detect its subunits by, e.g., compressing the sequence in various ways to identify meaningful patterns? Similarly, a genome is a very long sequence of DNA nucleotides so can functional subunits or elements within that sequence be identified by compressing the sequence? Very recent efforts to understand how humans learn and process information leverage compression and related techniques as well as entropy analyses [6–10].

We propose a method that is complementary to the methodology used in many different settings. For example, community detection algorithms used to detect patterns and hierarchies in networks are very mature and have been shown to be capable of finding modules or tightly-coupled groups of nodes in a network. Attempts to apply them to sequences have been mixed [10], but is an ongoing research problem. In addition, the phenomenon of the ‘emergence’ of hierarchies and causal connections between subunits and units within different levels of a hierarchy in complex systems has been extensively studied [11–15]. However, much of this previous work focused on networked systems such as logic gates, directed acyclic graphs (DAGs), and some types of Markov chains. Our method helps bridge the gap between legacy network analysis and sequence analysis, as it focuses on sequences, not networks, using similar concepts but different statistical constructs. Our method partitions a sequence and finds subunits that emerge at higher levels of a hierarchy consistent with, and likely generative of, the observed patterns in a sequence. In addition, our method could be helpful to the theory and understanding of cognition, as psychologists, neuroscientists, and cognitive scientists have been studying how humans read and learn using natural neural networks apparent in the brain for some time [7–9], and our proposed method could provide a foundation for the operation of these networks.

We want to emphasize that our methodology is indeed complementary to previous related work and can in fact bridge the gap between relevant network analysis and sequence analysis. In network analysis, there are many popular algorithms such as Infomap, for detecting communities in a network, but Infomap cannot be used for modelling sequences or for detecting the subunits of a sequence [16]. On the other hand, in sequence analysis, arbitrary-order Markov chain models have been used to model sequences [10], and in fact this approach is also used to find the theoretical lower limit of compression, but it cannot detect the subunits of a sequence. Detecting the subunits of a sequence is an open research problem, and to our knowledge there is no powerful method for that purpose. Our methodology is an attempt to fill that gap with the following in mind: 1. We model sequences as particular Markov trajectories on the source Markov chain; 2. We develop a new equation to measure the entropy of a particular Markov trajectory, and we develop a greedy algorithm to detect the subunits of a sequence by minimizing this equation. Our equation may look similar to the Infomap equation since both utilize the MDL (Minimum Description Length) principle, but they are very different in their derivations and formulations; and 3. Our method determines the theoretical lower limit of sequence compression based on our two-level coding mechanism and it achieves better compression rates than other methods as explained in the results section. Therefore, our method, when applied to different data sets, shows empirically its improvements over other methods.

## Methods: Modelling sequences as Markov trajectories

Language makes infinite use of finite media or subunits [17]. Authors of written texts encode their ideas and thoughts into writing, and the readers decode them to ideas by reading [17]. Creating new subunits or words for each possible combination of ideas or concepts would be pointless and would require massive storage or memory if captured on media like computers. The use of subunits or words, or combinations of subunits or words, as labels for some basic ideas, concepts, or objects in the real world results in a storage of the idea as an information flow between the subunits of sequences. For example, “quantum” is a label for a complex idea, but its information is stored as a flow between subunits of long text sequences including the word "quantum."

We propose the use of Markov trajectories of a random walker to describe the dynamics across the nodes and links of a Markov chain source that generates a sequence harboring information [18–20]. Markov trajectories can also be shown to represent the local associations between the subunits within a system encompassing the specific sequence and the information it conveys. These local associations within and between subunits generates a system-wide flow of information which characterizes the behavior of the full system. To understand the relationships between atomic elements, substructures, generated sequence, and the characteristics and behavior of the system generating sequences, the flow of information needs to be exposed. Markov trajectories of a random walker can be used to capture efficient coarse-grained descriptions of the information flow between atomic elements, subunits and substructures within sequences that provide information.

### Random walkers, compression strategies and coding theory

We exploit the transition probabilities of a random walker across revealed Markov processes inherent in a sequence as a proxy for information flow within that sequence. We identify the subunits of a sequence by compressing the information flow within the system [16,21–23] and thus the subunit identification process can be viewed as a coding or compression problem in information theory. Thus, in our scheme, finding regularities within a sequence depends on our ability to compress the sequence in various ways [24–26]. Coding theory dictates that a sequence can be compressed by a code which exploits the regularities in the source that generated the sequence [27–29]. A random walker along a Markov Chain network that can generate the sequence can reveal these regularities and the structures that led to them. To start the process on a given sequence, we generate the Markov Chain network from the whole sequence, where each node is a symbol (i.e., an atomic symbol) and links are the transition probabilities between them. We then assume that a random walker traverses this network infinitely using computed transition probabilities and a small teleportation probability. For each step, the assumed random walker at a specific point in the sequence ‘visits’ a neighboring symbol with 0.85 probability and visits all nodes within the revealed network with 0.15 probability. We choose the teleportation probability of τ=0.15 to be consistent with the Google PageRank algorithm [30–32] but of course this could be modified and the use of different values explored. With this approach, we can reach the unique steady stationary distribution, where each symbol α has an ergodic node size $p_{\alpha }$ and $\sum p_{\alpha }=1$.

### Entropy of a particular Markov trajectory

The Markov trajectories on the source Markov Chain network which generates the sequence can analyzed for various properties including local and more global entropy values. A Markov trajectory $t_{\alpha \beta }$ is a path from start state α to final state β of a Markov chain without intervening the final state β. This is consistent with real life sequences; e.g., a word starts after a space, and it ends with a space, but a word cannot include a space character, or similarly a sentence starts and ends with a period but cannot include a period. Because of that, we introduced a hypothetical exit symbol *q* while partitioning the whole sequence into subunits. Ekroot & Cover computed the entropy of Markov trajectories, $t_{\alpha \beta }$, by finding the probability of all possible trajectories from start state α to final state β, and applying Shannon’s entropy formula ∑plog(p) [18]. This method quantifies the randomness of Markov trajectories with fixed initial and final states. Our method focuses on the entropy of a particular Markov trajectory where all states of the trajectory are fixed, which represent subunits in a hierarchical organization. Thus, a subunit *i* of length *k* is a particular Markov trajectory $T_{i}=qt_{\alpha 1}t_{\alpha 2}...t_{\alpha k}q$ which starts and ends at special exit state *q* and visit the states from $\alpha_{1}$ to $\alpha_{k}$. For each state α in the Markov Chain, we computed its outflow entropy $H(O_{\alpha })$ using Shannon’s formula ∑plog(p). A subunit can also be viewed as a sequence of independent but not identically distributed random variables generated by a Markov process $T_{i}=O_{\alpha 1}O_{\alpha 2}...O_{\alpha k}$, where each random variable is the outflow entropy of a corresponding state. Such subunits are not identically distributed, because each state’s outgoing links are different. Cover showed that the entropy of independent, but not identically distributed, random variables generated by a stochastic process equals the sum of each random variable’s entropy in the sequence [29]. In our scheme, we need to add the outflow entropy of exit states too. Thus, the entropy of a particular Markov trajectory, $T_{i}$, equals $H(T_{i})=H(O_{q})+\sum_{\alpha =1}^{k}H(O_{\alpha })$.

### Communication games and coding

To demonstrate the relationship between implicit codings and Markov trajectories, assume both an author of a written document and a reader know the same language which is represented by a weighted and directed Markov Chain network. They implicitly choose a code which efficiently describes a particular trajectory of a random walker on this Markov Chain network. Consider Fig 1A which shows a Markov Chain network of a sequence with 95 particular steps. In Fig 1 this particular trajectory is depicted with increasing levels of compression (Fig 1A–D), exploiting regularities in the Markov Chain network that generates it. A straightforward way of labeling links in the chain is to use uniform code. Each node exploits a unique dictionary covering the outgoing transitions. We can choose a uniform code for the outgoing links where all code words are of equal length. In Fig 1A, node *i* has 5 outgoing links, each link can be encoded using ⌈log5⌉=3 bits. After giving names to the outgoing links of all nodes, we can describe this 95-step trajectory using 154 bits, with code length L = 1.62 bits per step. Huffman coding is an efficient way of labeling the links since it assigns short code words to more frequent events and long code words to rare events. Fig 1B shows the Huffman coding for our sample trajectory. We can describe the same 95-step trajectory using 134 bits, L = 1.41 bits per step. In Fig 1 we assign actual code words for demonstration; however, our focus is not on the code words themselves, but rather the theoretical lower limit of the coding describing a trajectory.

**Fig 1:**
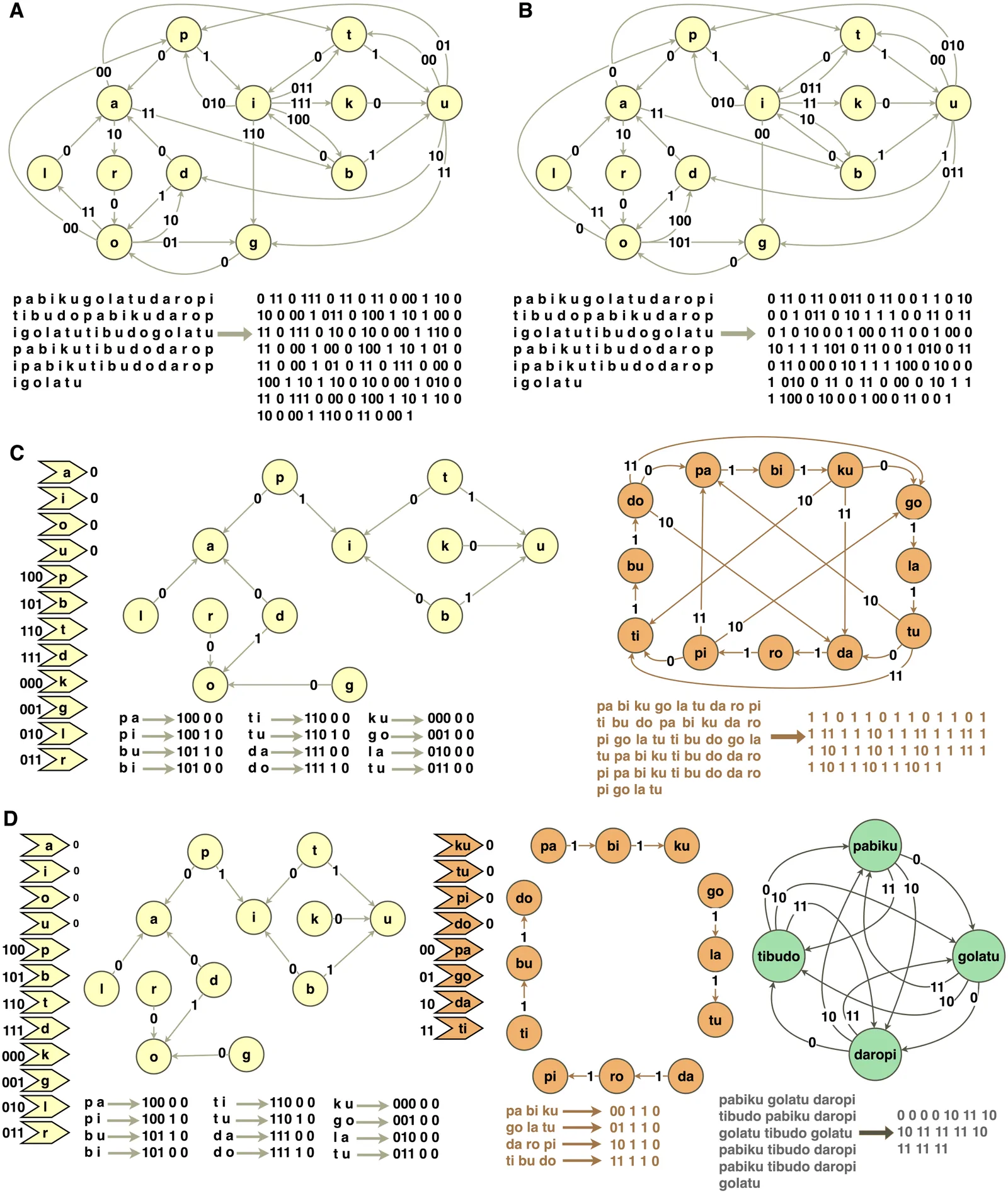
Detecting subunits by compressing the description of information flows on sequences. (A) We want to describe a sequence using the trajectory of a random walk on its Markov Chain network, such that each unique subunit has a unique code. A basic approach is to give a unique code to the outgoing links of each node. The uniform code illustrated here describes the sample trajectory in (A) using 154 bits, starting with 0 for the first transition from p to a, 11 for the second transition from a to b, etc. and ending with 1 for the last transition from t to u, on the walk. (B) The Huffman code illustrated here is more efficient way to encode the same walk using only 134 bits. (C) A two-level description of the random walk, where we encode the whole sequence using two Markov Chains. The codes used to indicate entering the Markov Chain from each subunit and exiting the Markov Chain from each subunit are shown to the left and right of the arrows near the network, respectively. While encoding the subunit “pa”, the first three bits '100' indicate that the walk begins from “p” in yellow Markov Chain, and the bit 0 indicates the transition from p to a, and the last bit 0 indicates the exit from subunit a. Using this two-level description, we can describe the same walk using shorter description with 118 bits. (D) A hierarchical approach generalizes the two-level description to multiple levels. Using the hierarchical three level description illustrated here, we can further compress and encode the same walk using 106 bits.

### Identifying and highlighting important structures

Although maximal compression of sequences is an important element in our strategy, it is not the only one. We also want to detect subunits of the sequence which correspond to the phenomena for which the information in the sequence is most relevant. For example, while writing a book, an author needs to use intermediate-level subunits such as words or phrases to assign real-world objects, but there is a need to separate the coarse grained subunits used in the book from the fine-grained symbols. Therefore there is a need to describe the sequence using at least two levels of description. We can give unique code to the coarse-grained subunits, but we can reuse the codes for fine-grained subunits. This is a common approach in writing as words have different meanings in different contexts. For example, the words “hot” or “season” have different meanings whether they are written in the weather section or the food section of a newspaper. A two-level description allows us to describe the same Markov trajectory in fewer bits. Thus, we can exploit the regularities in a Markov Chain network that reflect where a random walker will spend more time in certain sub-trajectories. In this light, we assume that two Markov Chains are needed, as illustrated in Fig 1C. We can reuse the same codes for different subunits on different levels. This is advantageous in capturing hidden structures as is identifying others structures in the sequence by using longer or larger of subunits inferred from the sequence. Using the two-level description in Fig 1C, we can describe the same Markov trajectory in 118 bits, L = 1.24 bits per step. There is a duality between identifying the subunit structure of a sequence and compression of the sequence. We have considered a two-level description by applying the Minimum Description Length (MDL) principle [25]. If the subunits are too long, then we need more bits to encode the flow within subunits and less bits to encode the flow between subunits. If the subunits are too small, we need less bits to encode flow within subunits but more bits to encode the flow between the subunits. According to MDL principle, the optimal partition of the whole sequence into subunits will give the shortest description length of the whole sequence [24–26]. To find an efficient code, we then look for sequence partition, *S*, of *n* symbols into *m* subunits:


$$\begin{array}{c} L(S)=qH(T_{whole})+\sum_{i=1}^{m}F_{i}H(T_{i}) \end{array}$$ (1)

Equation (1) has two terms: first is the entropy of the Markov trajectory between the subunits, and second is the entropy of Markov trajectories within the subunits. Each one is weighted by the frequency with which it occurs, or by the time the random walker spends on that trajectory. Here *q* is the time that the random walker switches between these two Markov Chain networks, and $F_{i}$ is the time random walker spent time in the subunit *i*.

There are an exponential number of possible ways to partition a sequence into its subunits. Therefore, it is not feasible to check all of them to find the partition that minimizes our equation. We develop a greedy heuristic approximation algorithm to optimize equation (1) for a given sequence. We first compute the ergodic node visit frequencies of the random walker using the power method and then explore the space of possible partitions in a greedy approach [16,33]. One limitation of our algorithm is that it can find the subunits of simple sequences such as in the ‘pabiku’ lullaby, but future work will need to increase its applicability for more complex real-world sequences (see the S1 File for more details). Ultimately, as emphasized throughout, we can generalize this basic two-level description to identify higher-level subunits at multiple levels within an encompassing hierarchy [34–36]. Thus, starting from the finest level subunits, we can iteratively use the proposed strategy to find more coarse-level subunits. Fig 1D illustrates the further compression of the example sequences using a three-level description of the same Markov trajectory in 106 bits, L = 1.12 bits per step. Similarly to lexical complexity, we are measuring the complexity of a text, which depends on sequence length, the number of subunits, regularities between these subunits, and the number of hierarchical levels. It is important to emphasize that our strategy does not depend on sequence length alone since, for example, if we replicate the sequence in Fig 1 many times and increase its length from hundreds to thousands of characters, its description under our scheme per transition step will be very similar, around 1.12 bits per step.

**Algorithm 1 Finding Subunits of a Sequence**

1: Input S: sequence of symbols

2: Build initial Markov Chain network from the sequence

3: Compute stationary distribution of the Markov chain

4: Calculate initial description length of S

5: **while** there is improvement on code length **do**

6:  # Phase 1: Local movement

7:  **while** there is movement (moved == true) **do**

8:   moved = false

9:   **for** each symbol in S **do**

10:    Consider moving the symbol to one of the neighboring subunits

11:    **if** moving symbol to another subunit decrease the code length **then**

12:     complete the movement

13:     moved = true

14:    **end if**

15:   **end for**

16:  **end while**

17:  # Phase 2: Subunit aggregation

18:  Build a new sequence S’ where its atomic symbols are the subunits

19:  Apply Phase 1 to the new sequence S’

20: **end while**

21: Output the best partition

The pseudocode of our greedy algorithm is shown in Algorithm 1. Given a sequence of symbols as input, we first build the initial Markov chain network where each node is a symbol and links are the transition probabilities between them. We then compute the stationary distribution of the Markov chain using the power method and compute the initial description length. Our greedy optimization algorithm has two phases that are repeated iteratively. In the first phase, we start from an extreme case where each symbol has its own subunit in the sequence, such that the number of symbols equals the number of subunits. This extreme case yields the maximum possible code length in our two-level description. Within a for-loop, the algorithm chooses a symbol from the sequence in random sequential order and computes the gain in total entropy by removing it from its subunit and merging it with one of the neighboring subunits or leaving at alone. This process is applied repeatedly in a 'while' loop until there is no movement between subunits; therefore, a symbol can be considered to move between subunits several times. This completes a first phase. In a second phase, we build a new sequence whose atomic symbols are now the subunits found in the first phase. Ergodic node size visit frequencies and flow sizes between nodes are aggregated, and flow conservation is satisfied in the new sequence and its corresponding new Markov chain network. The subunits are converted to atomic symbols and become nodes of the network at a higher level. When this second phase is completed, we reapply the first phase of the algorithm to the new sequence. These two phases will continue until there is no positive gain in entropy; thus, our algorithm reveals the hierarchical subunit structure of the sequence with multiple levels. The running time complexity of our algorithm depends on the structure of the sequence and how fast the first phase converges groups of symbols into subunits. If there are no pathological cases such as the pendulum effect during the movement of symbols, the worst-case running time complexity of the algorithm is *O*(*n*^2^); i.e., quadratic in the number of symbols within the sequence. To avoid an infinite loop due to pathological cases, we put a limit on the number of iterations in the outermost loop, and also a small epsilon threshold on the improvement of code length.

## Results: Comparing sequences of different complexity and origin

Many obscure and easily recognized sequences harbor information that can be difficult to unpack in certain settings. Writing is a highly organized human ability that allows humans (and now computers) to express and communicate, e.g., ideas, knowledge, attributes objects have, and the timelines and significance of events in nature. The sequence of symbols used is highly variable as is easily recognized from comparisons across different languages. More broadly, the symbols within a sequence or message are often highly variable; for example, nucleotides in DNA sequences used by cells to store and express genetic information. Efficient and succinct communication is one of the goals of good writing. We believe that the information-theoretic approach we propose can characterize and reveal the essential building blocks or ingredients, as well as the hierarchical organization associated with, sequences that convey information. To showcase our method we compare several texts of varying difficulty from a lullaby to entry-level to mid-level children's' books, and also several protein sequences.

Claude Shannon once proposed that the entropy of the English language is between 0.6 bits and 1.3 bits per character, assuming that all written English corpora were taken into account [37]. In our method, we compute the entropy per transition step using only the input sequence. Table 1 shows the code length per transition step for sequences of varying complexity and origin. We tested our method on four natural language texts with increasing lexical complexity, a lullaby and three children's books. We started from the ’pabiku’ lullaby which is generated by Saffran et al. and tested on 8-month-old babies for early language acquisition [3]. Dr. Seuss wrote ‘Green Eggs and Ham’ using just 50 distinct monosyllabic words, and he intentionally worked on the statistics of the book to achieve repetition and rhythm for early readers. The ‘Cat in the Hat’ is another book by Dr. Seuss written using 236 distinct words and also aims to achieve repetition and rhythm. ‘Midnight on the Moon’ is a mid-level children's book for second grade readers. Using our two-level description that highlights only words, we find that the code length of these four sequences are 1.30, 1.64, 2.10, and 2.30 bits per transition step, respectively. Our method also demonstrates the relative complexity of these sequences as reflected in Table 1.

**Table 1:** Comparison of the bits per transition step for different sequences.

Sequence	sequence length	2-level code length	3-level code length	4-level code length
1. pabulin lullaby	72	1.30	0.99	
2. Green Eggs and Ham	2452	1.64	1.24	0.91
3. The Cat in the Hat	5097	2.10	1.40	0.96
4. Midnight on the Moon	23075	2.30	1.55	1.03
5. Human Insulin CDS	330	1.53		
6. Human Lysozyme CDS	444	1.62		
7. Human Myoglobin CDS	462	1.65		

For each sequence, the table shows the total sequence length, and the code length for different description levels. Code length is the average bits per transition step to describe the sequence.

We can compress sequences further using multiple-level descriptions and intermediate level subunits. We applied three and four levels of descriptions to the children's books using atomic symbols (i.e., letters), words, sentences, and paragraphs. We find the code length of ‘Green Eggs and Ham’ as 1.64, 1.24, and 0.91 bits per step for two, three, and four level descriptions, respectively. Table 1 shows the code length of other sequences at multiple levels. Additionally, we applied our method to the coding DNA sequence (CDS) of three proteins: Human Insulin, Lysozyme, and Myoglobin. These proteins illustrate increasing functional complexity, from simple to medium to complex. Table 1 shows the number of bits per step to describe their sequence using two-level descriptions. The first level encodes the information flow between the nucleotides within the amino acids, and the second level encodes the information flow between the amino acids. In this manner, we can describe their sequences using 1.53, 1.62, and 1.65 bits per step for Human Insulin, Lysozyme, and Myoglobin, respectively. There is a correlation between their structural and functional complexity and the number of bits per step that describes their sequence using our method. There are likely many exceptions however, as there are many factors that affect the complexity of a protein, such as repetitions, subunit structures, and others.

We discuss the compression limits for the upper and lower bounds and compare our method with standard baseline compression methods in Table 2 for various sequences with different lengths and numbers of unique symbols. For a given sequence, the upper bound limit on the compression equals the logarithm of the number of unique symbols in that sequence. For example, there are 12 unique symbols in the ’pabiku’ lullaby and in the worst-case scenario the theoretical upper limit for the compression of its sequence is *log*_2_(12) = 3.58 bits per symbol. If we assume that the sequence is generated by a Markov chain process, then the entropy of that Markov chain would be equal to the theoretical lower limit of compression for this assumption, and it is 1.22 bits per symbol for the ’pabiku’ lullaby. Lempel-Ziv is a lossless compression algorithm which keeps history of repeated patterns and reuses them in future occurrences [38,39]. There are two popular variations of Lempel-Ziv algorithm called Gzip and LZMA. Gzip uses a shorter sliding window and a smaller dictionary, so its compression speed is fast, whereas LZMA uses a longer sliding window and a larger dictionary, so its compression speed is slow, but LZMA achieves better compression ratios than Gzip. However, their power can be observed in larger files as their compression rates are better than the first-order Markov chain entropy for ’Midnight on the Moon’ such that LZMA achieves 3.26 bits/symbol, whereas MC Entropy is 3.46 bits per symbol. Table 2 also shows the theoretical lower limit of compression based on our method, which performs better than others.

**Table 2:** Comparison with baseline methods.

Sequence	Length	N	U.B.	MCE	Gzip	LZMA	2-L	3-L	4-L
1 pabiku lullaby	72	12	3.58	1.22	9.22	12.00	1.30	0.99	
2 Green Eggs and Ham	2,452	21	4.39	2.40	2.00	2.08	1.64	1.24	0.91
3 The Cat in the Hat	5,261	24	4.58	3.16	3.47	3.56	2.10	1.40	0.96
4 Midnight on the Moon	23,075	26	4.70	3.46	3.39	3.26	2.30	1.55	1.03
5 Human Insulin CDS	330	4	2	1.79	4.19	5.43	1.53		
6 Human Lysozyme CDS	444	4	2	1.92	4.00	5.19	1.62		
7 Human Myoglobin CDS	462	4	2	1.87	3.90	4.92	1.65		

For each sequence, the table shows the length of the sequence, the number of unique symbols (N), and the upper bound (U.B.) on the compression limit (max = log(N)). MCE is the entropy of the first-order Markov chain for the sequence. For Gzip and LZMA, we find the number of bits/step by multiplying the file size with 8 (1byte = 8bits) and divide it by the length of the sequence. Our method with two-level (2-L), three-level (3-L), and four-level (4-L) descriptions.

Table 3 shows the comparison of our method with another method based on an arbitrary-order Markov chain models, and suggests a unified statistical framework for finding community structure in temporal networks and sequences [10]. The method models the data as a sequence of tokens using higher-order Markov chains with dynamic community structure. To avoid overfitting and select the right model, it utilizes a nonparametric Bayesian inference framework based on the MDL principle. Using this method, fourth-order Markov chain model gives the shortest description length for the complete book of ’War and Peace’ with 2.35 bits per symbol, as shown in Table 3. We also compress the complete book of War and Peace using LZMA (Lempel-Ziv Markov chain Algorithm) which achieves a compression rate of 2.51 bits per symbol. Their result is very close that of LZMA (Lempel-Ziv Markov chain Algorithm), because both methods are based on higher-order Markov chain models. One of them finds the theoretical lower limit of compression based on higher-order Markov models, whereas LZMA implementation achieves this compression in practice. For the same book, our method finds the theoretical lower limit of compression as 1.28 bits per symbol, based on our five-level hierarchical Markov chain model and Markov trajectories. We use the natural partitioning by the author of the book, with five-levels that contains 2,514,272 symbols, 565,762 words, 38,971 sentences, 11,725 paragraphs, and 365 chapters. Our method cannot determine the optimal solution or the true theoretical lower limit of compression for a given sequence. In fact, no method can determine the true theoretical lower limit, but Shannon estimated the entropy of English to be between 0.6 and 1.3 bits per symbol [37]. Current state-of-the-art compression algorithms such as LZMA utilize the theory of higher-order Markov chain models, but remain beyond behind the compression limit estimated by Claude Shannon. Our method represents a lower theoretical limit than other methods achieve. Therefore, we can conclude that methods based on hierarchical Markov chain models demonstrate a methodological advance over methods based on higher-order Markov chain models for compression. We show that it is theoretically possible to compress the chosen text sequences beyond the current state-of-the-art LZMA algorithm. Future compression algorithms implementing multi-level hierarchical Markov chain models could thus surpass current methods such as LZMA.

**Table 3:** Comparison on the complete book of War and Peace.

War and Peace	
Length (total symbols)	2,514,272
N (number unique symbols)	26
**Baseline methods**	
Max upper bound =log(N)	4.70
MC Entropy	3.59
gzip	3.11
LZMA	2.51
Arbitrary-order Markov chain (n = 4)	2.35
**Our method**	
two-level code length	3.05
three-level code length	2.14
four-level code length	1.89
five-level code length	1.28

The complete book of War and Peace corresponds to a sequence with an alphabet of size N = 26 (excluding space, punctuation, special symbols and then lowercase all letters) and a total length of 2,514,272 symbols.

## Discussion

We developed a new method that exploits the optimization of an objective function to uncover the hierarchical Markov sources that generate a sequence. Previous work has used arbitrary-order Markov chains to model sequences [10], whereas our work leverages Markov trajectories to model a sequence and the mechanics behind those trajectories that lead to patterns within that sequence. Our method does not partition a network representation of the sequence but rather partitions the sequence and finds relevant subunits reflecting patterns by compressing the information flow on inferred Markov sources. The method basically finds the most likely Markov sources based on patterns among the atomic elements, subunits, units, etc. at different levels of a hierarchy that generated a sequence. It is based on the transition probabilities between these elements, subunits, and units, and not their frequencies. Our method does not encode the nodes of the Markov chain, but rather only encodes the links of the Markov chain (i.e., the transitional probabilities between the nodes). One way of framing our method is to note that sequence ‘dynamics’ reflect the relationship between motion (i.e., the flow of information) within a system and exploring these relationships is different than exploring forces that may affect this motion (i.e., network structure). While partitioning the sequence in different ways to uncover specific patterns and associations between atomic elements, subunits, units, etc., we are changing the structure of a potential generative Markov Chain (the probabilities between the states) and then determining its effect on the flow of information across the sequence and defined subunits. By exploring the dynamics within each change of assumed structure, our method seeks to identify the optimal partitioning by optimizing a specific objective function. In this light, our goal is not to achieve maximal compression as in algorithms such as Lempel-Ziv, Gzip or Huffman-Coding, but rather use compression to learn and explore a generative Markov Process for the sequence. In this way our approach is consistent with Minimum Description Length (MDL) principles for which learning can be represented as a data compression problem [26].

Recent advances in deep learning have applications to many areas with different types of neural network models behind, e.g., Large Language Models (LLMs). These models are rooted in predicting symbols in a sequence based on patterns in earlier sequences, such as sequence-to-sequence models, transformer-based models, convolutional and other models. AlphaGenome, for example, is a sequence to function model, which has similar goals to our method [40]. AlphaGenome is trained on known genomic elements (i.e., defined stretches of DNA sequence) with particular biological functions to learn how to map the sequences to their functions. AlphaGenome is an ’all in one’ approach to interpret DNA sequences, since it is trained by all known structural and functional DNA elements of a genome with their corresponding DNA sequences. Based on how AlphaGenome was trained, it can predict functions of complicated non-coding sequences with some probability. Although explaining the inner workings of generative models and multi-layer neural networks is an ongoing area of research, it is clear LLMs and neural networks exploit regularities and the transition probabilities between tokens of interest to predict the next token or stretch of tokens. Unlike standard neural nets and LLMs, our method is an explainable and unsupervised method which seeks to predict the possibly hierarchical subunit structure of a sequence harboring information. Although our method has potential, future work will need to consider its application to larger texts, whole genome sequences, and many other types of sequences.

## Supporting information

S1 File Supporting information includes supporting text, S1 Fig, S1 ti S7 Datasets, and SI references. (PDF)
