# Supplementary material for: Trajectories of random walks on Markov sources reveal the structure of complex sequences
Source: PLoS One. 2026 Nov 11;21(11):e0359402. doi: 10.1371/journal.pone.0359402 (PMC13754223; doi:10.1371/journal.pone.0359402)
Supplement: Supplementary file 1 [file pone.0359402.s001.pdf]

Supporting Information for

Trajectories of random walks on Markov sources reveal the structure of complex sequences

Ali Altunkaya, Arunabha Sen, Nicholas J. Schork

This PDF file includes:

Supporting text
Fig. S1
Datasets S1 to S7
SI References

Supporting Information Text

Here we present a detailed description of our method and the objective function. Then we explain the implementation of our method using a greedy heuristic algorithm. Lastly, we demonstrate the greedy procedure in 36 subsequent slides, which shows the step-by-step process to find shorter codelengths, by exploiting regularities within the Markov Chain source that generates the sequence.

Two-level encoding

We use i, j to enumerate subunits and α, β to enumerate symbols. Assume that the whole sequence of symbols has length l and contains n different symbols. Also assume that our partition will result in a sequence of subunits which has length k and contains m different subunits. There are many ways to partition the whole sequence into subunits. Our equation measures the total entropy of the whole sequence (a particular Markov Trajectory) according to a particular partition as below:

$$L(S) = qH(T_{whole}) + \sum_{i=1}^{m} F_i H(T_i) \quad (1)$$

This equation has two terms: first one is the entropy of whole sequence trajectory between the subunits, and the second one is the entropy of subunit trajectories between the symbols. Each one is weighted with the frequency that it occurs, or the time spent by the random walker on that trajectory.

Exit size q: To partition, we introduce a hypothetical symbol q, which indicates whether the random walker entering to a subunit or exiting from a subunit. For each subunit i, q_i is the probability of exiting from that subunit is:

$$q_i = \tau P_i(1 - P_i) + (1 - \tau) \sum_{\alpha \in i} \sum_{\beta \notin i} p_\alpha w_{\alpha\beta} \quad (2)$$

First term is the contribution from teleportation where $\tau = 0.15$, which is multiplied by the subunit size $P_i = \sum_{\alpha \in i} p_\alpha$ and $(1 - P_i)$. And second term is the contribution from the neighboring links, which is the outFlow size of the last symbol in the subunit.

We are encoding the links of the Markov Chain, not the nodes. So, we need to compute the total flow size within the subunits $F_i = q_i + P_i = q_i + \sum_{\alpha \in i} p_\alpha$, and weight the entropy of subunit trajectories $H(T_i) = H(O_q) + \sum_{\alpha=1}^{k} H(O_\alpha)$ with the corresponding F_i.

$q = \sum_{i=1}^{m} q_i$ is the total exit size, which corresponds to the total time, the random walker spent between the subunits. Therefore, the entropy of trajectory between the subunits $H(T_{whole})$ is weighted with q.

Convergence of random variables: By definition, a stochastic process is an indexed sequence of random variables. Stochastic convergence means that a sequence of random events can be settle into a pattern. A certain sequence of random variables $X_1, X_2, ..., X_n$ can be converged to a new random variable Y under the Asymptotic Equipartition Property [1]. Asymptotic Equipartition Property is the analog of law of large numbers in information theory and can be applied at the stationary distribution. In our case, the sequence of transitions between the symbols settled into a pattern and converge into a new kind of random variable, as we called subunits.

For each subunit i, we compute its entropy rate using its outFlow sizes: $H(O_i) = \sum_i \frac{f_{ij}}{q_i} log \frac{f_{ij}}{q_i}$, where f_{ij} is the flow size from subunit i to j, and $q_i = \sum_i f_{ij}$ is its total outFlow size. Then the entropy of the trajectory between the subunits will be $H(T_{whole}) = \sum_{i=1}^{k} H(O_i)$.

In this way, we can reuse the same codes for encoding the outLinks of symbols and the outLinks of subunits. Because we are using a special exit code, which indicates the start and finish of a subunit. Now we explained all terms of the equation. Furthermore, we apply this method hierarchically to identify higher-level subunits at multi-levels [2–4].

Convergence of random variables can be also considered as phase transitions in Stochastic Markov process, where micro symbols converge into macro subunits, during this phase transition [5].

The local interactions between the symbols within subunits and the interactions between the subunits within the whole sequence generate system-wide dynamics within the Markov Chain network. Dynamics is the relationship between the motion (flow of information) and the forces affecting motion (network structure). While partitioning the sequence we are changing the structure of the Markov Chain (the probabilities between the states) and see its effect on the flow of information. By playing with the dynamics, we try to find the optimal partitioning.

There are two types of links between the symbols or subunits: the actual neighboring links and the teleportation links. In the smaller subunits such as symbols, syllables, words, the neighboring links have more influence because of repetitions, therefore big portion of the transition probabilities between them comes from neighboring links. In the bigger subunits such as sentences, paragraphs and sections, teleportation links have more influence, because we usually don't see repetition of the same paragraph in a book. So, a big portion of the transition probabilities between them comes from teleportation links. It can also be explained by the "Strength of Weak Links", where teleportation links are weak but occurs in great quantity.

Implementation

We developed a greedy heuristic algorithm to find the optimal partitioning by minimizing our objective function. We start from the extreme partition case where each symbol is also a subunit. The core of our optimization algorithm is very similar to the Louvain method as explained in [6]. It has two phases.

Just reminding that we are not partitioning the network, we are partitioning the sequence of symbols and adjust the network structure and information flow on the network for that particular partitioning.

In the first phase, each symbol has its own subunit in the sequence. So the number of symbols equals to number of subunits. In a for loop, the algorithm chooses a symbol in random sequential order within the sequence and computes the gain in total entropy by merging the symbols with one of the neighboring subunits: left or right. Then the symbol is merged with the neighboring subunit, which results in the highest positive gain in total Entropy. If there is no positive gain, the symbols remain in the same subunit. This iteration continues until there is no movement between subunits; therefore, a symbol can be considered to move between subunits several times. This algorithm uses a threshold to prevent infinite loop; it stops if the gain is less than epsilon.

After the first phase, subunits will become new atomic symbols of the sequence. And we apply the first phase again to detect subunits on the upper level.

References

1. Cover TM. Elements of information theory. John Wiley & Sons; 2006.
2. Lin HW, Tegmark M. Critical behavior from deep dynamics: a hidden dimension in natural language. arXiv preprint arXiv:160606737. 2016.

3. Lin HW, Tegmark M. Criticality in formal languages and statistical physics. arXiv preprint arXiv:160606737. 2016.
4. Lin HW, Tegmark M, Rolnick D. Why does deep and cheap learning work so well? Journal of Statistical Physics. 2017;168:1223-47.
5. Blythe R. An introduction to phase transitions in stochastic dynamical systems. In: Journal of Physics: Conference Series. vol. 40. IOP Publishing; 2006. p. 1.
6. Blondel VD, Guillaume JL, Lambiotte R, Lefebvre E. Fast unfolding of communities in large networks. Journal of statistical mechanics: theory and experiment. 2008;2008(10):P10008.
7. Rosvall M, Bergstrom CT. Maps of random walks on complex networks reveal community structure. Proceedings of the national academy of sciences. 2008;105(4):1118-23.

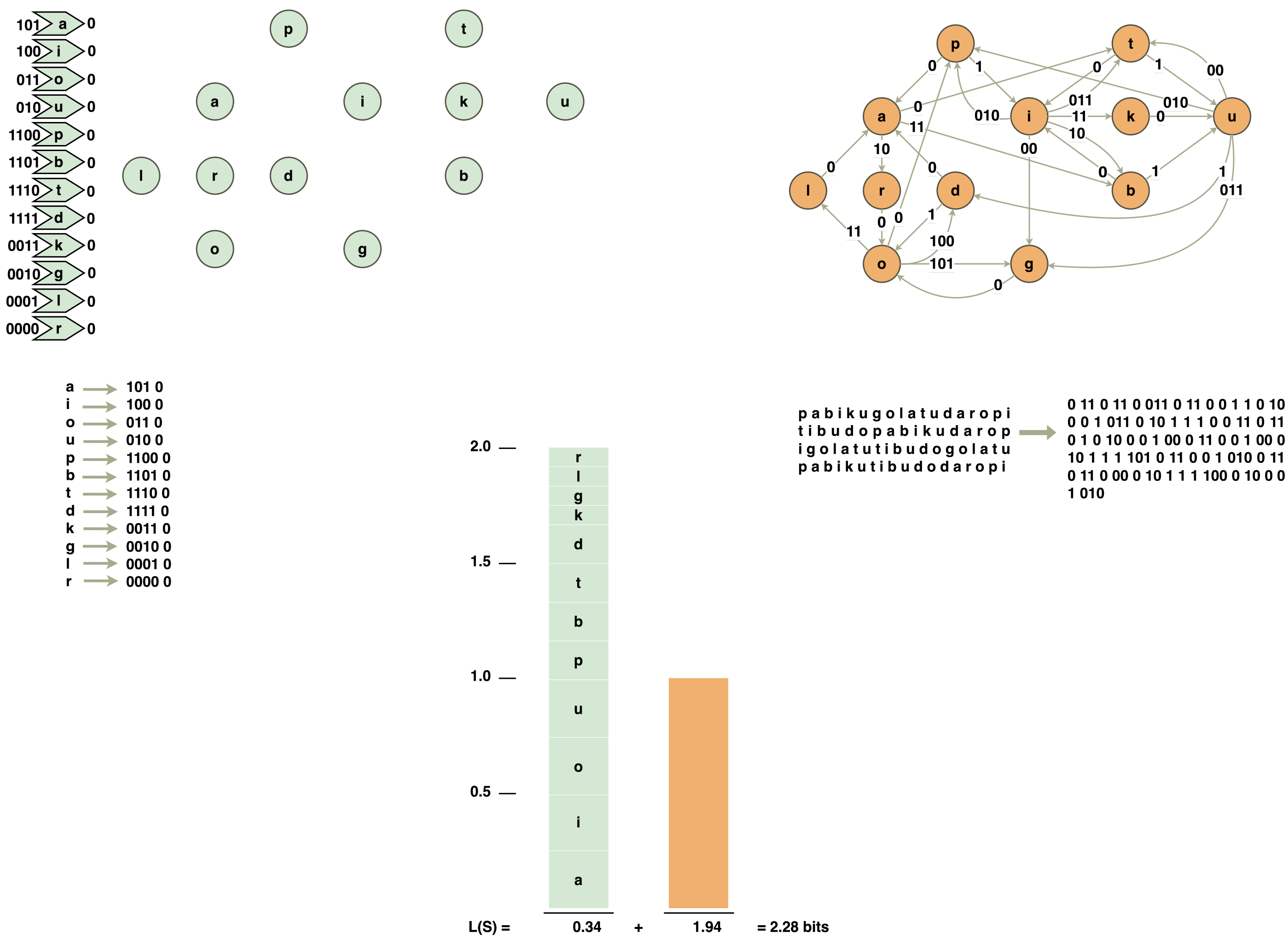

Fig 1. The greedy heuristic algorithm begins from an extreme case where each symbol is also a subunit. The left Markov Chain generates the subunits using the information flow between the symbols. The right Markov Chain generates the whole sequence using the information flow between the subunits. The slides on the following 36 pages shows how the greedy algorithm searches for partitioning the sequence into subunits by optimizing the equation; finding shorter encodings by exploiting regularities in the sequence. We follow the notation and scheme described in [7]. In this extreme case, the max rate is 2.0 because we add an extra exit symbol between each symbol. To demonstrate the duality between finding subunits and coding, the code structure to the corresponding partition is shown by the stacked boxes at the bottom. The height of each box corresponds to the per step rate of that codeword.

The left stack represents the codes associated with subunits. The height of each box is equal to the sum of ergodic node visit probabilities within that subunit, $F_i = q_i + P_i = q_i + \sum_{\alpha \in i} p_\alpha$. The length of the codeword for subunit i corresponds to the entropy of that particular Markov trajectory between the symbols $H(T_i) = H(O_q) + \sum_{\alpha=1}^{k} H(O_\alpha)$. The per step description length is the length of the codeword weighted by their rate, and normalized by their number of steps.

The right stack represents the code associates with the whole sequence. The height of the box is equal to the total exit size, $q = \sum_{i=1}^{m} q_i$, the total time random walker spent between the subunits. The length of the codeword for the whole sequence corresponds to the entropy of that particular Markov trajectory between the subunits $H(T_{whole}) = \sum_{i=1}^{k} H(O_i)$. The per step description length is the length of the codeword weighted by their rate, and normalized by their number of steps.

The total description length L(S) for the subunit partition S of the sequence is the sum of the contributions from the flow between and within the subunits. It takes minimum value on the last slide.

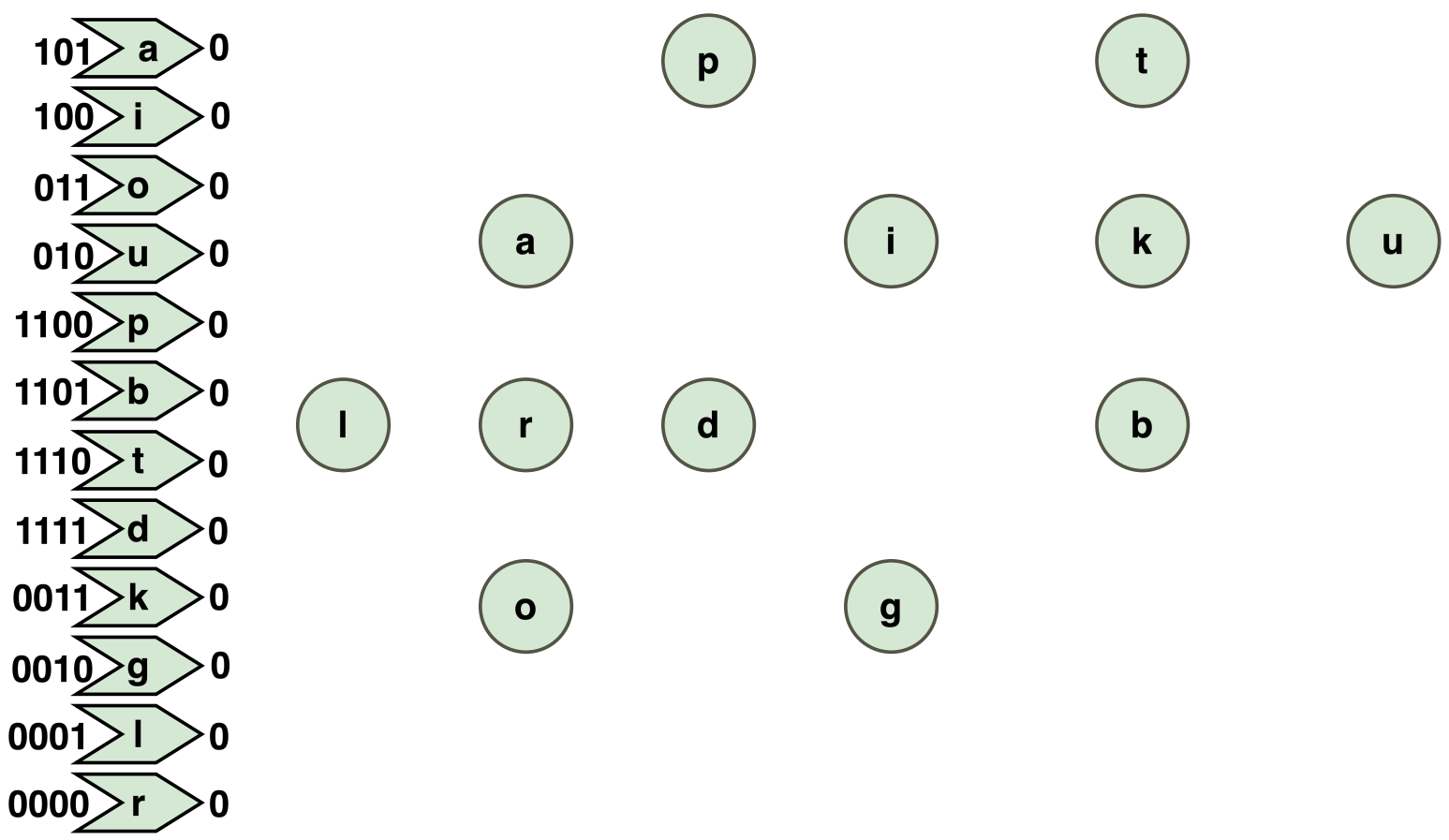

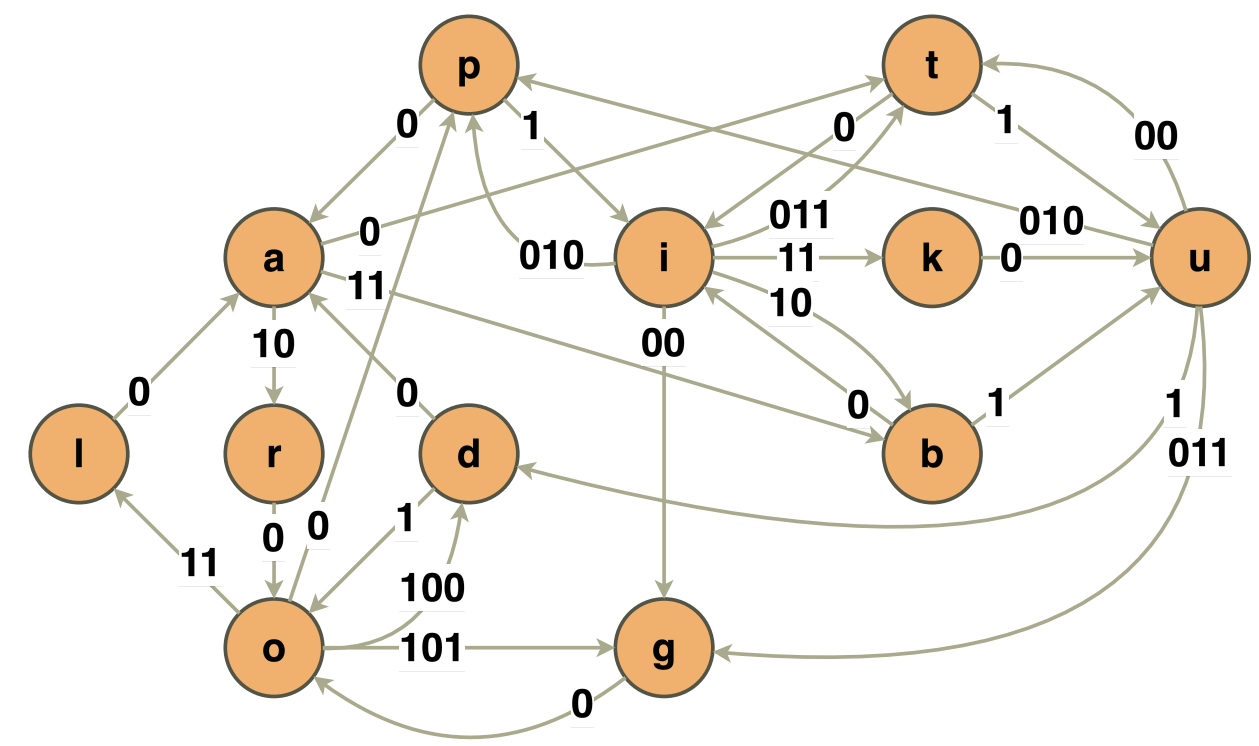

a → 101 0
i → 100 0
o → 011 0
u → 010 0
p → 1100 0
b → 1101 0
t → 1110 0
d → 1111 0
k → 0011 0
g → 0010 0
l → 0001 0
r → 0000 0

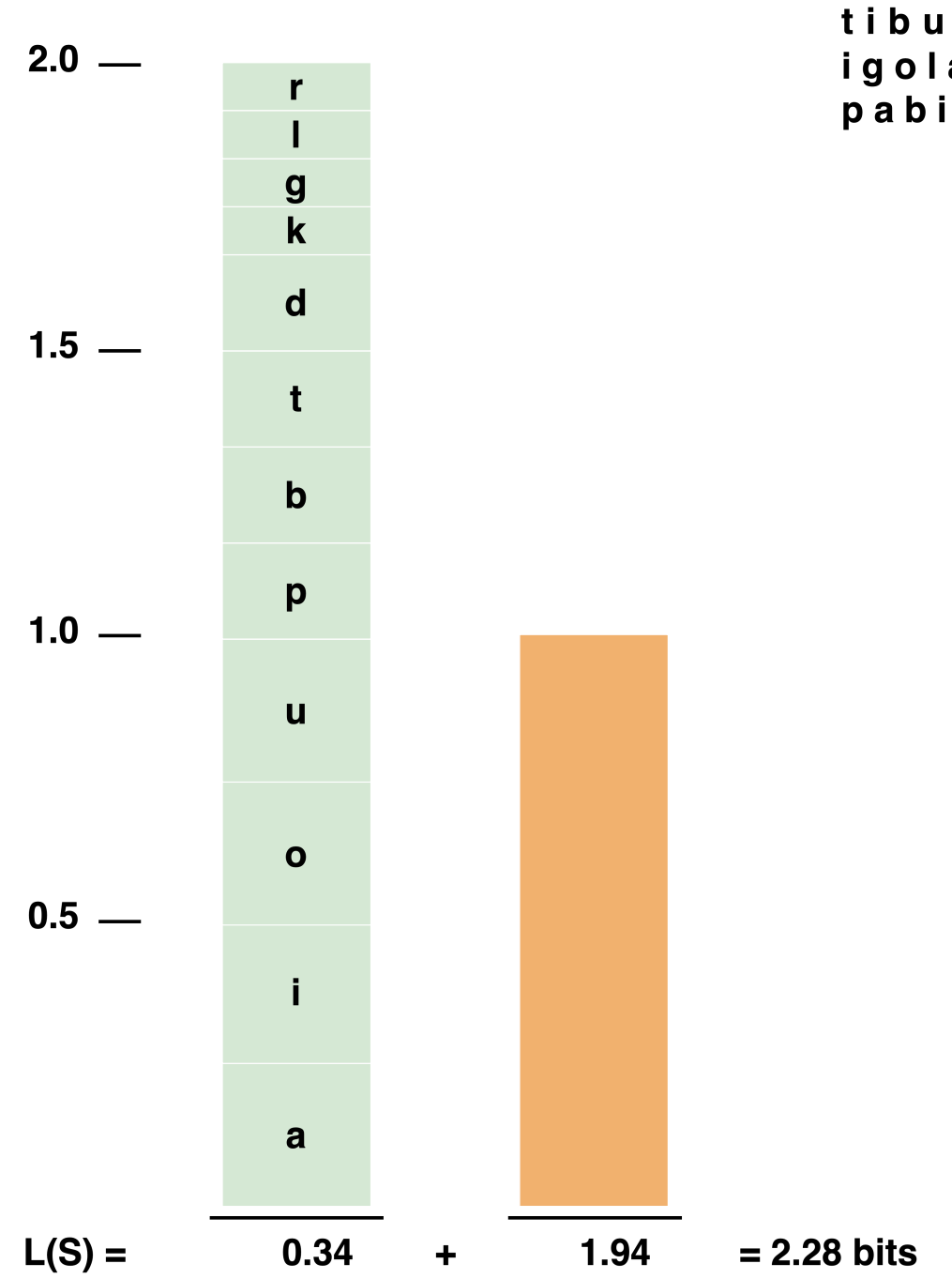

p a b i k u g o l a t u d a r o p i
t i b u d o p a b i k u d a r o p
i g o l a t u t i b u d o g o l a t u
p a b i k u t i b u d o d a r o p i

→

0 11 0 11 0 011 0 11 0 0 1 1 0 10
0 0 1 011 0 10 1 1 1 0 0 11 0 11
0 1 0 10 0 0 1 00 0 11 0 0 1 00 0
10 1 1 1 101 0 11 0 0 1 010 0 11
0 11 0 00 0 10 1 1 1 100 0 10 0 0
1 010

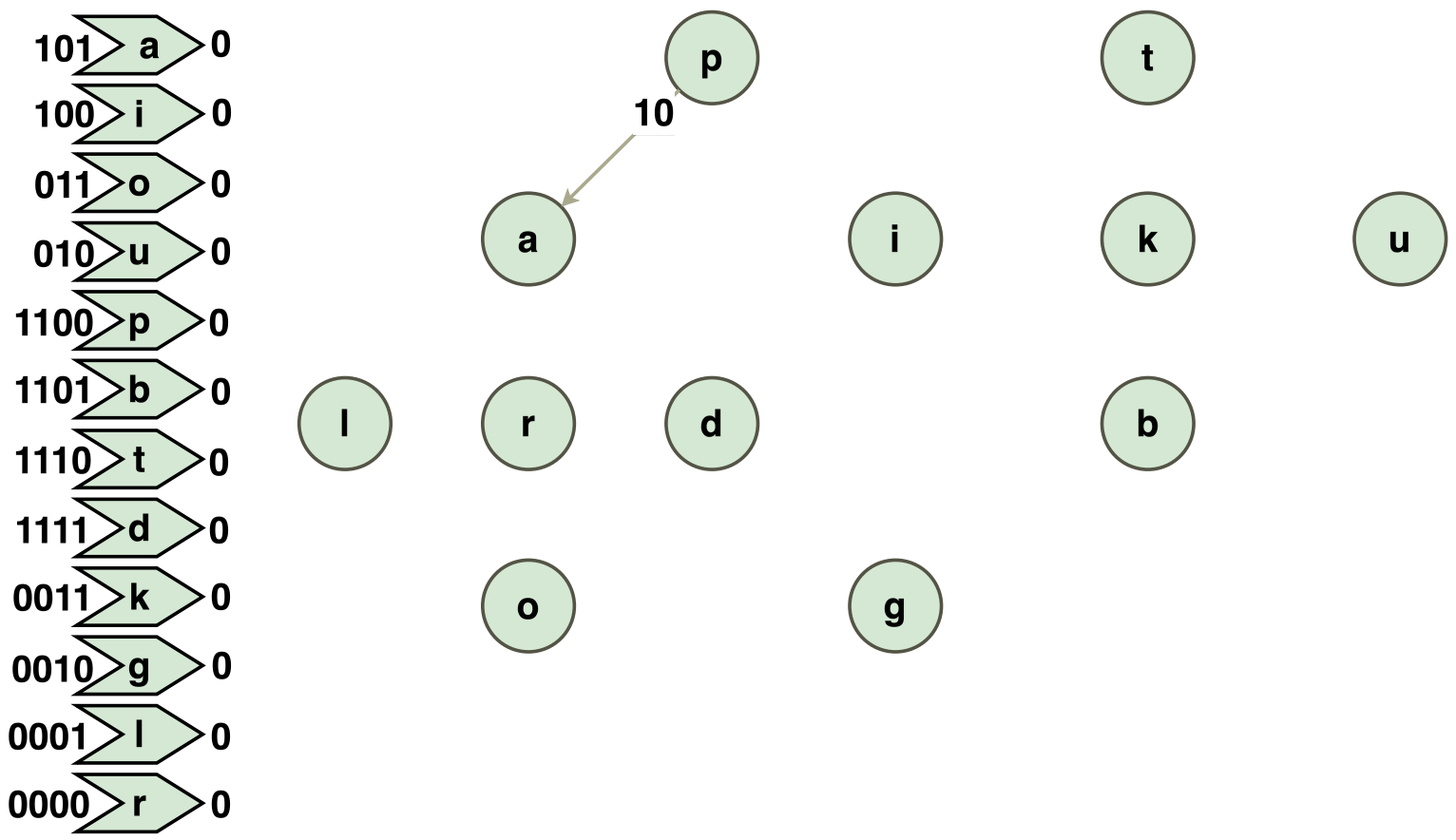

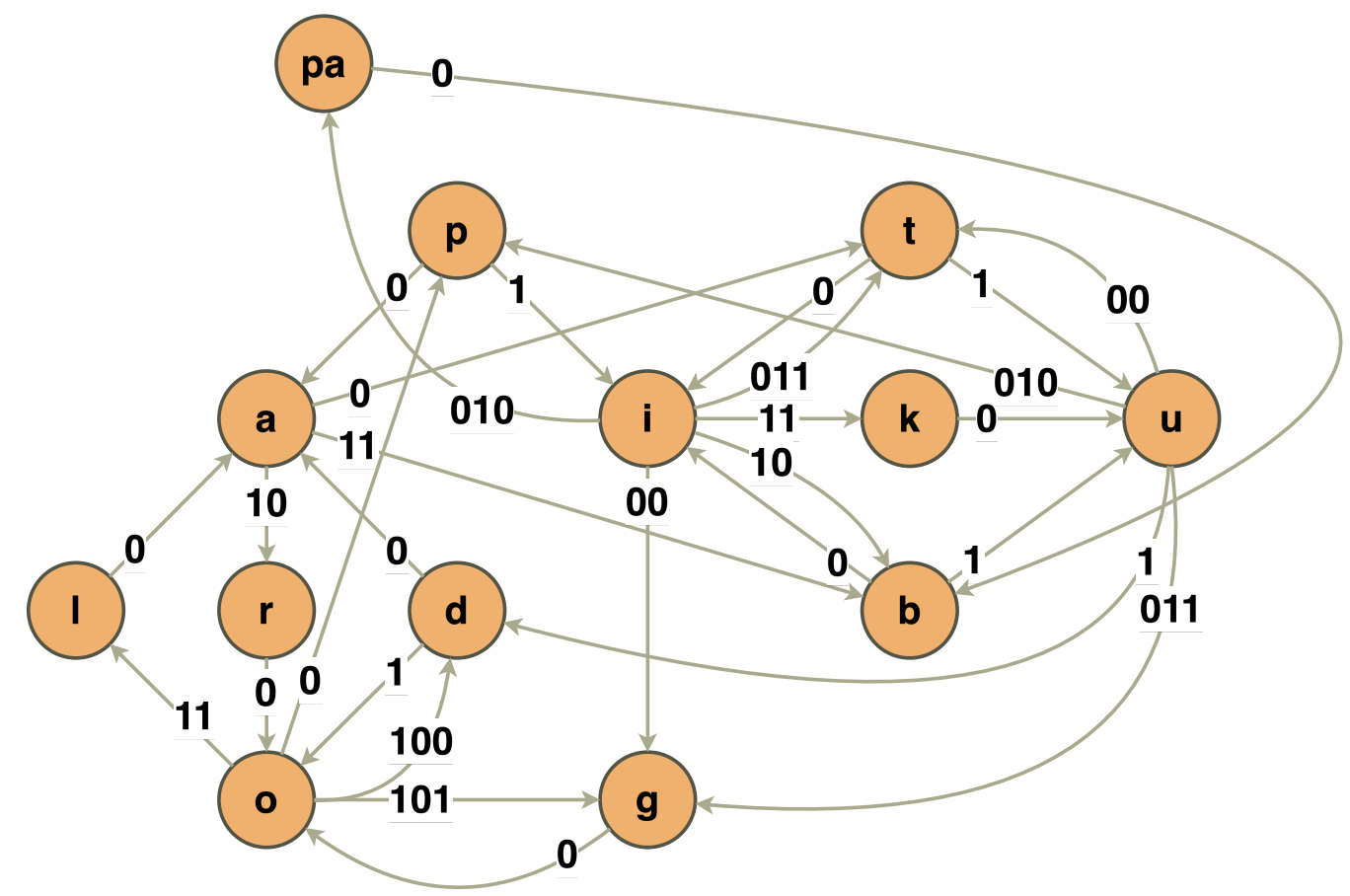

a → 101 0
i → 100 0
o → 011 0
u → 010 0
p → 1100 0
b → 1101 0
t → 1110 0
d → 1111 0
k → 0011 0
g → 0010 0
l → 0001 0
r → 0000 0

p a → 1100 10 0

pa b i k u g o l a t u d a r o p i t
i b u d o p a b i k u d a r o p i g
o l a t u t i b u d o g o l a t u p
a b i k u t i b u d o d a r o p i

→

0 0 11 0 011 0 11 0 0 1 1 0 10 0
0 1 011 0 10 1 1 1 0 0 11 0 11
0 1 0 10 0 0 1 00 0 11 0 0 1 00 0
10 1 1 1 101 0 11 0 0 1 010 0 11
0 11 0 00 0 10 1 1 1 100 0 10 0 0
1 010

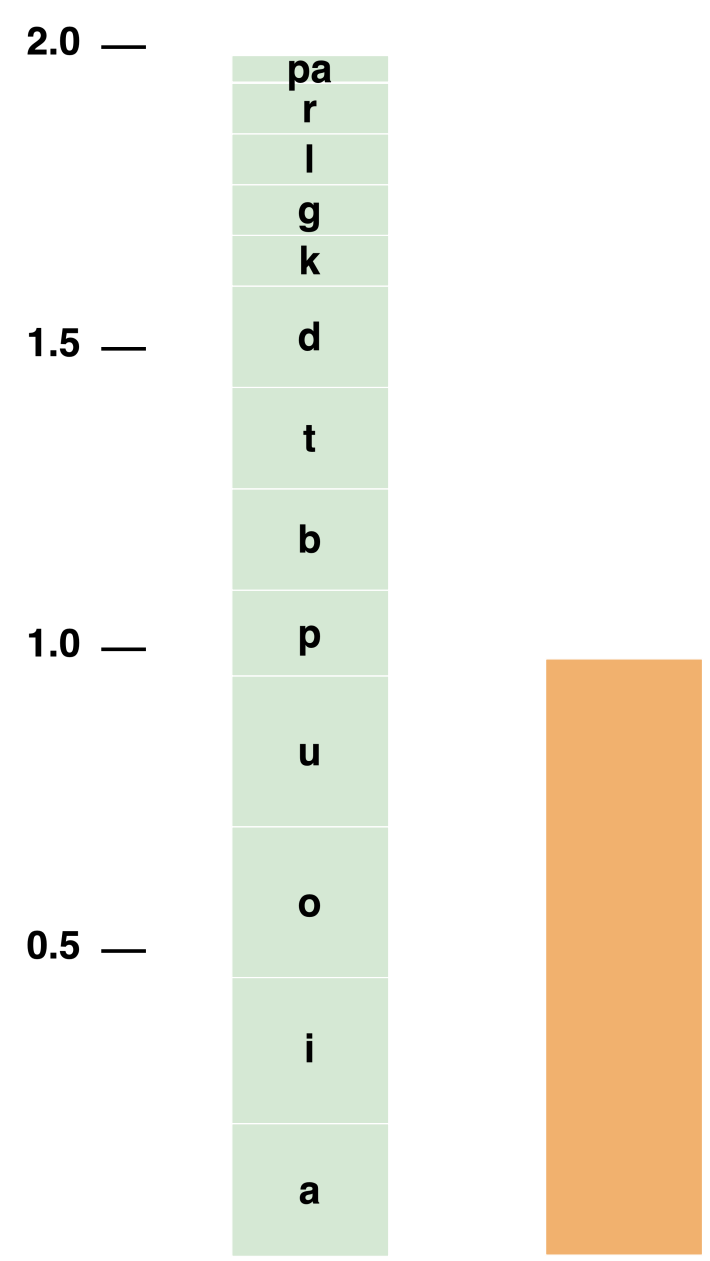

L(S) = 0.33 + 1.93 = 2.26 bits

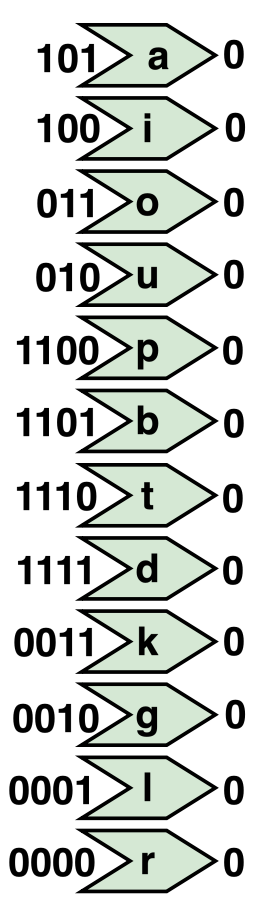

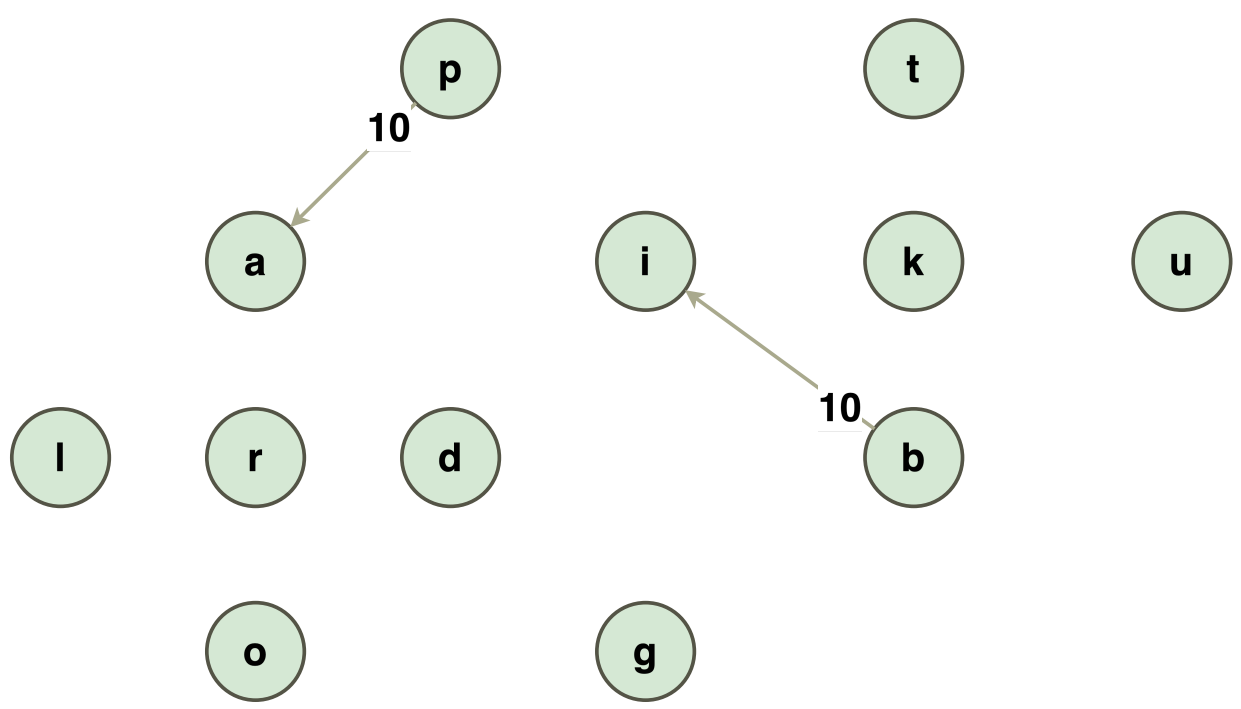

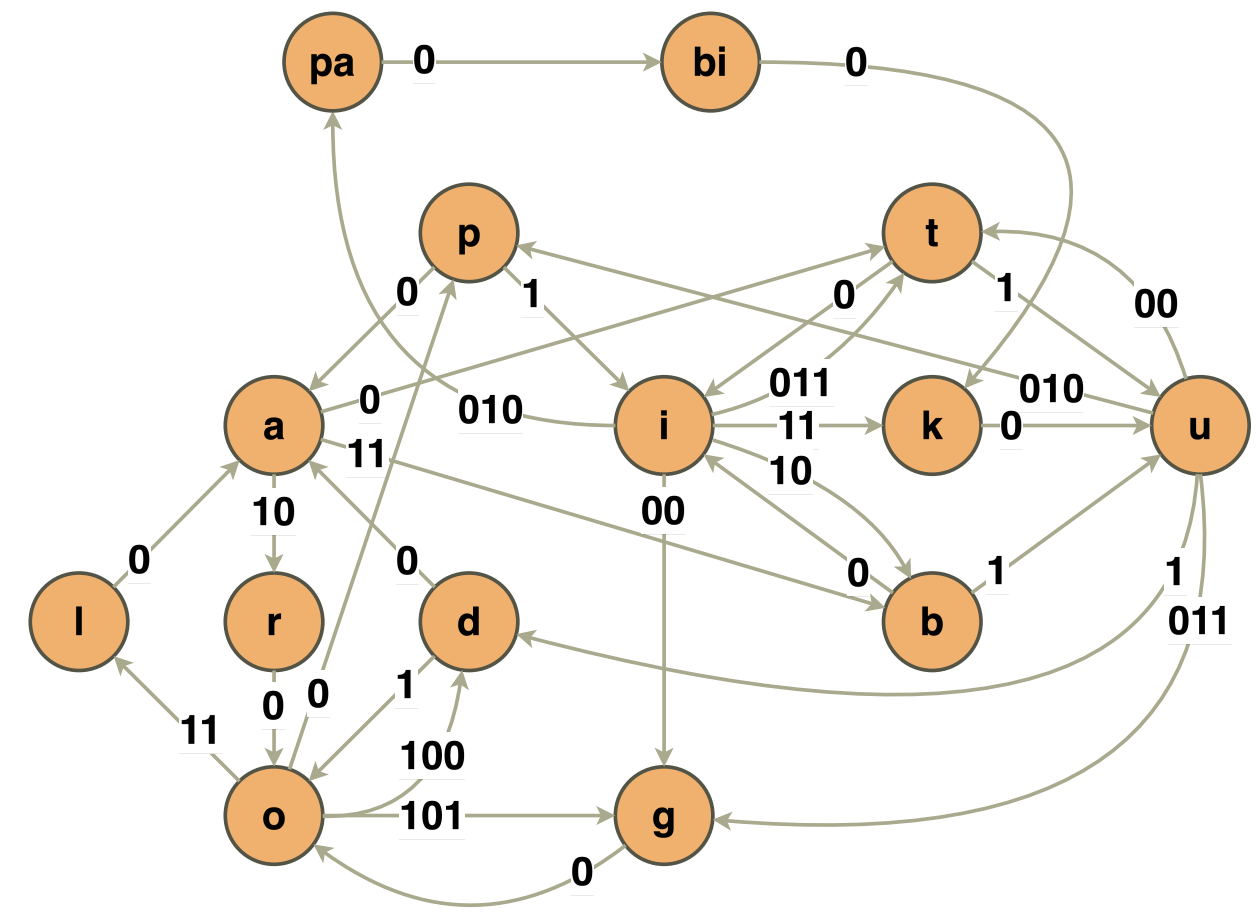

a → 101 0
i → 100 0
o → 011 0
u → 010 0
p → 1100 0
b → 1101 0
t → 1110 0
d → 1111 0
k → 0011 0
g → 0010 0
l → 0001 0
r → 0000 0

p a → 1100 10 0
b i → 1101 10 0

pa bi k u g o l a t u d a r o p i t
i b u d o p a b i k u d a r o p i g
o l a t u t i b u d o g o l a t u p
a b i k u t i b u d o d a r o p i

→

0 0 0 011 0 11 0 0 1 1 0 10 0 0
1 011 0 10 1 1 1 0 0 11 0 11
0 1 0 10 0 0 1 00 0 11 0 0 1 00 0
10 1 1 1 101 0 11 0 0 1 010 0 11
0 11 0 00 0 10 1 1 1 100 0 10 0 0
1 010

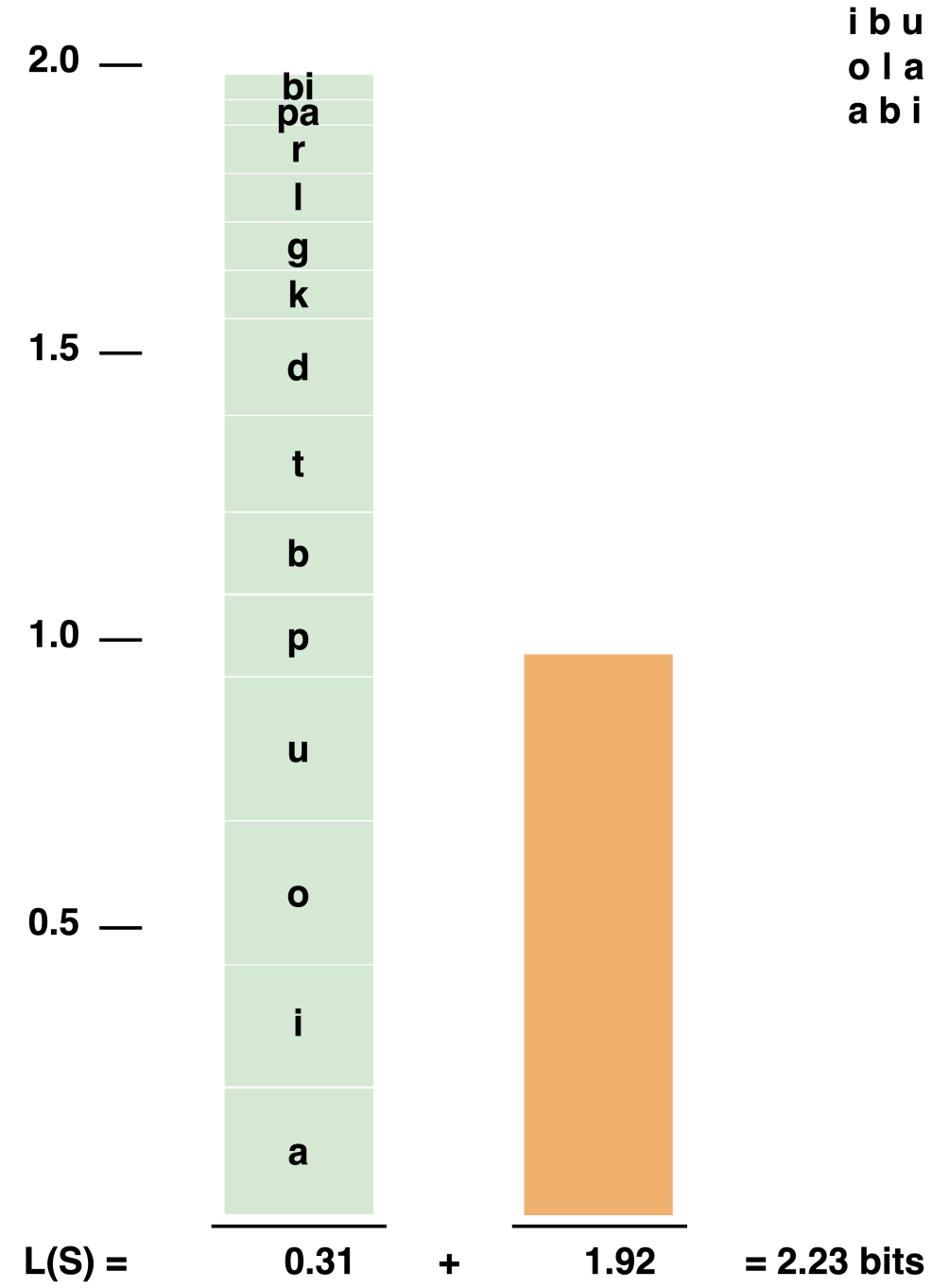

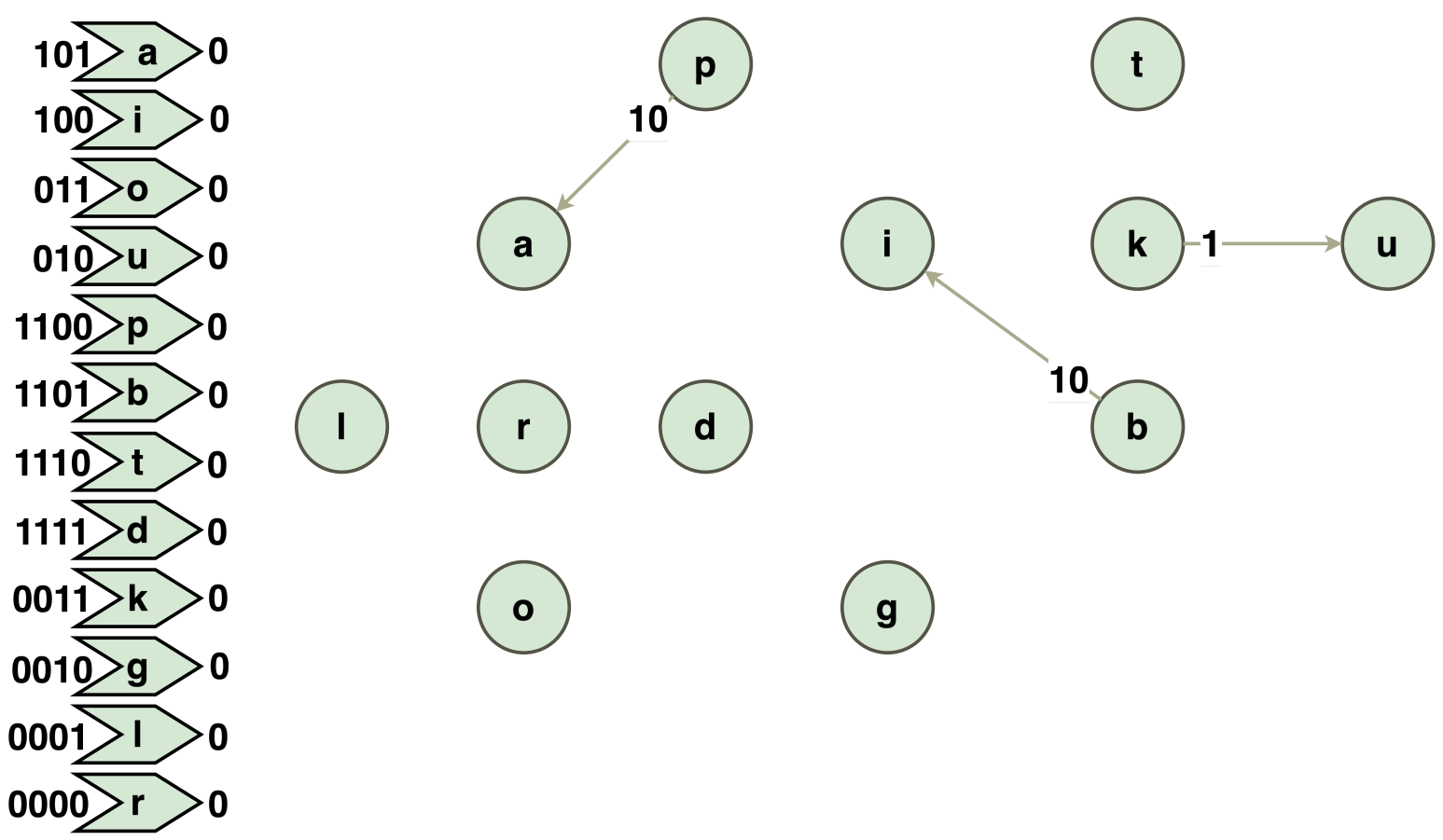

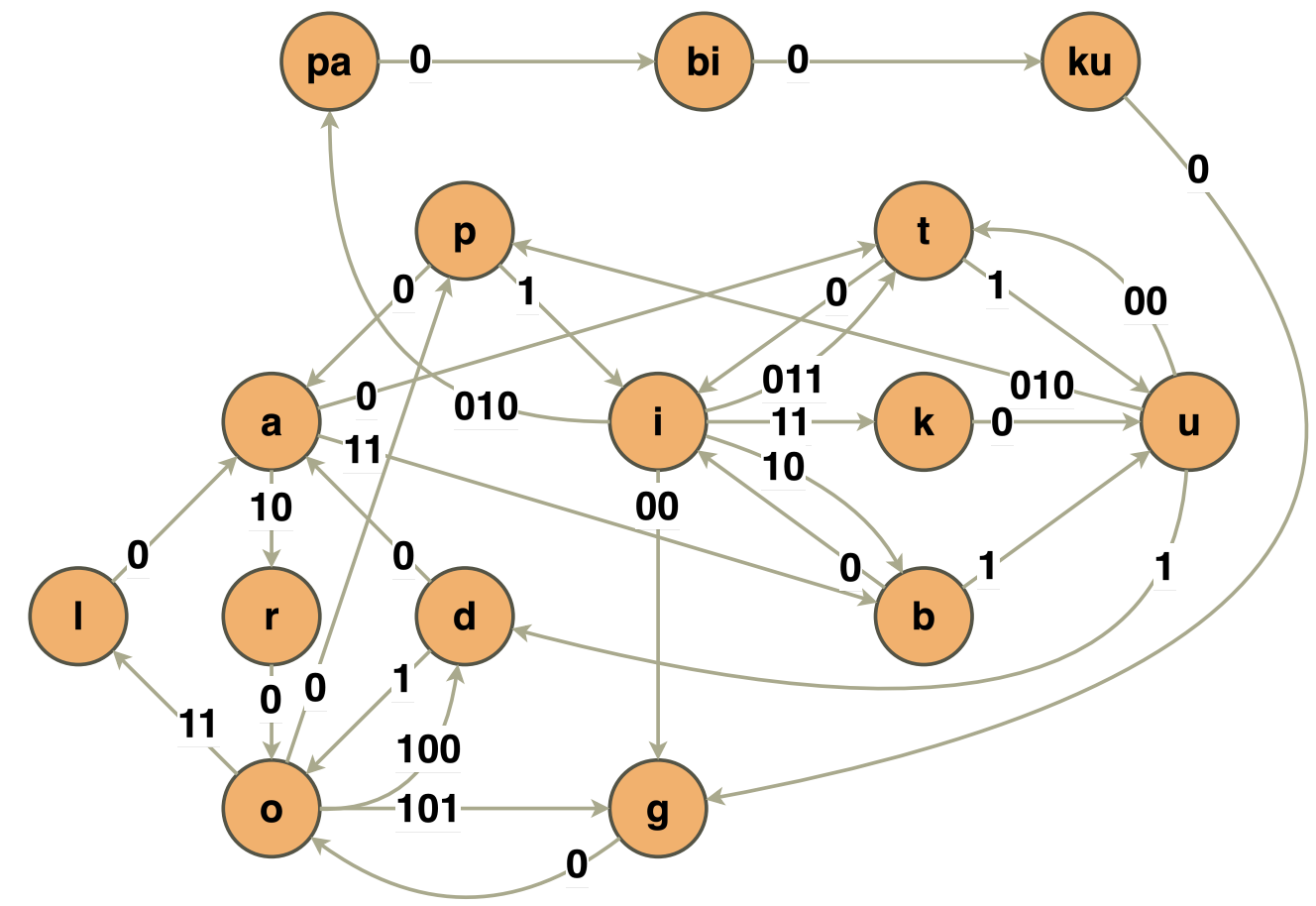

a → 101 0
i → 100 0
o → 011 0
u → 010 0
p → 1100 0
b → 1101 0
t → 1110 0
d → 1111 0
k → 0011 0
g → 0010 0
l → 0001 0
r → 0000 0

p a → 1100 10 0
b i → 1101 10 0
k u → 0011 1 0

pa bi ku g o l a t u d a r o p i t i
b u d o p a b i k u d a r o p i g
o l a t u t i b u d o g o l a t u p
a b i k u t i b u d o d a r o p i
→
0 0 0 0 11 0 0 1 1 0 10 0 0
1 011 0 10 1 1 1 0 0 11 0 11
0 1 0 10 0 0 1 00 0 11 0 0 1 00 0
10 1 1 1 101 0 11 0 0 1 010 0 11
0 11 0 00 0 10 1 1 1 100 0 10 0 0
1 010

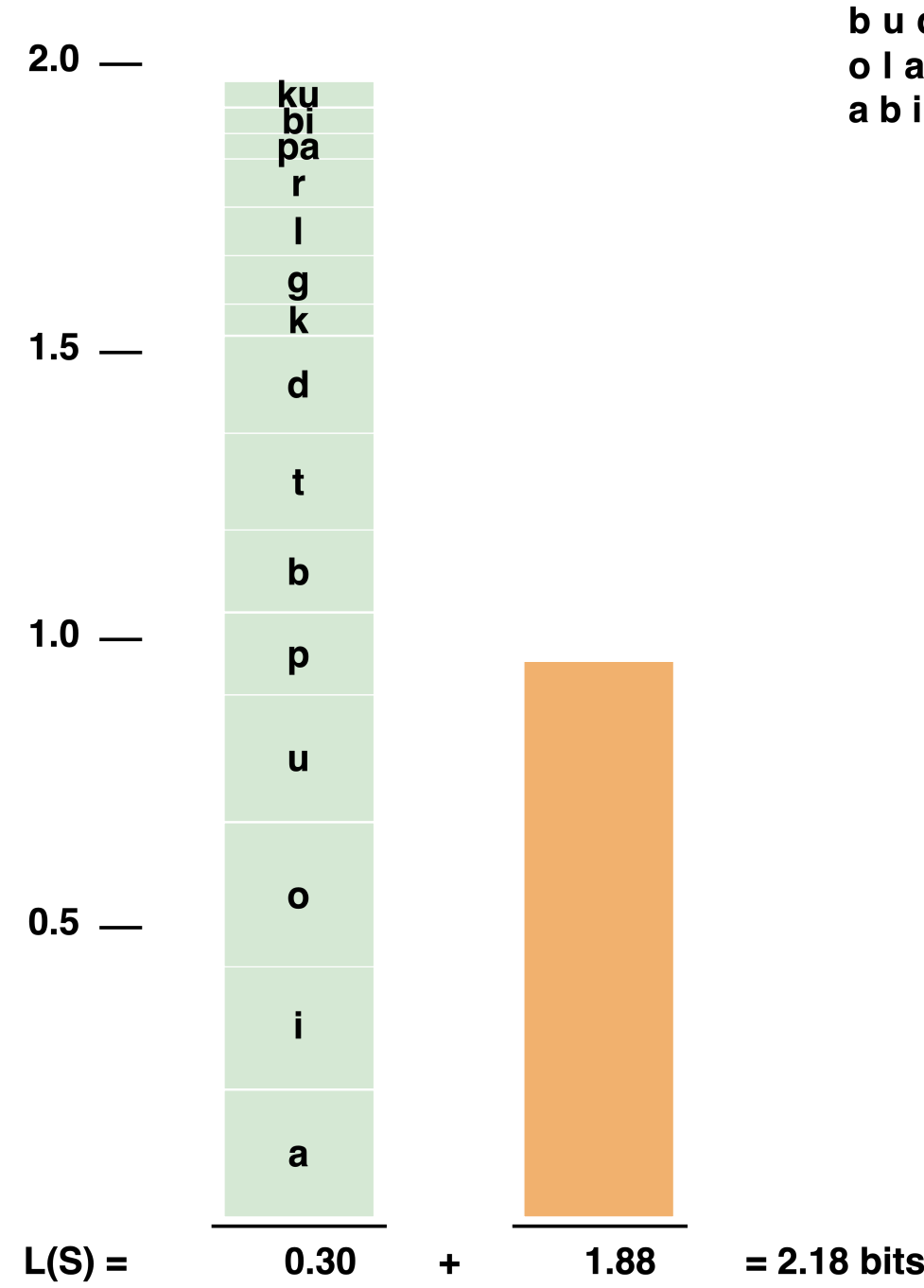

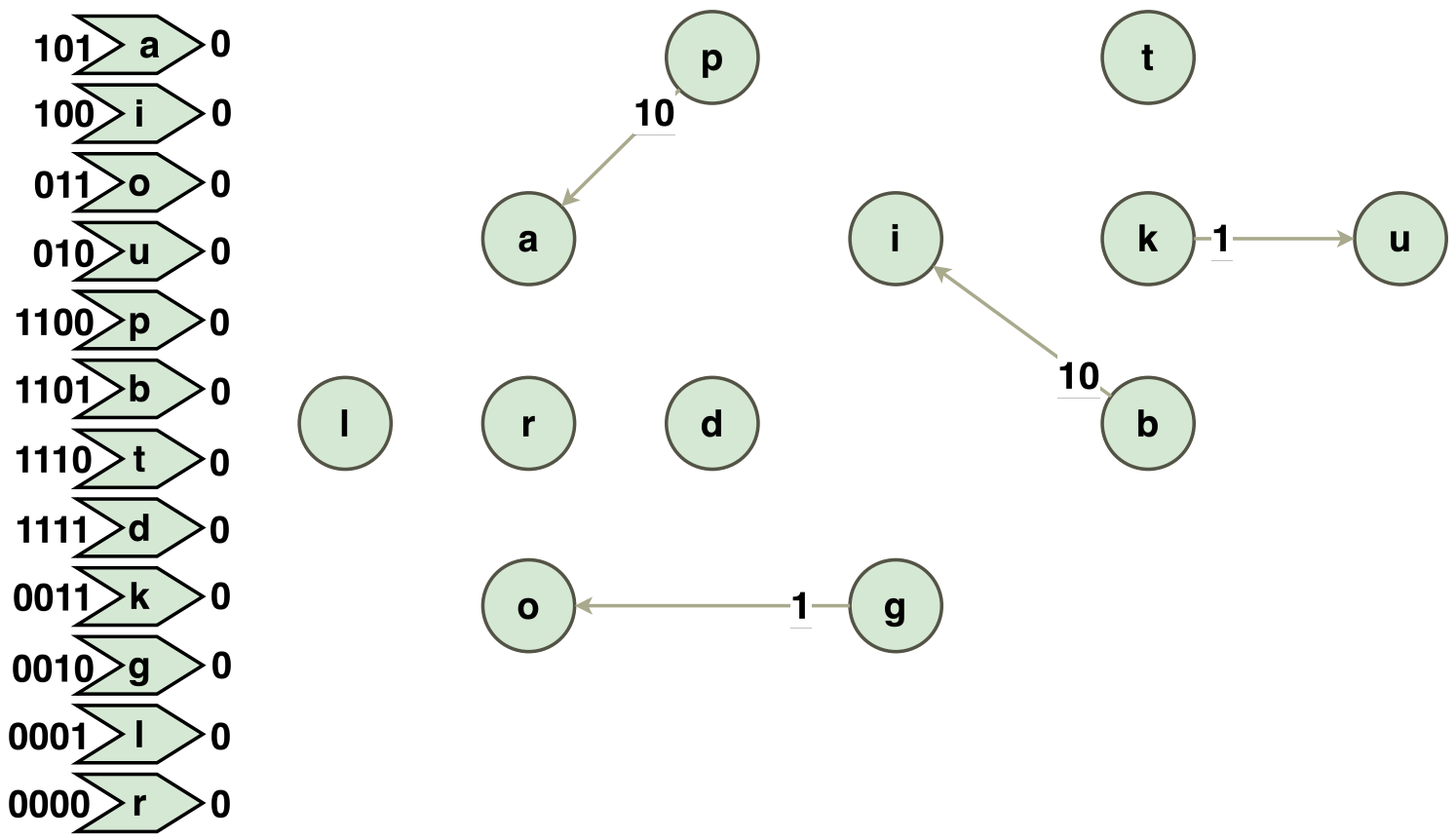

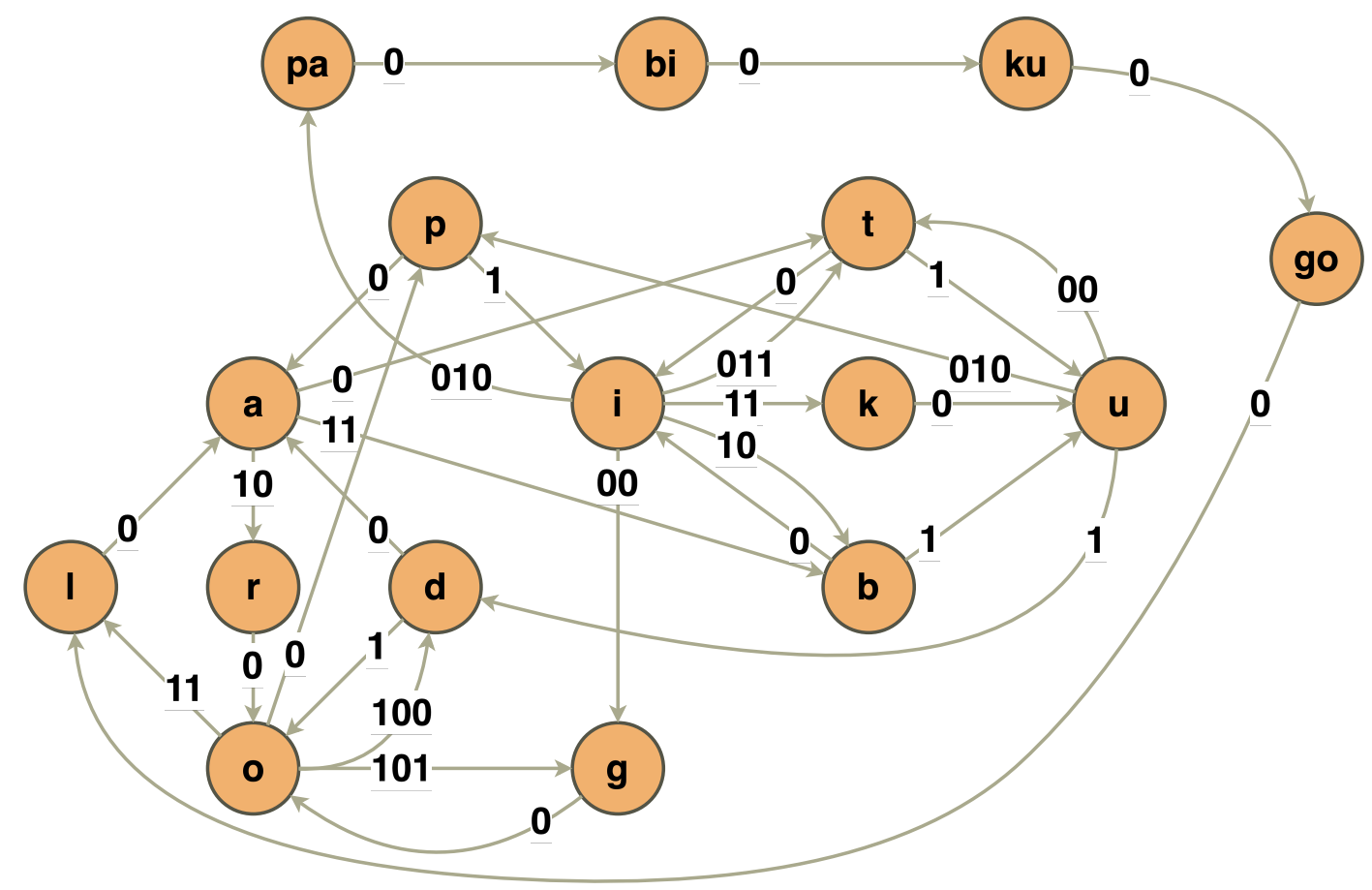

a → 101 0
i → 100 0
o → 011 0
u → 010 0
p → 1100 0
b → 1101 0
t → 1110 0
d → 1111 0
k → 0011 0
g → 0010 0
l → 0001 0
r → 0000 0

p a → 1100 10 0
b i → 1101 10 0
k u → 0011 1 0
go → 0010 1 0

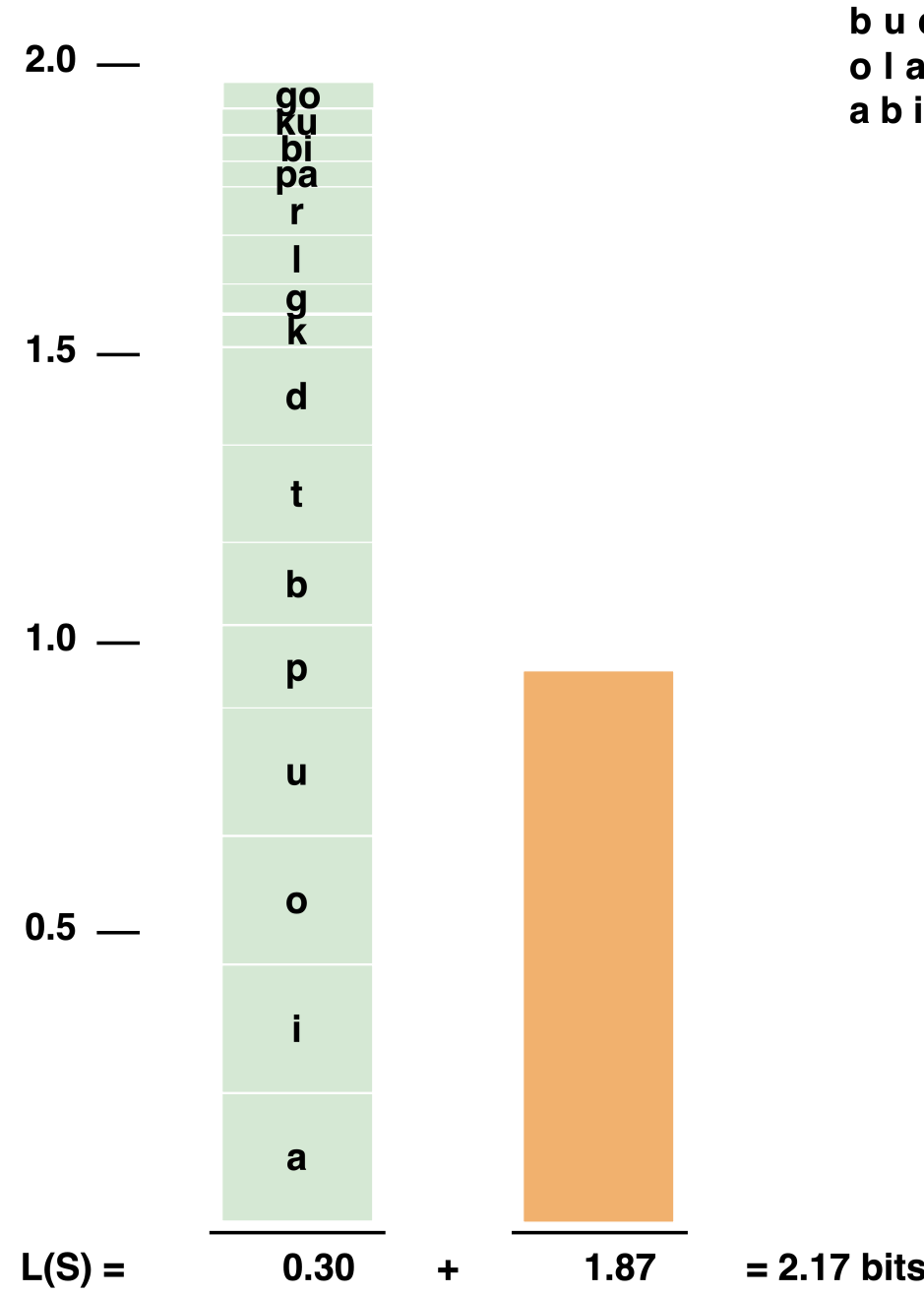

pa bi ku go l a t u d a r o p i t i
b u d o p a b i k u d a r o p i g
o l a t u t i b u d o g o l a t u p
a b i k u t i b u d o d a r o p i

→

0 0 0 0 0 0 1 1 0 10 0 0 1 011 0
10 1 1 1 0 0 11 0 11 0 1 0 10 0 0
1 00 0 11 0 0 1 00 0 10 1 1
1 101 0 11 0 0 1 010 0 11 0 11
0 00 0 10 1 1 1 100 0 10 0 0
1 010

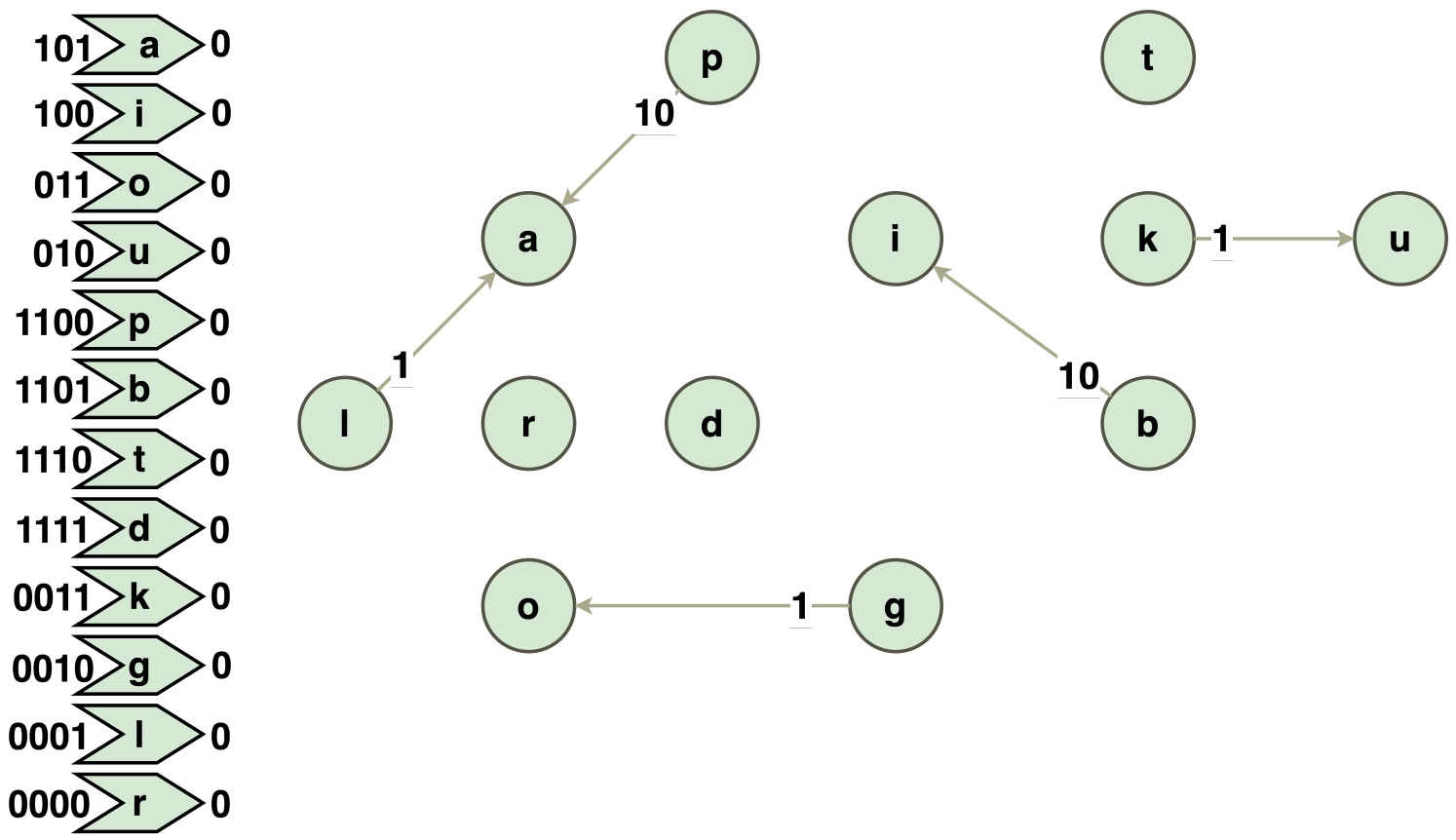

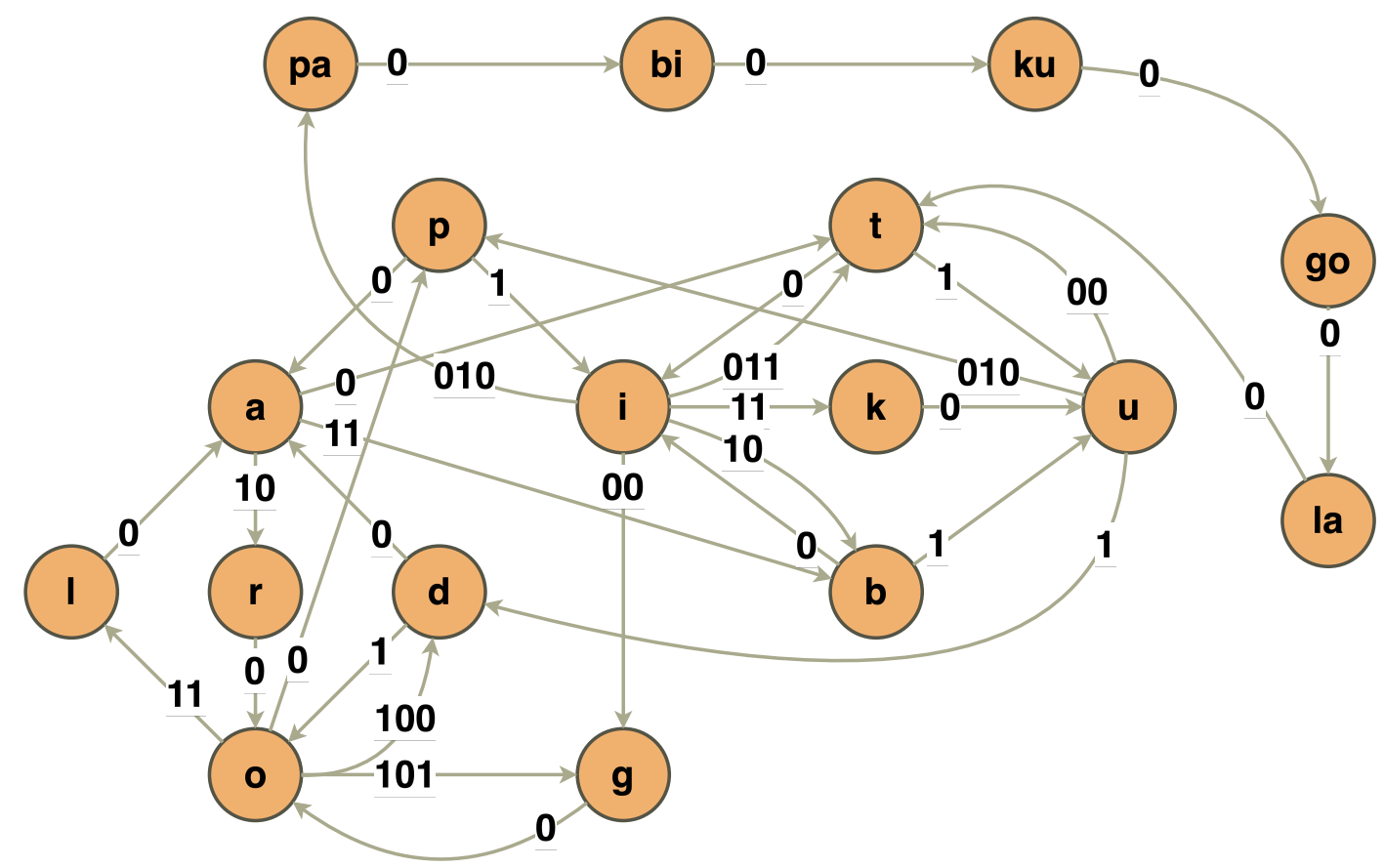

a → 101 0
i → 100 0
o → 011 0
u → 010 0
p → 1100 0
b → 1101 0
t → 1110 0
d → 1111 0
k → 0011 0
g → 0010 0
l → 0001 0
r → 0000 0

p a → 1100 10 0
b i → 1101 10 0
k u → 0011 1 0
go → 0010 1 0
l a → 0001 1 0

pa bi ku go la t u d a r o p i t i
b u d o p a b i k u d a r o p i g
o l a t u t i b u d o g o l a t u p
a b i k u t i b u d o d a r o p i

→

0 0 0 0 0 1 1 0 10 0 0 1 011 0 10
1 1 1 0 0 11 0 11 0 1 0 10 0 0
1 00 0 11 0 0 1 00 0 10 1 1
1 101 0 11 0 0 1 010 0 11 0 11
0 00 0 10 1 1 1 100 0 10 0 0
1 010

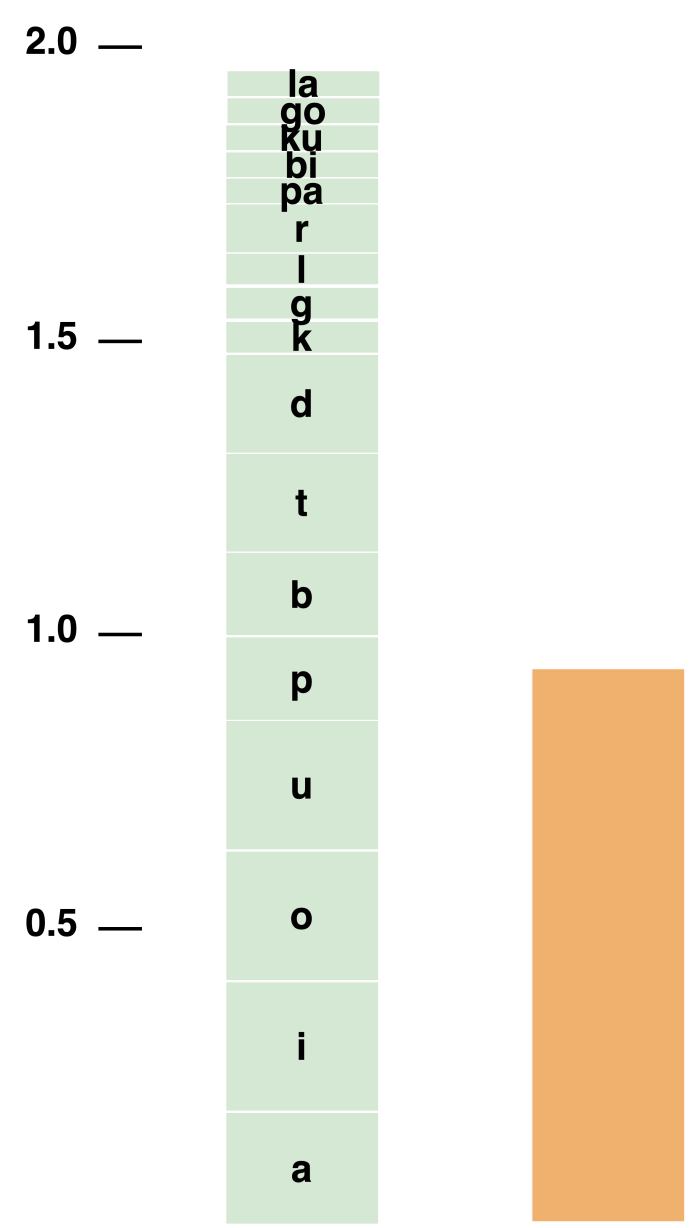

L(S) = 0.28 + 1.87 = 2.15 bits

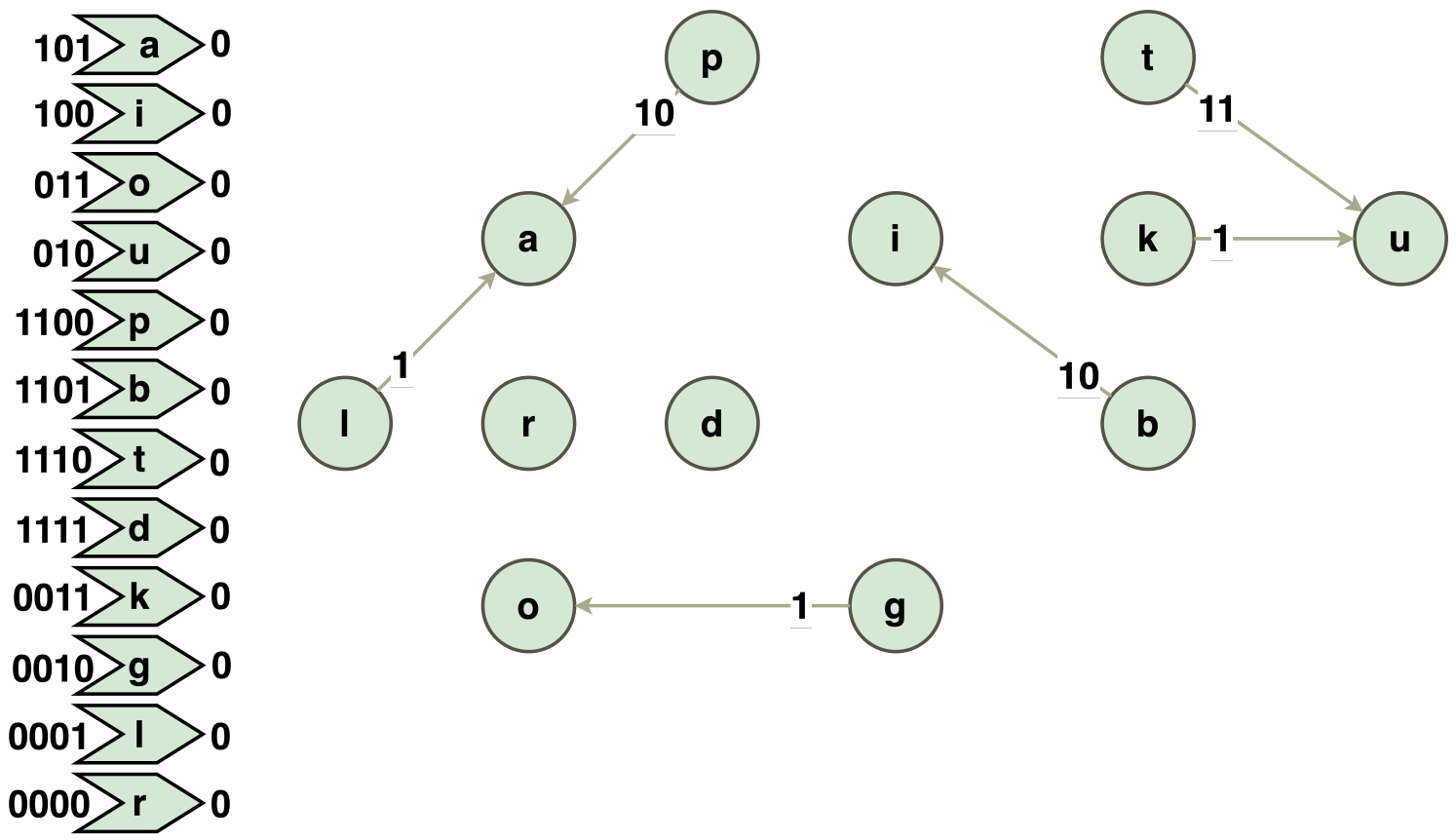

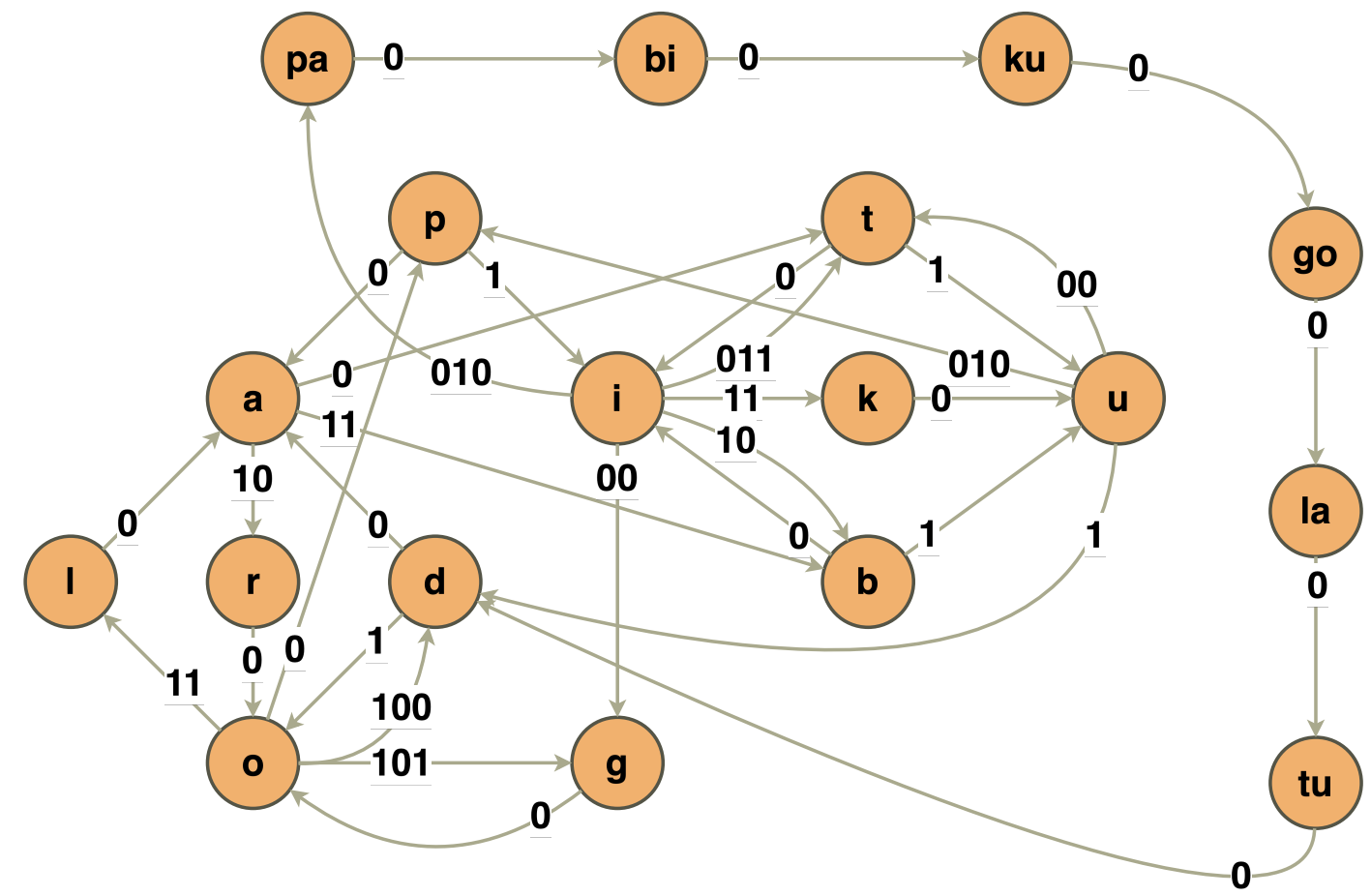

a → 101 0
i → 100 0
o → 011 0
u → 010 0
p → 1100 0
b → 1101 0
t → 1110 0
d → 1111 0
k → 0011 0
g → 0010 0
l → 0001 0
r → 0000 0

p a → 1100 10 0
b i → 1101 10 0
k u → 0011 1 0
go → 0010 1 0
l a → 0001 1 0
t u → 1110 11 0

pa bi ku go la tu d a r o p i t i
b u d o p a b i k u d a r o p i g
o l a t u t i b u d o g o l a t u p
a b i k u t i b u d o d a r o p i

→

0 0 0 0 0 0 0 10 0 0 1 011 0 10 1
1 1 0 0 11 0 11 0 1 0 10 0 0
1 00 0 11 0 0 1 00 0 10 1 1
1 101 0 11 0 0 1 010 0 11 0 11
0 00 0 10 1 1 1 100 0 10 0 0
1 010

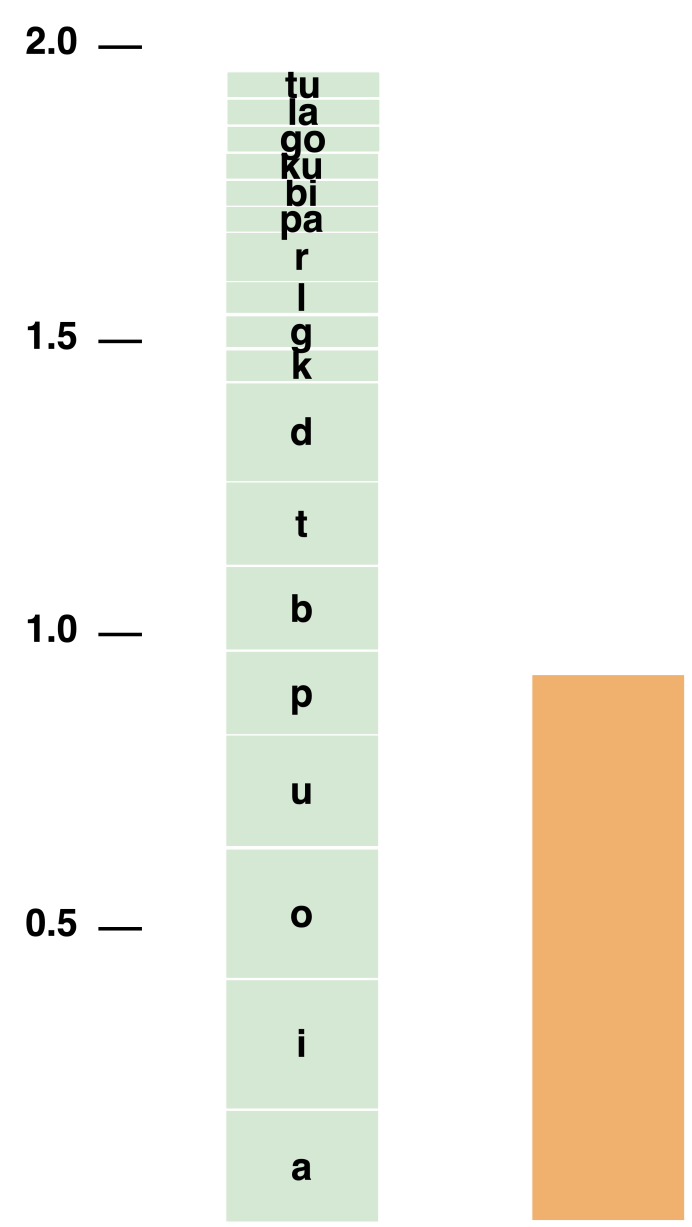

L(S) = 0.27 + 1.86 = 2.13 bits

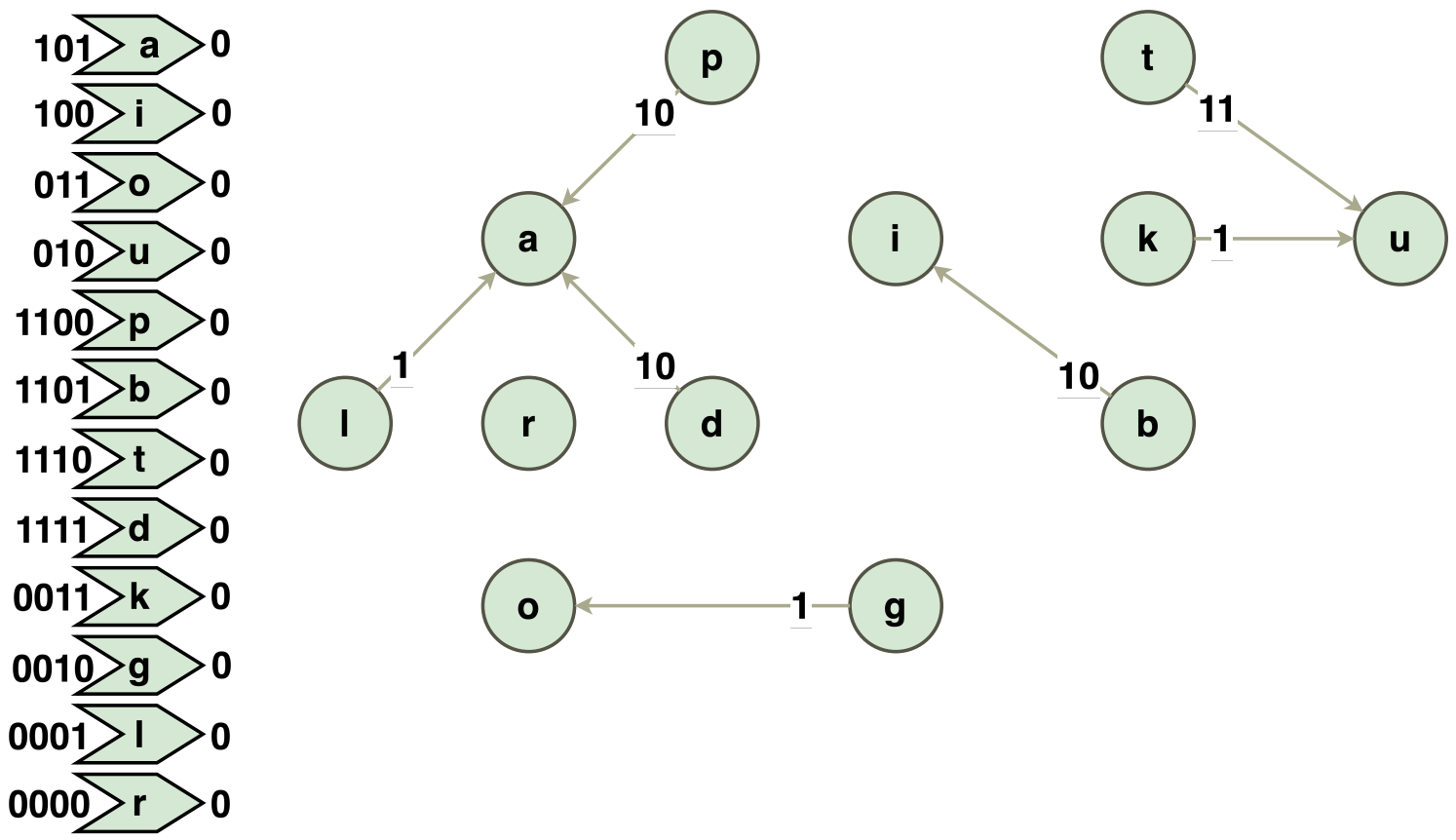

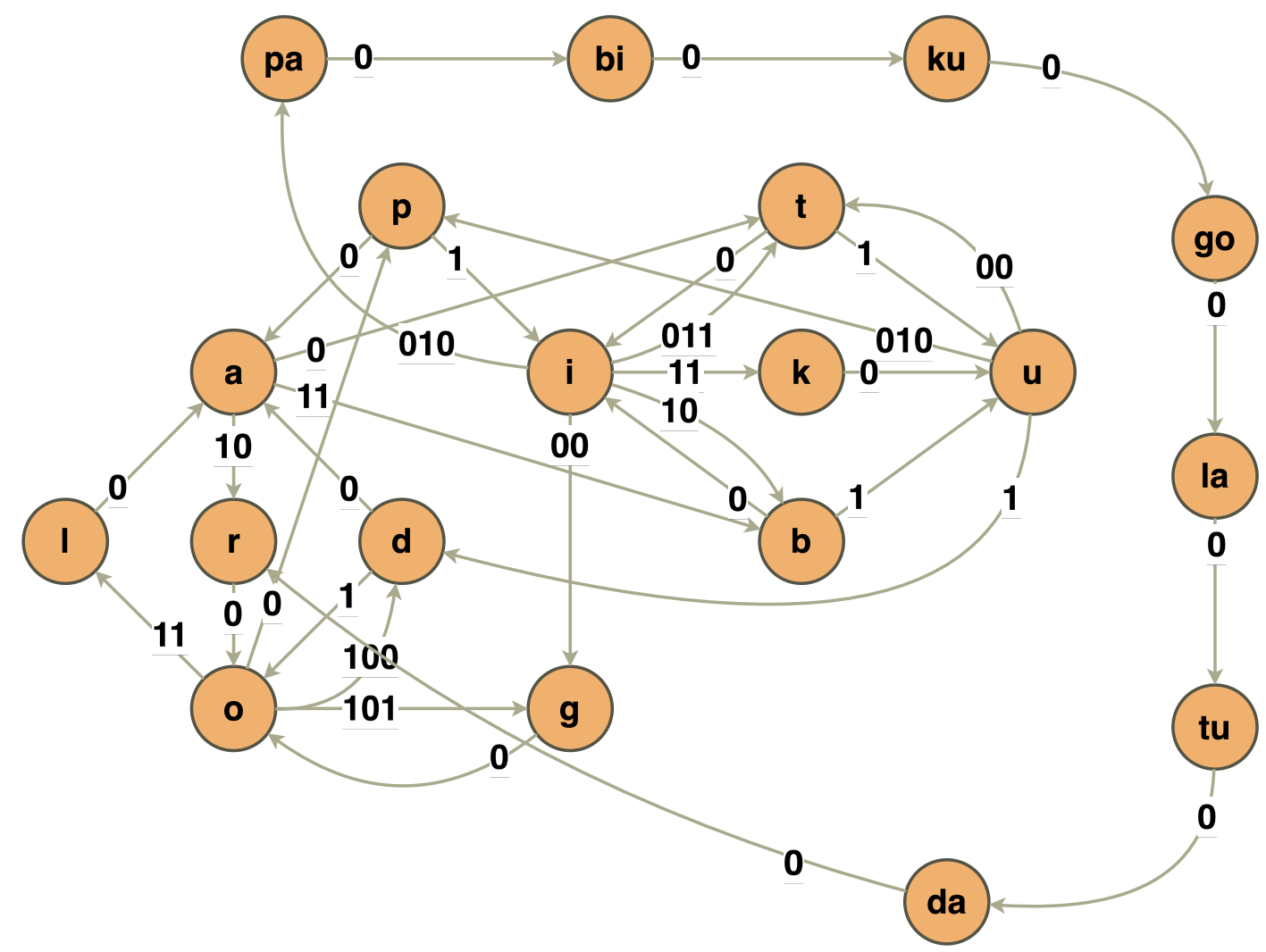

a	→	101 0
i	→	100 0
o	→	011 0
u	→	010 0
p	→	1100 0
b	→	1101 0
t	→	1110 0
d	→	1111 0
k	→	0011 0
g	→	0010 0
l	→	0001 0
r	→	0000 0

p a	→	1100 10 0
b i	→	1101 10 0
k u	→	0011 1 0
go	→	0010 1 0
l a	→	0001 1 0
t u	→	1110 11 0
d a	→	1111 10 0

pa bi ku go la tu da r o p i t i b
u d o p a b i k u d a r o p i g o l
a t u t i b u d o g o l a t u p a b
i k u t i b u d o d a r o p i

→

0 0 0 0 0 0 0 0 0 1 011 0 10 1 1
1 0 0 11 0 11 0 1 0 10 0 0 1 00 0
11 0 0 1 00 0 10 1 1 1 101 0 11 0
0 1 010 0 11 0 11 0 00 0 10 1 1
1 100 0 10 0 0 1 010

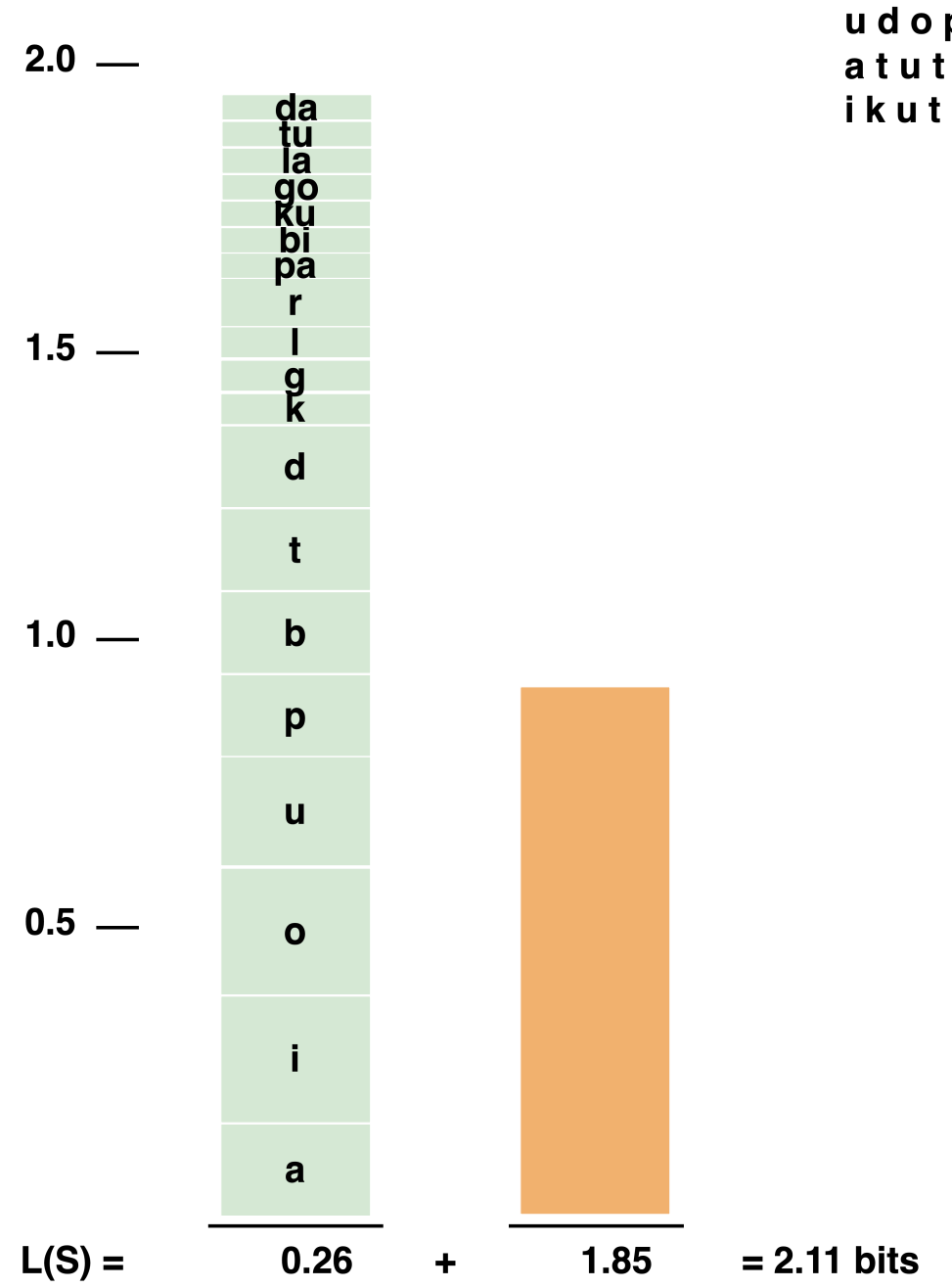

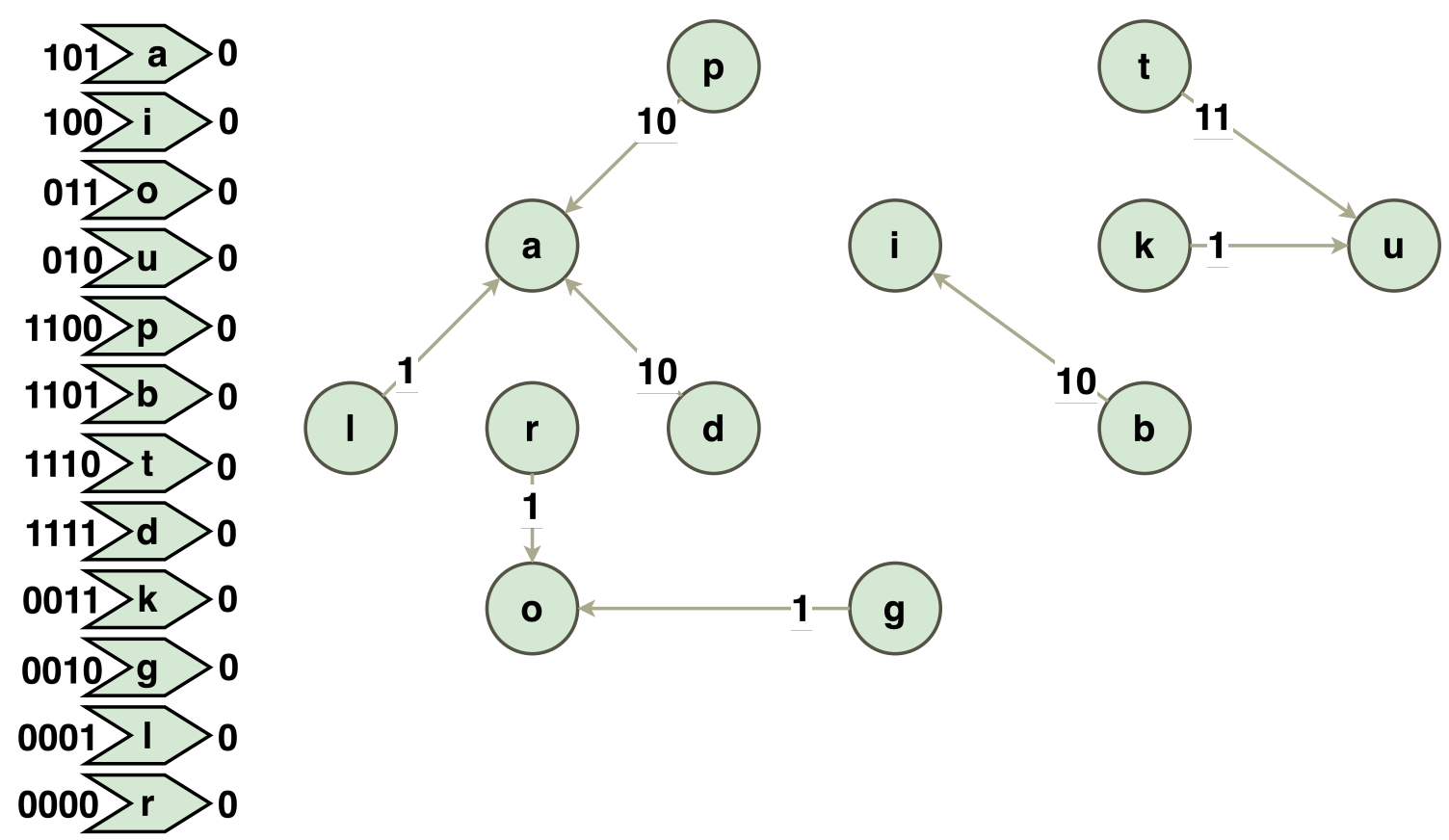

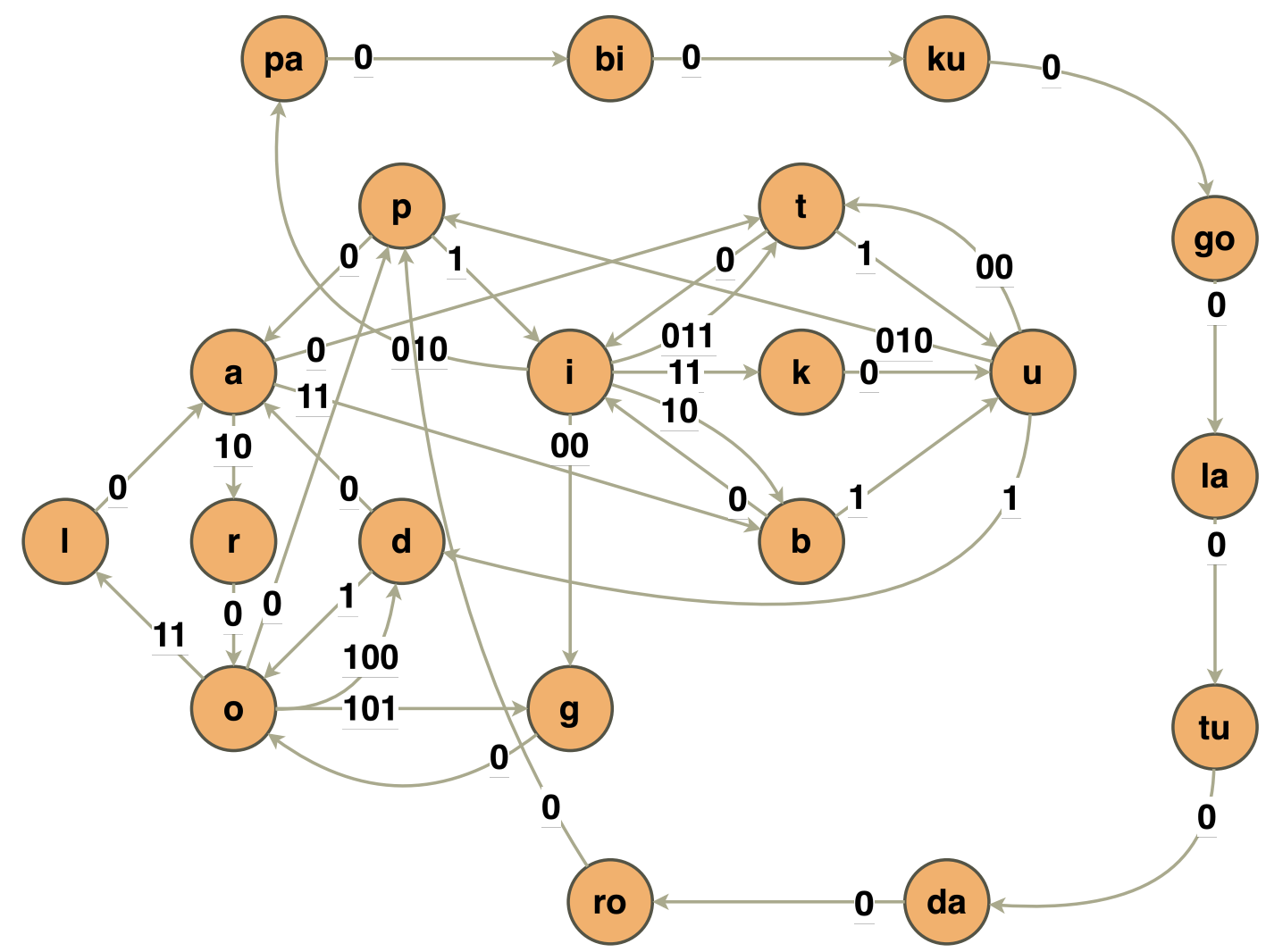

a → 101 0
i → 100 0
o → 011 0
u → 010 0
p → 1100 0
b → 1101 0
t → 1110 0
d → 1111 0
k → 0011 0
g → 0010 0
l → 0001 0
r → 0000 0

p a → 1100 10 0
b i → 1101 10 0
k u → 0011 1 0
go → 0010 1 0
l a → 0001 1 0
t u → 1110 11 0
d a → 1111 10 0
r o → 0000 1 0

pa bi ku go la tu da ro p i t i b
u d o p a b i k u d a r o p i g o l
a t u t i b u d o g o l a t u p a b
i k u t i b u d o d a r o p i

→

0 0 0 0 0 0 0 0 1 011 0 10 1 1
1 0 0 11 0 11 0 1 0 10 0 0 1 00 0
11 0 0 1 00 0 10 1 1 1 101 0 11 0
0 1 010 0 11 0 11 0 00 0 10 1 1
1 100 0 10 0 0 1 010

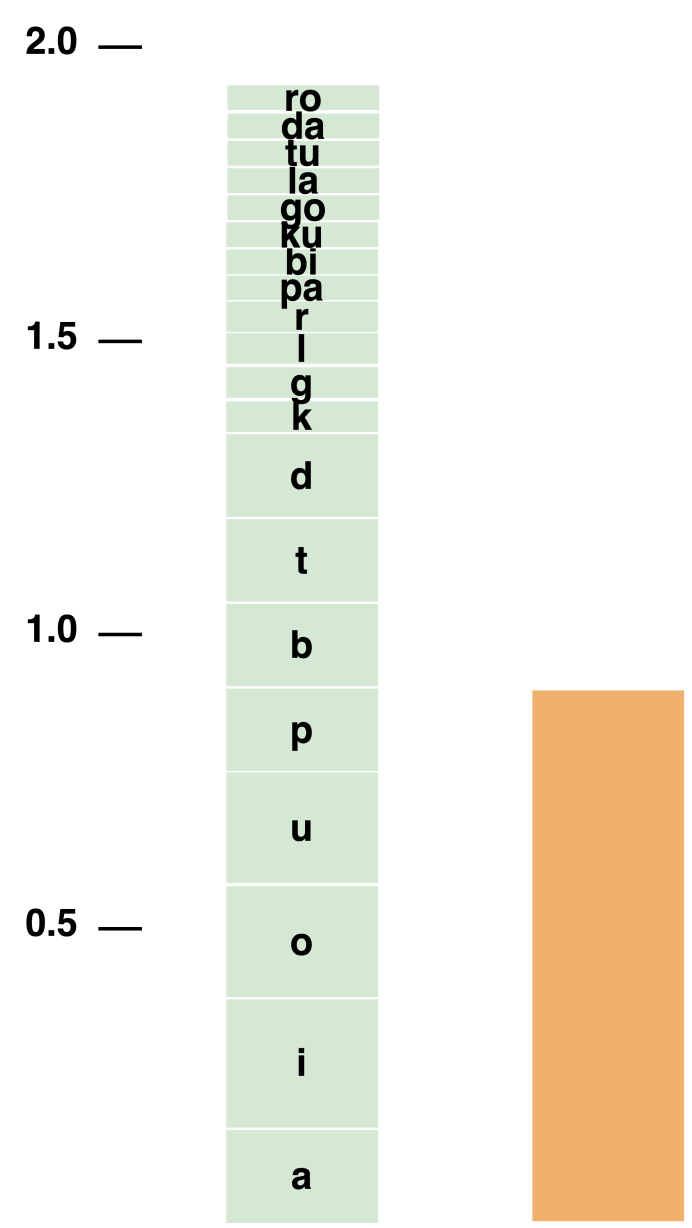

L(S) = 0.25 + 1.85 = 2.10 bits

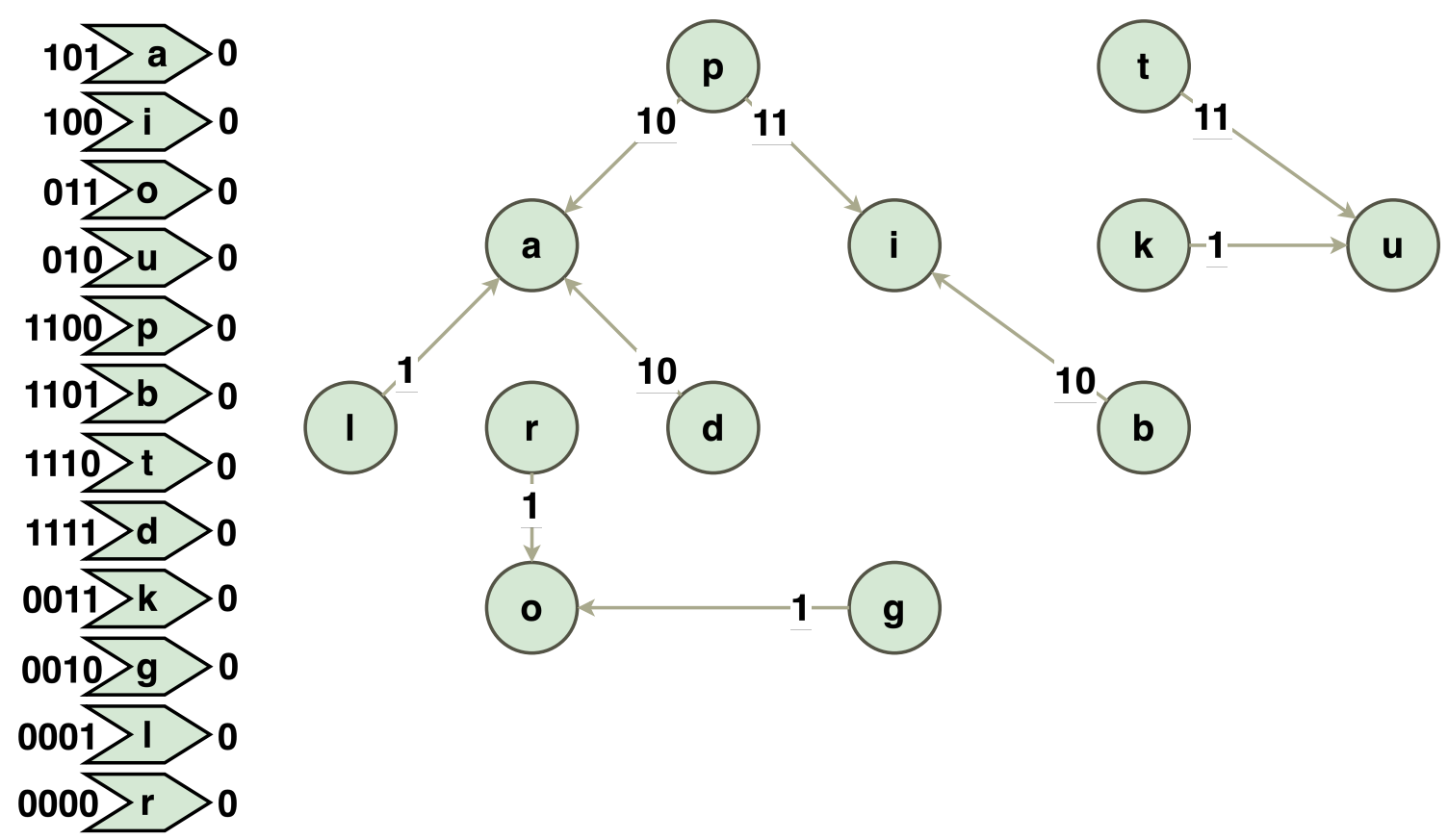

a	101 0	p a	1100 10 0
i	100 0	b i	1101 10 0
o	011 0	k u	0011 1 0
u	010 0	go	0010 1 0
p	1100 0	l a	0001 1 0
b	1101 0	t u	1110 11 0
t	1110 0	d a	1111 10 0
d	1111 0	r o	0000 1 0
k	0011 0	p i	1100 11 0
g	0010 0		
l	0001 0		
r	0000 0		

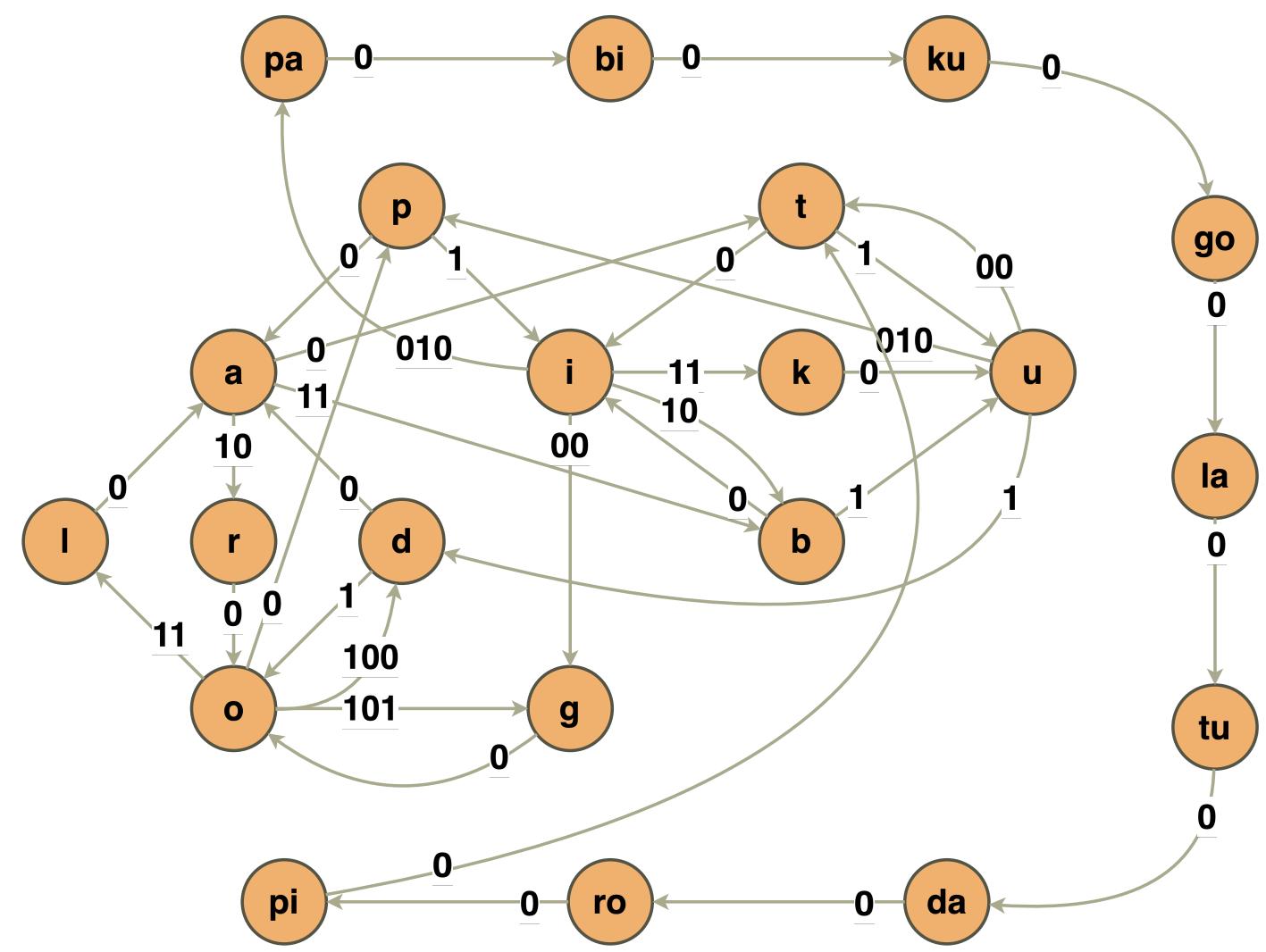

pa bi ku go la tu da ro pi t i b
u d o p a b i k u d a r o p i g o l
a t u t i b u d o g o l a t u p a b
i k u t i b u d o d a r o p i

→

0 0 0 0 0 0 0 0 0 10 1 1 1 0 0
11 0 11 0 1 0 10 0 0 1 00 0 11 0
0 1 00 0 10 1 1 1 101 0 11 0 0
1 010 0 11 0 11 0 00 0 10 1 1
1 100 0 10 0 0 1 010

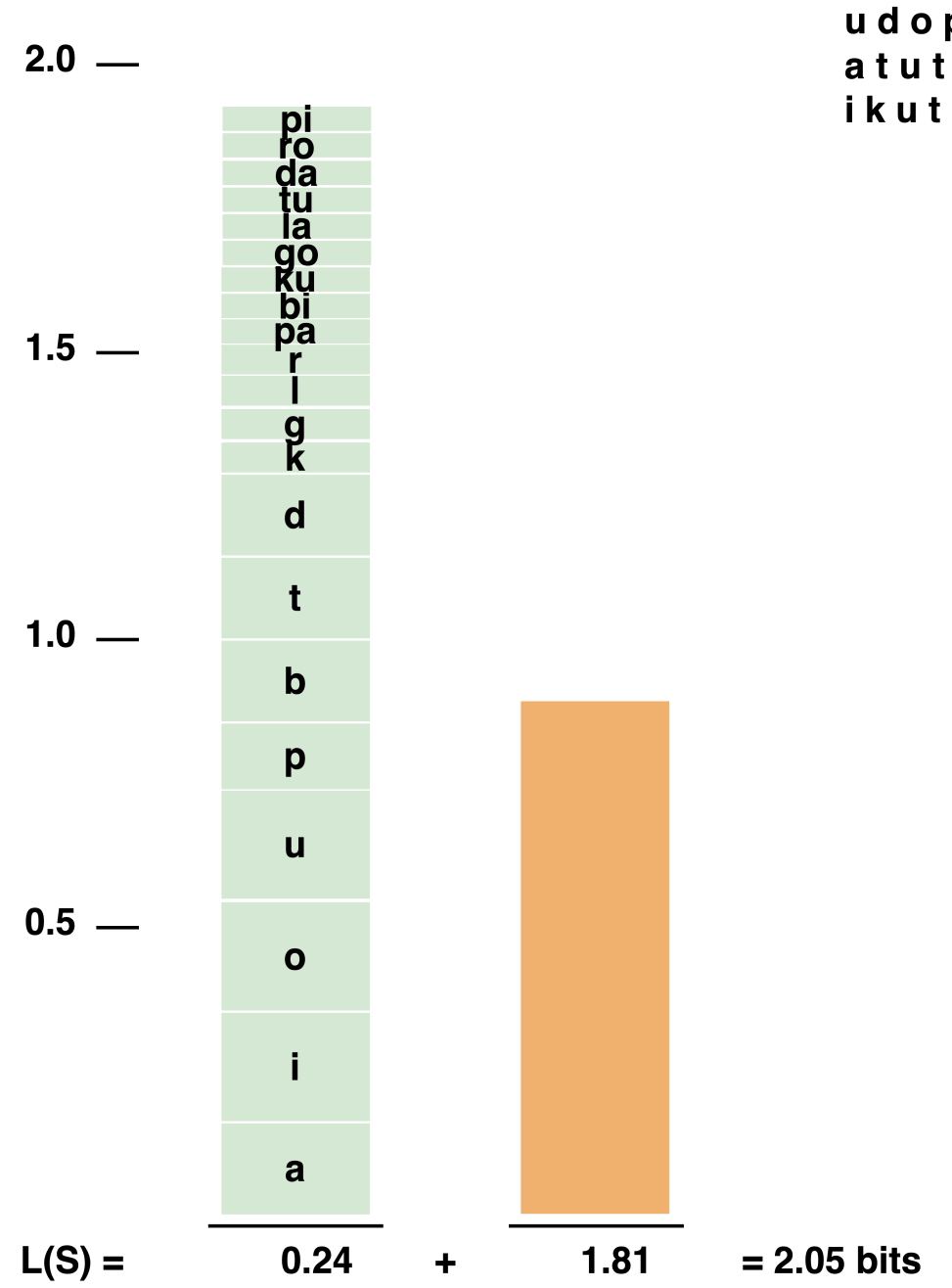

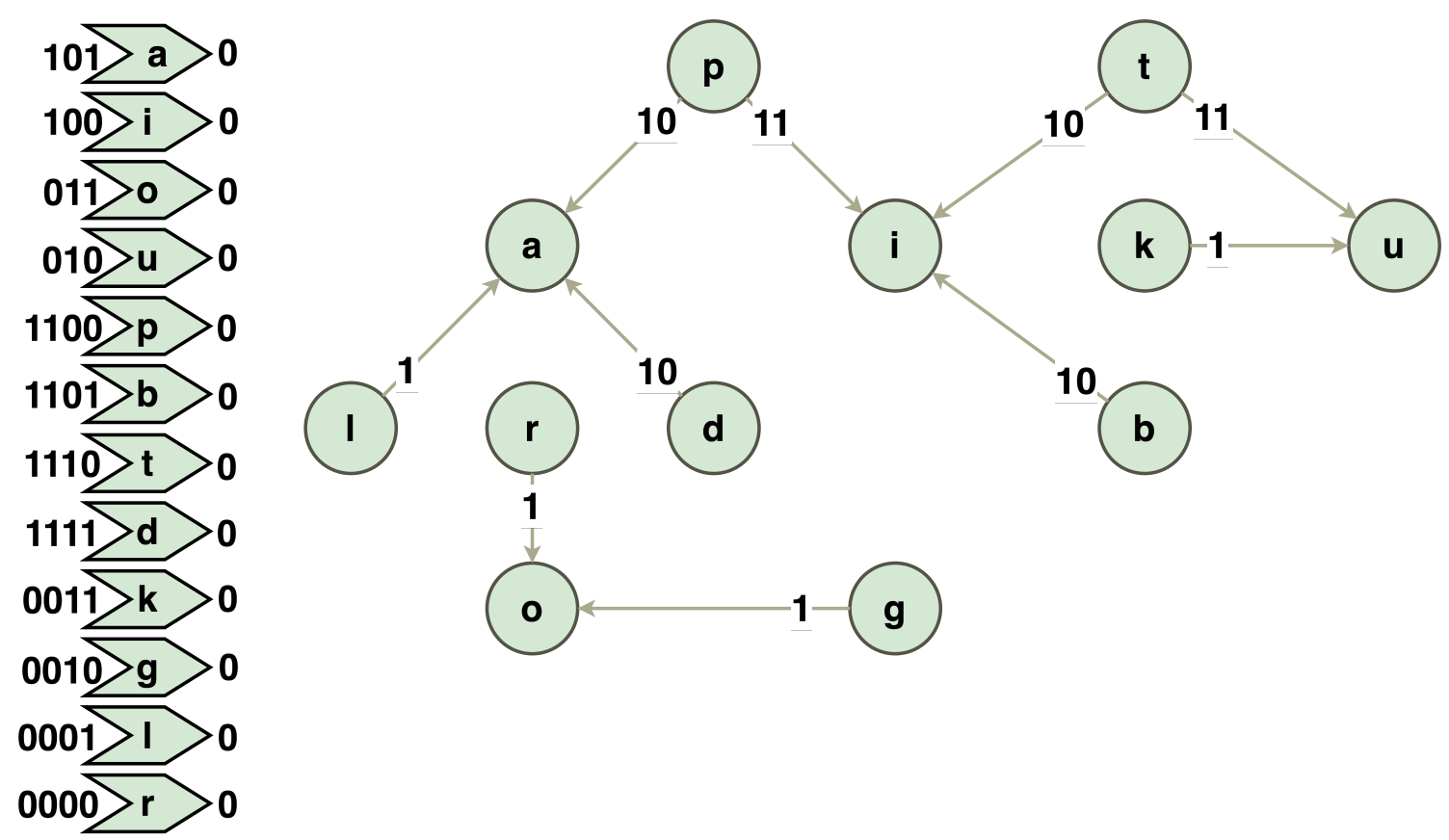

a	101 0	p a	1100 10 0
i	100 0	b i	1101 10 0
o	011 0	k u	0011 1 0
u	010 0	go	0010 1 0
p	1100 0	l a	0001 1 0
b	1101 0	t u	1110 11 0
t	1110 0	d a	1111 10 0
d	1111 0	r o	0000 1 0
k	0011 0	p i	1100 11 0
g	0010 0	t i	1110 10 0
l	0001 0		
r	0000 0		

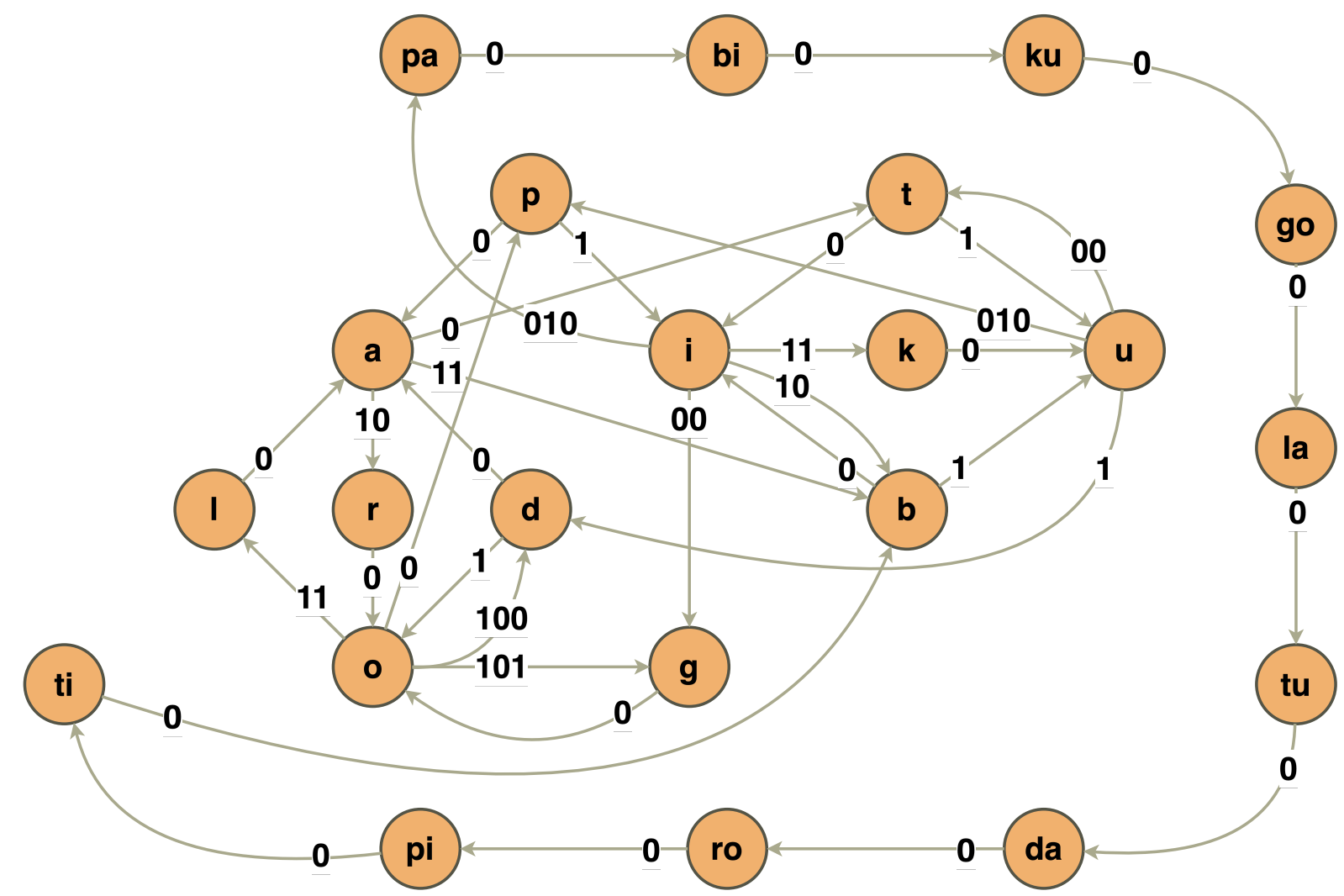

pa bi ku go la tu da ro pi ti b u
d o p a b i k u d a r o p i g o l a
t u t i b u d o g o l a t u p a b i
k u t i b u d o d a r o p i

→

0 0 0 0 0 0 0 0 0 0 1 1 1 0 0 11 0
11 0 1 0 10 0 0 1 00 0 11 0 0
1 00 0 10 1 1 1 101 0 11 0 0
1 010 0 11 0 11 0 00 0 10 1 1
1 100 0 10 0 0 1 010

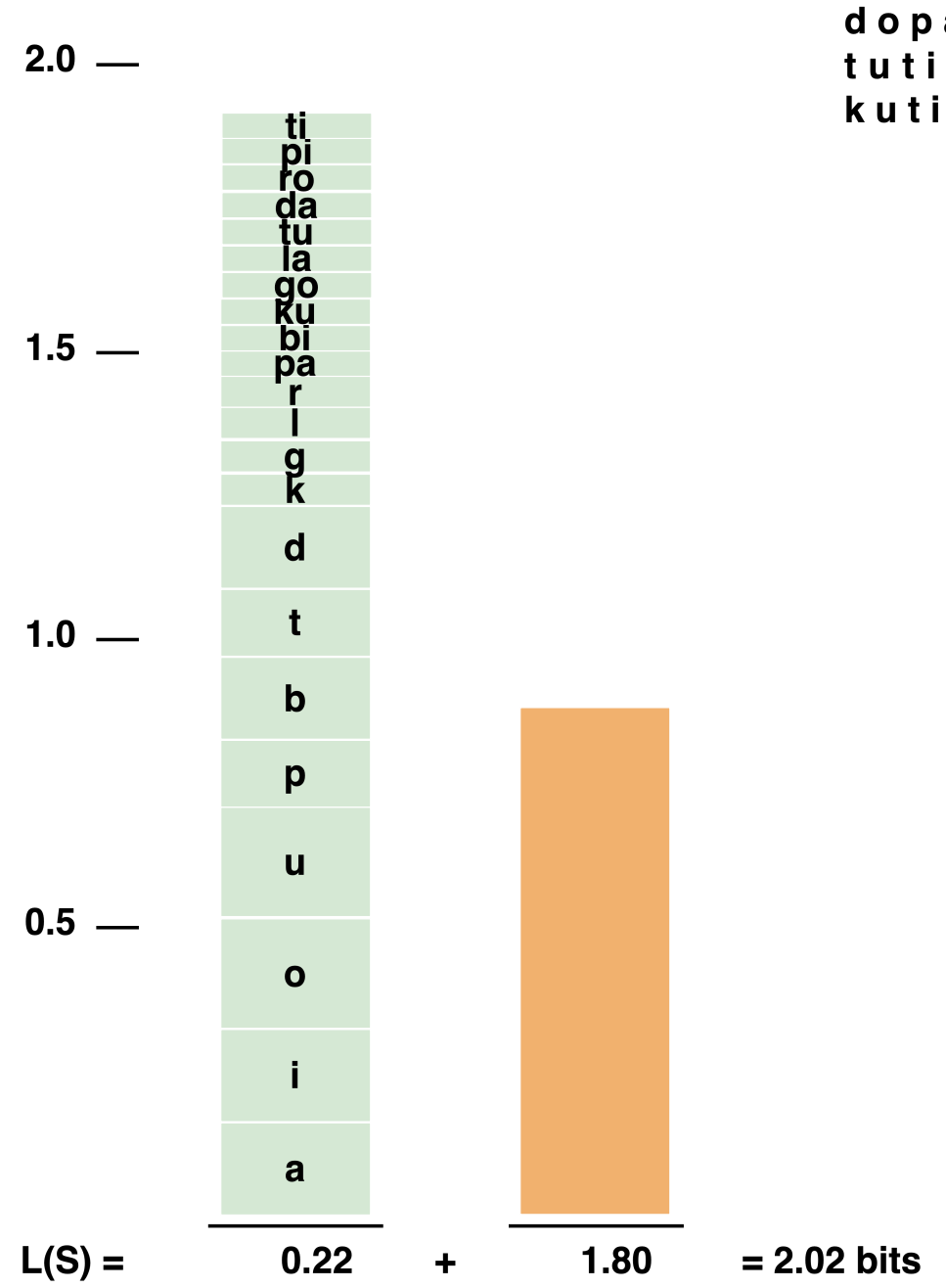

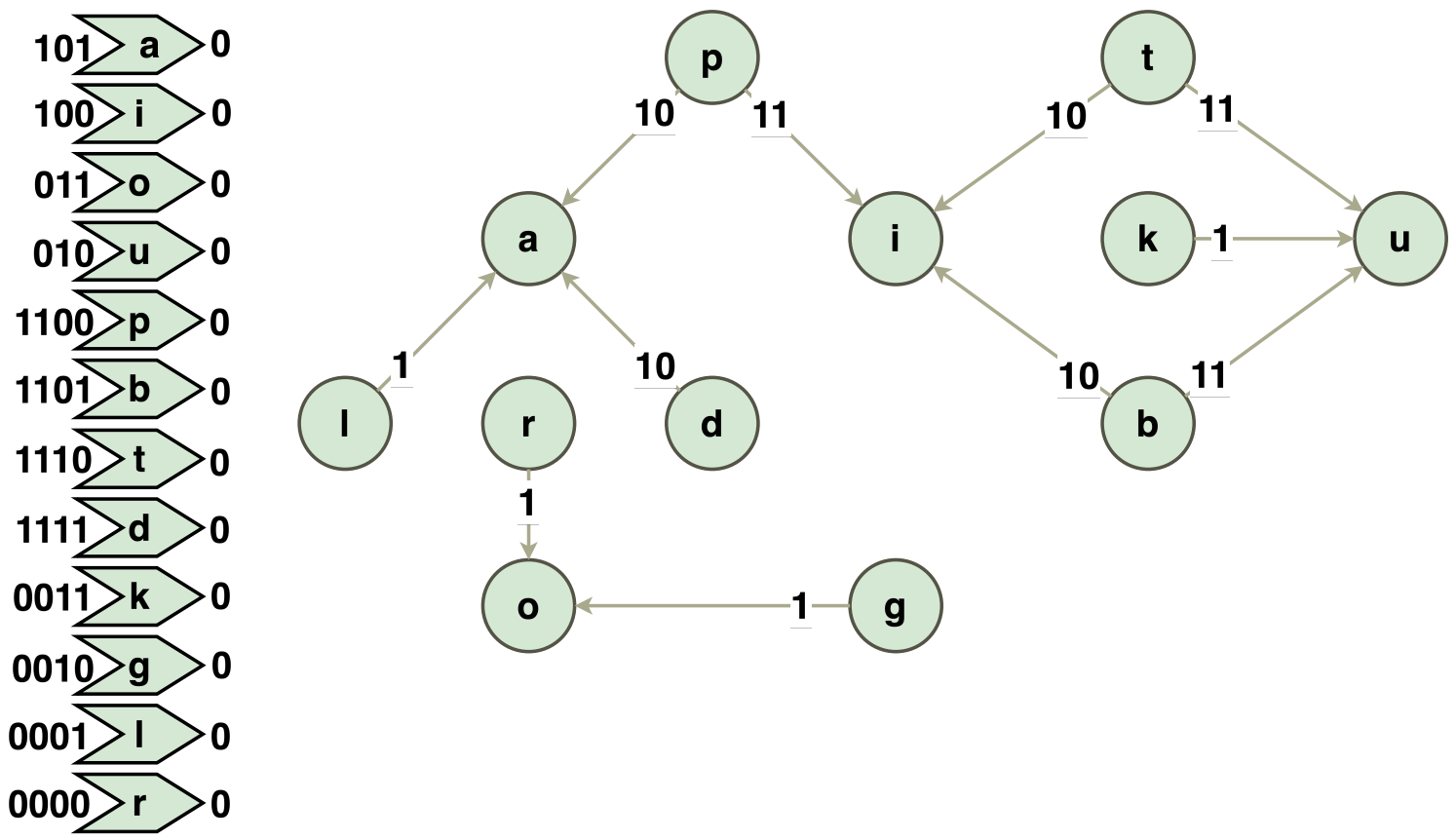

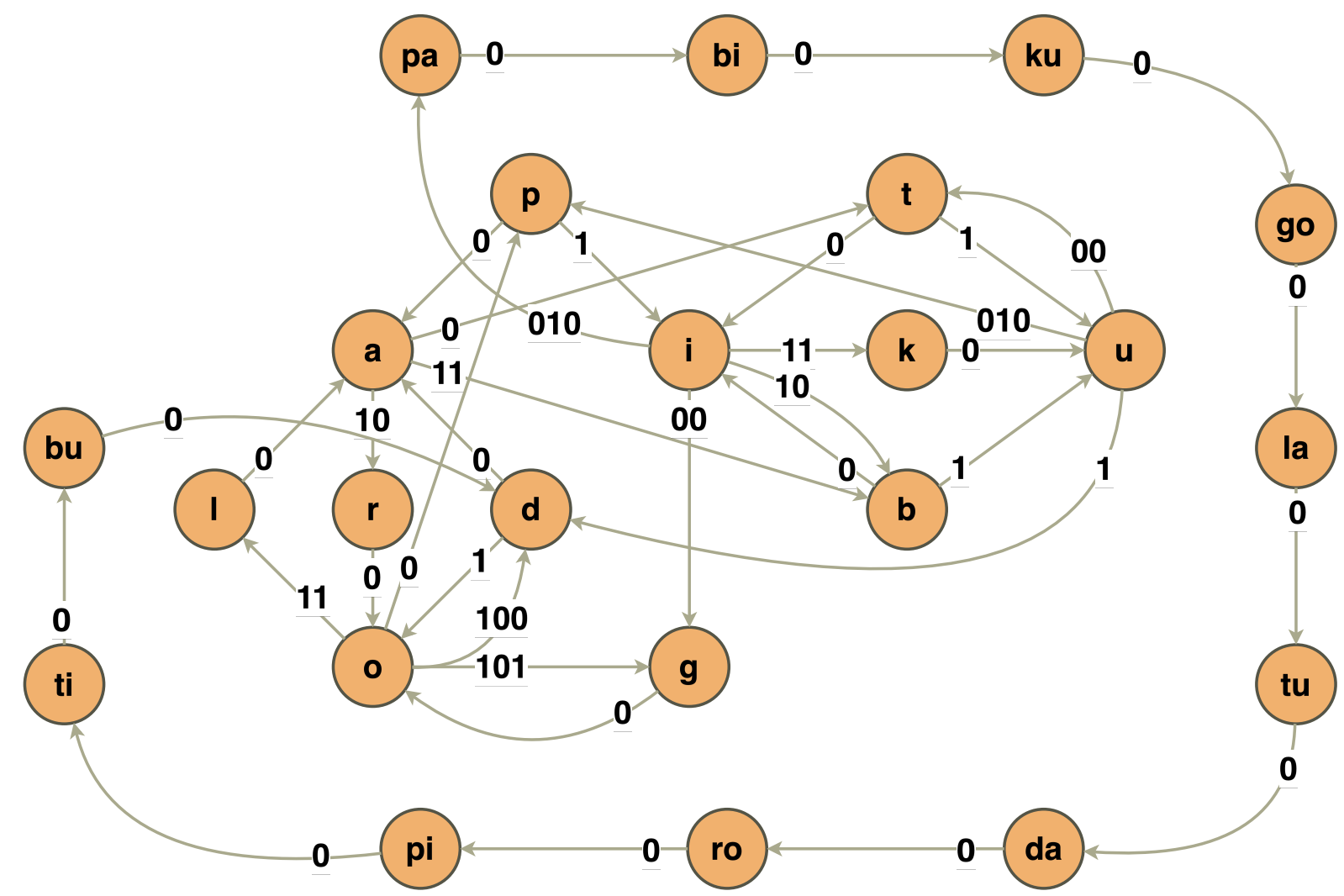

a	→	101 0
i	→	100 0
o	→	011 0
u	→	010 0
p	→	1100 0
b	→	1101 0
t	→	1110 0
d	→	1111 0
k	→	0011 0
g	→	0010 0
l	→	0001 0
r	→	0000 0

p a	→	1100 10 0
b i	→	1101 10 0
k u	→	0011 1 0
go	→	0010 1 0
l a	→	0001 1 0
t u	→	1110 11 0
d a	→	1111 10 0
r o	→	0000 1 0
p i	→	1100 11 0
t i	→	1110 10 0
b u	→	1101 11 0

pa bi ku go la tu da ro pi ti bu
d o p a b i k u d a r o p i g o l a
t u t i b u d o g o l a t u p a b i
k u t i b u d o d a r o p i

→

0 0 0 0 0 0 0 0 0 0 0 1 0 0 11 0
11 0 1 0 10 0 0 1 00 0 11 0 0
1 00 0 10 1 1 1 101 0 11 0 0
1 010 0 11 0 11 0 00 0 10 1 1
1 100 0 10 0 0 1 010

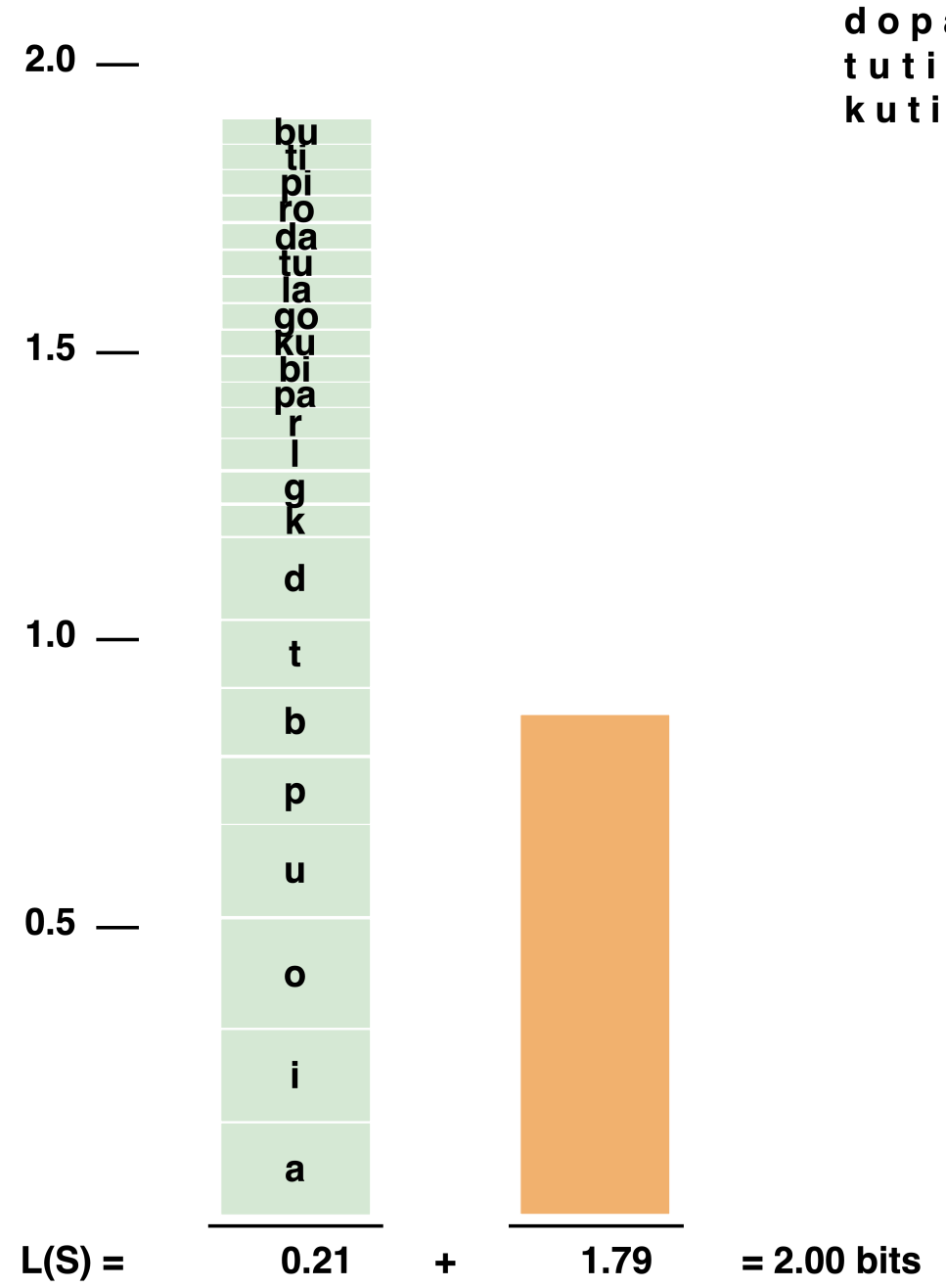

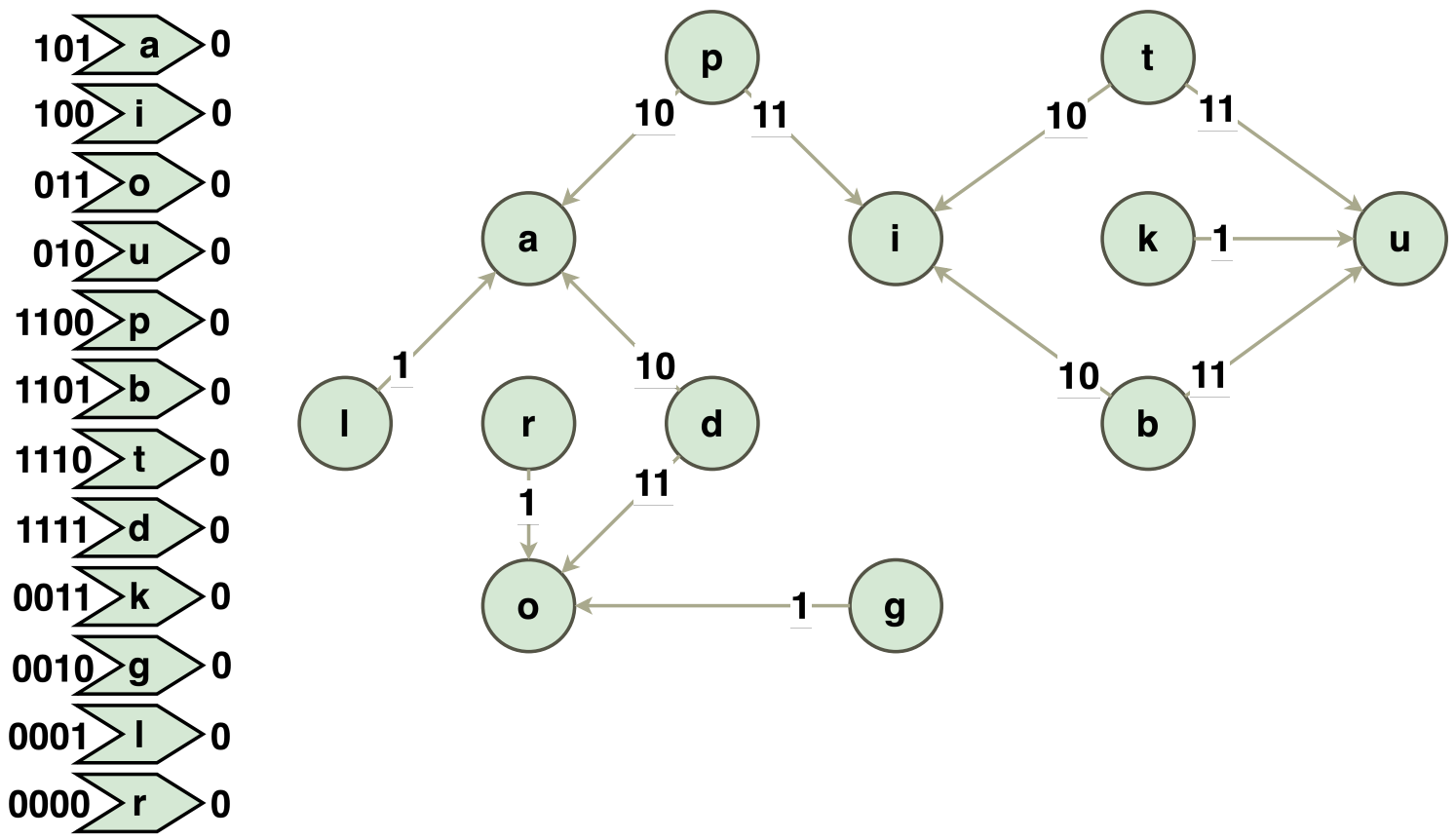

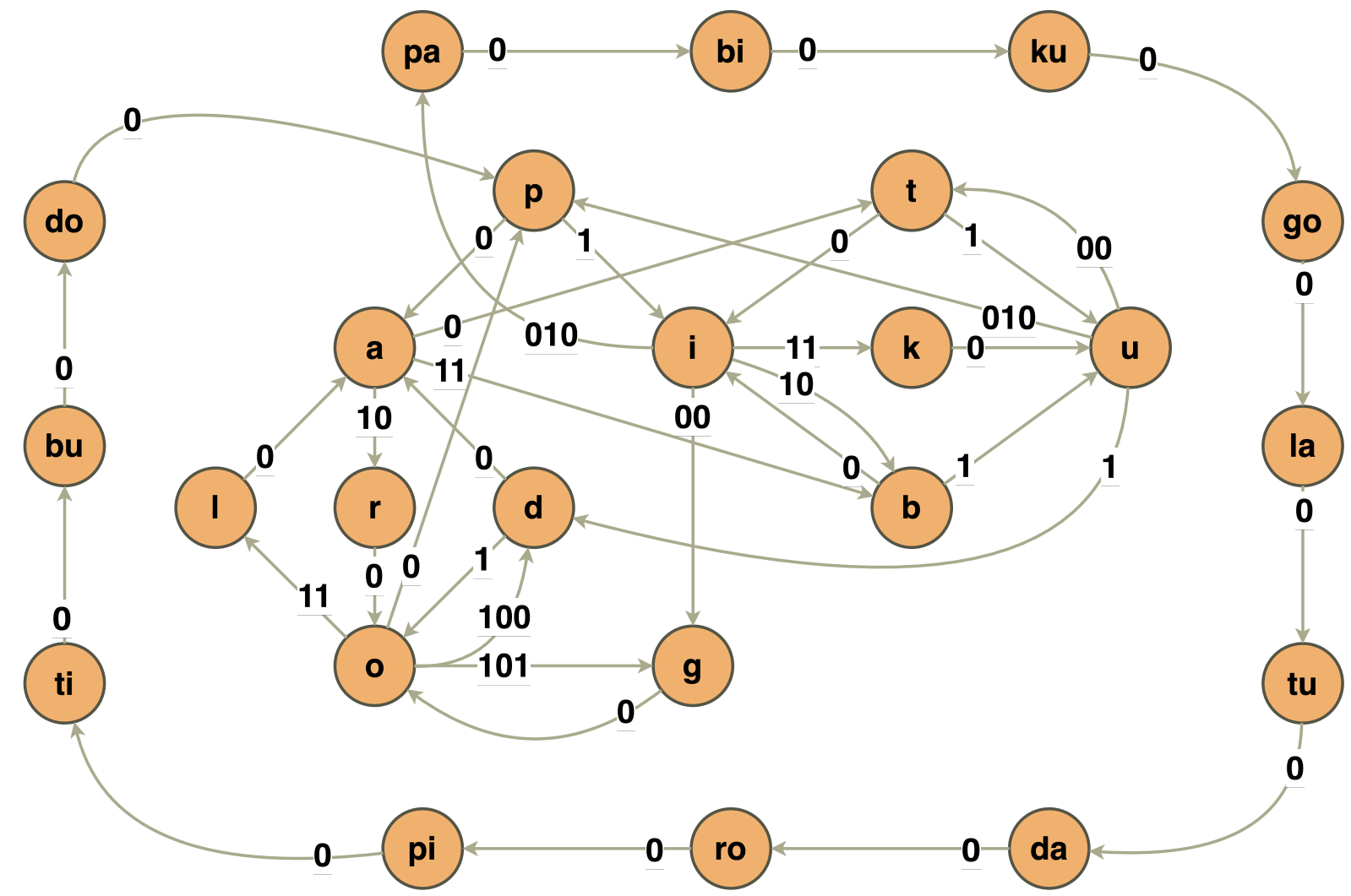

a	→	101 0	p a	→	1100 10 0
i	→	100 0	b i	→	1101 10 0
o	→	011 0	k u	→	0011 1 0
u	→	010 0	go	→	0010 1 0
p	→	1100 0	l a	→	0001 1 0
b	→	1101 0	t u	→	1110 11 0
t	→	1110 0	d a	→	1111 10 0
d	→	1111 0	r o	→	0000 1 0
k	→	0011 0	p i	→	1100 11 0
g	→	0010 0	t i	→	1110 10 0
l	→	0001 0	b u	→	1101 11 0
r	→	0000 0	d o	→	1111 11 0

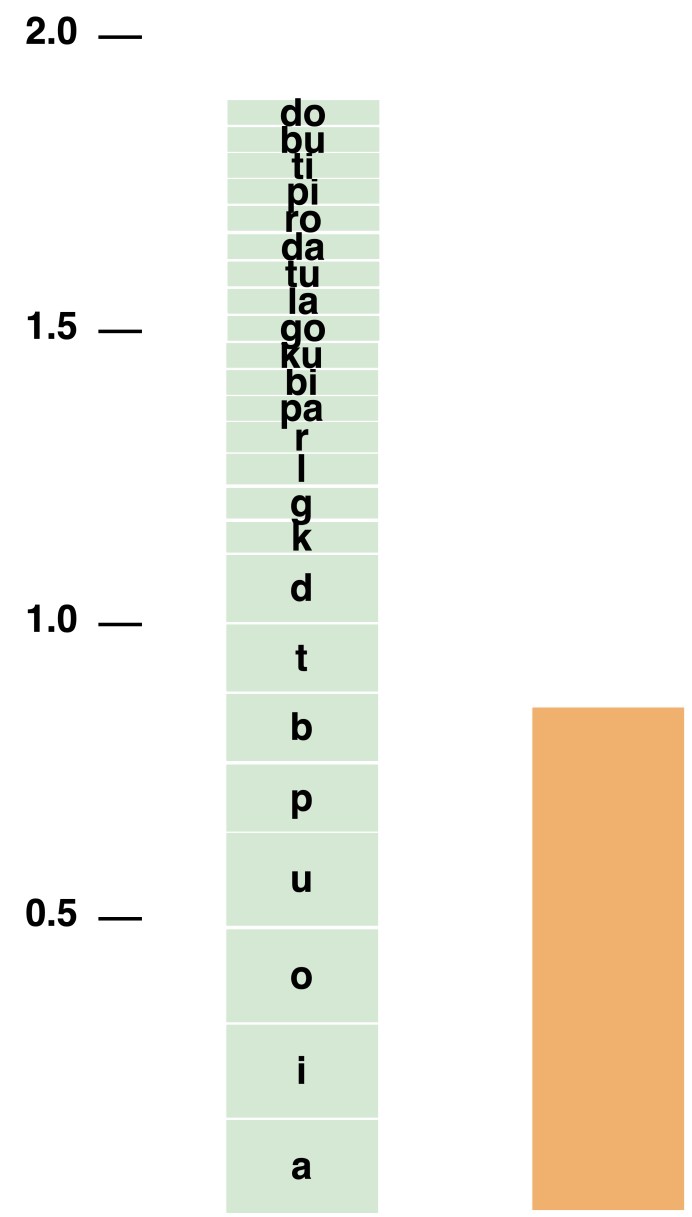

L(S) = 0.20 + 1.78 = 1.98 bits

pa bi ku go la tu da ro pi ti bu
do p a b i k u d a r o p i g o l a
t u t i b u d o g o l a t u p a b i
k u t i b u d o d a r o p i

→

0 0 0 0 0 0 0 0 0 0 0 0 0 0 11 0 11
0 1 0 10 0 0 1 00 0 11 0 0 1 00 0
10 1 1 1 101 0 11 0 0 1 010 0 11
0 11 0 00 0 10 1 1 1 100 0 10 0 0
1 010

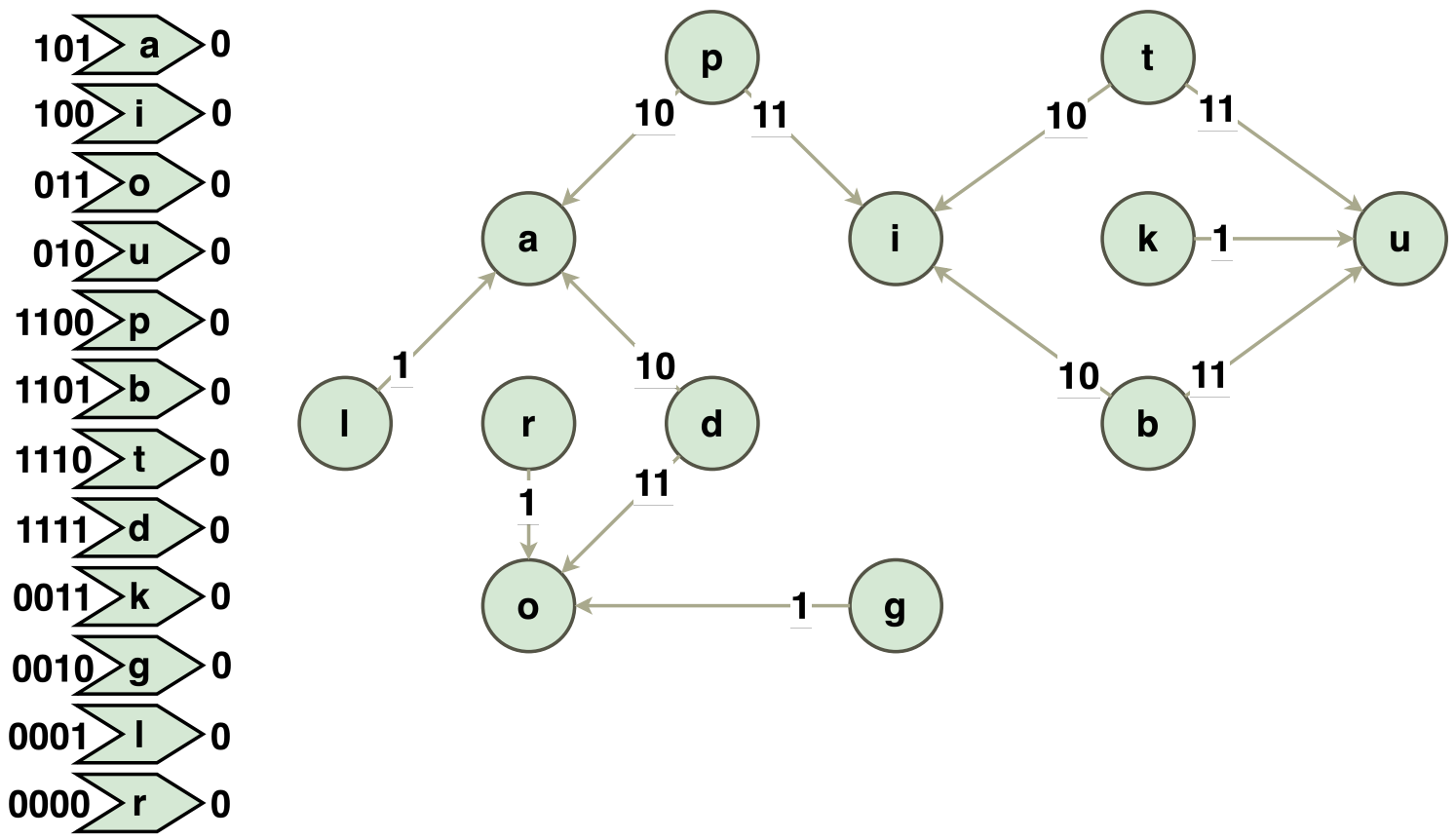

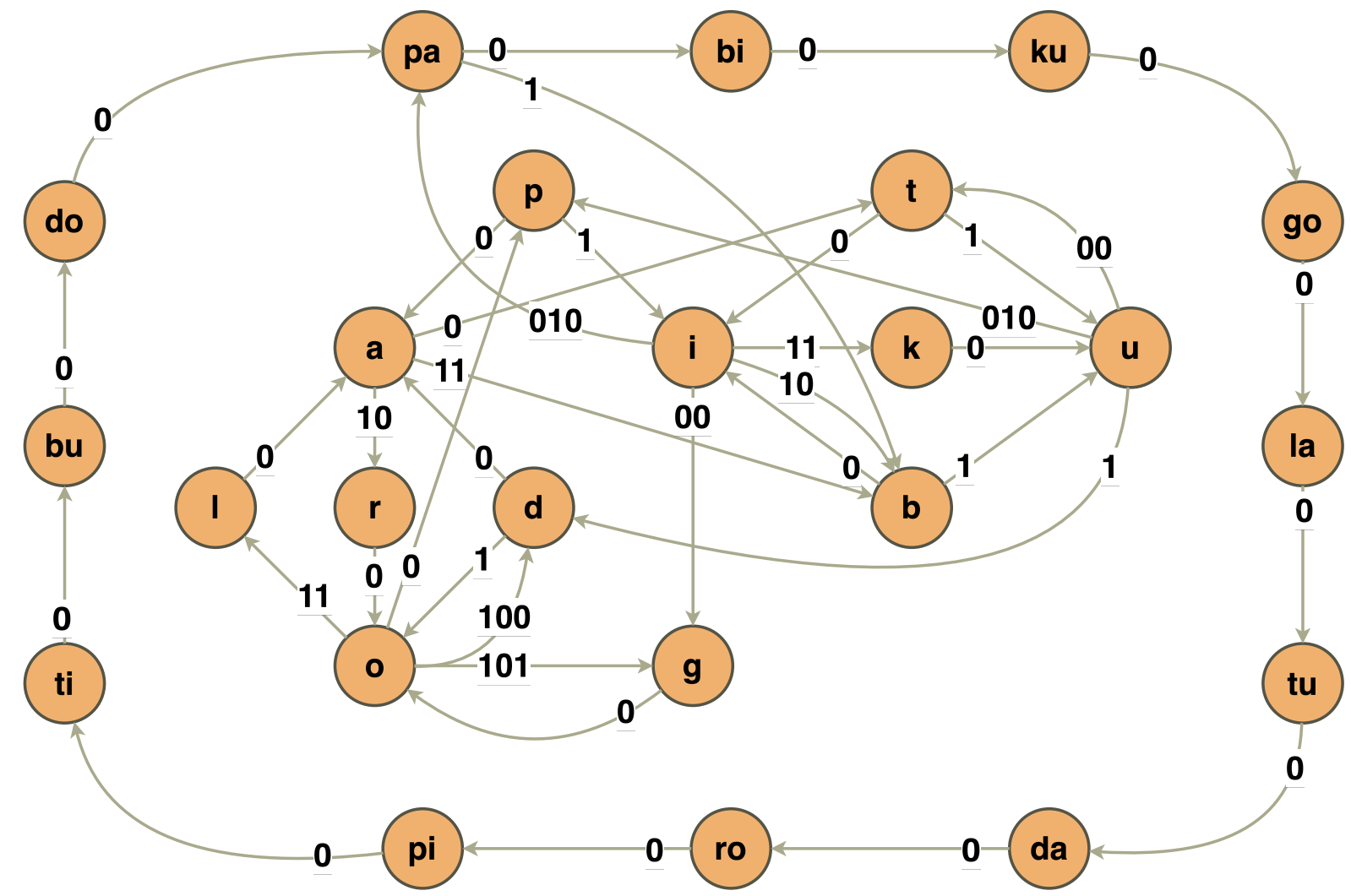

a → 101 0
i → 100 0
o → 011 0
u → 010 0
p → 1100 0
b → 1101 0
t → 1110 0
d → 1111 0
k → 0011 0
g → 0010 0
l → 0001 0
r → 0000 0

p a → 1100 10 0
b i → 1101 10 0
k u → 0011 1 0
go → 0010 1 0
l a → 0001 1 0
t u → 1110 11 0
d a → 1111 10 0
r o → 0000 1 0
p i → 1100 11 0
t i → 1110 10 0
b u → 1101 11 0
d o → 1111 11 0

pa bi ku go la tu da ro pi ti bu
do pa b i k u d a r o p i g o l a t
u t i b u d o g o l a t u p a b i k
u t i b u d o d a r o p i

→

0 0 0 0 0 0 0 0 0 0 0 0 1 0 11
0 1 0 10 0 0 1 00 0 11 0 0 1 00 0
10 1 1 1 101 0 11 0 0 1 010 0 11
0 11 0 00 0 10 1 1 1 100 0 10 0 0
1 010

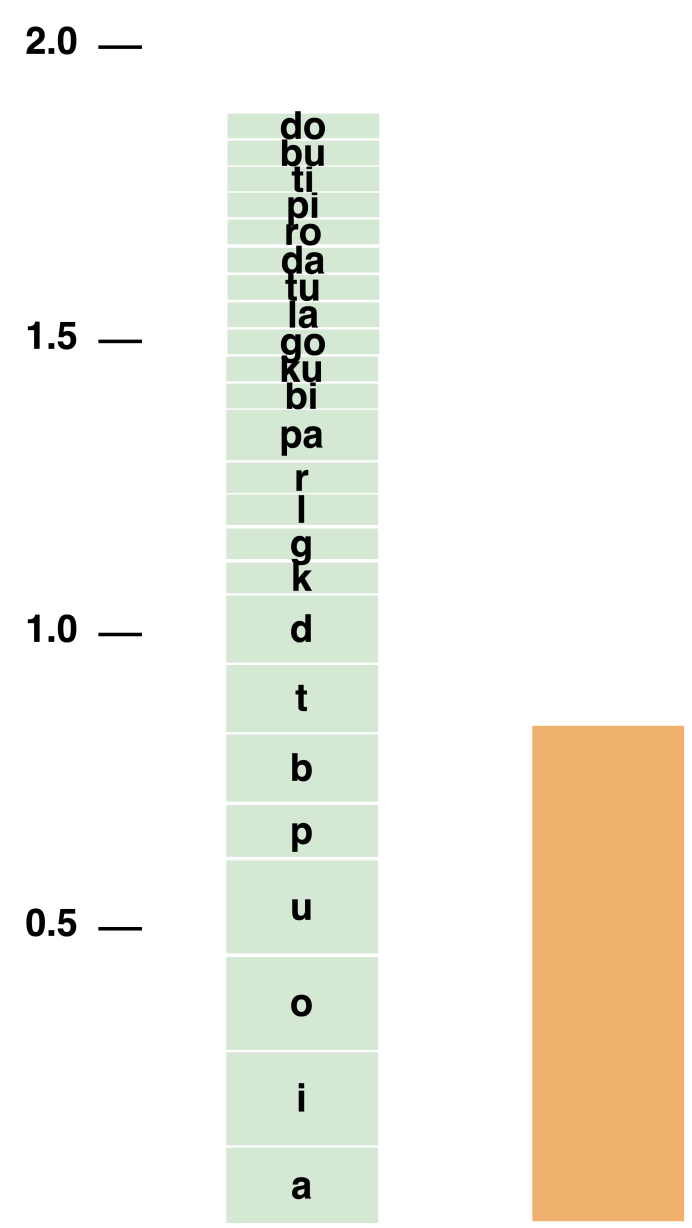

L(S) = 0.19 + 1.77 = 1.96 bits

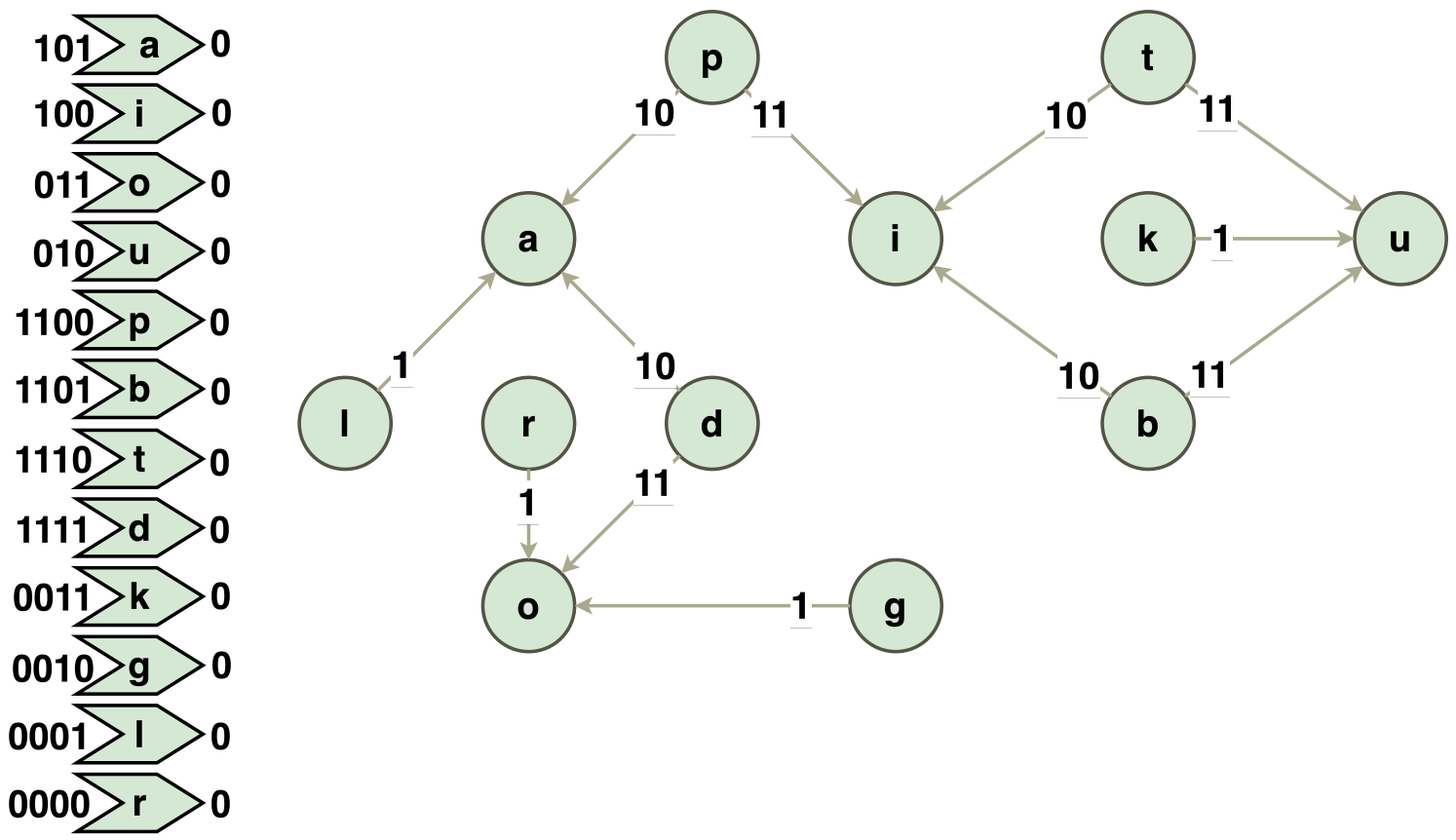

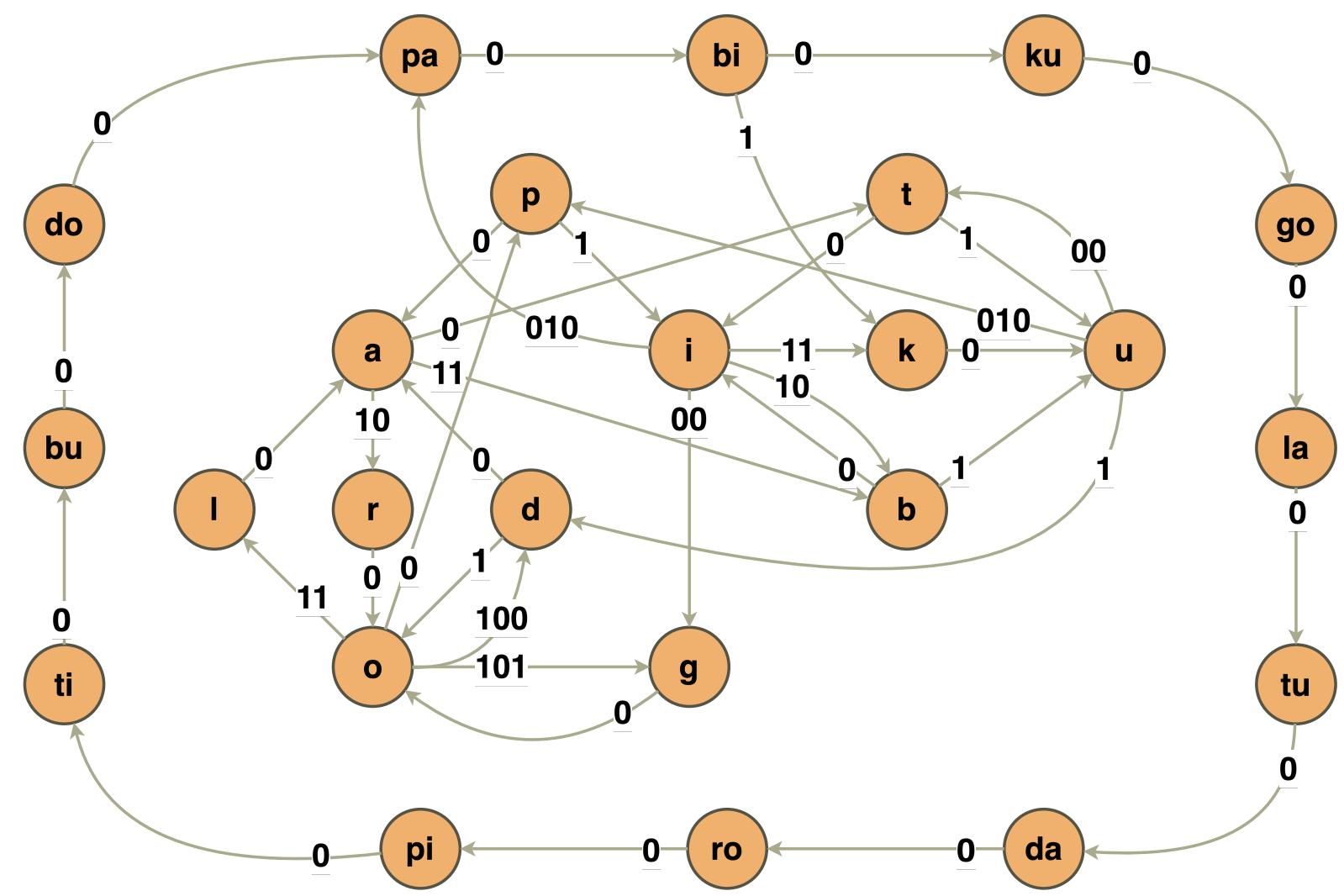

a	→	101 0
i	→	100 0
o	→	011 0
u	→	010 0
p	→	1100 0
b	→	1101 0
t	→	1110 0
d	→	1111 0
k	→	0011 0
g	→	0010 0
l	→	0001 0
r	→	0000 0

p a	→	1100 10 0
b i	→	1101 10 0
k u	→	0011 1 0
go	→	0010 1 0
l a	→	0001 1 0
t u	→	1110 11 0
d a	→	1111 10 0
r o	→	0000 1 0
p i	→	1100 11 0
t i	→	1110 10 0
b u	→	1101 11 0
d o	→	1111 11 0

pa bi ku go la tu da ro pi ti bu
do pa bi k u d a r o p i g o l a t
u t i b u d o g o l a t u p a b i k
u t i b u d o d a r o p i

→

0 0 0 0 0 0 0 0 0 0 0 0 0 1 0 1 0
10 0 0 1 00 0 11 0 0 1 00 0 10 1
1 1 101 0 11 0 0 1 010 0 11 0 11
0 00 0 10 1 1 1 100 0 10 0 0
1 010

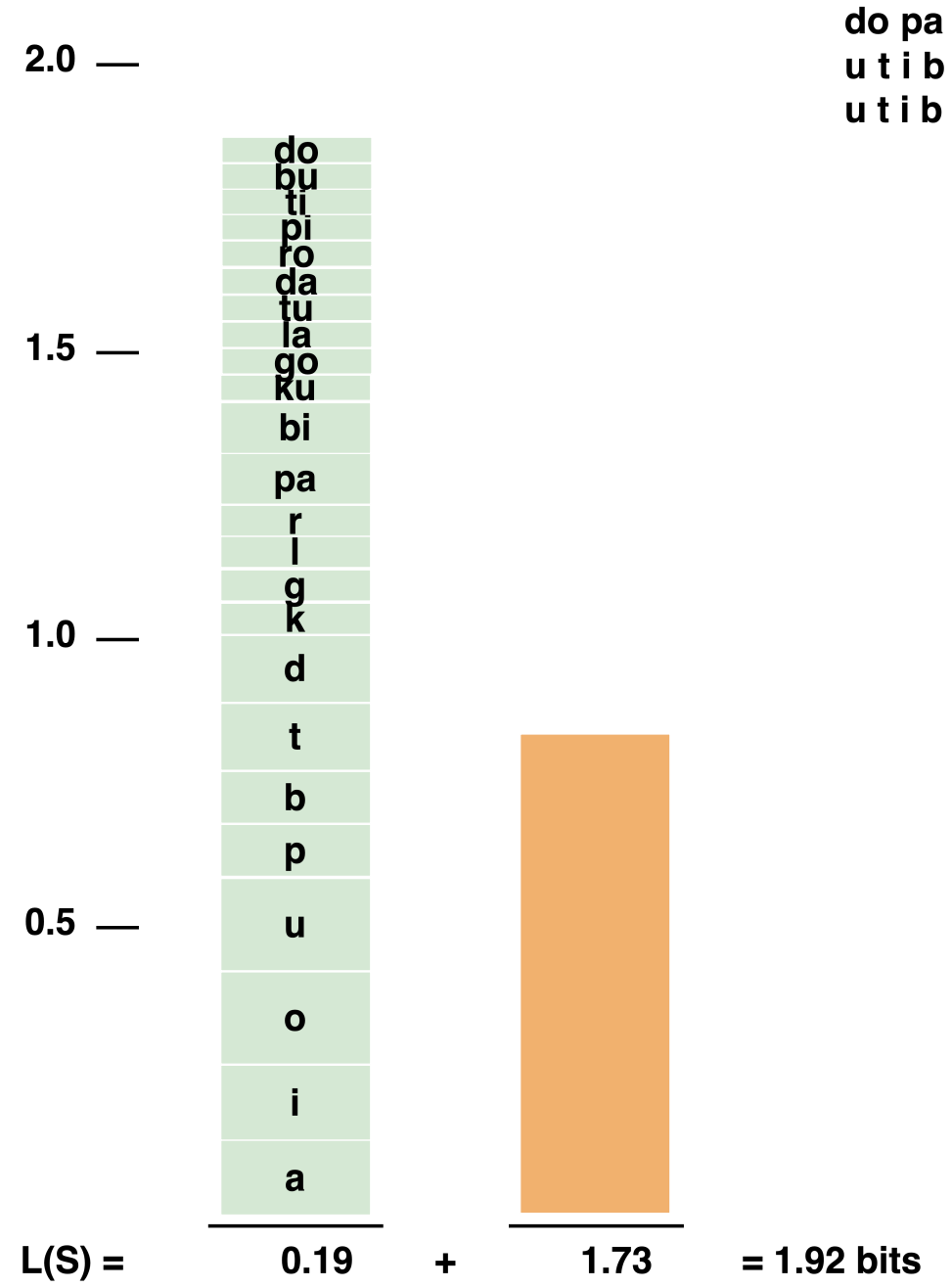

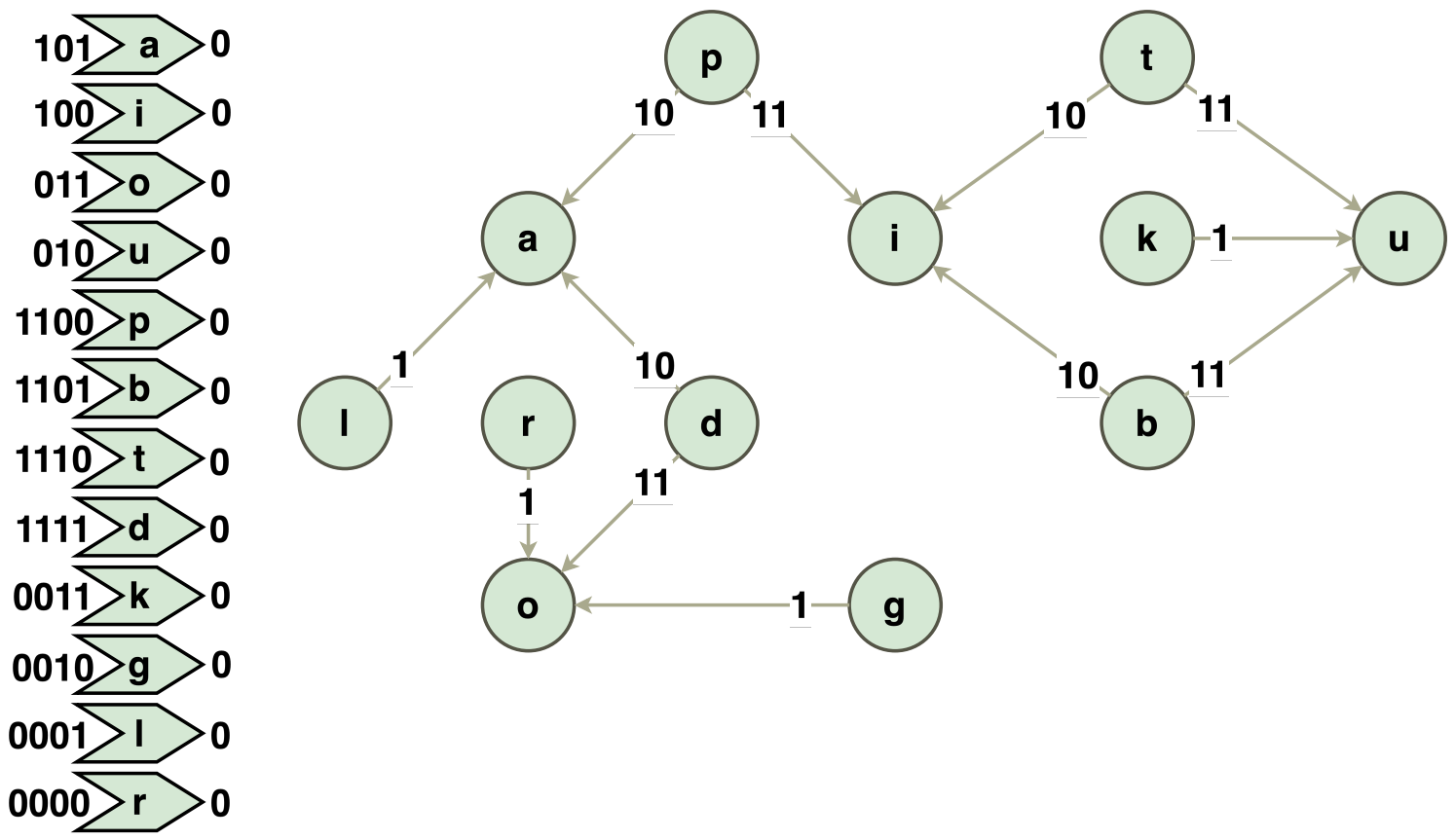

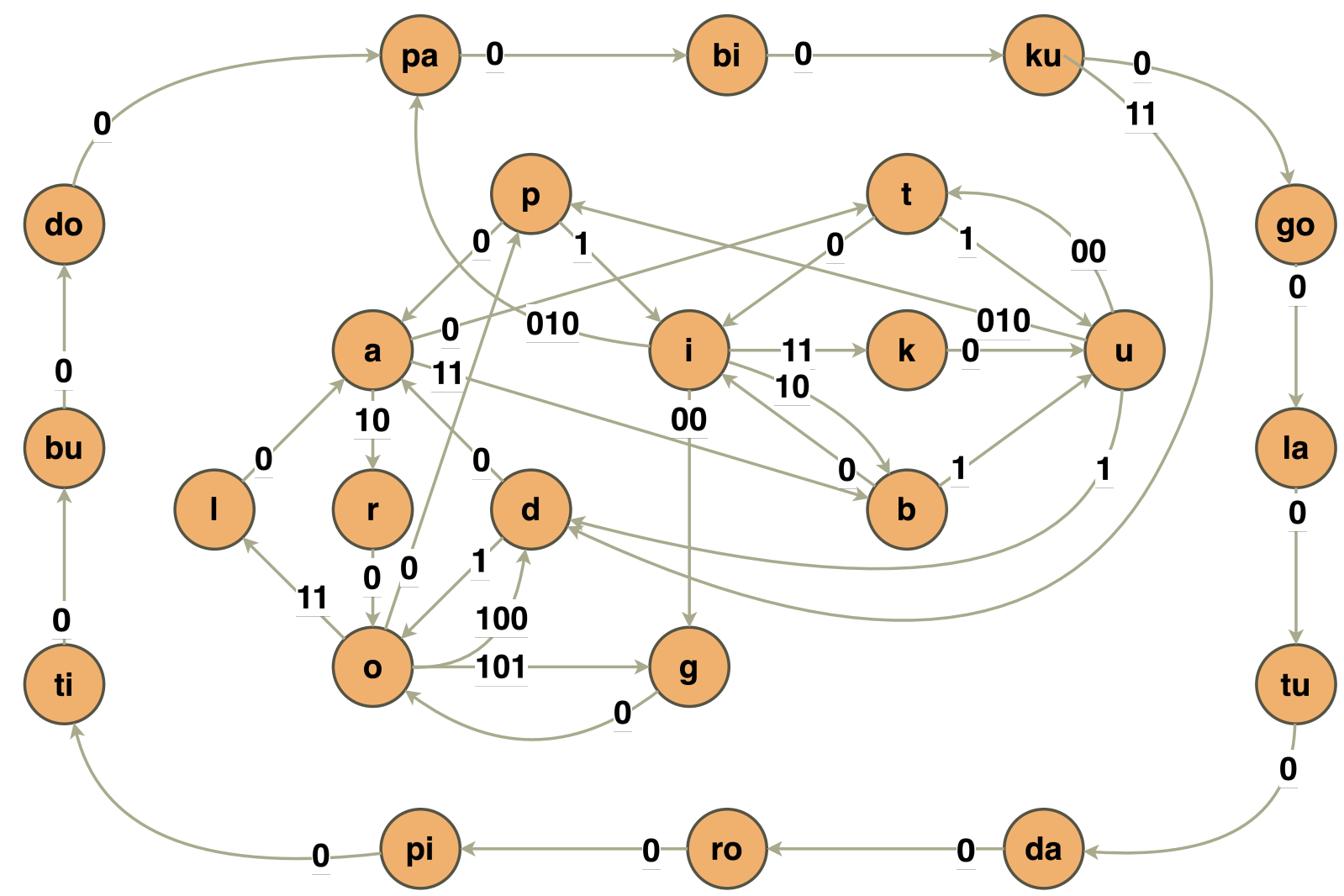

a →	101 0	p a →	1100 10 0
i →	100 0	b i →	1101 10 0
o →	011 0	k u →	0011 1 0
u →	010 0	go →	0010 1 0
p →	1100 0	l a →	0001 1 0
b →	1101 0	t u →	1110 11 0
t →	1110 0	d a →	1111 10 0
d →	1111 0	r o →	0000 1 0
k →	0011 0	p i →	1100 11 0
g →	0010 0	t i →	1110 10 0
l →	0001 0	b u →	1101 11 0
r →	0000 0	d o →	1111 11 0

pa bi ku go la tu da ro pi ti bu
do pa bi ku d a r o p i g o l a t
u t i b u d o g o l a t u p a b i k
u t i b u d o d a r o p i

→

0 0 0 0 0 0 0 0 0 0 0 0 0 0 11 0
10 0 0 1 00 0 11 0 0 1 00 0 10 1
1 1 101 0 11 0 0 1 010 0 11 0 11
0 00 0 10 1 1 1 100 0 10 0 0
1 010

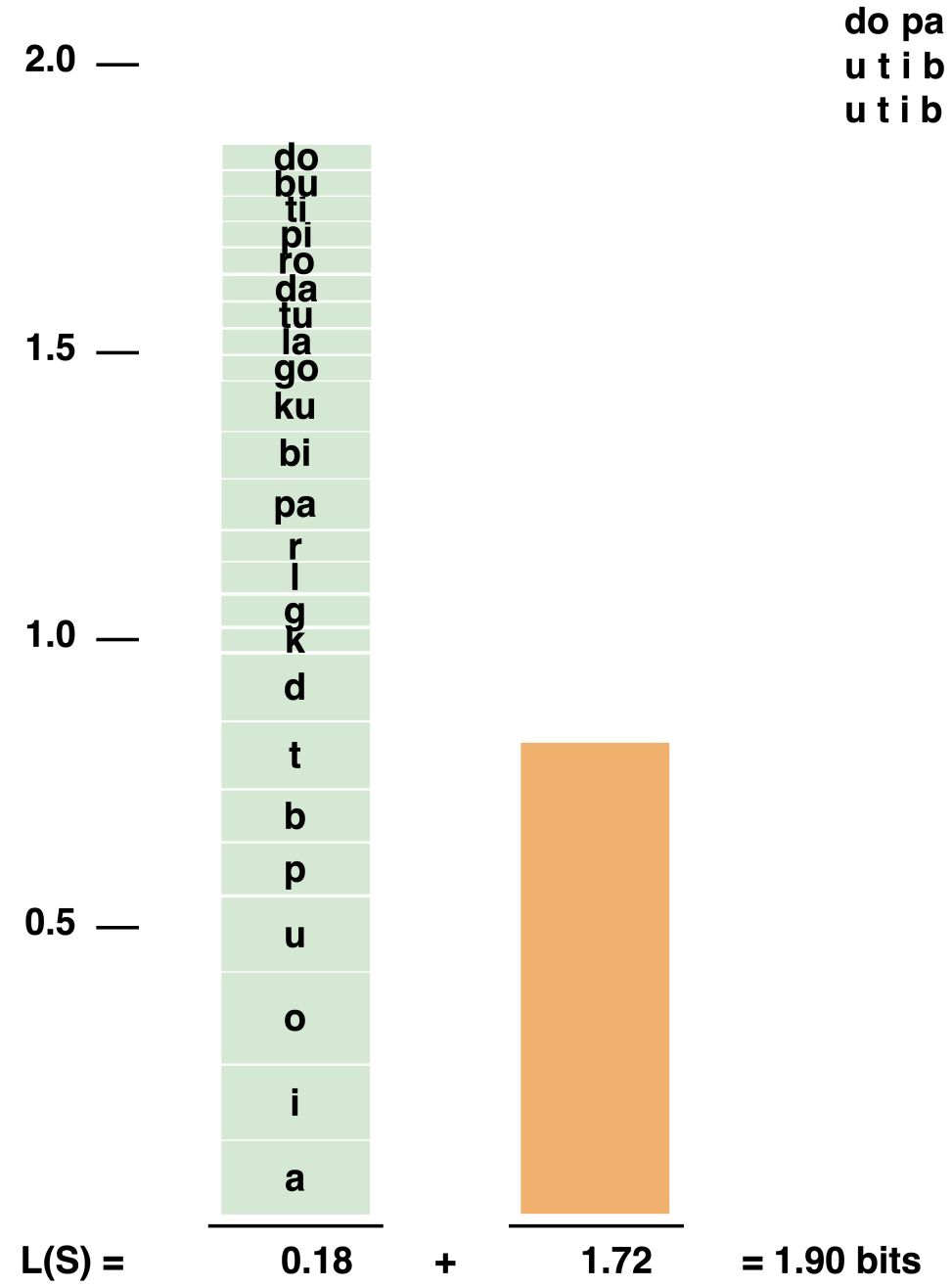

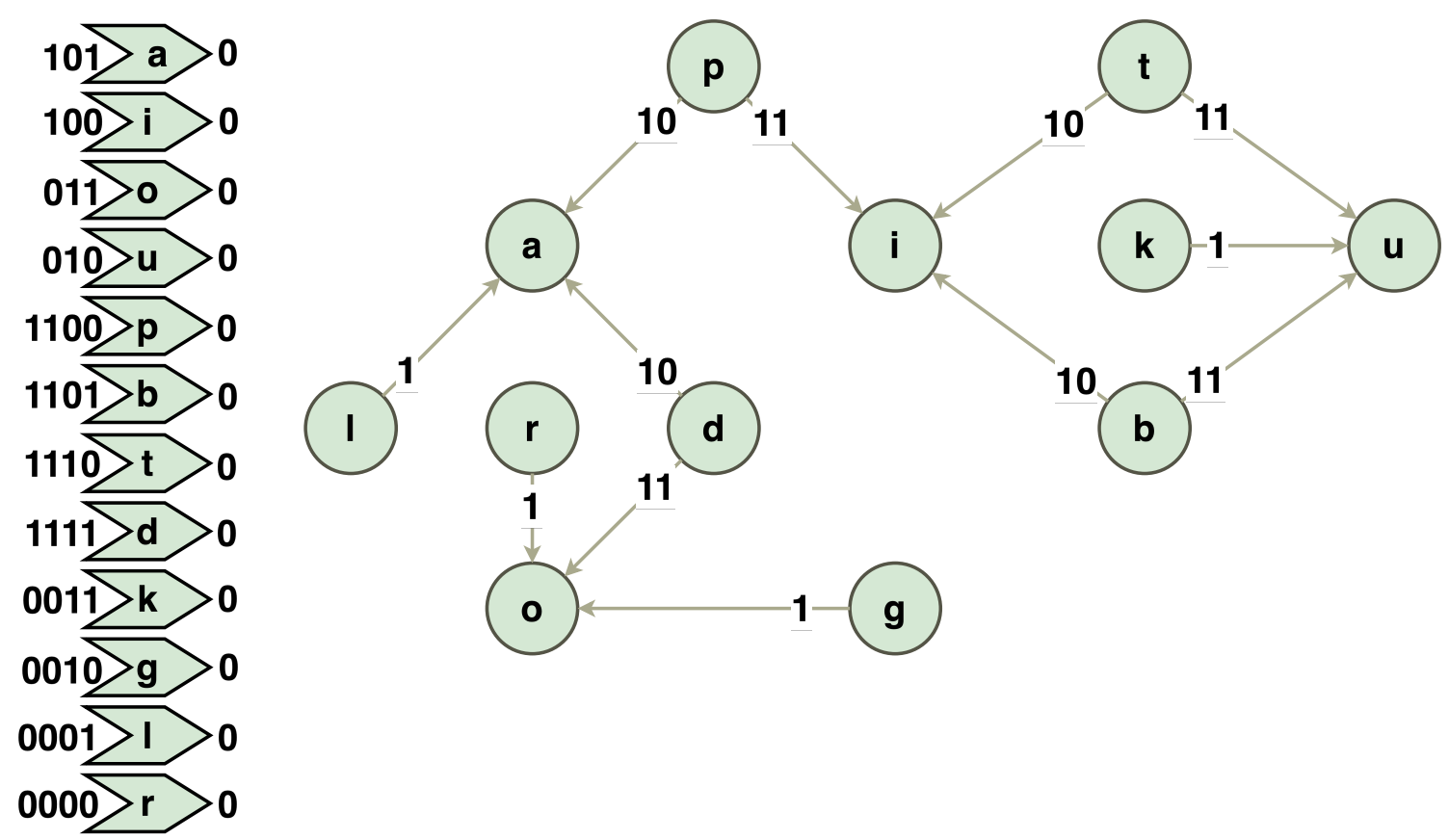

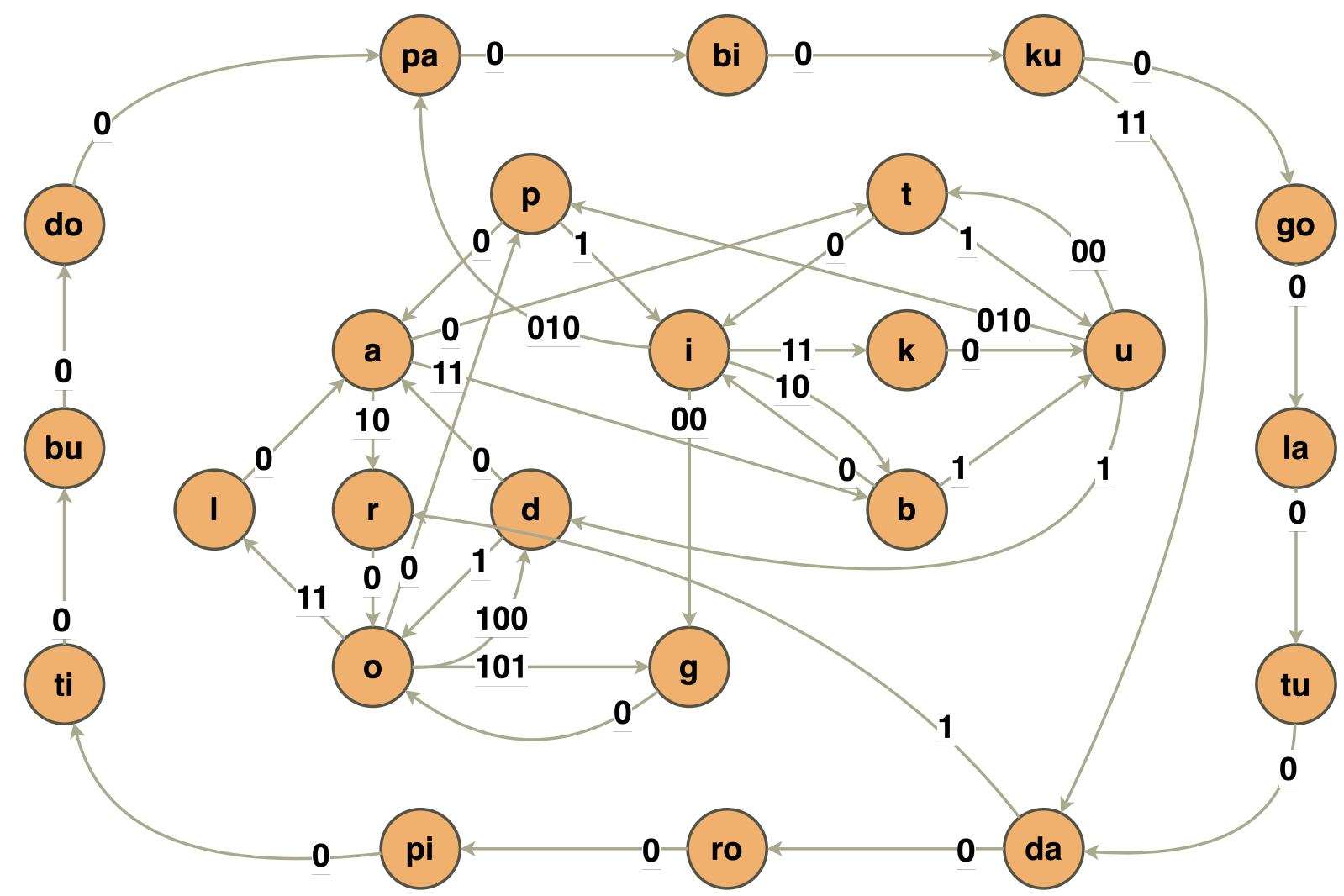

a	→	101 0	p a	→	1100 10 0
i	→	100 0	b i	→	1101 10 0
o	→	011 0	k u	→	0011 1 0
u	→	010 0	go	→	0010 1 0
p	→	1100 0	l a	→	0001 1 0
b	→	1101 0	t u	→	1110 11 0
t	→	1110 0	d a	→	1111 10 0
d	→	1111 0	r o	→	0000 1 0
k	→	0011 0	p i	→	1100 11 0
g	→	0010 0	t i	→	1110 10 0
l	→	0001 0	b u	→	1101 11 0
r	→	0000 0	d o	→	1111 11 0

pa bi ku go la tu da ro pi ti bu do pa bi ku da r o p i g o l a t u t i b u d o g o l a t u p a b i k u t i b u d o d a r o p i

→

0 0 0 0 0 0 0 0 0 0 0 0 0 0 11 1 0 0 1 00 0 11 0 0 1 00 0 10 1 1 1 101 0 11 0 0 1 010 0 11 0 11 0 00 0 10 1 1 1 100 0 10 0 0 1 010

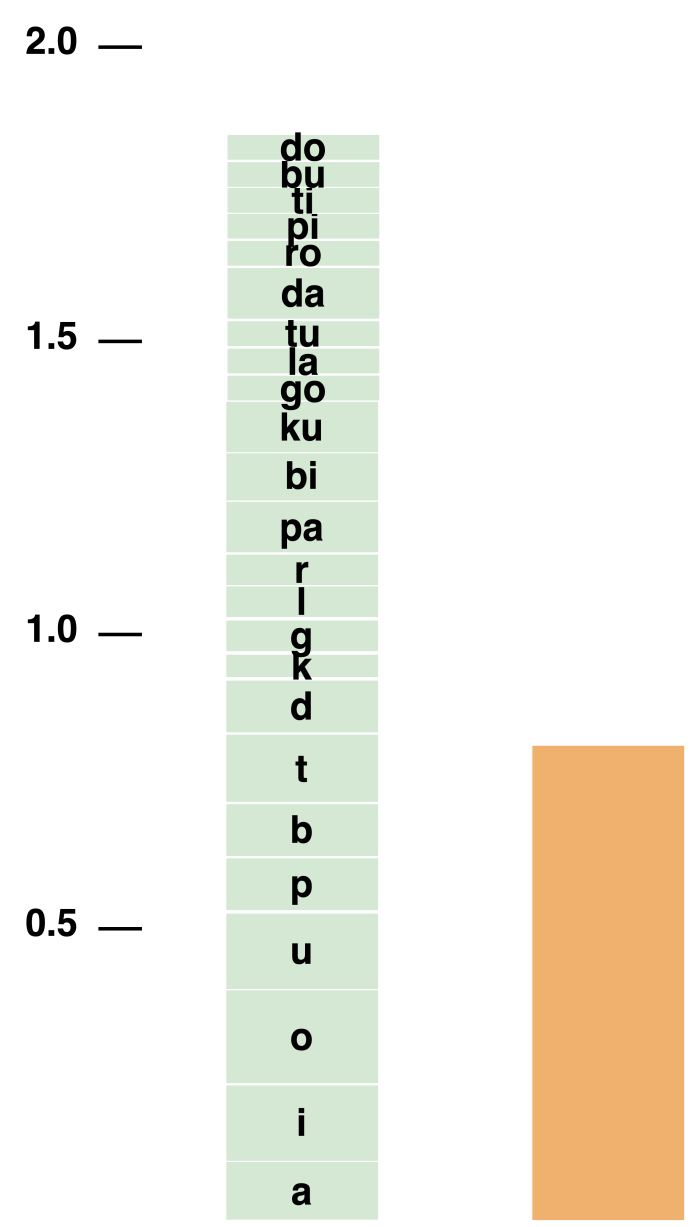

L(S) = 0.17 + 1.71 = 1.88 bits

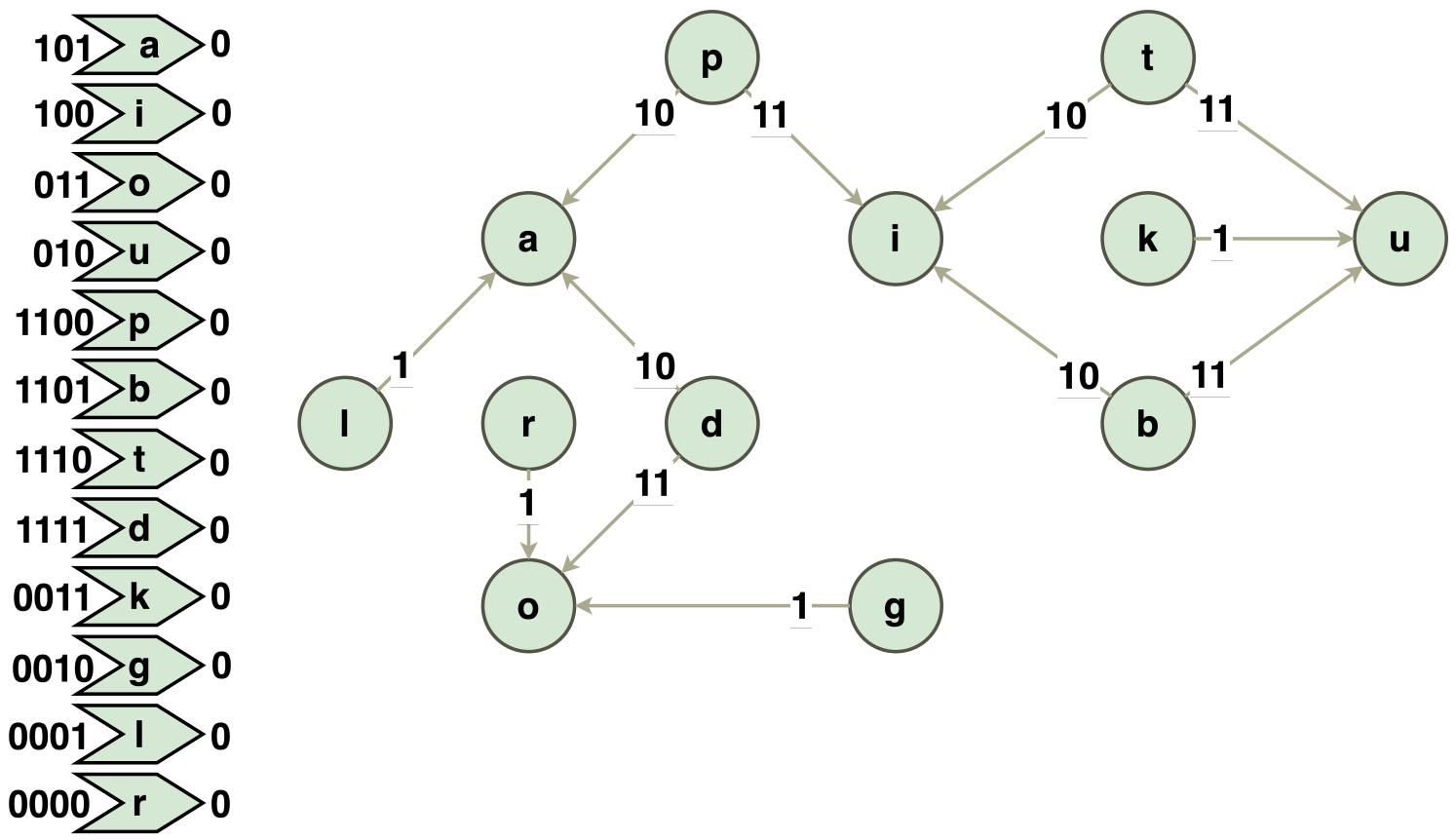

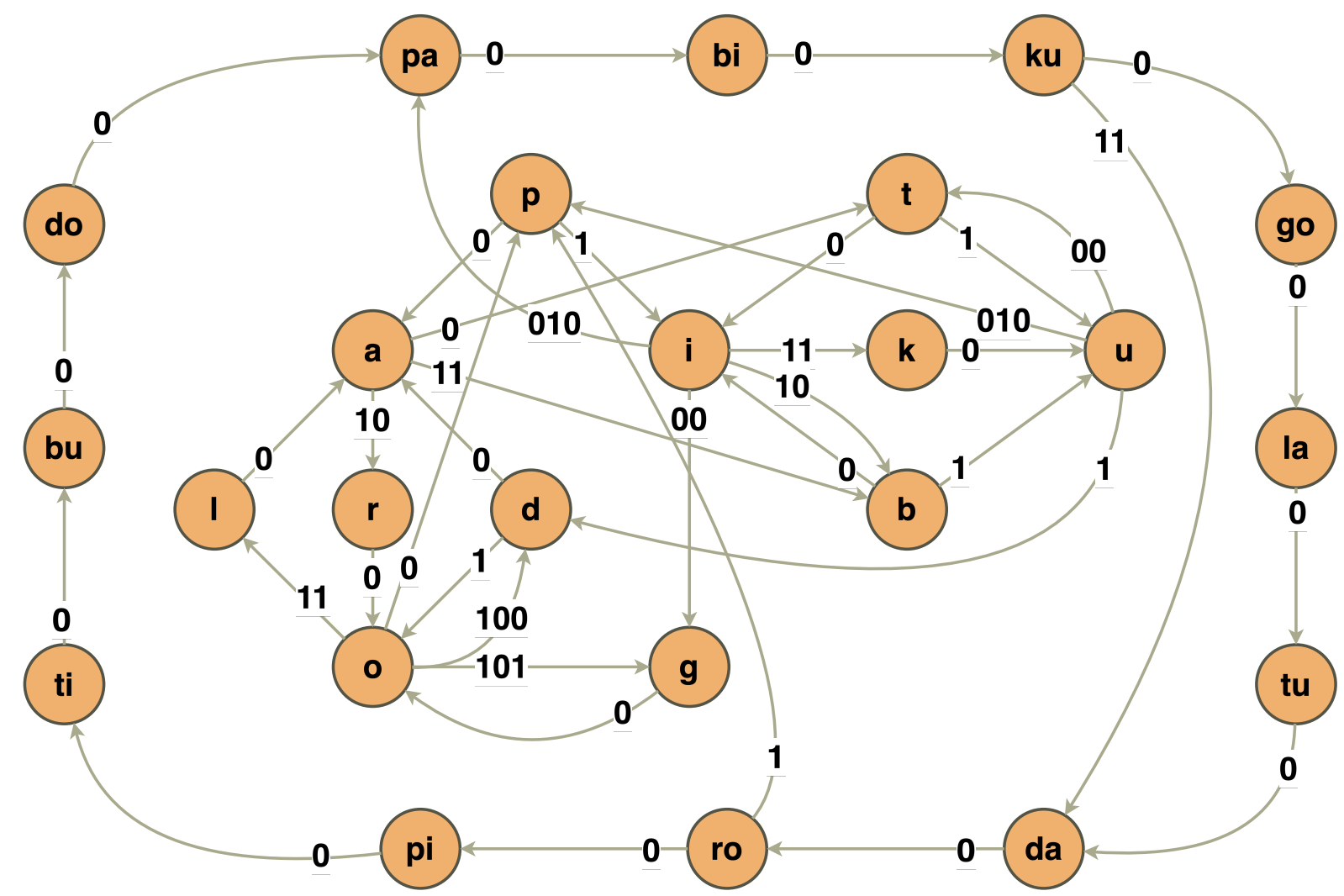

a	101 0	p a	1100 10 0
i	100 0	b i	1101 10 0
o	011 0	k u	0011 1 0
u	010 0	go	0010 1 0
p	1100 0	l a	0001 1 0
b	1101 0	t u	1110 11 0
t	1110 0	d a	1111 10 0
d	1111 0	r o	0000 1 0
k	0011 0	p i	1100 11 0
g	0010 0	t i	1110 10 0
l	0001 0	b u	1101 11 0
r	0000 0	d o	1111 11 0

pa bi ku go la tu da ro pi ti bu do pa bi ku da ro p i g o l a t u t i b u d o g o l a t u p a b i k u t i b u d o d a r o p i → 0 0 0 0 0 0 0 0 0 0 0 0 0 0 11 0 1 1 00 0 11 0 0 1 00 0 10 1 1 1 101 0 11 0 0 1 010 0 11 0 11 0 00 0 10 1 1 1 100 0 10 0 0 1 010

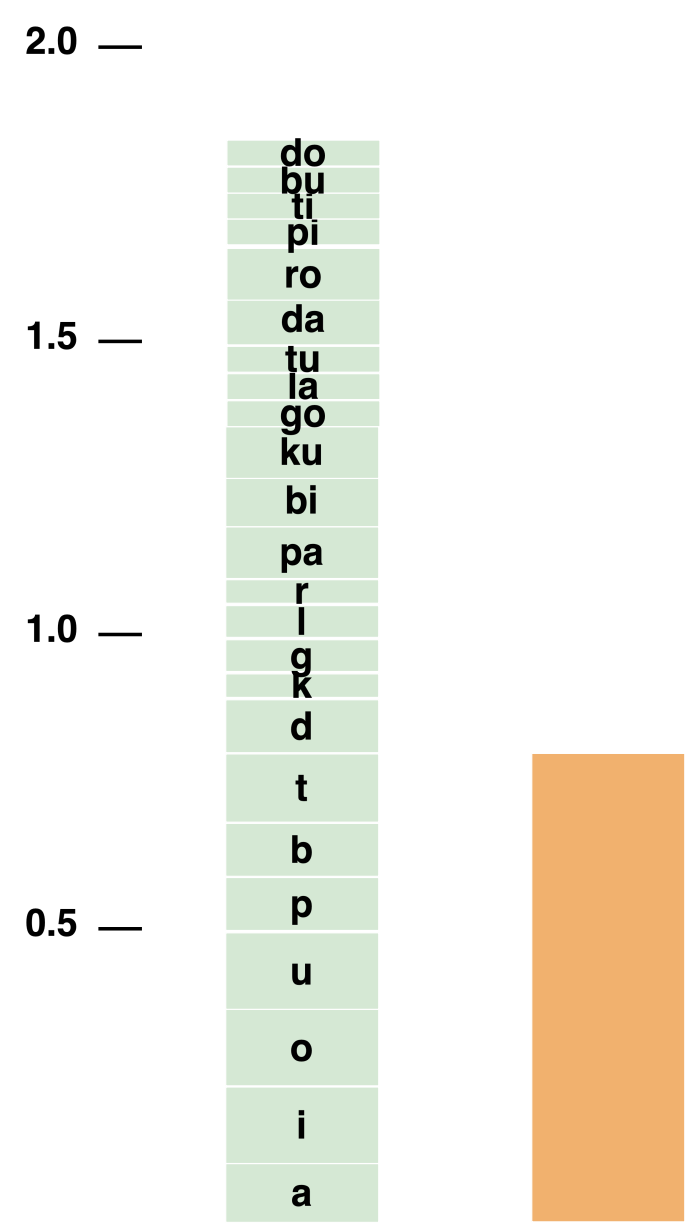

L(S) = 0.17 + 1.68 = 1.85 bits

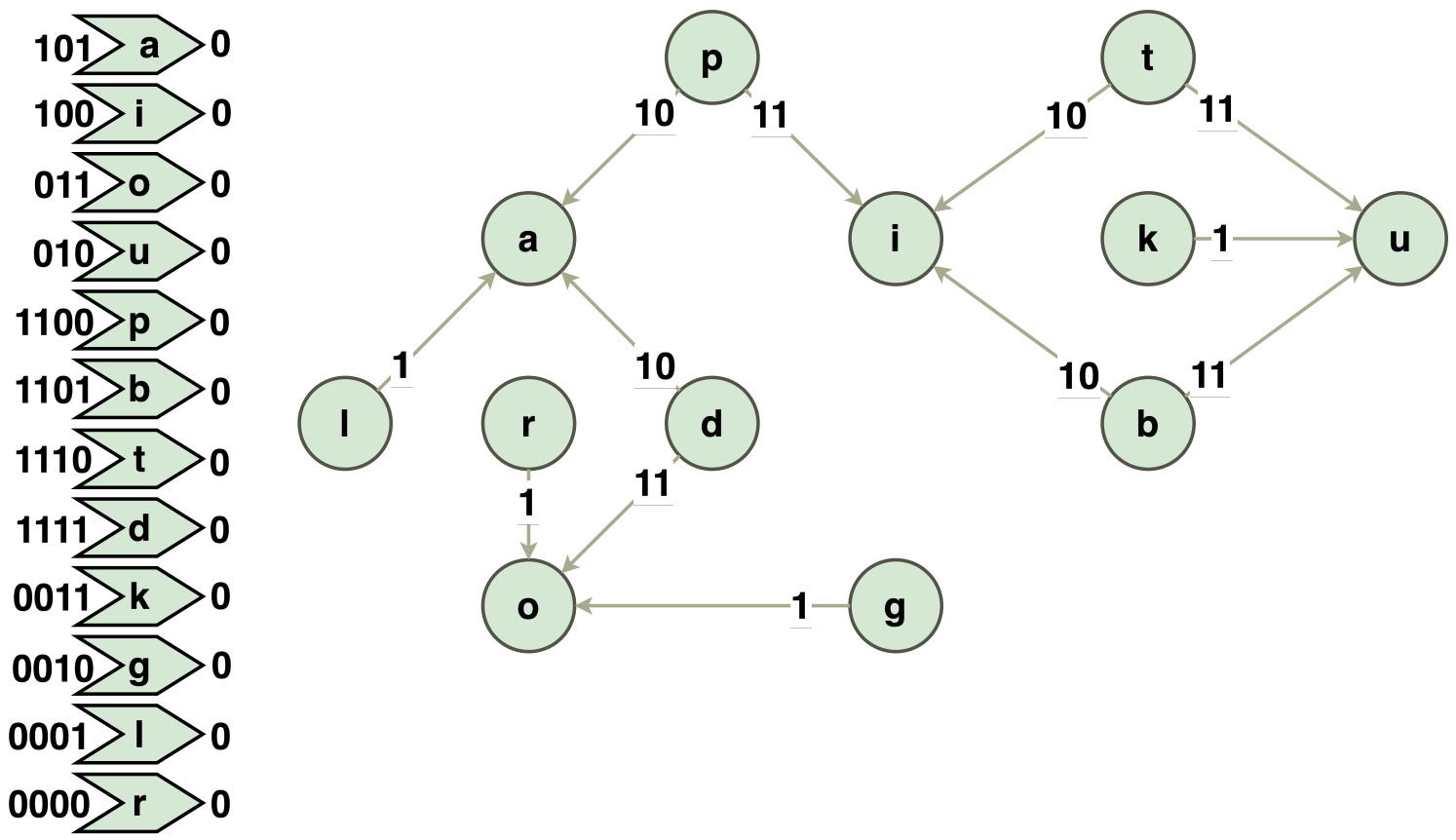

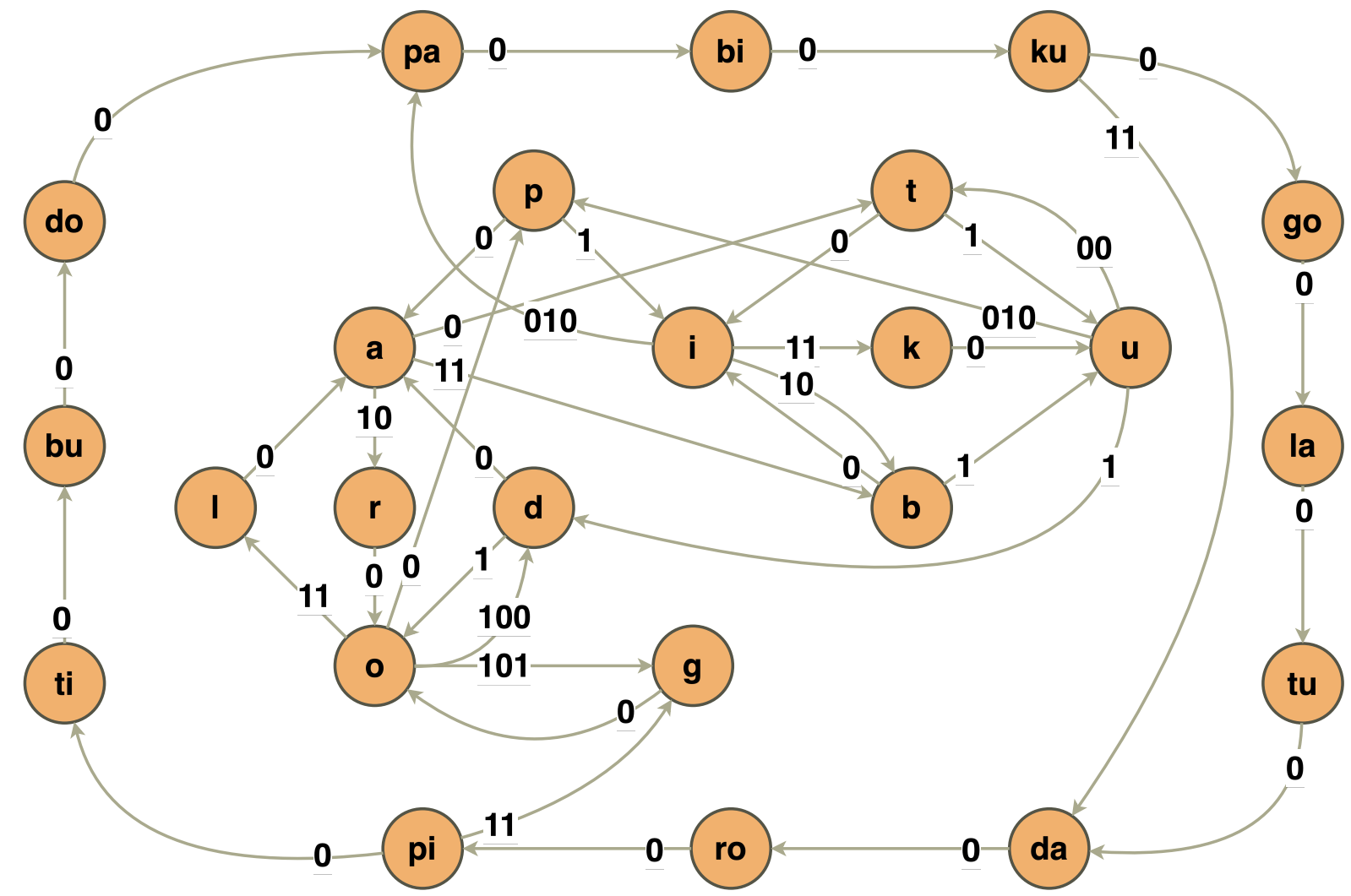

a	101 0		p a	1100 10 0
i	100 0		b i	1101 10 0
o	011 0		k u	0011 1 0
u	010 0		go	0010 1 0
p	1100 0		l a	0001 1 0
b	1101 0		t u	1110 11 0
t	1110 0		d a	1111 10 0
d	1111 0		r o	0000 1 0
k	0011 0		p i	1100 11 0
g	0010 0		t i	1110 10 0
l	0001 0		b u	1101 11 0
r	0000 0		d o	1111 11 0

pa bi ku go la tu da ro pi ti bu
do pa bi ku da ro pi g o l a t u
t i b u d o g o l a t u p a b i k u
t i b u d o d a r o p i

→

0 0 0 0 0 0 0 0 0 0 0 0 0 0 11 0
0 11 0 11 0 0 1 00 0 10 1 1
1 101 0 11 0 0 1 010 0 11 0 11
0 00 0 10 1 1 1 100 0 10 0 0
1 010

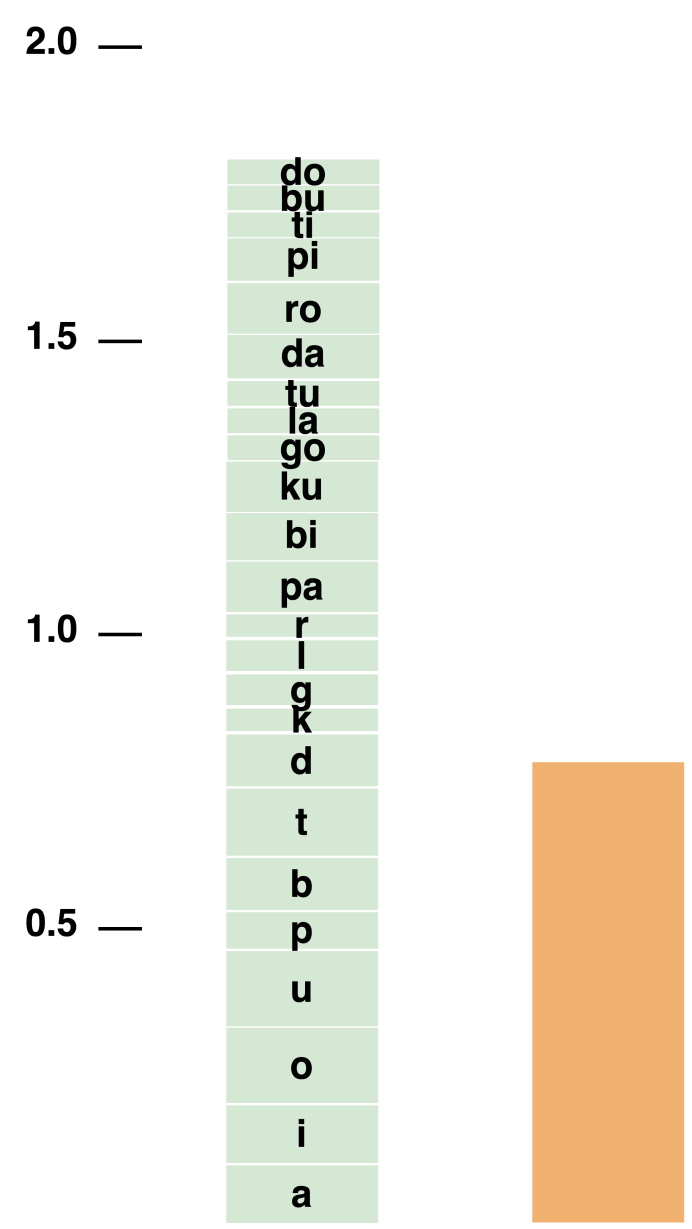

L(S) = 0.16 + 1.64 = 1.80 bits

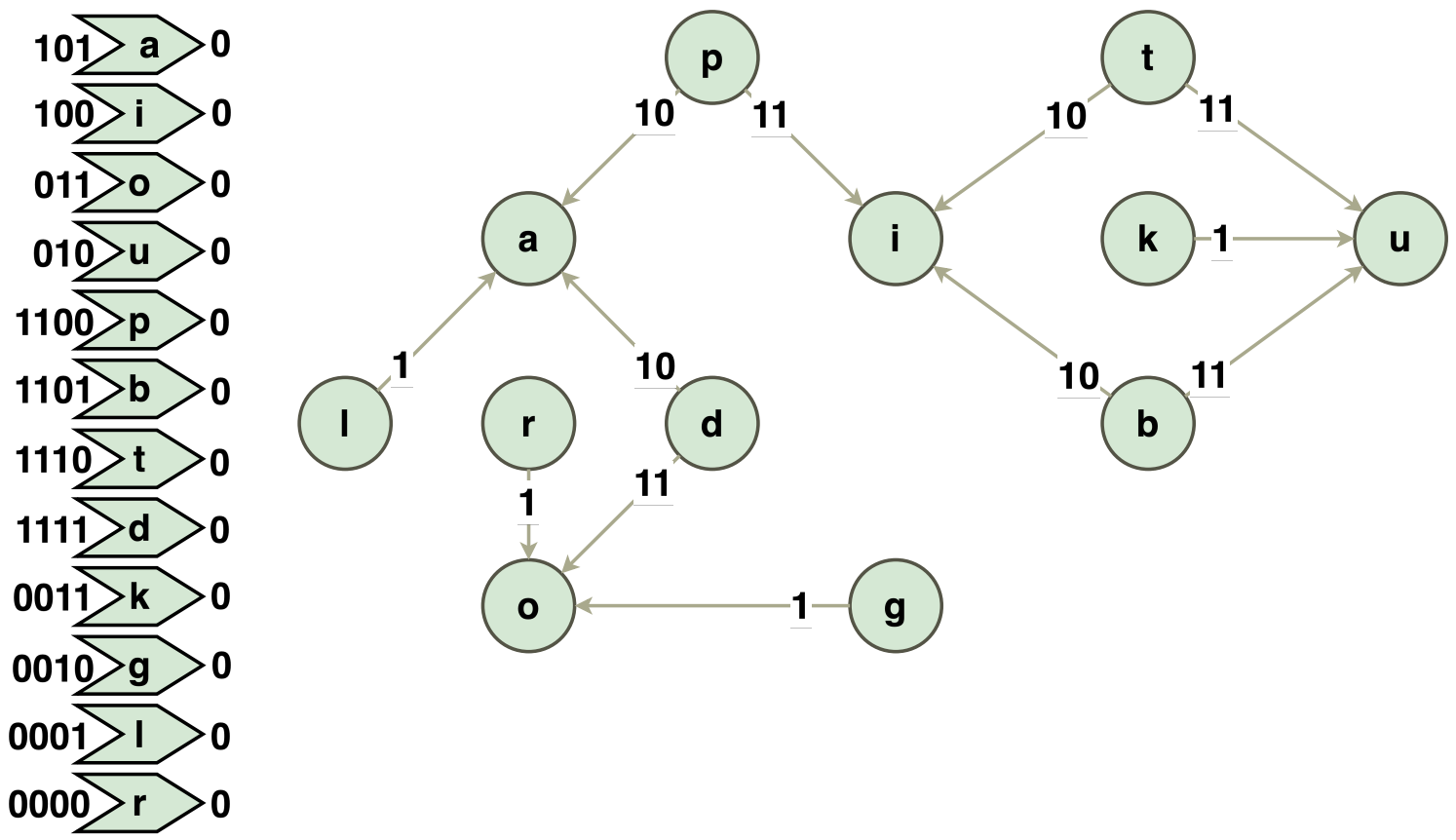

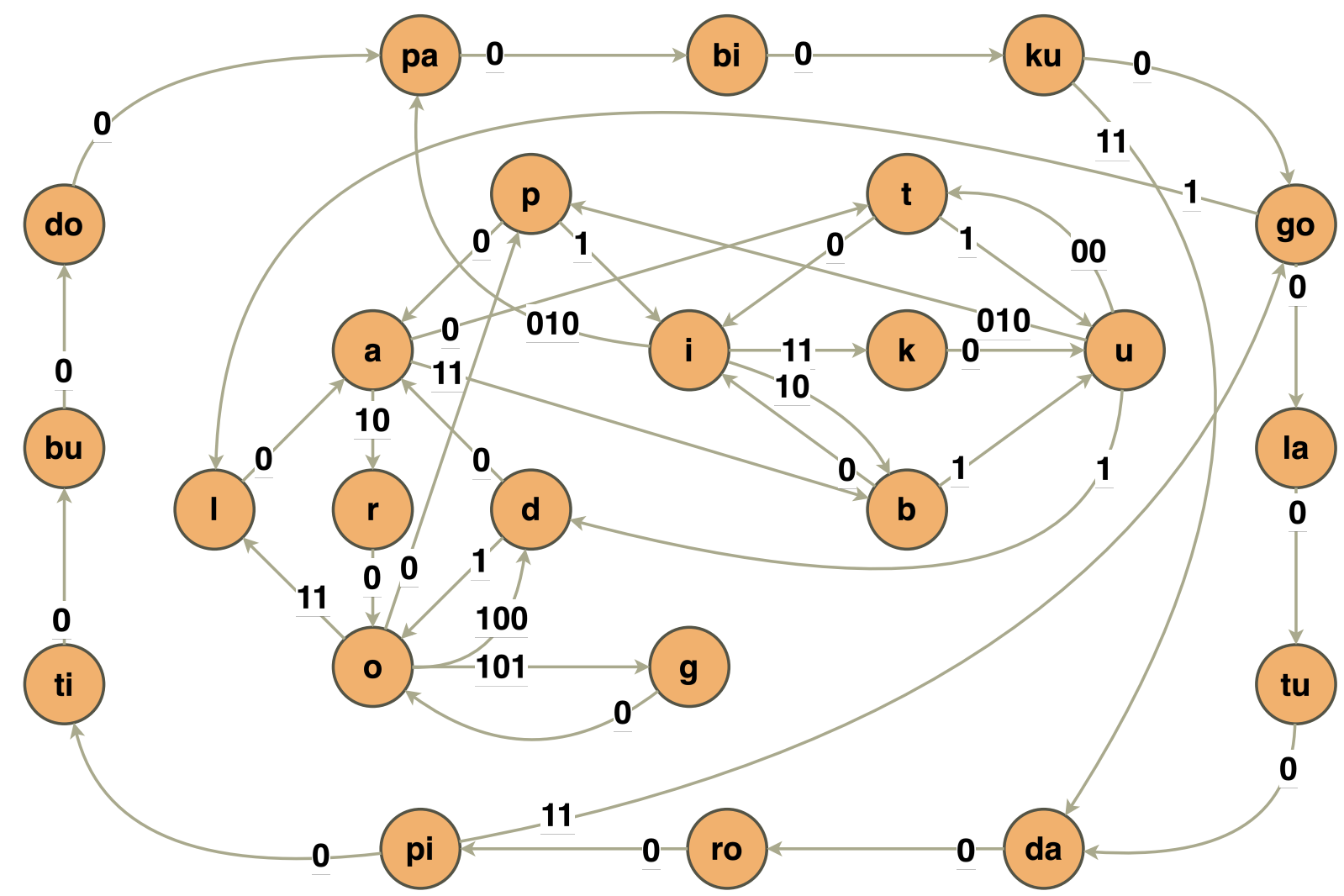

a	101 0		p a	1100 10 0
i	100 0		b i	1101 10 0
o	011 0		k u	0011 1 0
u	010 0		go	0010 1 0
p	1100 0		l a	0001 1 0
b	1101 0		t u	1110 11 0
t	1110 0		d a	1111 10 0
d	1111 0		r o	0000 1 0
k	0011 0		p i	1100 11 0
g	0010 0		t i	1110 10 0
l	0001 0		b u	1101 11 0
r	0000 0		d o	1111 11 0

pa bi ku go la tu da ro pi ti bu
do pa bi ku da ro pi go l a t u t
i b u d o g o l a t u p a b i k u t
i b u d o d a r o p i

→

0 0 0 0 0 0 0 0 0 0 0 0 0 0 0 11 0
0 11 1 0 0 1 00 0 10 1 1 1 101 0
11 0 0 1 010 0 11 0 11 0 00 0 10
1 1 1 100 0 10 0 0 1 010

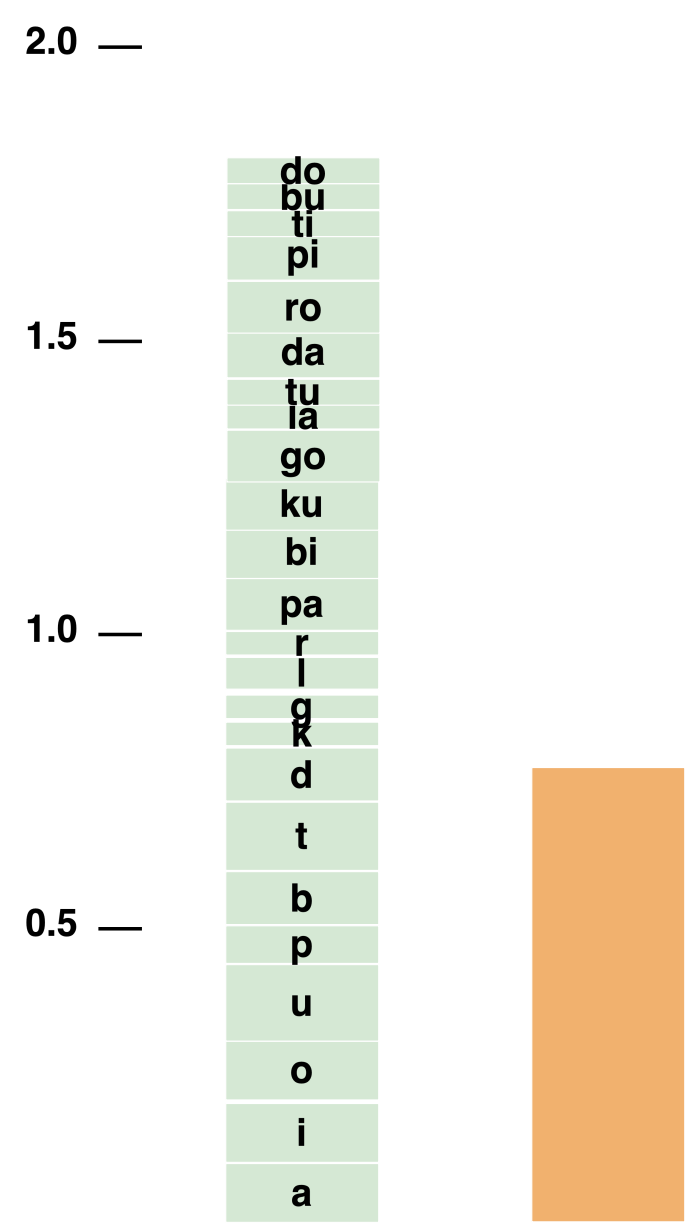

L(S) = 0.16 + 1.63 = 1.79 bits

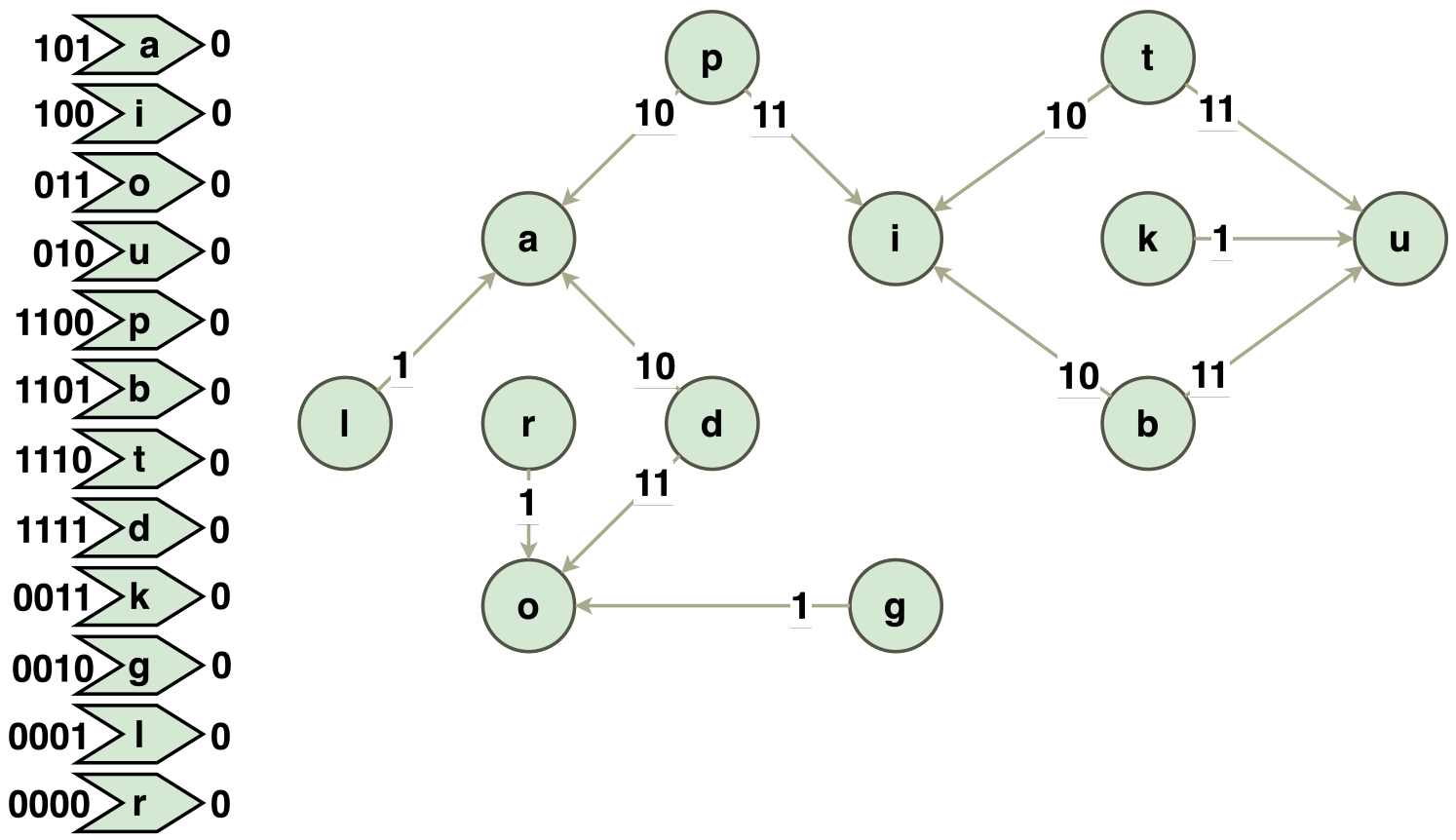

a	101 0	p a	1100 10 0
i	100 0	b i	1101 10 0
o	011 0	k u	0011 1 0
u	010 0	go	0010 1 0
p	1100 0	l a	0001 1 0
b	1101 0	t u	1110 11 0
t	1110 0	d a	1111 10 0
d	1111 0	r o	0000 1 0
k	0011 0	p i	1100 11 0
g	0010 0	t i	1110 10 0
l	0001 0	b u	1101 11 0
r	0000 0	d o	1111 11 0

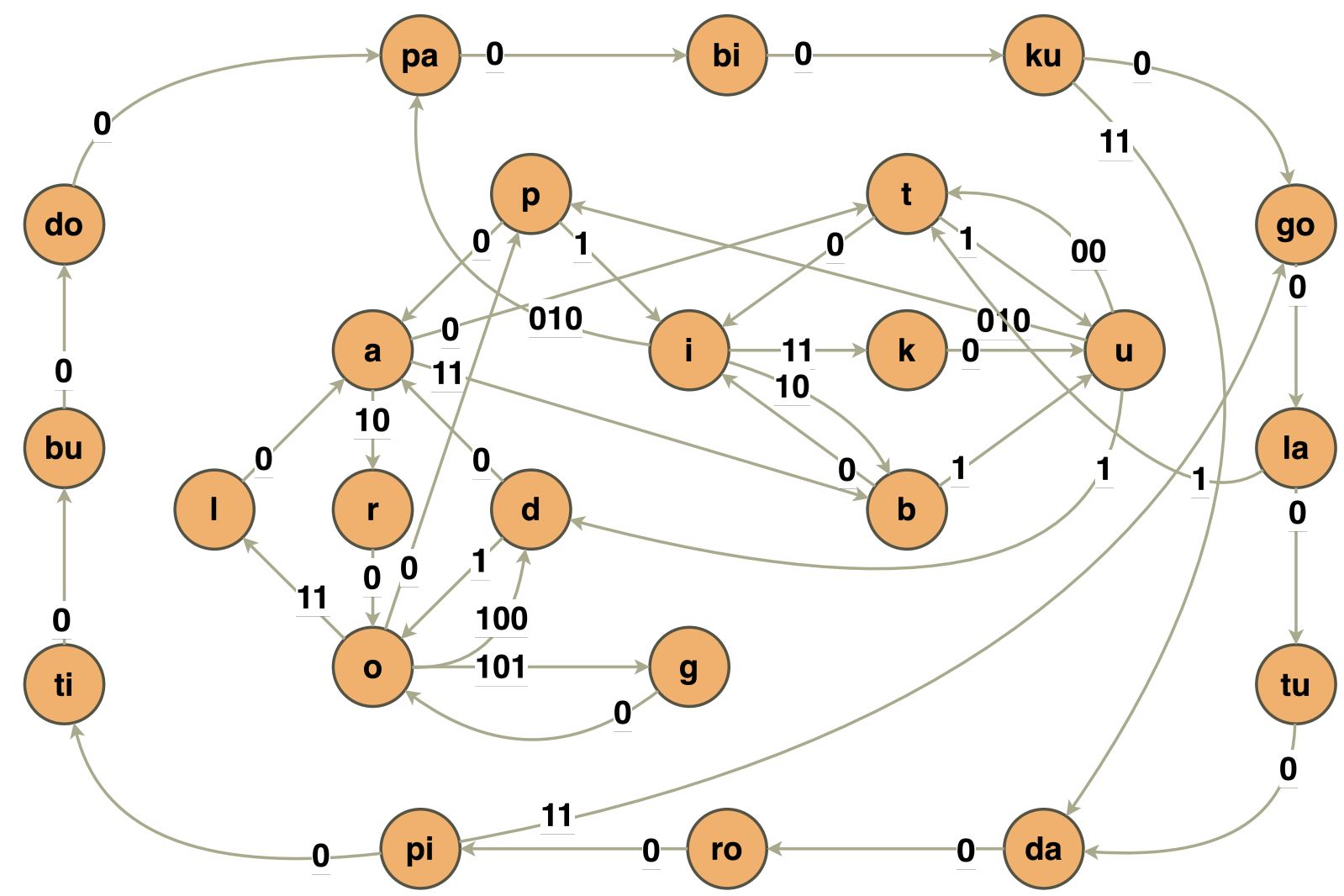

pa bi ku go la tu da ro pi ti bu do pa bi ku da ro pi go la t u t i b u d o g o l a t u p a b i k u t i b u d o d a r o p i → 0 0 0 0 0 0 0 0 0 0 0 0 0 0 11 0 0 11 0 1 1 00 0 10 1 1 1 101 0 11 0 0 1 010 0 11 0 11 0 00 0 10 1 1 1 100 0 10 0 0 1 010

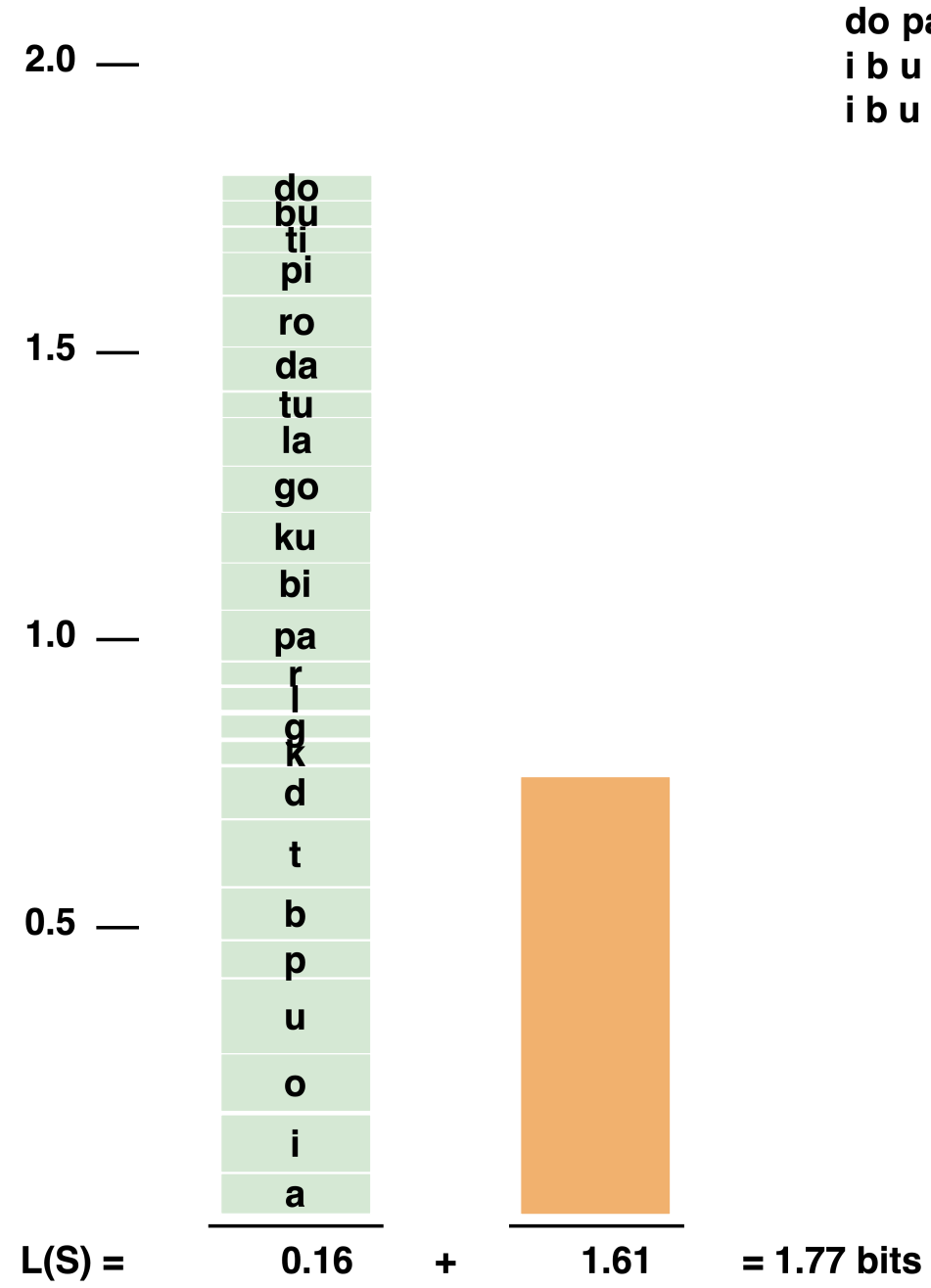

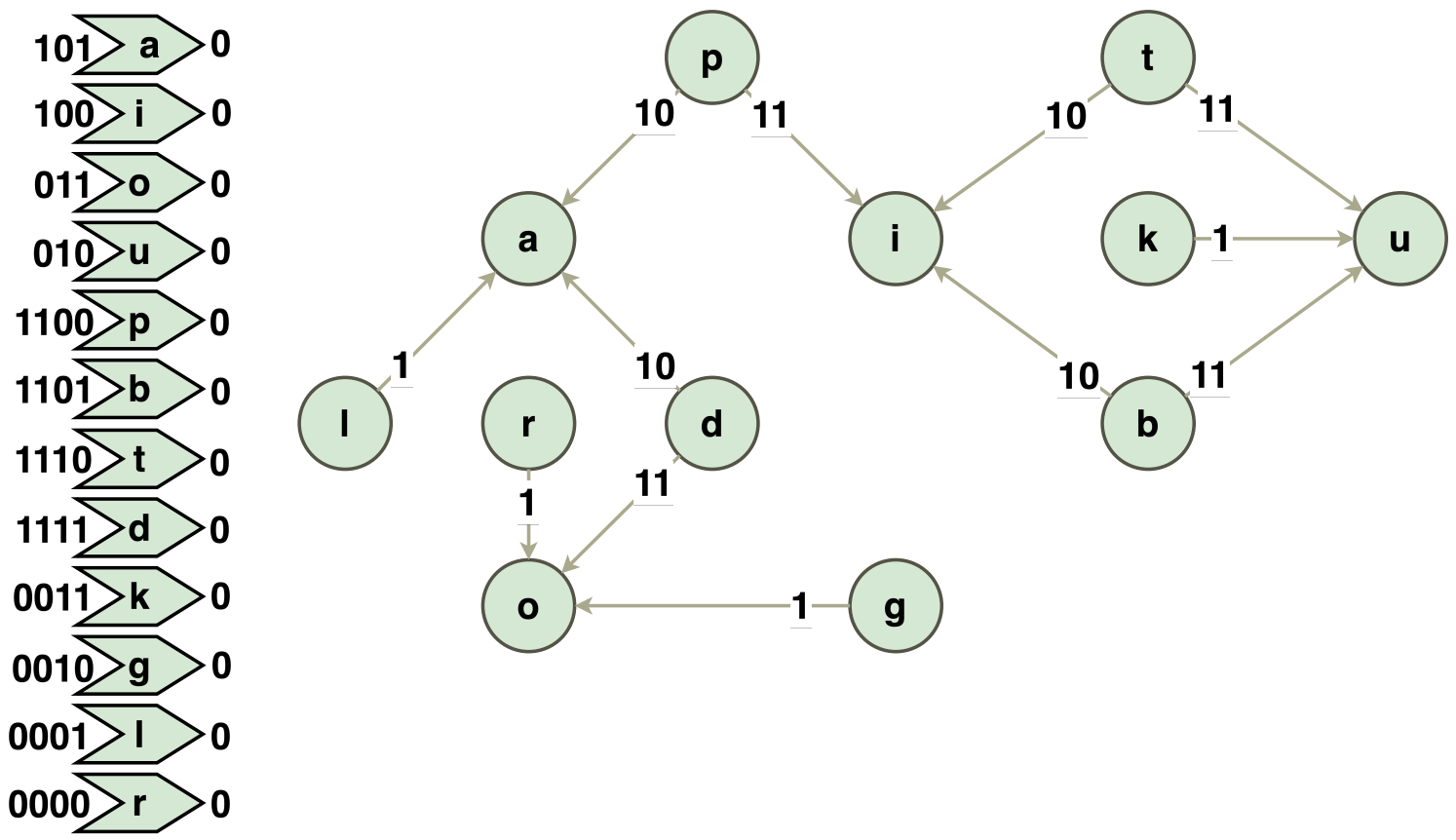

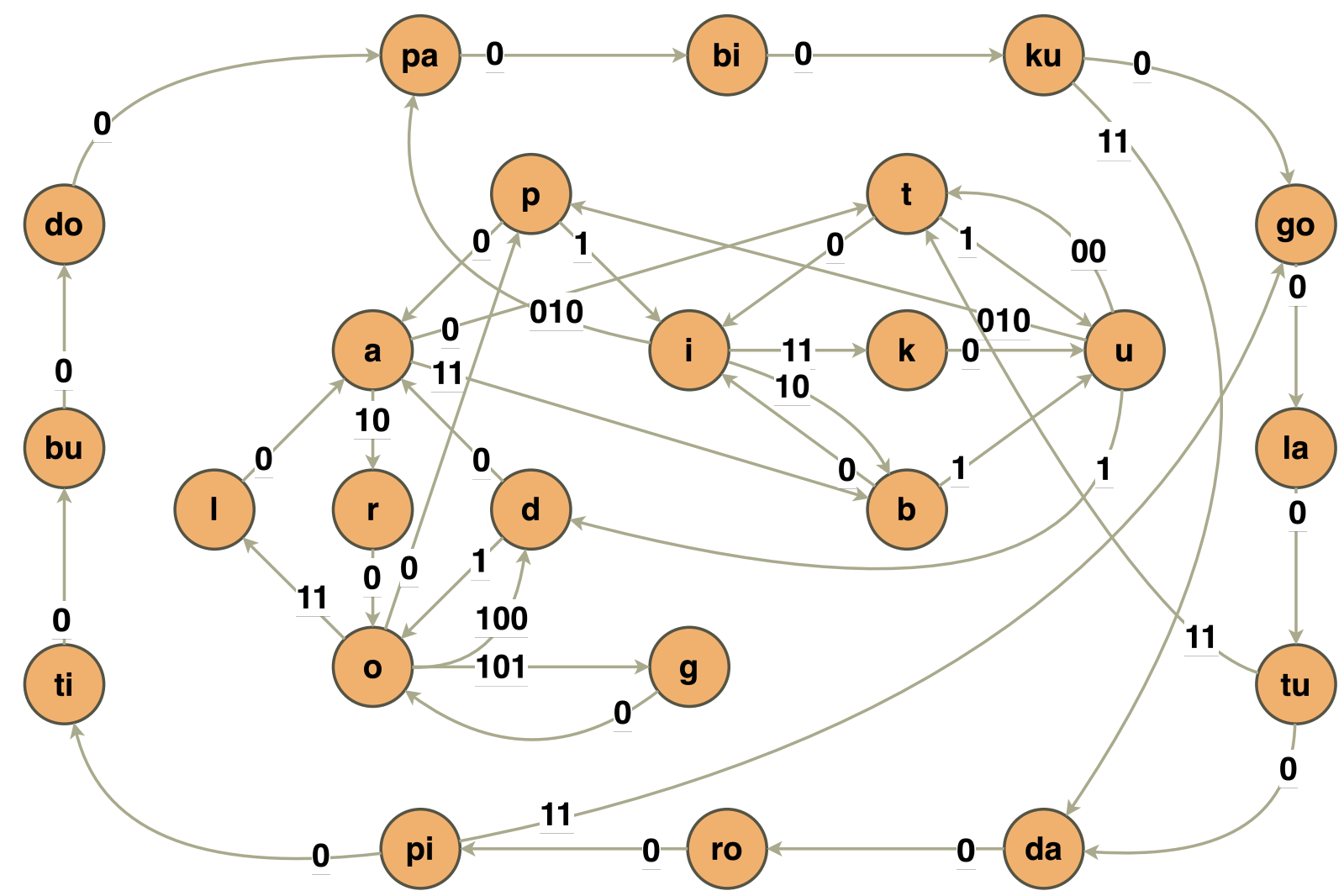

a	→ 101 0		p a	→ 1100 10 0
i	→ 100 0		b i	→ 1101 10 0
o	→ 011 0		k u	→ 0011 1 0
u	→ 010 0		go	→ 0010 1 0
p	→ 1100 0		l a	→ 0001 1 0
b	→ 1101 0		t u	→ 1110 11 0
t	→ 1110 0		d a	→ 1111 10 0
d	→ 1111 0		r o	→ 0000 1 0
k	→ 0011 0		p i	→ 1100 11 0
g	→ 0010 0		t i	→ 1110 10 0
l	→ 0001 0		b u	→ 1101 11 0
r	→ 0000 0		d o	→ 1111 11 0

pa bi ku go la tu da ro pi ti bu
do pa bi ku da ro pi go la tu t i
b u d o g o l a t u p a b i k u t i
b u d o d a r o p i

→

0 0 0 0 0 0 0 0 0 0 0 0 0 0 11 0
0 11 0 0 11 0 10 1 1 1 101 0 11 0
0 1 010 0 11 0 11 0 00 0 10 1 1
1 100 0 10 0 0 1 010

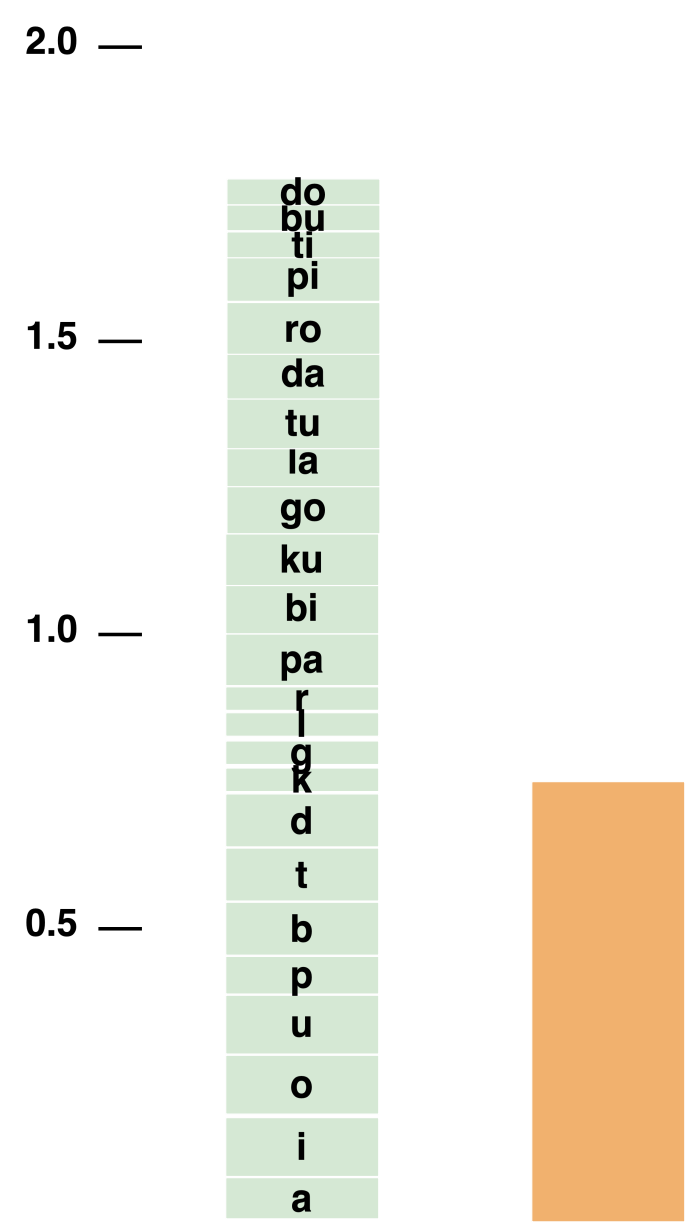

L(S) = 0.15 + 1.58 = 1.73 bits

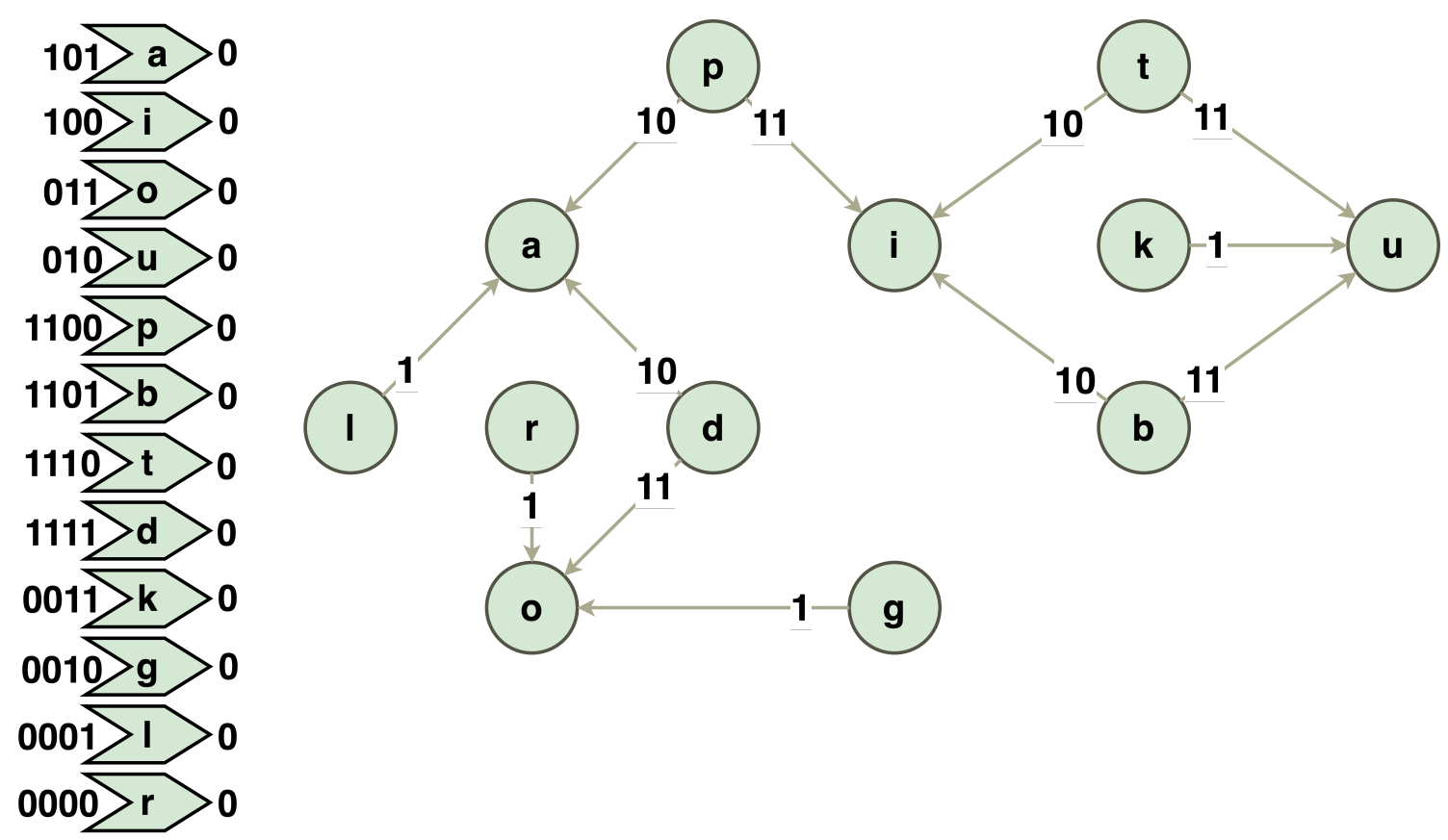

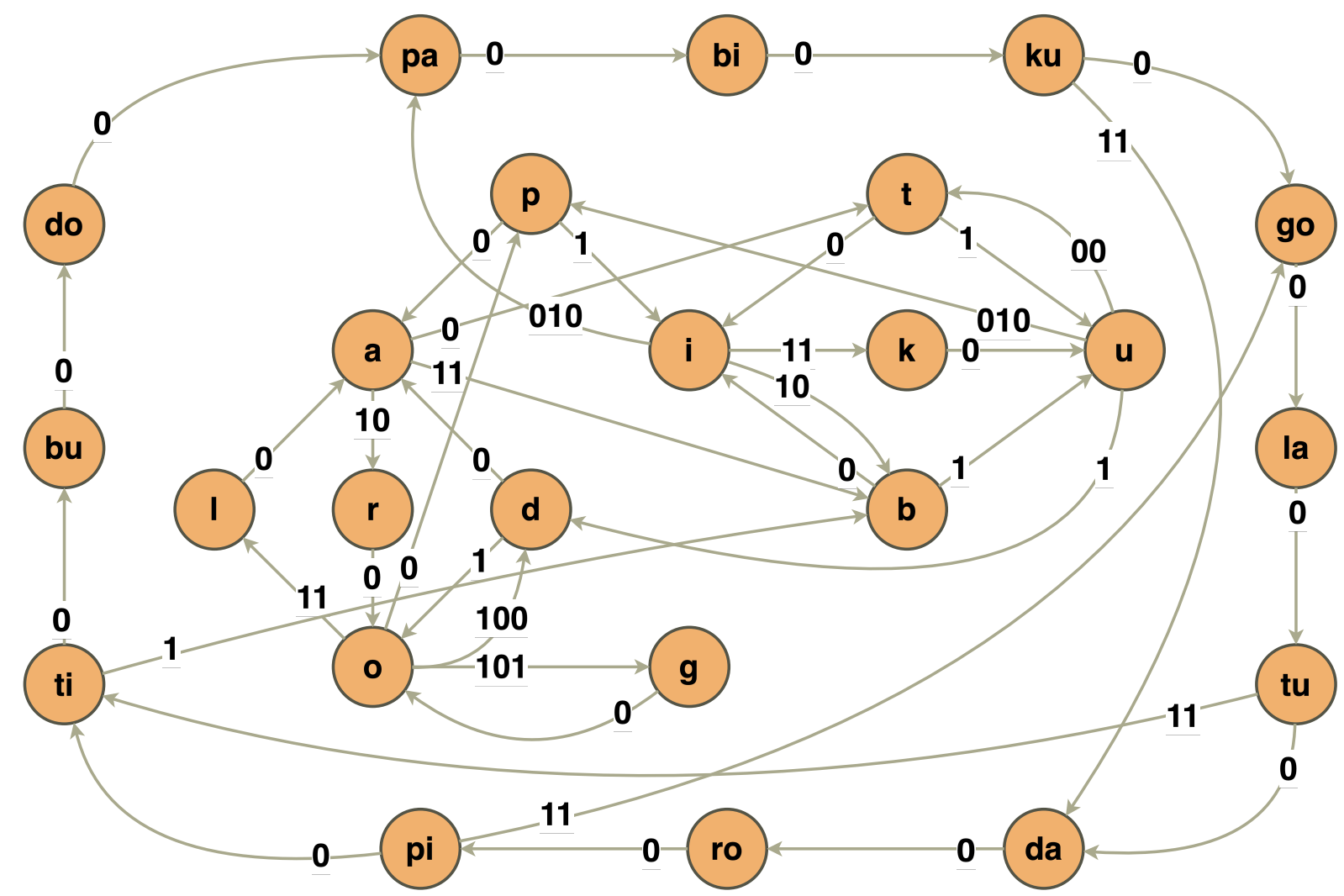

a	→	101 0	p a	→	1100 10 0
i	→	100 0	b i	→	1101 10 0
o	→	011 0	k u	→	0011 1 0
u	→	010 0	go	→	0010 1 0
p	→	1100 0	l a	→	0001 1 0
b	→	1101 0	t u	→	1110 11 0
t	→	1110 0	d a	→	1111 10 0
d	→	1111 0	r o	→	0000 1 0
k	→	0011 0	p i	→	1100 11 0
g	→	0010 0	t i	→	1110 10 0
l	→	0001 0	b u	→	1101 11 0
r	→	0000 0	d o	→	1111 11 0

pa bi ku go la tu da ro pi ti bu
do pa bi ku da ro pi go la tu ti
b u d o g o l a t u p a b i k u t i
b u d o d a r o p i

→

0 0 0 0 0 0 0 0 0 0 0 0 0 0 11 0
0 11 0 0 11 1 1 1 1 101 0 11 0 0
1 010 0 11 0 11 0 00 0 10 1 1
1 100 0 10 0 0 1 010

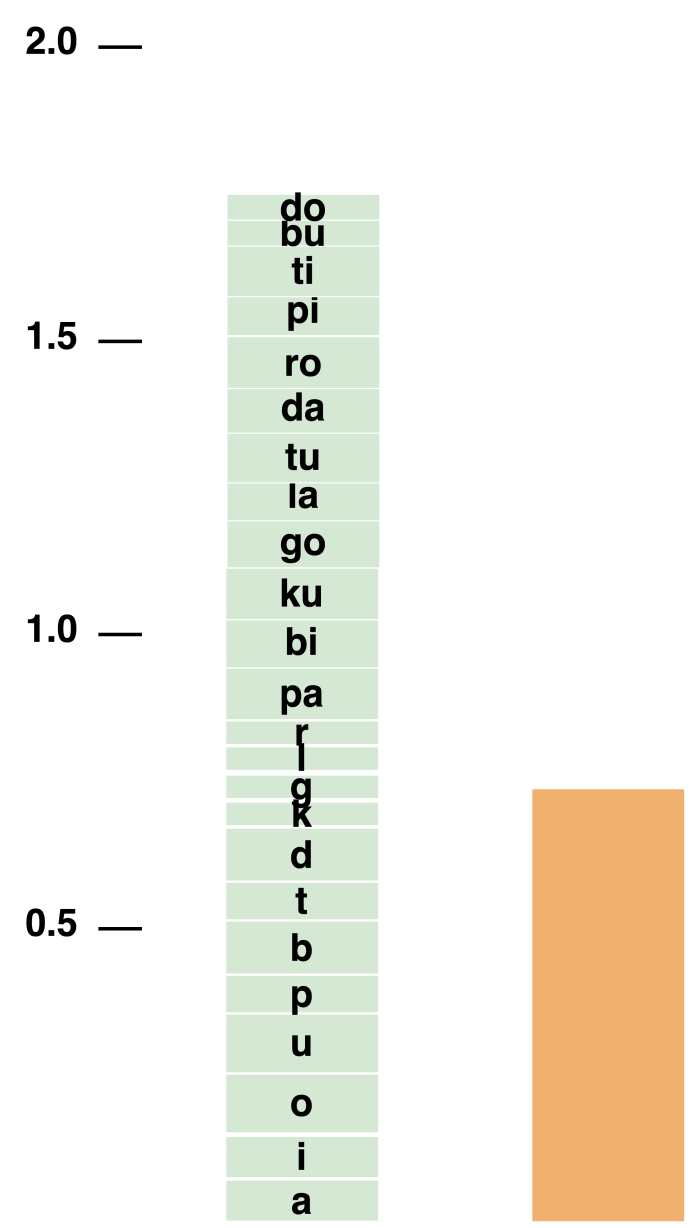

L(S) = 0.15 + 1.57 = 1.72 bits

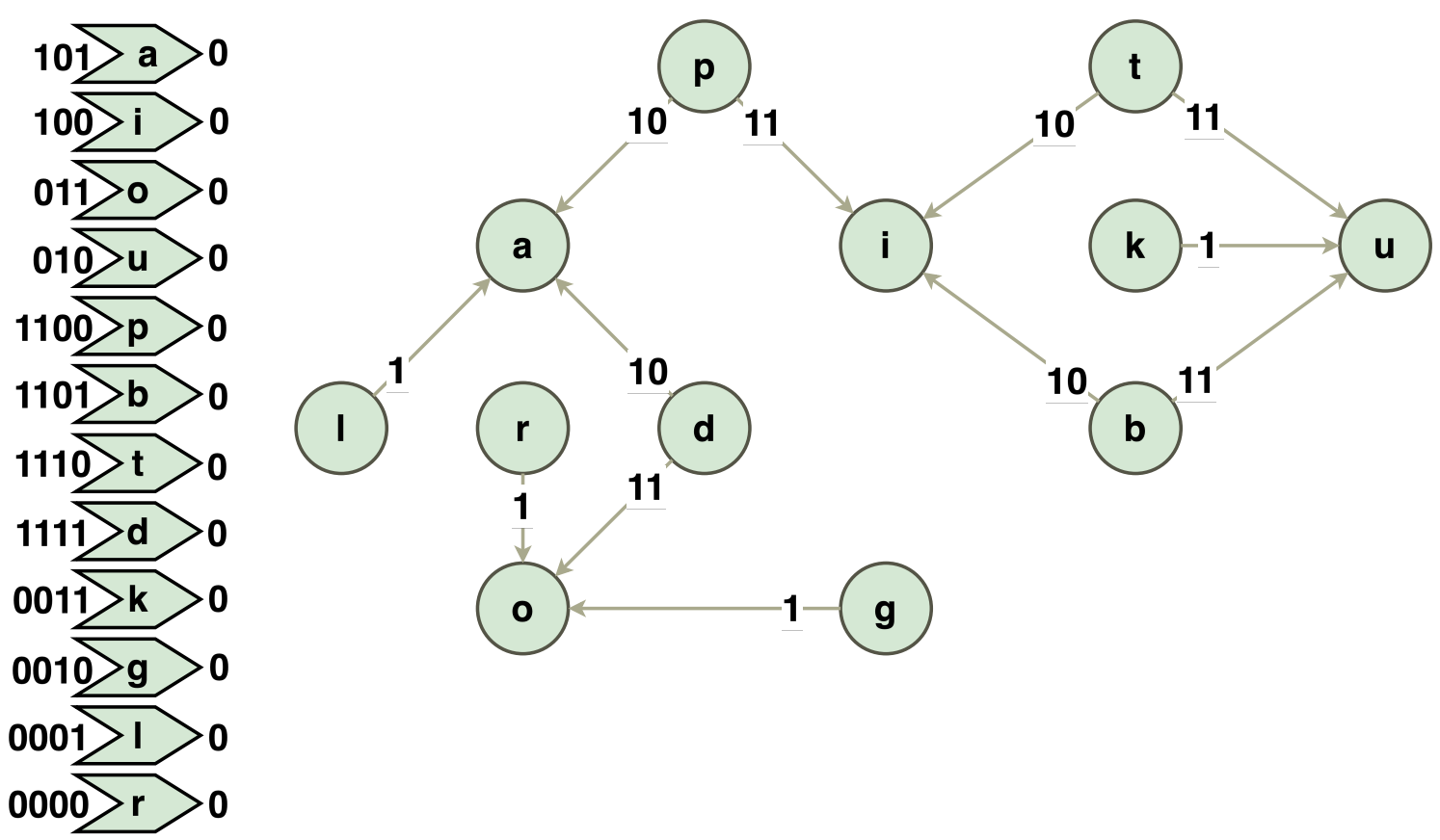

a	101 0	p a	1100 10 0
i	100 0	b i	1101 10 0
o	011 0	k u	0011 1 0
u	010 0	go	0010 1 0
p	1100 0	l a	0001 1 0
b	1101 0	t u	1110 11 0
t	1110 0	d a	1111 10 0
d	1111 0	r o	0000 1 0
k	0011 0	p i	1100 11 0
g	0010 0	t i	1110 10 0
l	0001 0	b u	1101 11 0
r	0000 0	d o	1111 11 0

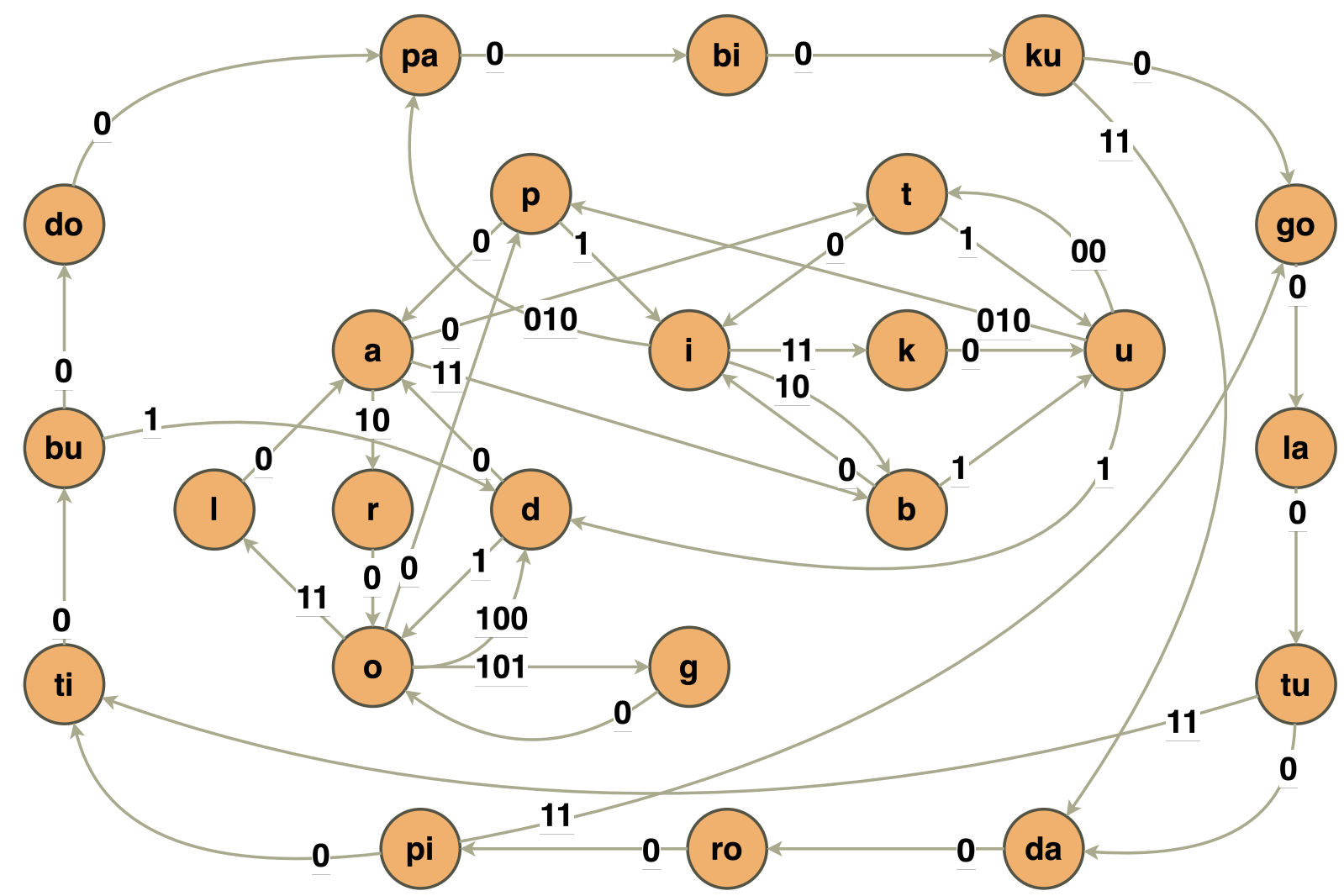

pa bi ku go la tu da ro pi ti bu
do pa bi ku da ro pi go la tu ti
bu d o g o l a t u p a b i k u t i
b u d o d a r o p i

→

0 0 0 0 0 0 0 0 0 0 0 0 0 0 11 0
0 11 0 0 11 0 1 1 101 0 11 0 0
1 010 0 11 0 11 0 00 0 10 1 1
1 100 0 10 0 0 1 010

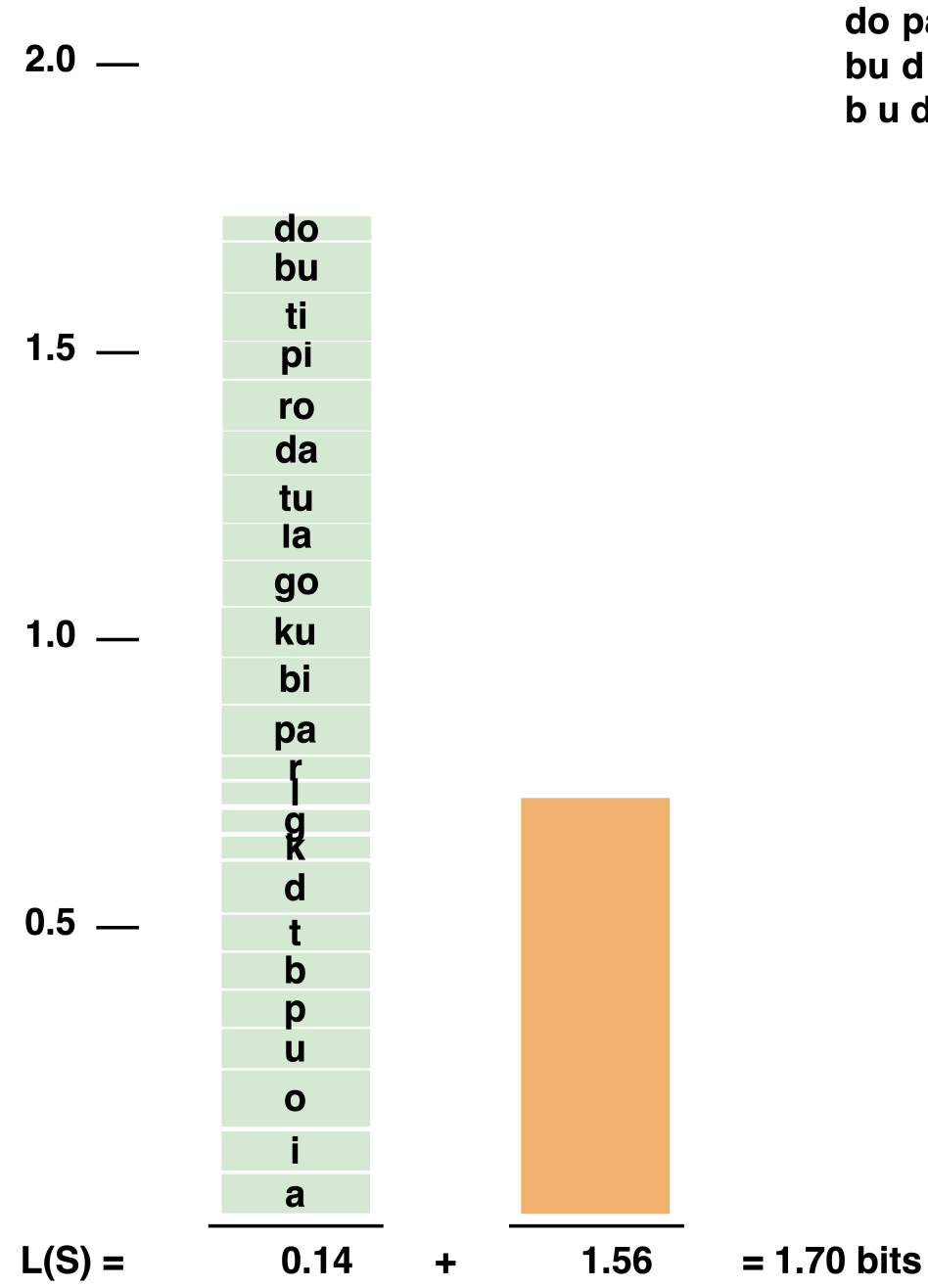

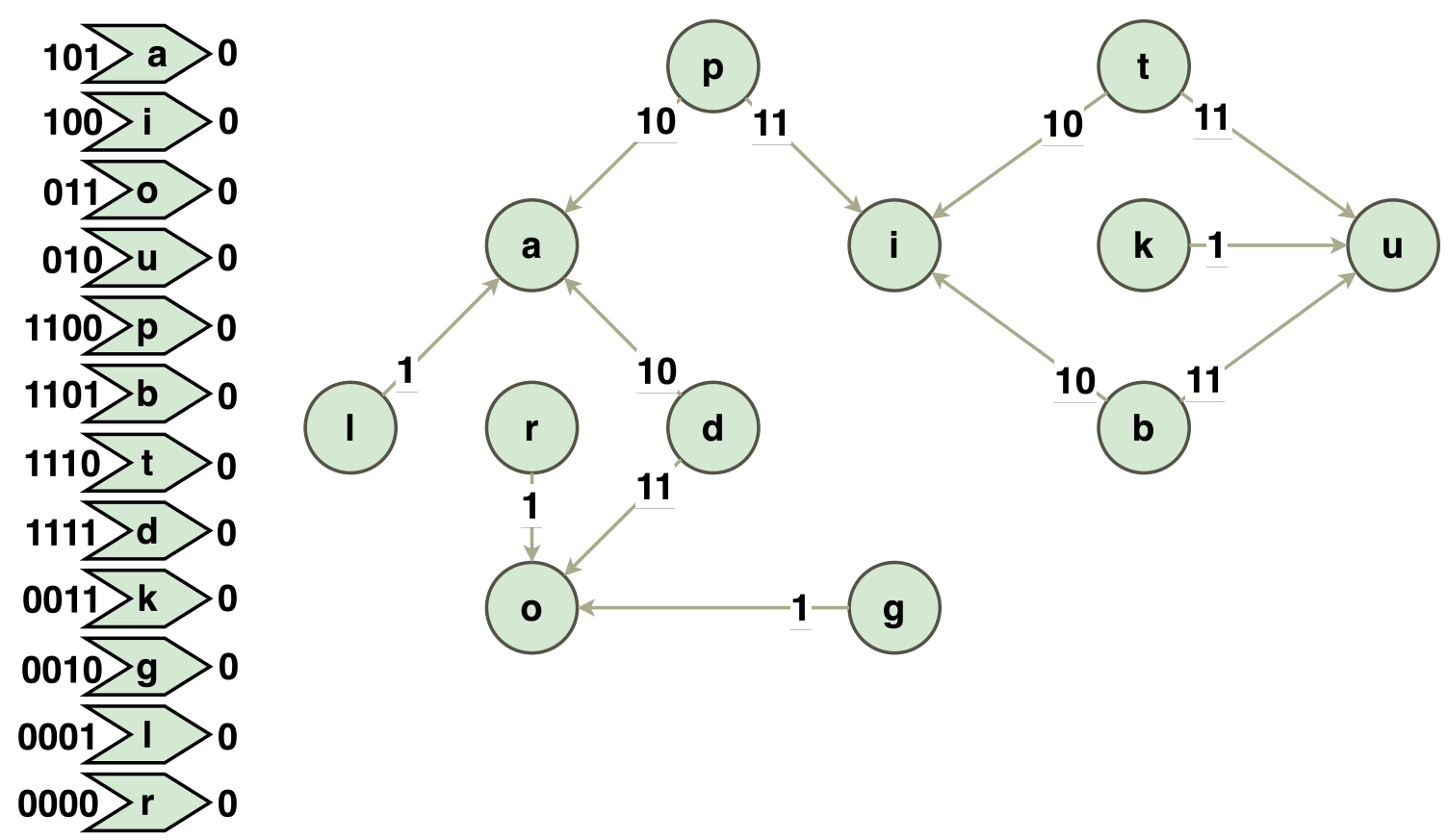

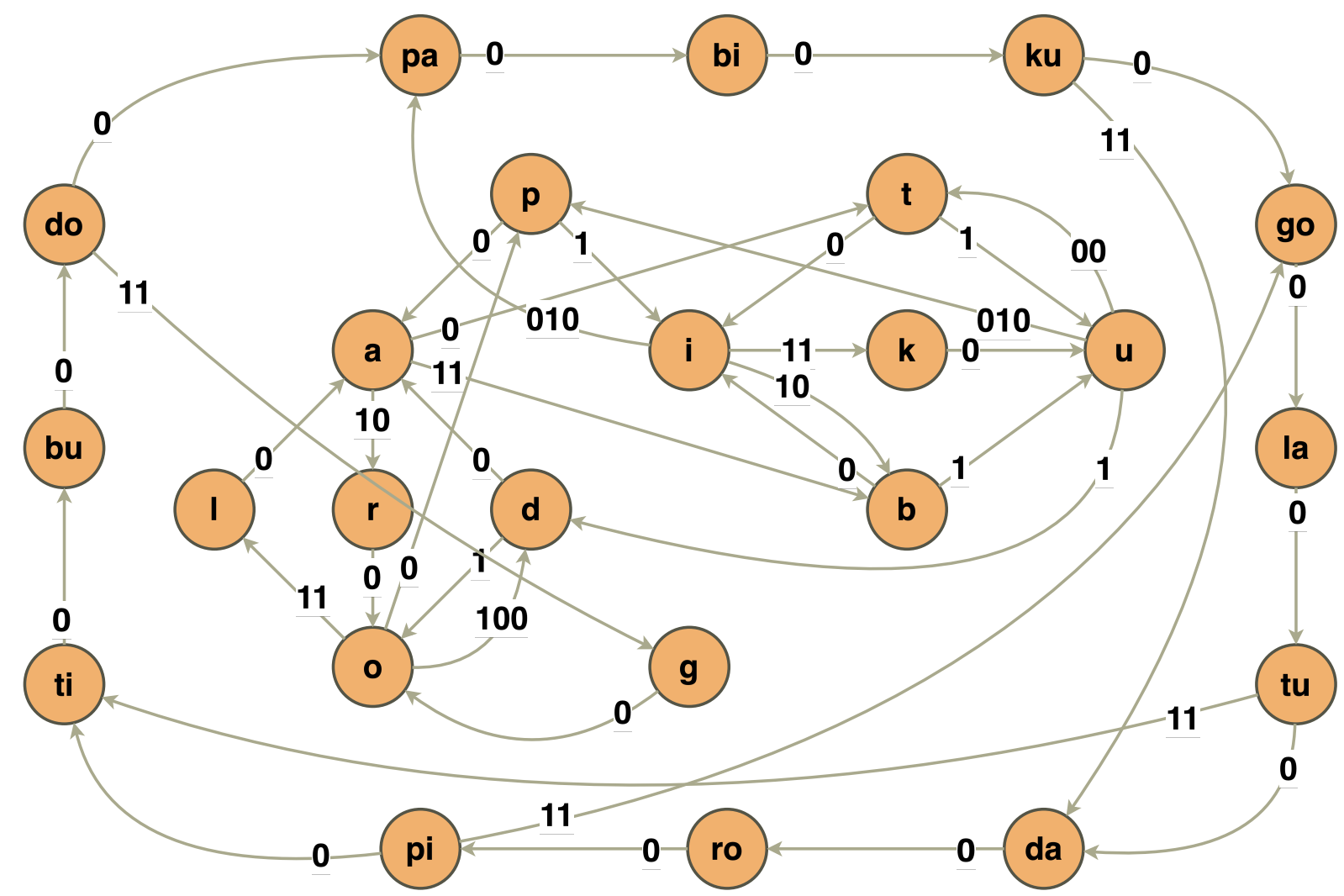

a	101 0	p a	1100 10 0
i	100 0	b i	1101 10 0
o	011 0	k u	0011 1 0
u	010 0	go	0010 1 0
p	1100 0	l a	0001 1 0
b	1101 0	t u	1110 11 0
t	1110 0	d a	1111 10 0
d	1111 0	r o	0000 1 0
k	0011 0	p i	1100 11 0
g	0010 0	t i	1110 10 0
l	0001 0	b u	1101 11 0
r	0000 0	d o	1111 11 0

pa bi ku go la tu da ro pi ti bu
do pa bi ku da ro pi go la tu ti
bu do g o l a t u p a b i k u t i b
u d o d a r o p i

→

0 0 0 0 0 0 0 0 0 0 0 0 0 0 0 11 0
0 11 0 0 11 0 0 11 0 11 0 0
1 010 0 11 0 11 0 00 0 10 1 1
1 100 0 10 0 0 1 010

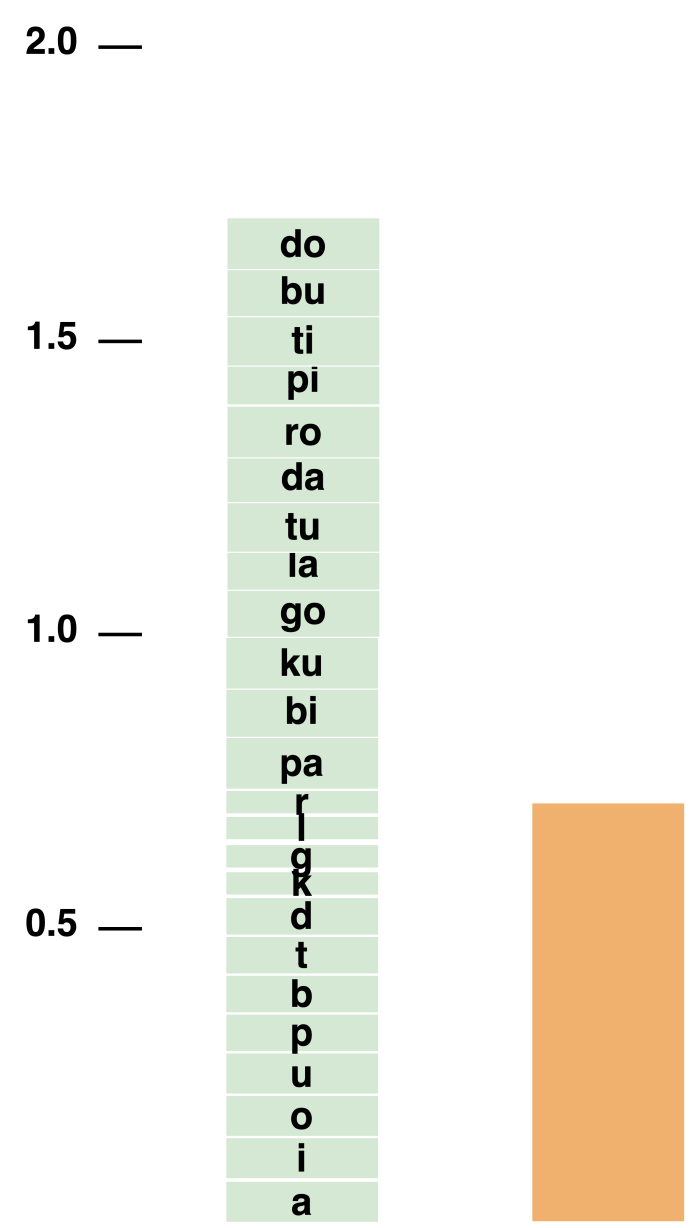

L(S) = 0.14 + 1.50 = 1.64 bits

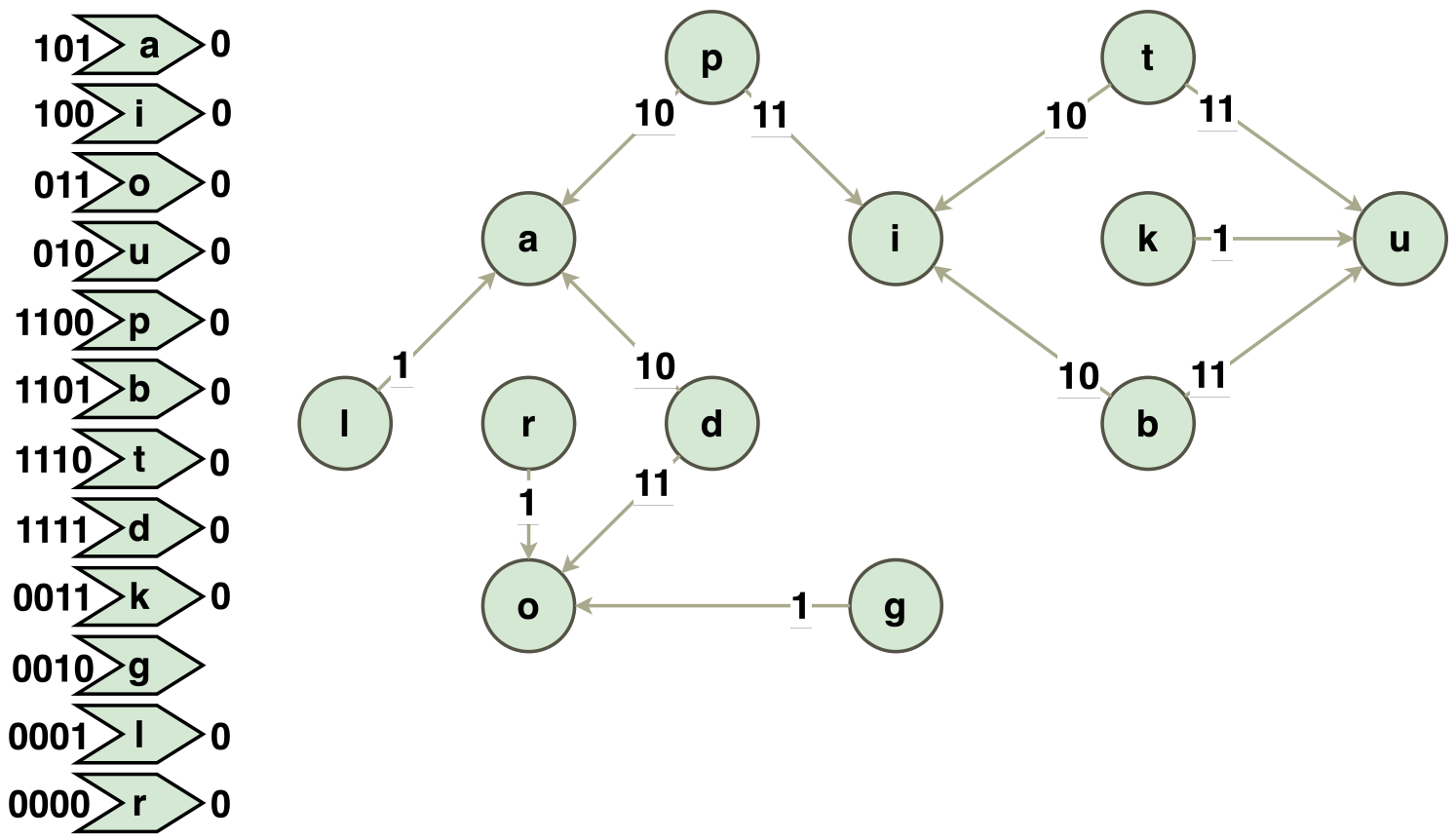

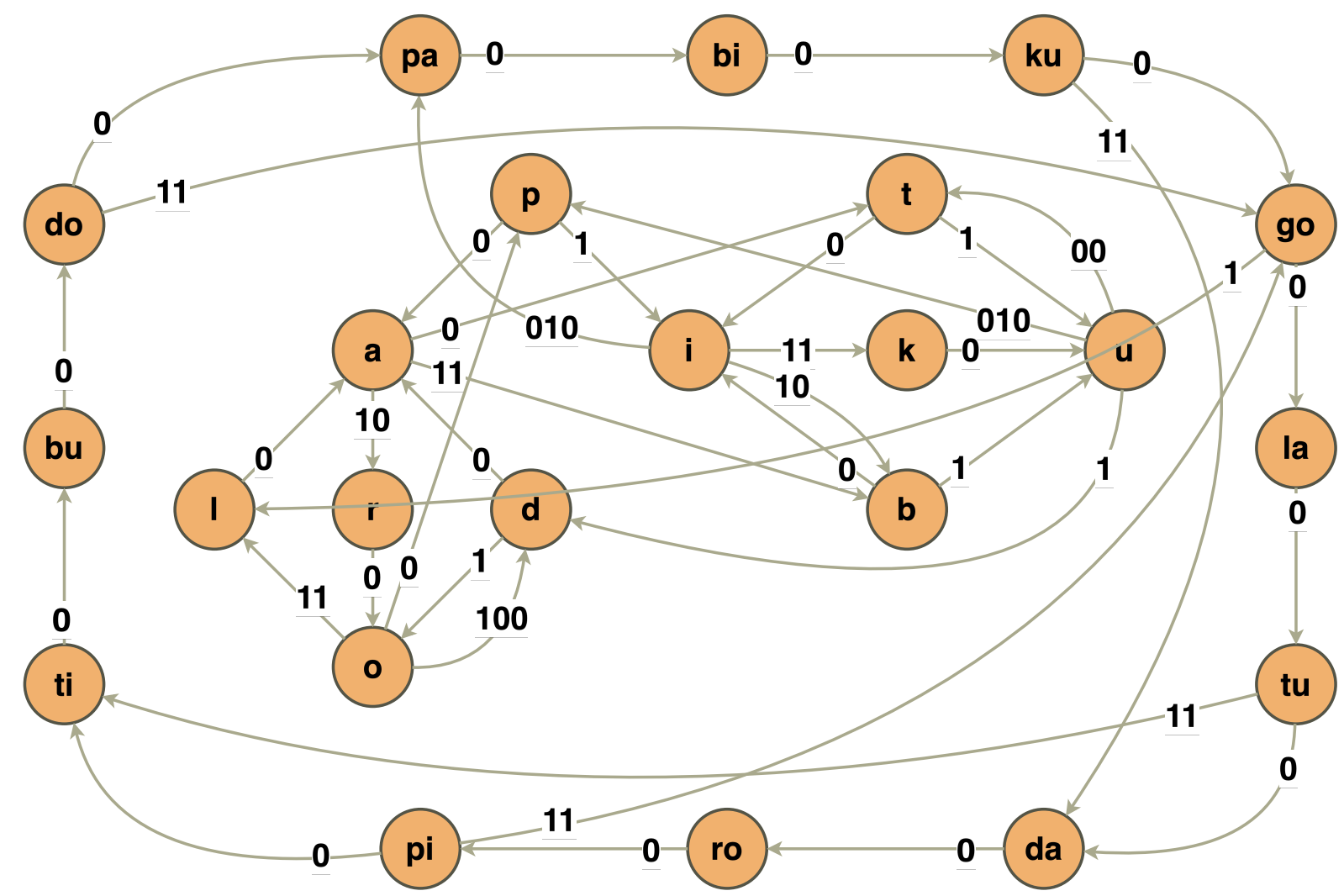

a	→	101 0
i	→	100 0
o	→	011 0
u	→	010 0
p	→	1100 0
b	→	1101 0
t	→	1110 0
d	→	1111 0
k	→	0011 0
l	→	0001 0
r	→	0000 0

p a	→	1100 10 0
b i	→	1101 10 0
k u	→	0011 1 0
go	→	0010 1 0
l a	→	0001 1 0
t u	→	1110 11 0
d a	→	1111 10 0
r o	→	0000 1 0
p i	→	1100 11 0
t i	→	1110 10 0
b u	→	1101 11 0
d o	→	1111 11 0

pa bi ku go la tu da ro pi ti bu
do pa bi ku da ro pi go la tu ti
bu do go l a t u p a b i k u t i b
u d o d a r o p i

→

0 0 0 0 0 0 0 0 0 0 0 0 0 0 11 0
0 11 0 0 11 0 0 11 1 0 0 1 010 0
11 0 11 0 00 0 10 1 1 1 100 0 10
0 0 1 010

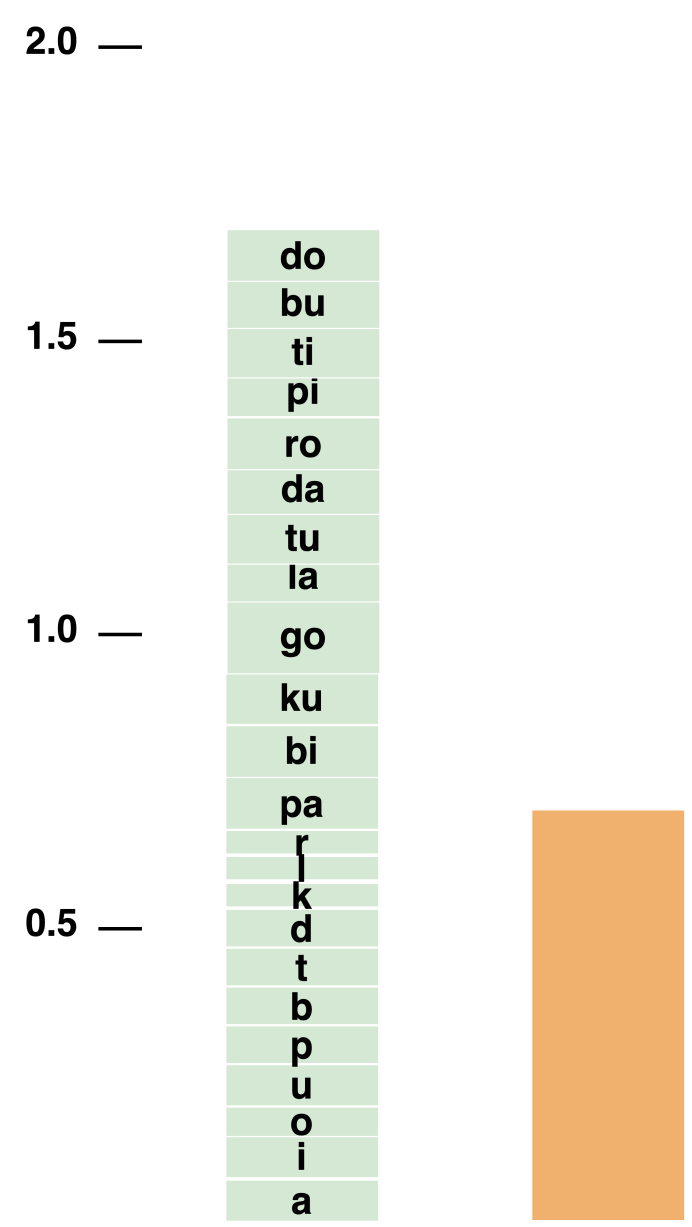

L(S) = 0.14 + 1.49 = 1.63 bits

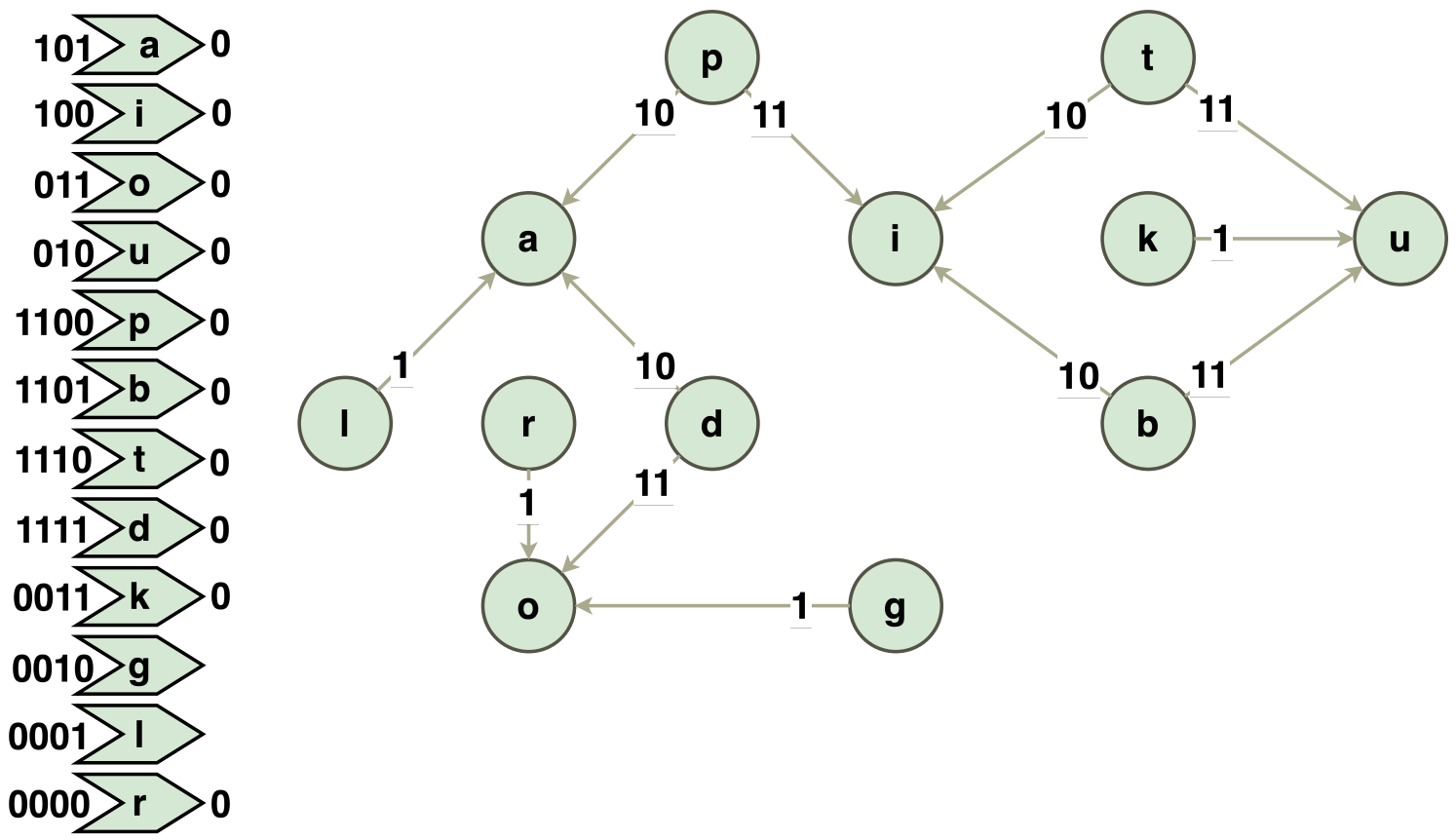

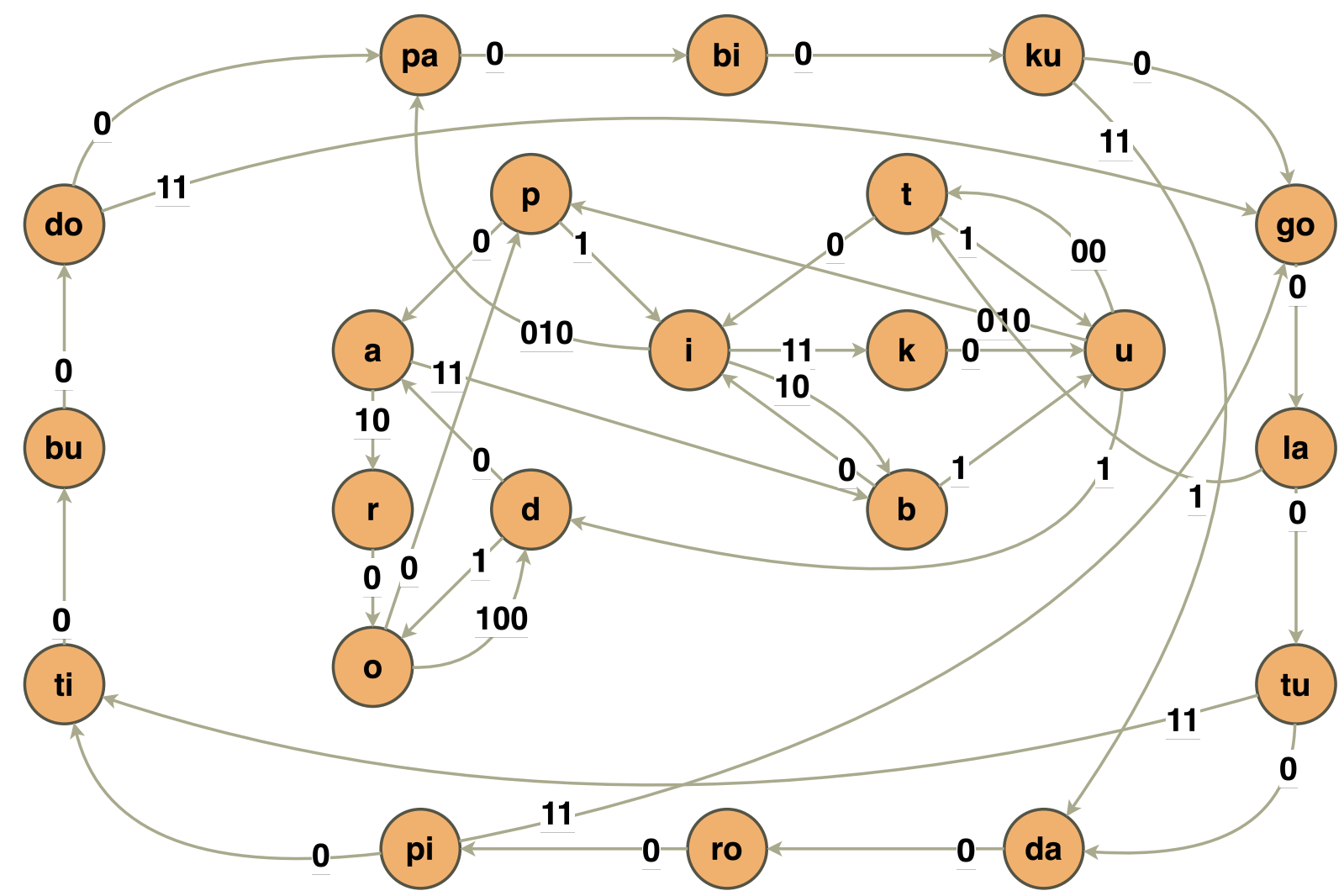

a	→	101 0
i	→	100 0
o	→	011 0
u	→	010 0
p	→	1100 0
b	→	1101 0
t	→	1110 0
d	→	1111 0
k	→	0011 0
r	→	0000 0

p a	→	1100 10 0
b i	→	1101 10 0
k u	→	0011 1 0
go	→	0010 1 0
l a	→	0001 1 0
t u	→	1110 11 0
d a	→	1111 10 0
r o	→	0000 1 0
p i	→	1100 11 0
t i	→	1110 10 0
b u	→	1101 11 0
d o	→	1111 11 0

pa bi ku go la tu da ro pi ti bu
do pa bi ku da ro pi go la tu ti
bu do go la t u p a b i k u t i b
u d o d a r o p i

→

0 0 0 0 0 0 0 0 0 0 0 0 0 0 11 0
0 11 0 0 11 0 0 11 0 1 1 010 0 11
0 11 0 00 0 10 1 1 1 100 0 10 0 0
1 010

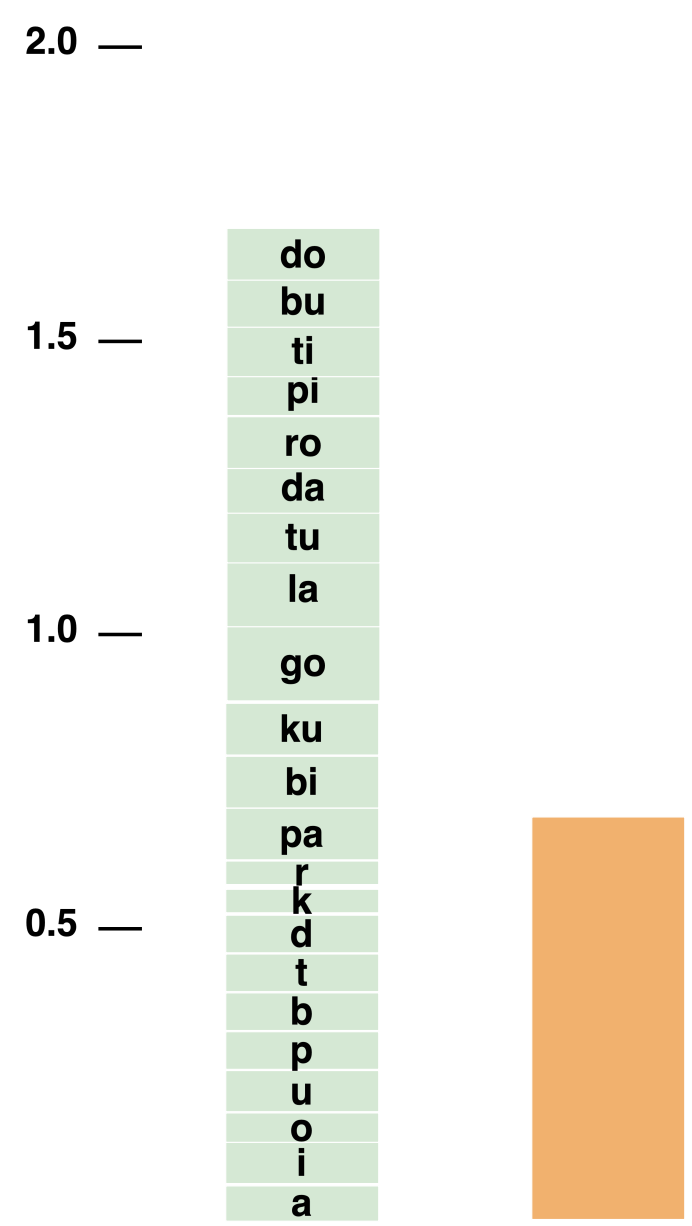

L(S) = 0.14 + 1.44 = 1.58 bits

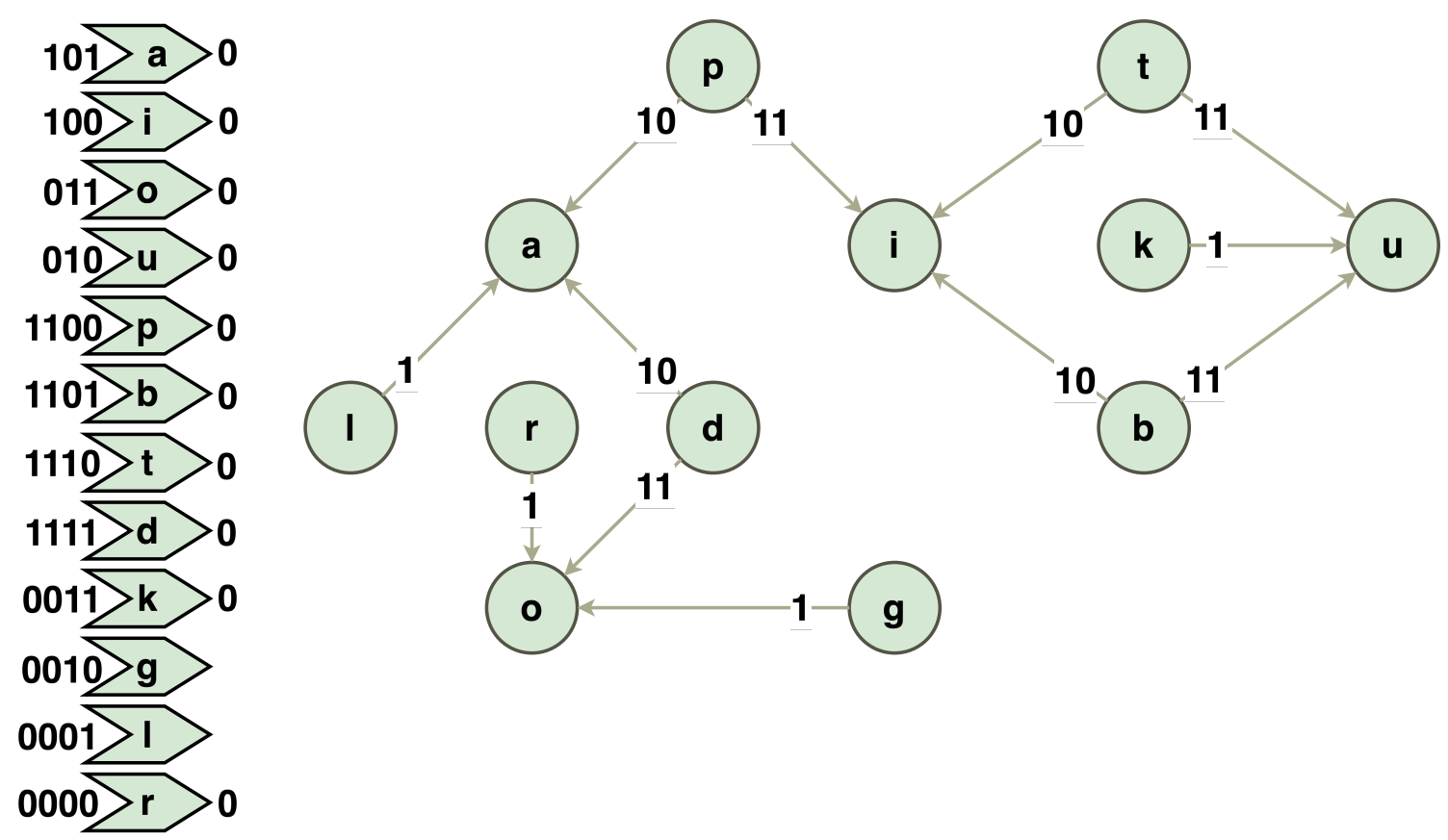

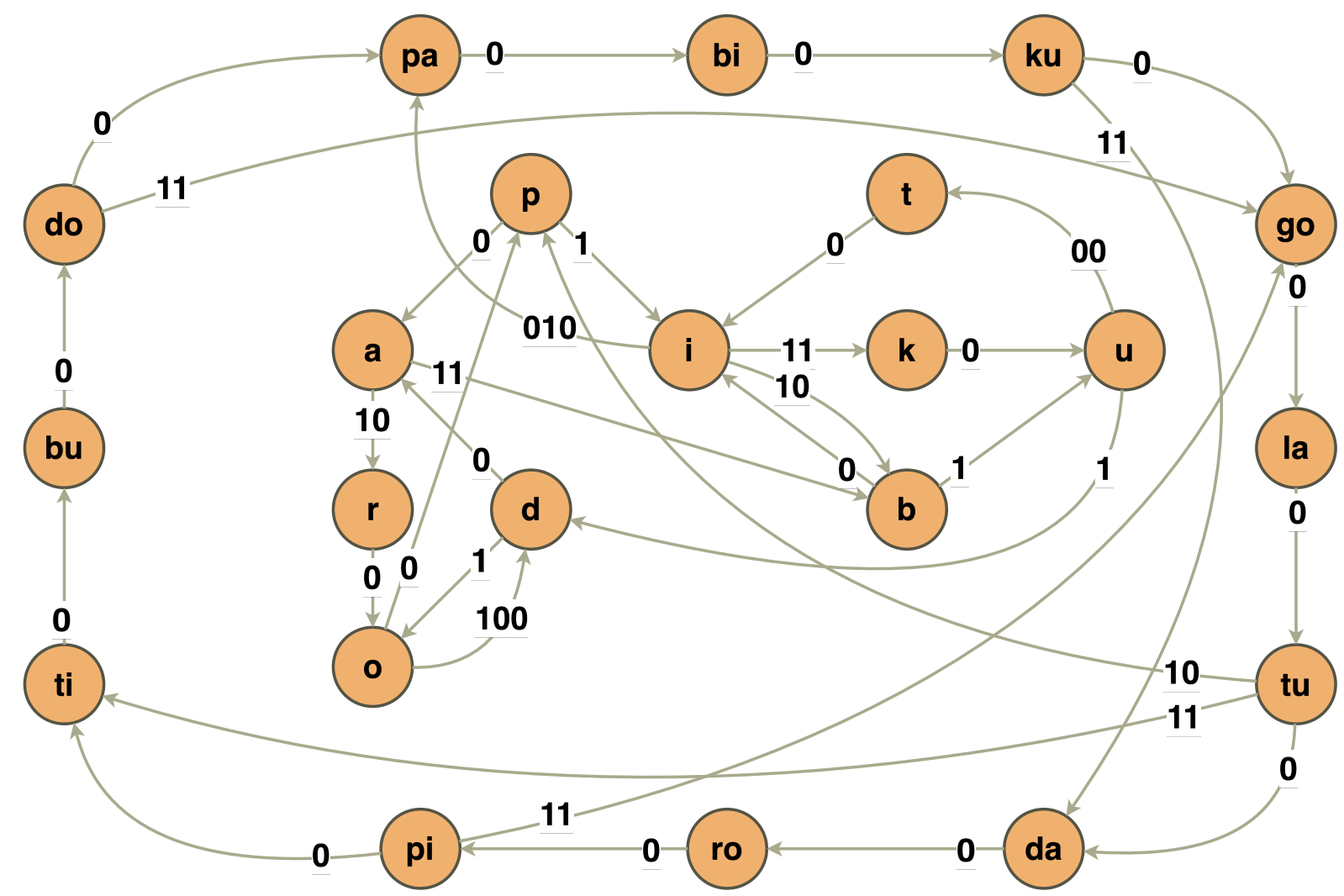

a	101 0	p a	1100 10 0
i	100 0	b i	1101 10 0
o	011 0	k u	0011 1 0
u	010 0	go	0010 1 0
p	1100 0	l a	0001 1 0
b	1101 0	t u	1110 11 0
t	1110 0	d a	1111 10 0
d	1111 0	r o	0000 1 0
k	0011 0	p i	1100 11 0
		t i	1110 10 0
		b u	1101 11 0
r	0000 0	d o	1111 11 0

pa bi ku go la tu da ro pi ti bu do pa bi ku da ro pi go la tu ti bu do go la tu p a b i k u t i b u d o d a r o p i → 0 0 0 0 0 0 0 0 0 0 0 0 0 0 11 0 0 11 0 0 11 0 0 11 0 0 11 0 0 10 0 11 0 11 0 00 0 10 1 1 1 100 0 10 0 0 1 010

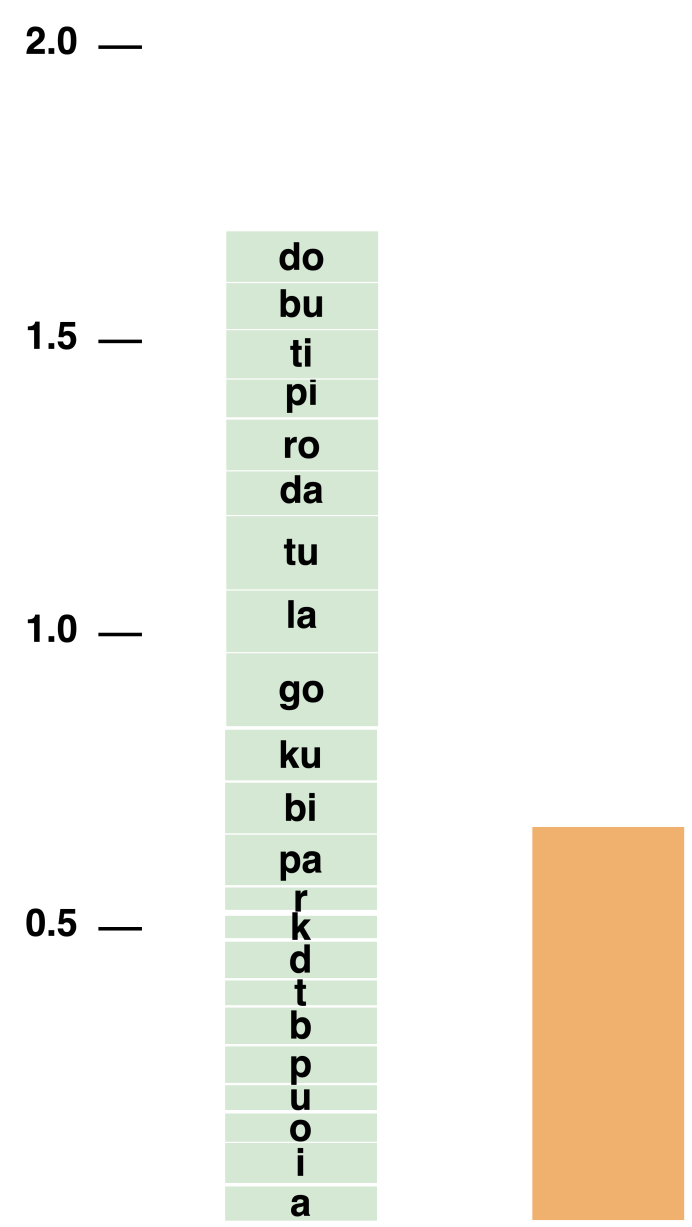

L(S) = 0.15 + 1.37 = 1.52 bits

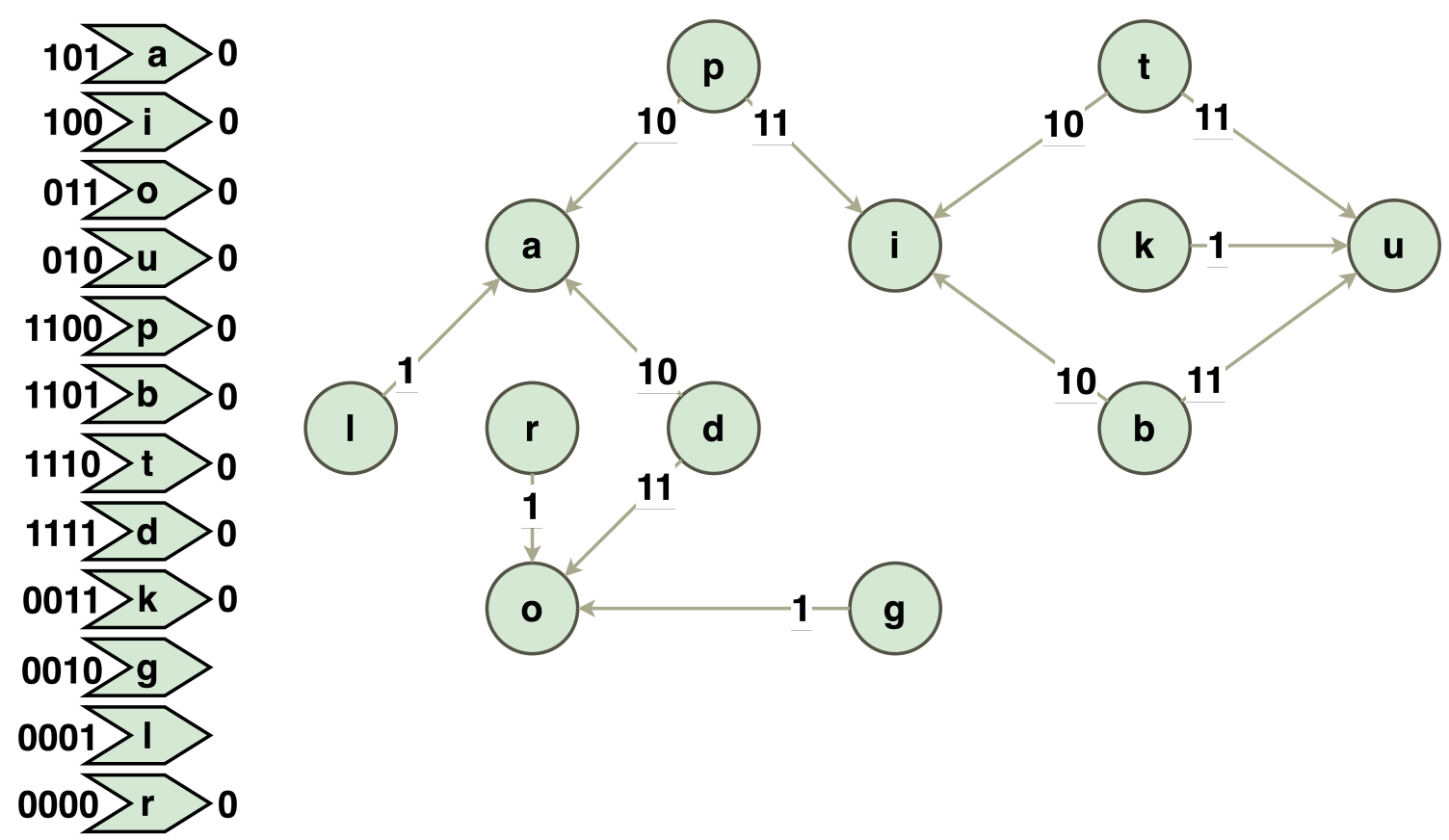

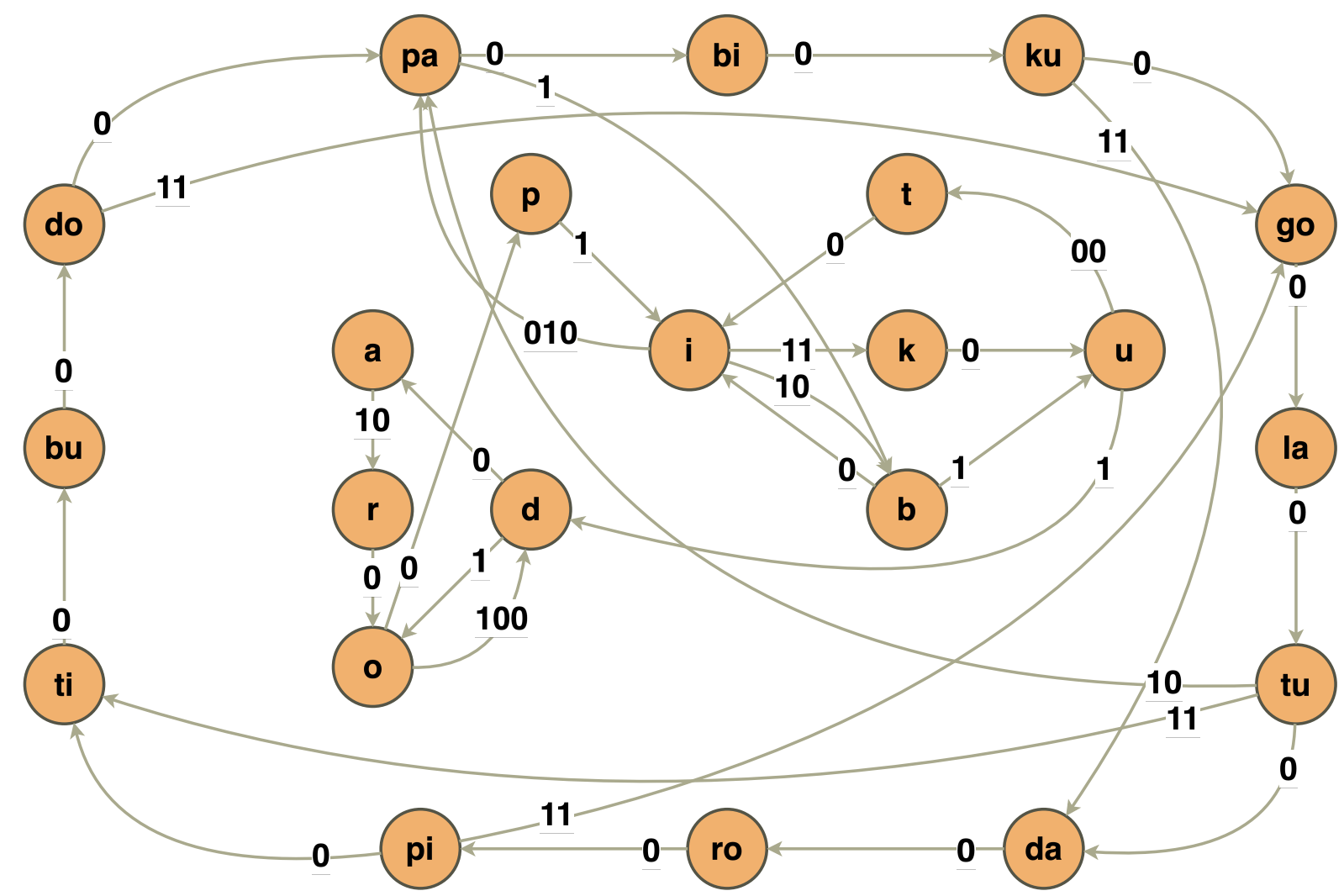

a → 101 0		p a → 1100 10 0	
i → 100 0		b i → 1101 10 0	
o → 011 0		k u → 0011 1 0	
u → 010 0		go → 0010 1 0	
p → 1100 0		l a → 0001 1 0	
b → 1101 0		t u → 1110 11 0	
t → 1110 0		d a → 1111 10 0	
d → 1111 0		r o → 0000 1 0	
k → 0011 0		p i → 1100 11 0	
		t i → 1110 10 0	
		b u → 1101 11 0	
r → 0000 0		d o → 1111 11 0	

pa bi ku go la tu da ro pi ti bu
do pa bi ku da ro pi go la tu ti
bu do go la tu pa b i k u t i b u
d o d a r o p i

→

0 0 0 0 0 0 0 0 0 0 0 0 0 0 11 0
0 11 0 0 11 0 0 11 0 0 10 1 0 11
0 00 0 10 1 1 1 100 0 10 0 0
1 010

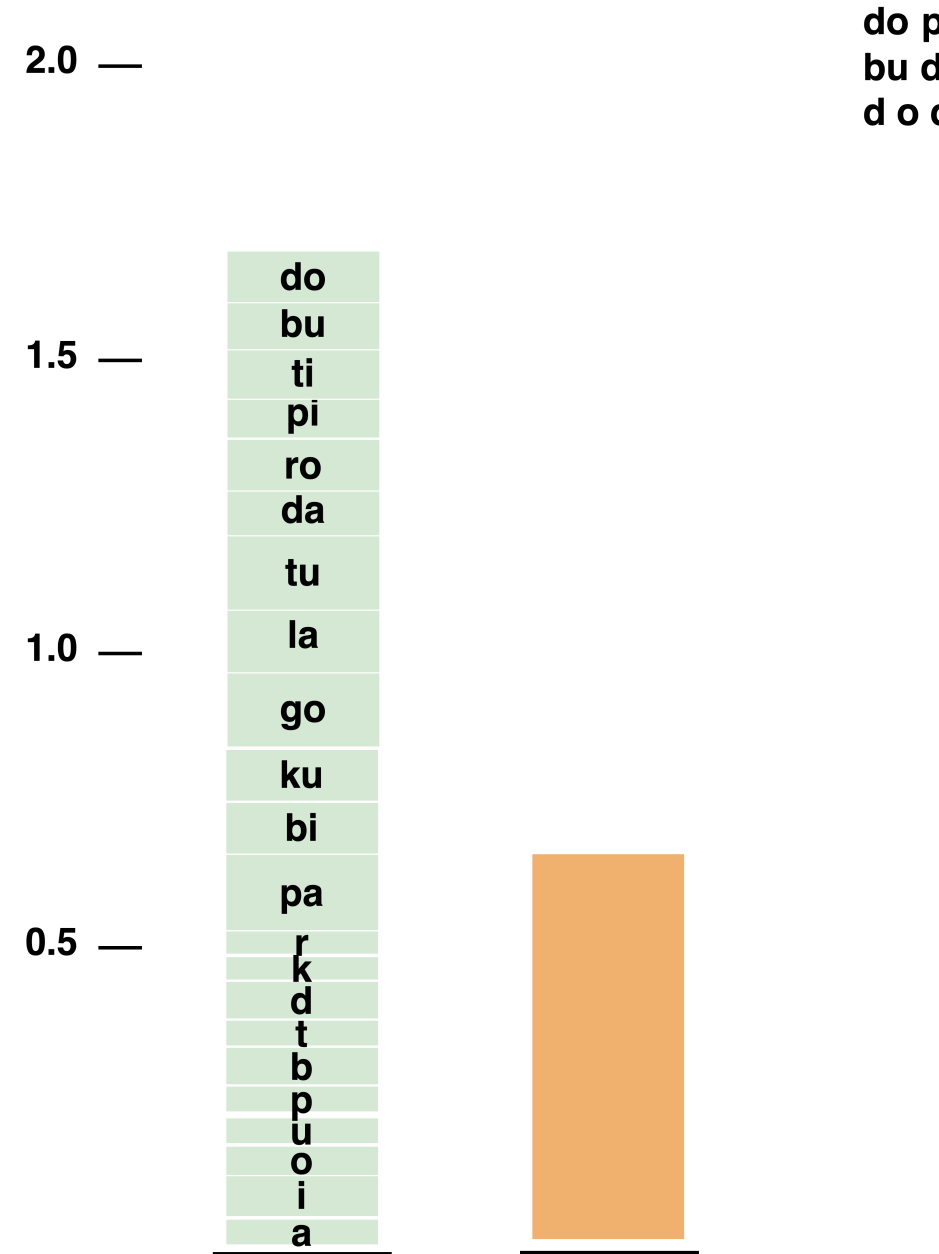

L(S) = 0.15 + 1.34 = 1.49 bits

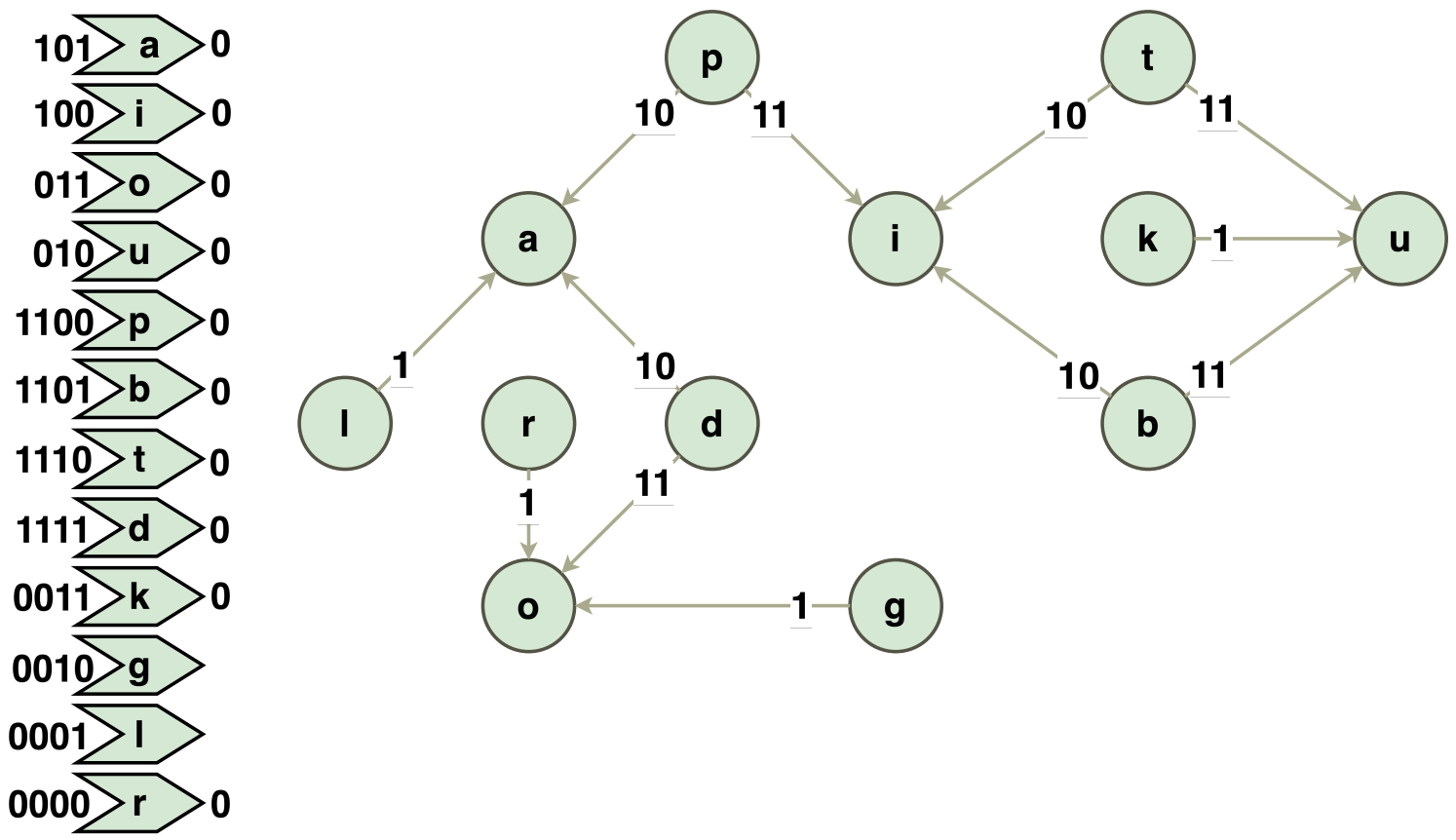

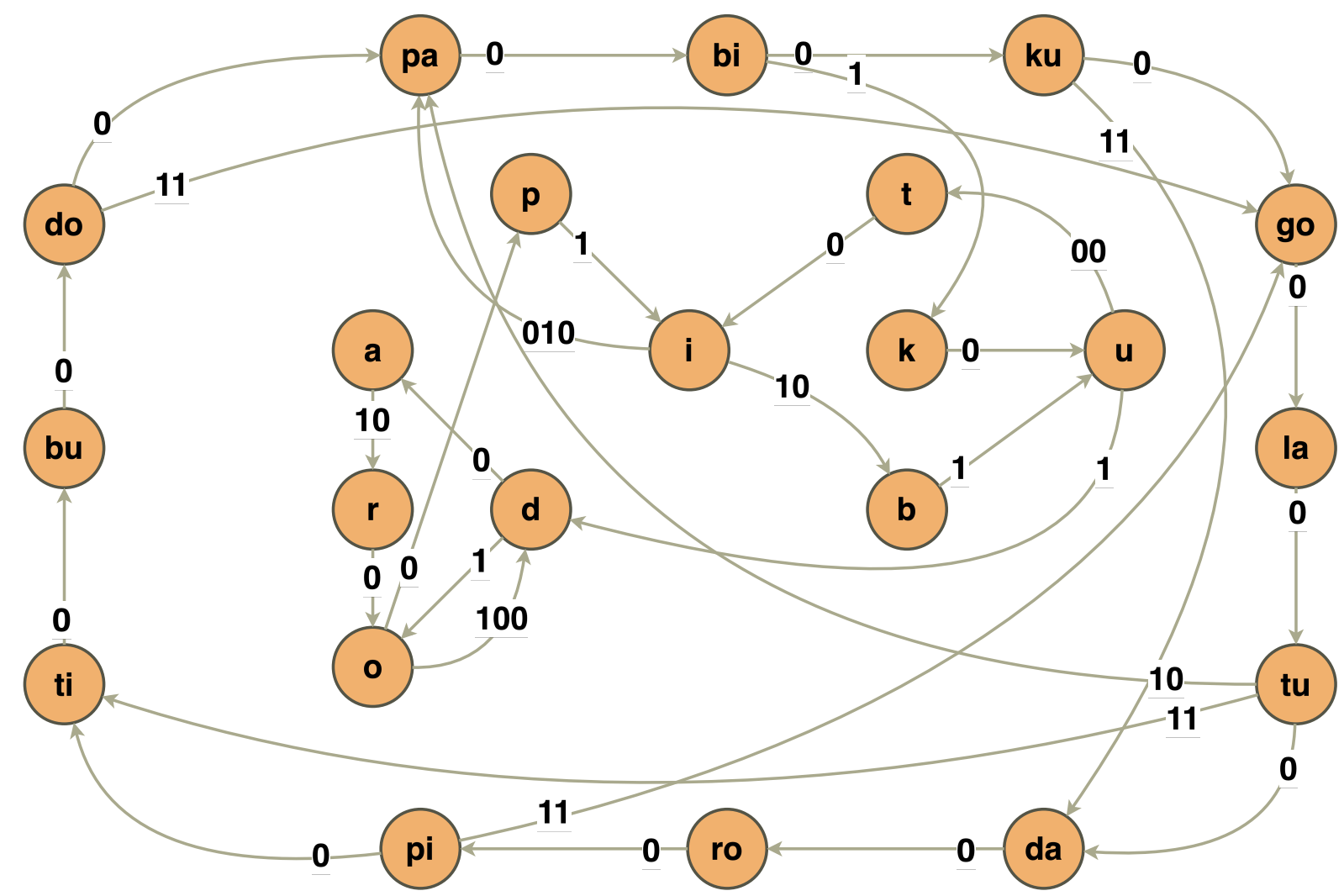

a	101 0		p a	1100 10 0
i	100 0		b i	1101 10 0
o	011 0		k u	0011 1 0
u	010 0		go	0010 1 0
p	1100 0		l a	0001 1 0
b	1101 0		t u	1110 11 0
t	1110 0		d a	1111 10 0
d	1111 0		r o	0000 1 0
k	0011 0		p i	1100 11 0
			t i	1110 10 0
			b u	1101 11 0
r	0000 0		d o	1111 11 0

pa bi ku go la tu da ro pi ti bu
do pa bi ku da ro pi go la tu ti
bu do go la tu pa bi k u t i b u
d o d a r o p i

0 0 0 0 0 0 0 0 0 0 0 0 0 0 11 0
0 11 0 0 11 0 0 11 0 0 10 0 1
0 00 0 10 1 1 1 100 0 10 0 0
1 010

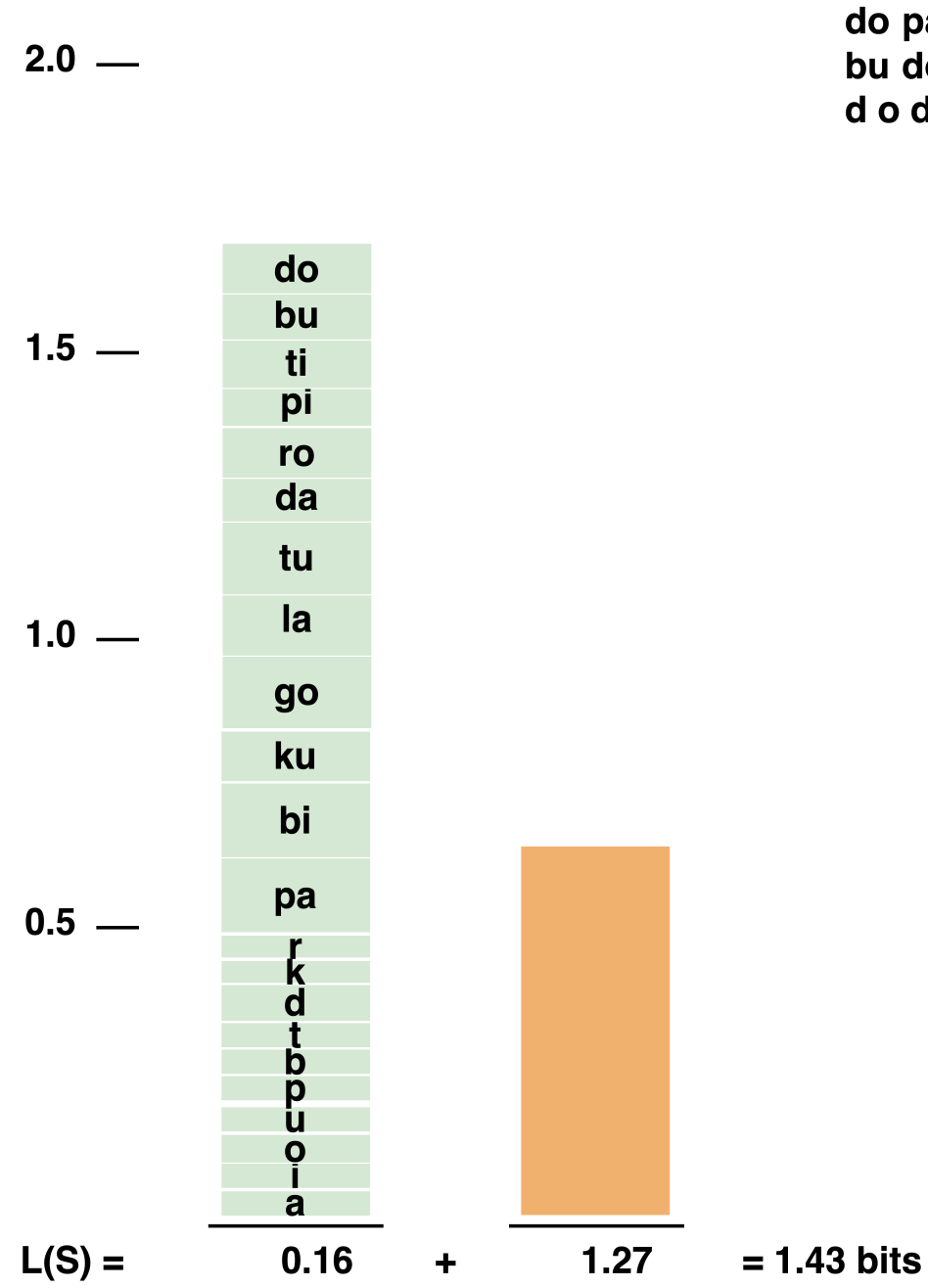

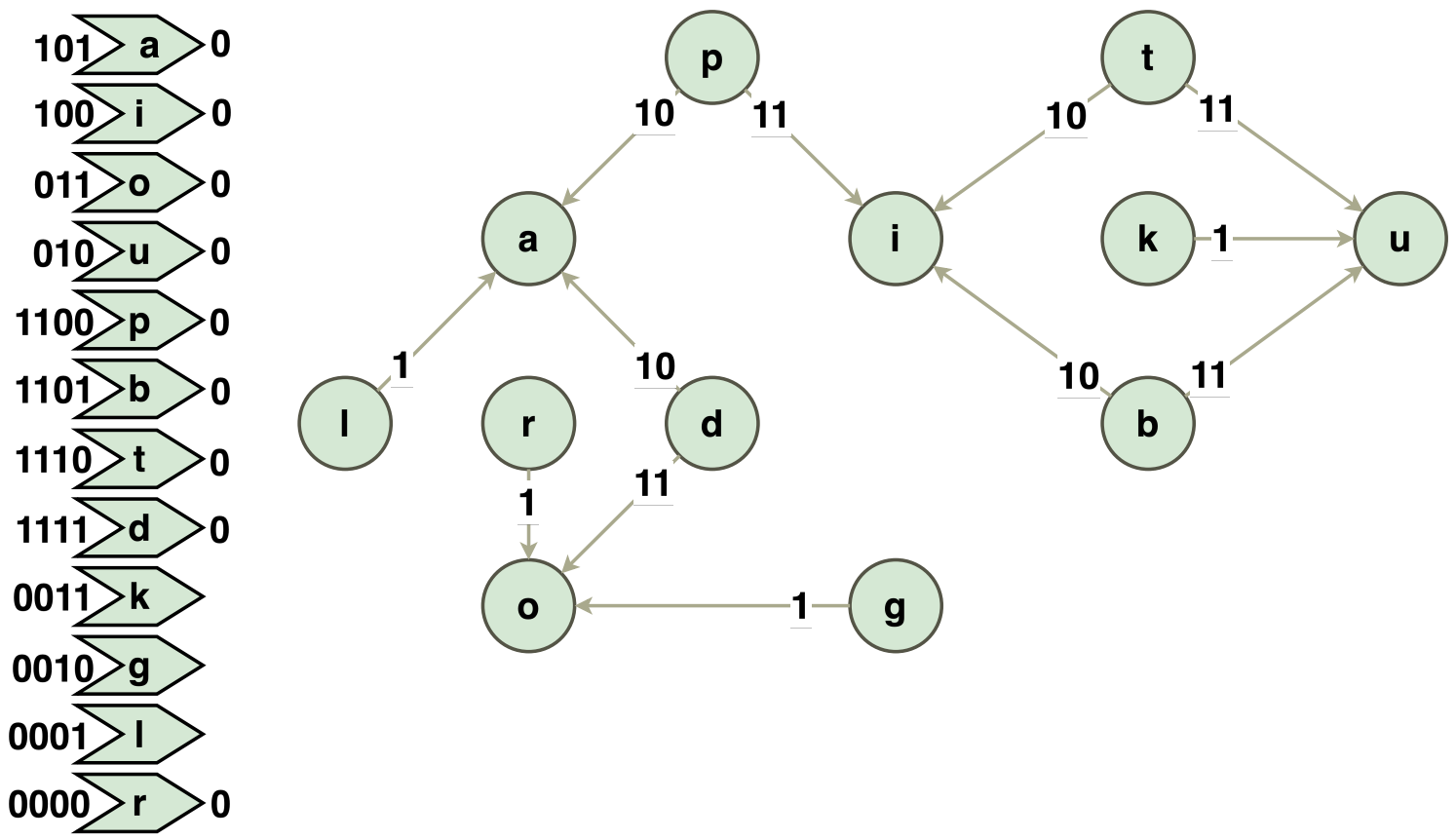

a →	101 0	p a →	1100 10 0
i →	100 0	b i →	1101 10 0
o →	011 0	k u →	0011 1 0
u →	010 0	go →	0010 1 0
p →	1100 0	l a →	0001 1 0
b →	1101 0	t u →	1110 11 0
t →	1110 0	d a →	1111 10 0
d →	1111 0	r o →	0000 1 0
		p i →	1100 11 0
		t i →	1110 10 0
		b u →	1101 11 0
r →	0000 0	d o →	1111 11 0

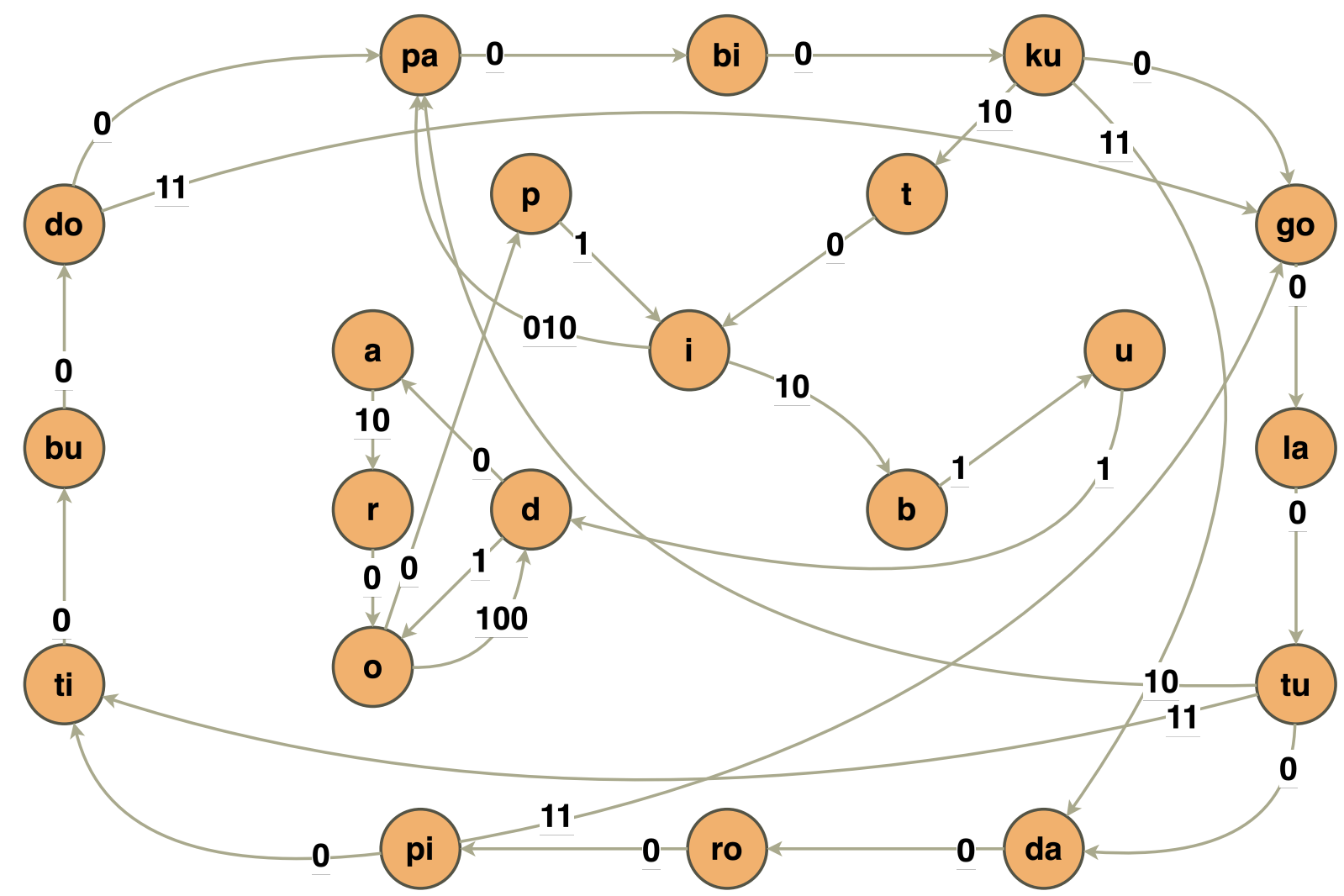

pa bi ku go la tu da ro pi ti bu do pa bi ku da ro pi go la tu ti bu do go la tu pa bi ku t i b u d o d a r o p i → 0 0 0 0 0 0 0 0 0 0 0 0 0 0 0 11 0 0 11 0 0 11 0 0 11 0 0 10 0 0 10 0 10 1 1 1 100 0 10 0 0 1 010

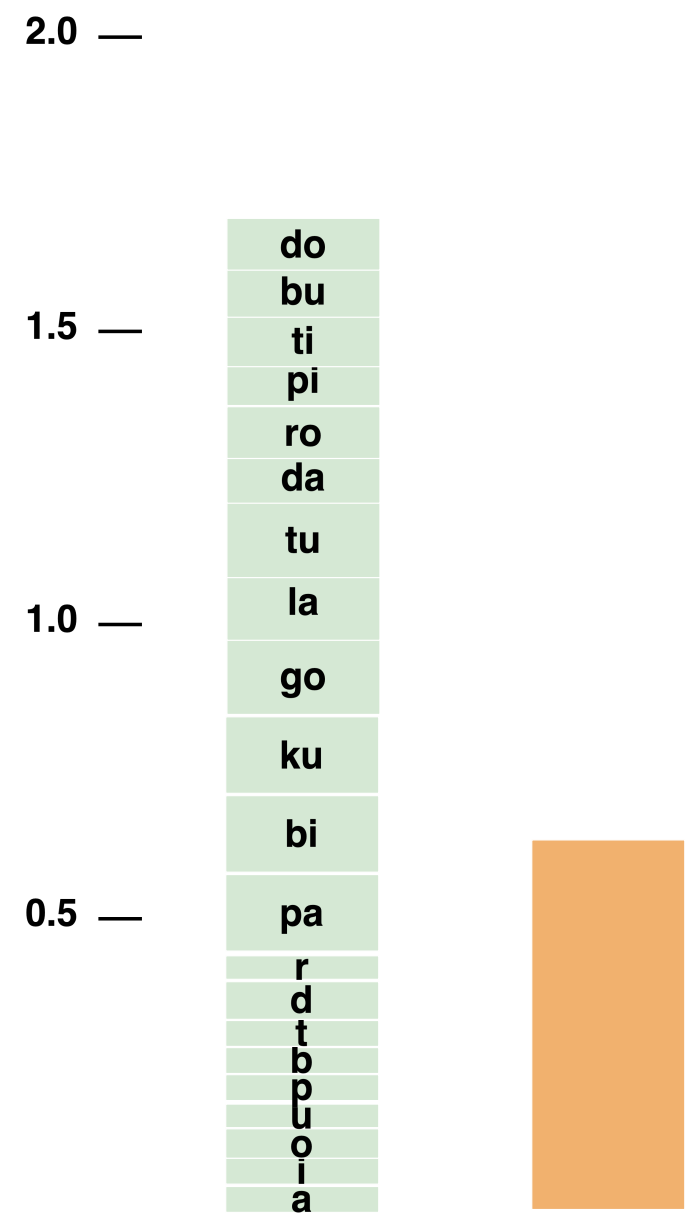

L(S) = 0.16 + 1.23 = 1.39 bits

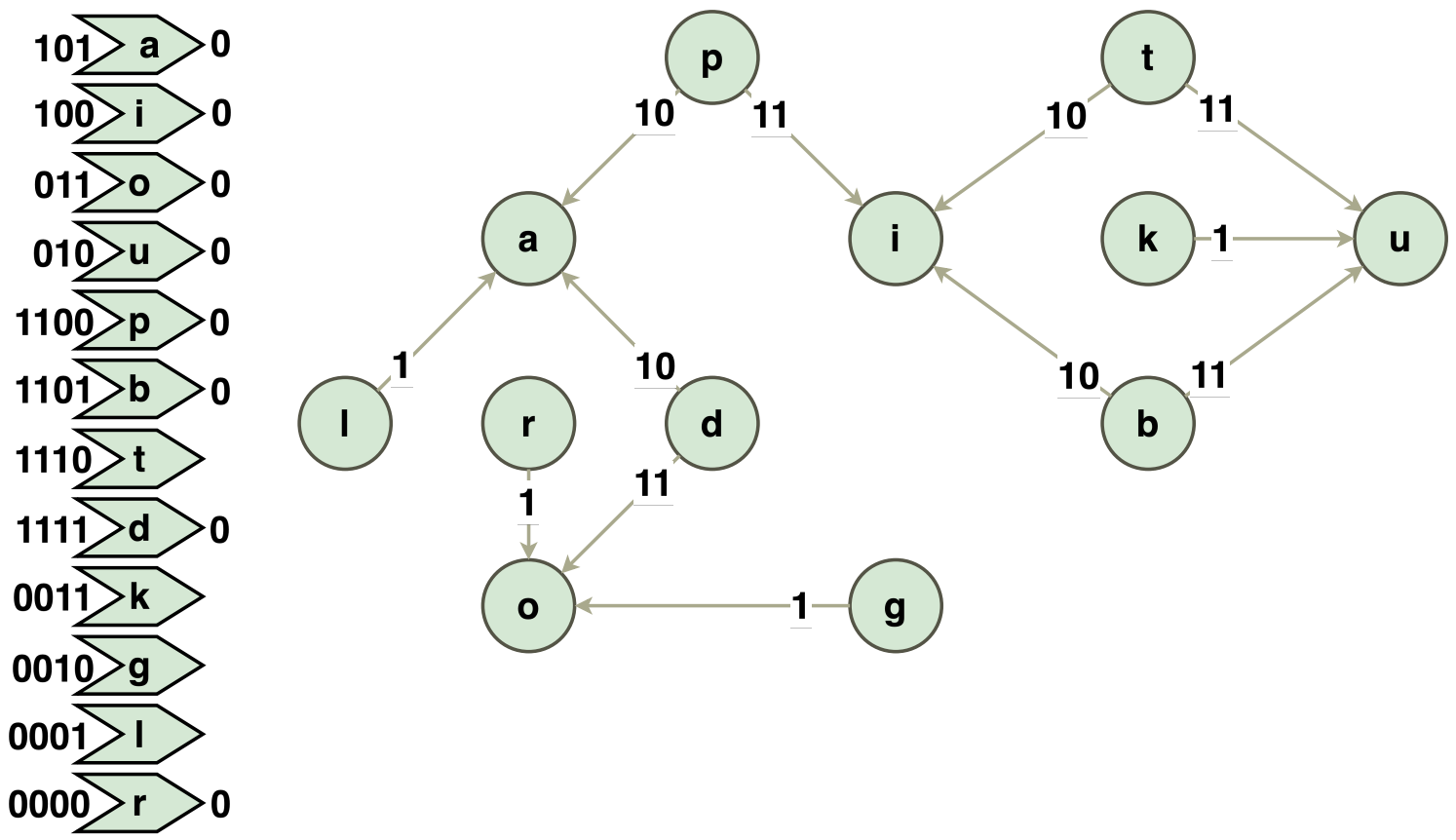

a	101 0	p a	1100 10 0
i	100 0	b i	1101 10 0
o	011 0	k u	0011 1 0
u	010 0	go	0010 1 0
p	1100 0	l a	0001 1 0
b	1101 0	t u	1110 11 0
		d a	1111 10 0
d	1111 0	r o	0000 1 0
		p i	1100 11 0
		t i	1110 10 0
		b u	1101 11 0
r	0000 0	d o	1111 11 0

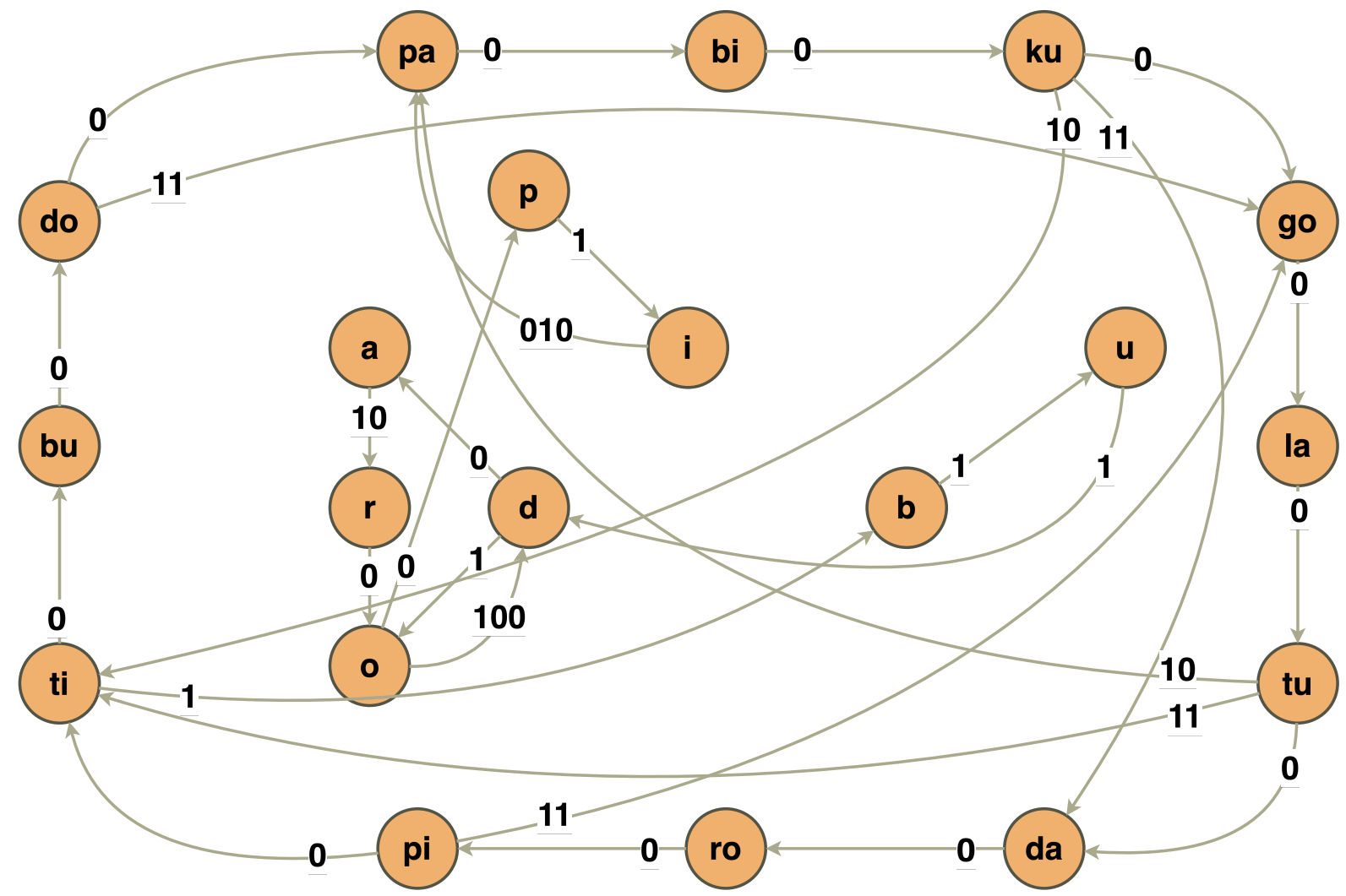

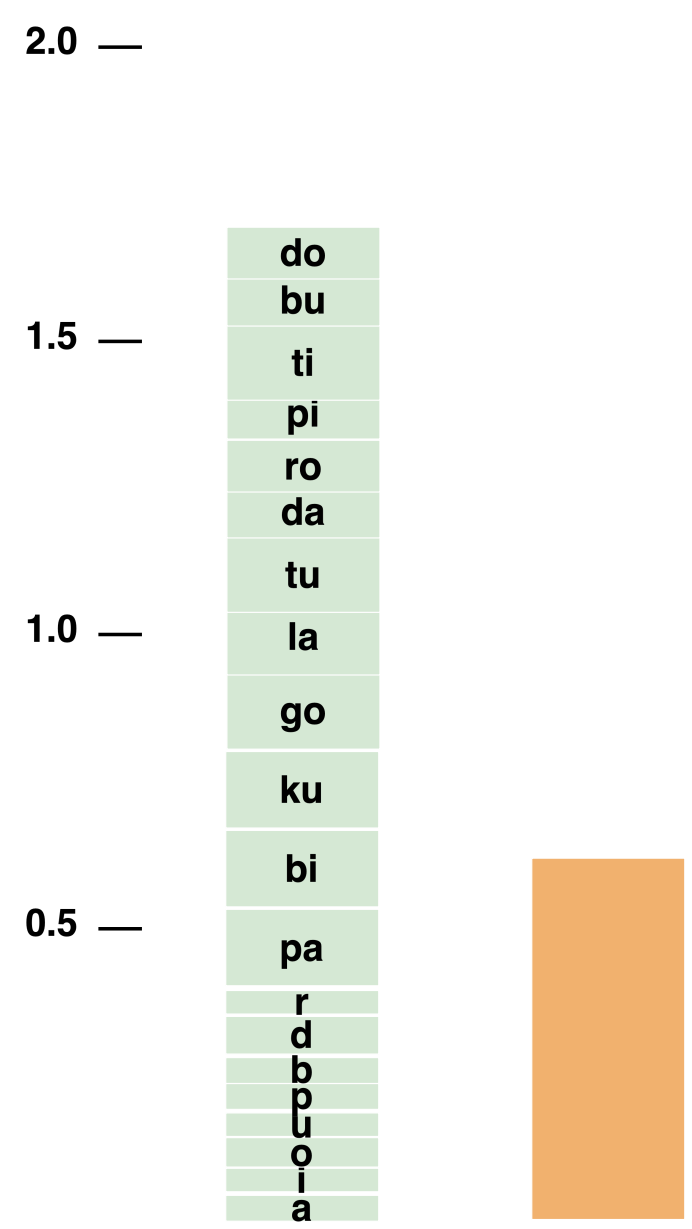

L(S) = 0.17 + 1.21 = 1.38 bits

pa bi ku go la tu da ro pi ti bu do pa bi ku da ro pi go la tu ti bu do go la tu pa bi ku ti b u d o d a r o p i → 0 0 0 0 0 0 0 0 0 0 0 0 0 0 0 11 0 0 11 0 0 11 0 0 11 0 0 10 0 0 10 1 1 1 1 100 0 10 0 0 1 010

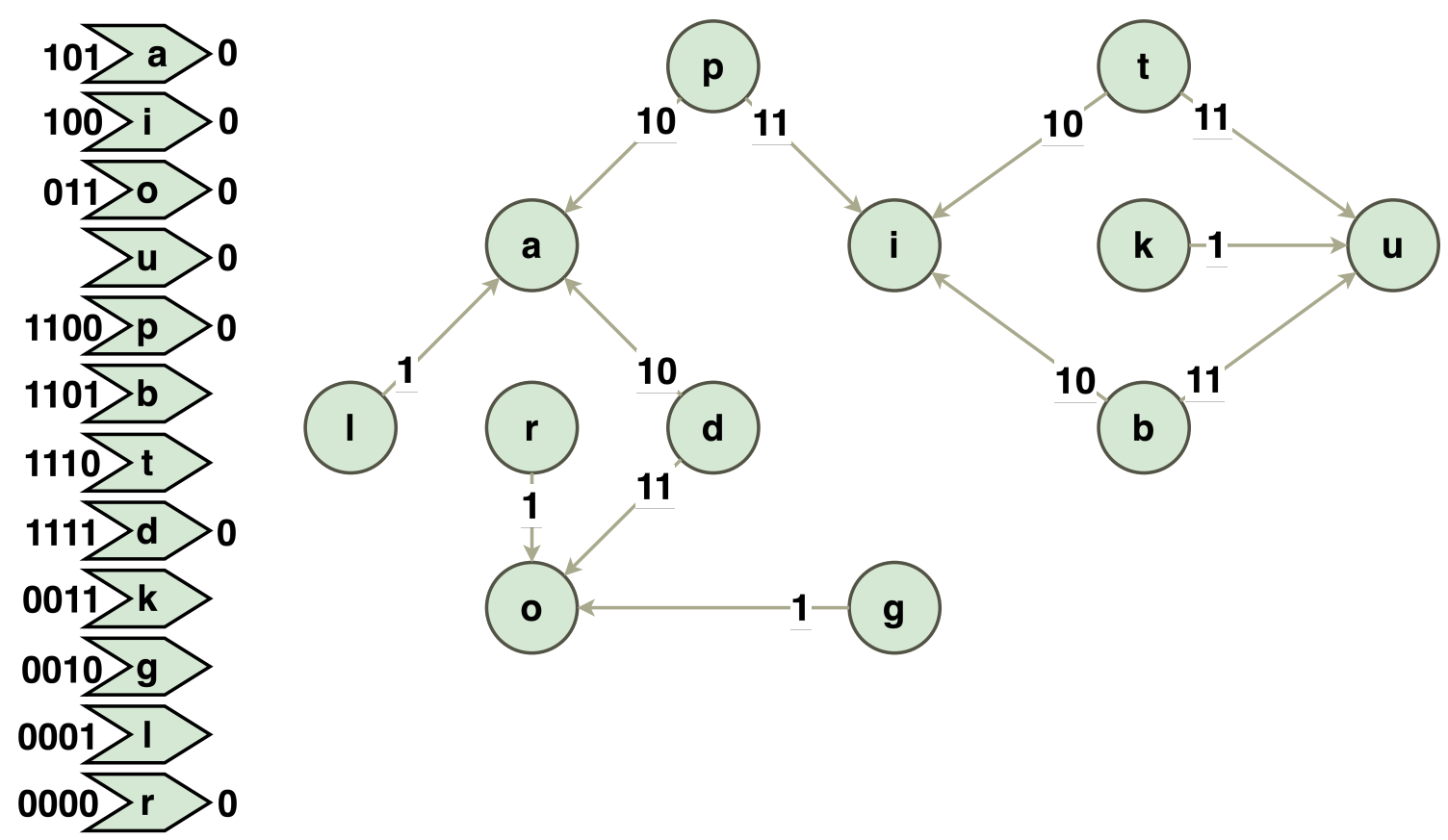

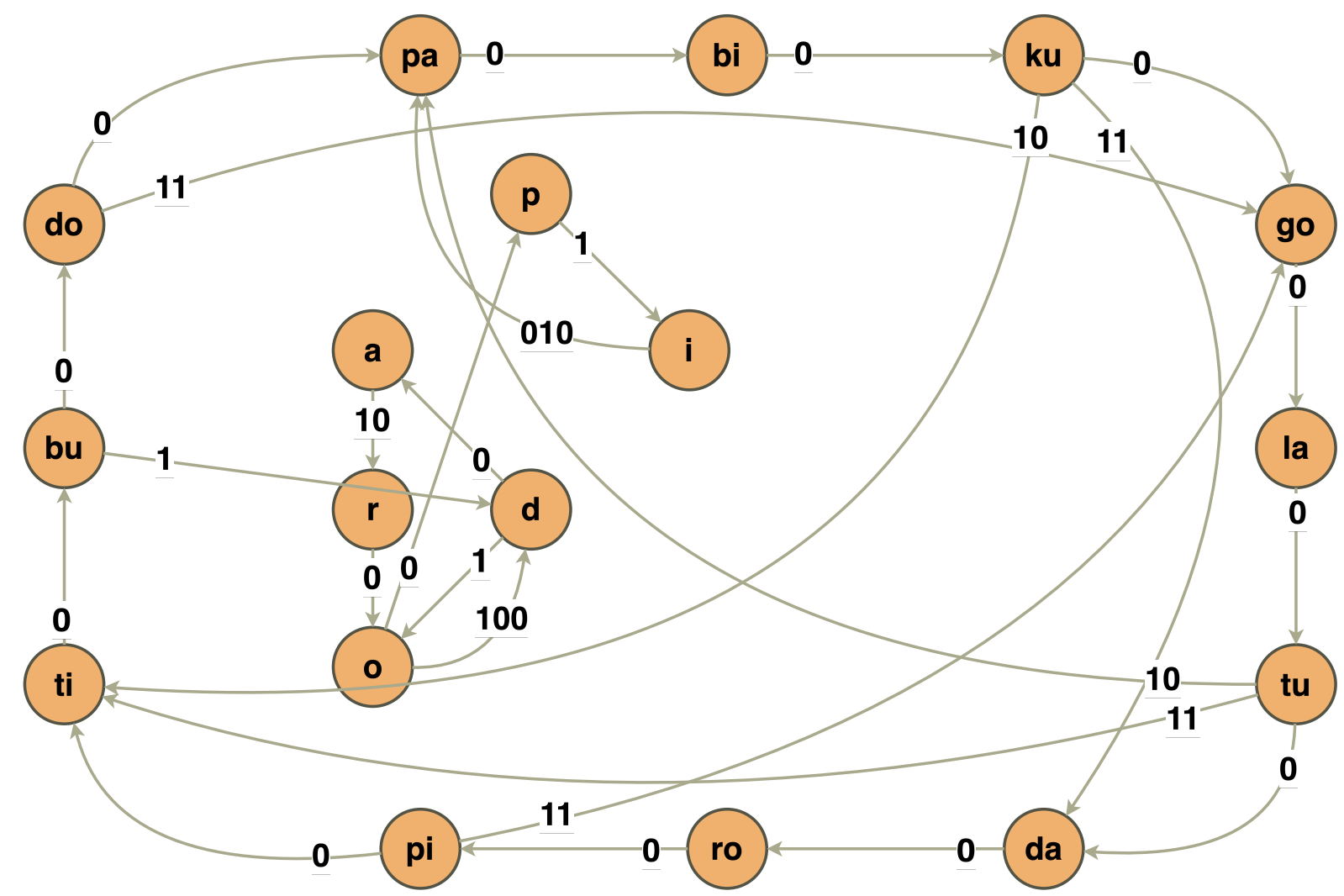

a	→	101 0
i	→	100 0
o	→	011 0
p	→	1100 0
d	→	1111 0
r	→	0000 0

p a	→	1100 10 0
b i	→	1101 10 0
k u	→	0011 1 0
go	→	0010 1 0
l a	→	0001 1 0
t u	→	1110 11 0
d a	→	1111 10 0
r o	→	0000 1 0
p i	→	1100 11 0
t i	→	1110 10 0
b u	→	1101 11 0
d o	→	1111 11 0

pa bi ku go la tu da ro pi ti bu do pa bi ku da ro pi go la tu ti bu do go la tu pa bi ku ti bu d o d a r o p i → 0 0 0 0 0 0 0 0 0 0 0 0 0 0 11 0 0 11 0 0 11 0 0 11 0 0 10 0 0 10 0 1 1 100 0 10 0 0 1 010

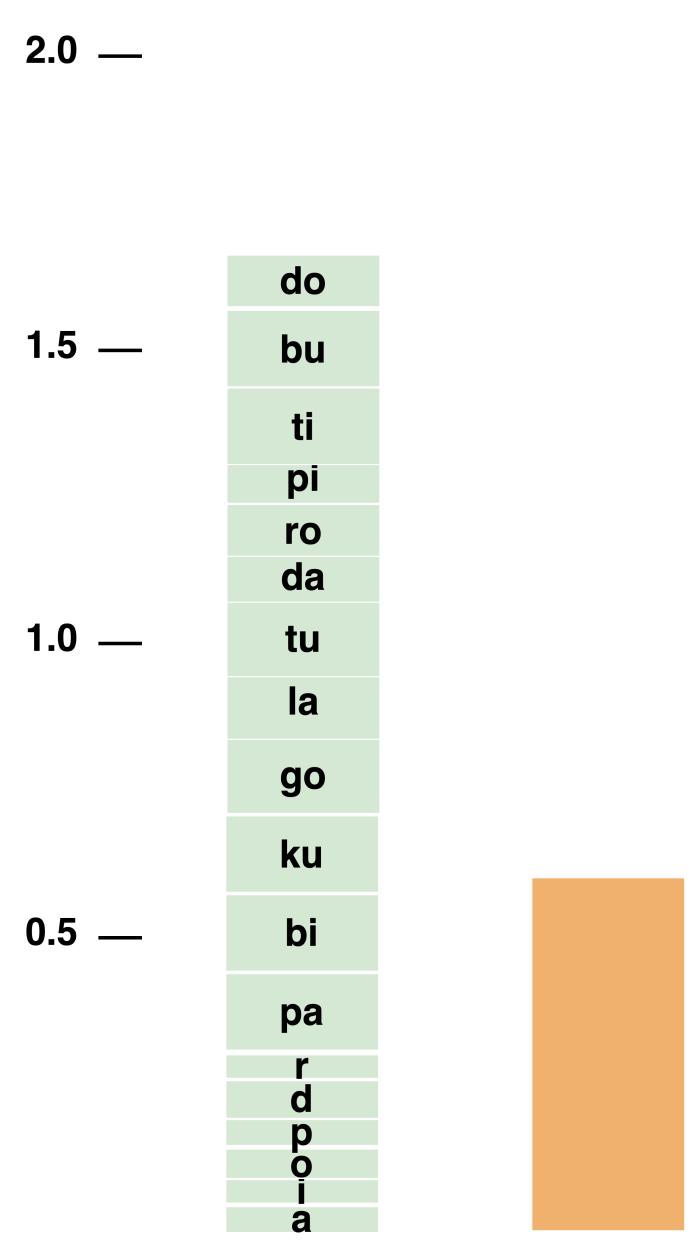

L(S) = 0.17 + 1.20 = 1.37 bits

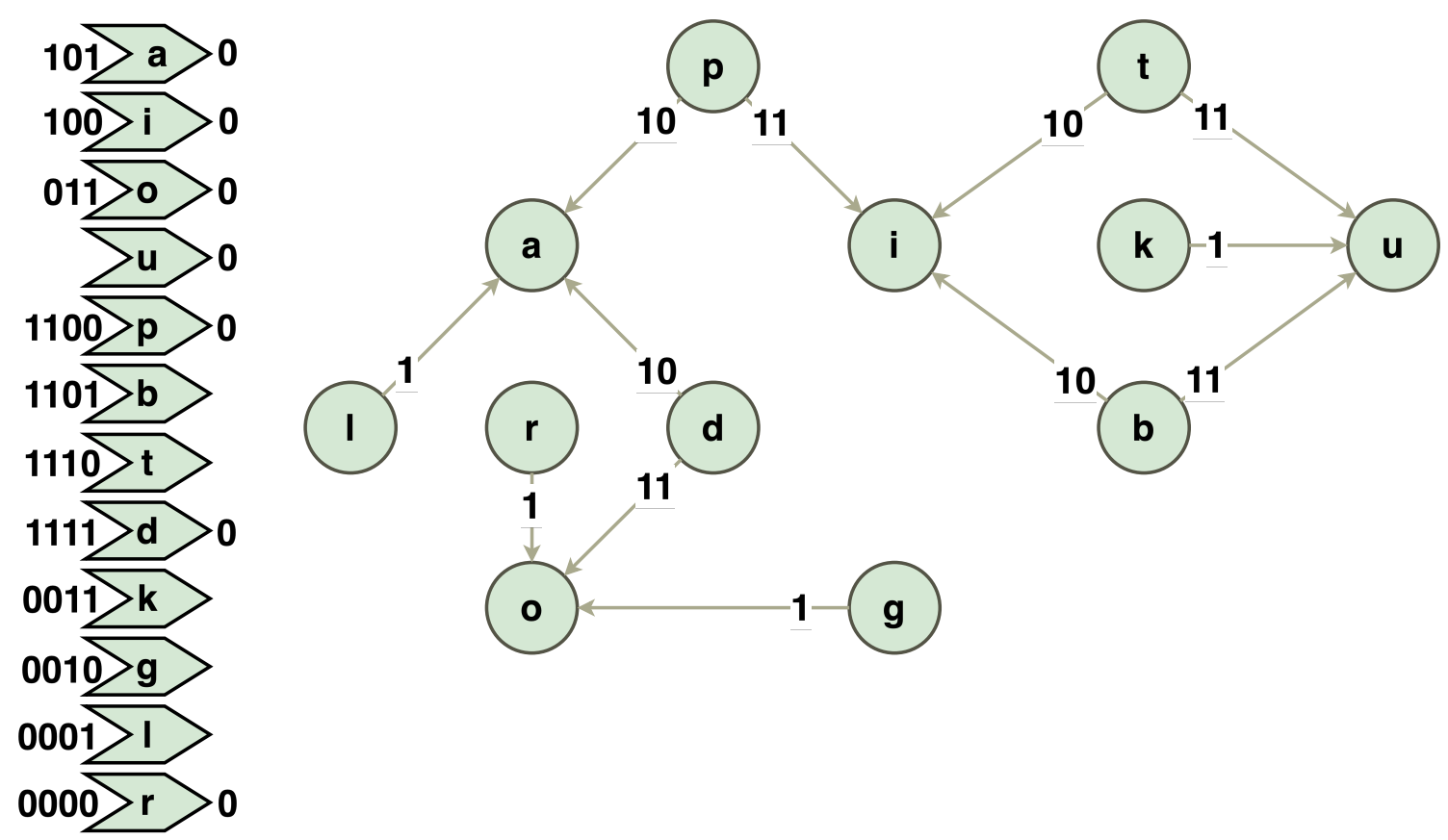

a → 101 0
i → 100 0
o → 011 0
p → 1100 0
d → 1111 0
r → 0000 0

p a → 1100 10 0
b i → 1101 10 0
k u → 0011 1 0
go → 0010 1 0
l a → 0001 1 0
t u → 1110 11 0
d a → 1111 10 0
r o → 0000 1 0
p i → 1100 11 0
t i → 1110 10 0
b u → 1101 11 0
d o → 1111 11 0

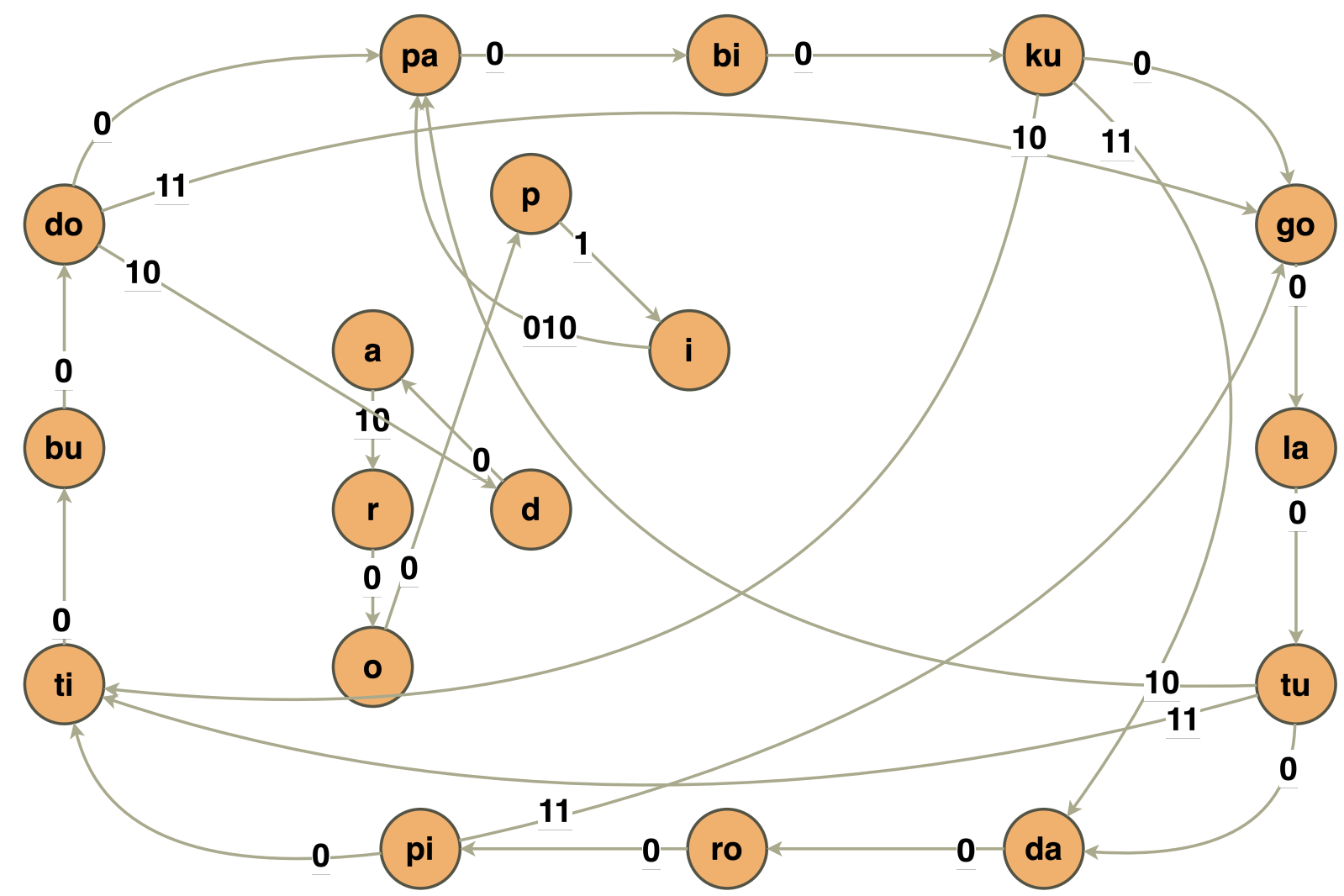

pa bi ku go la tu da ro pi ti bu do pa bi ku da ro pi go la tu ti bu do go la tu pa bi ku ti bu d o da r o p i → 0 0 0 0 0 0 0 0 0 0 0 0 0 0 11 0 0 11 0 0 11 0 0 11 0 0 10 0 0 10 0 0 10 0 10 0 0 1 010

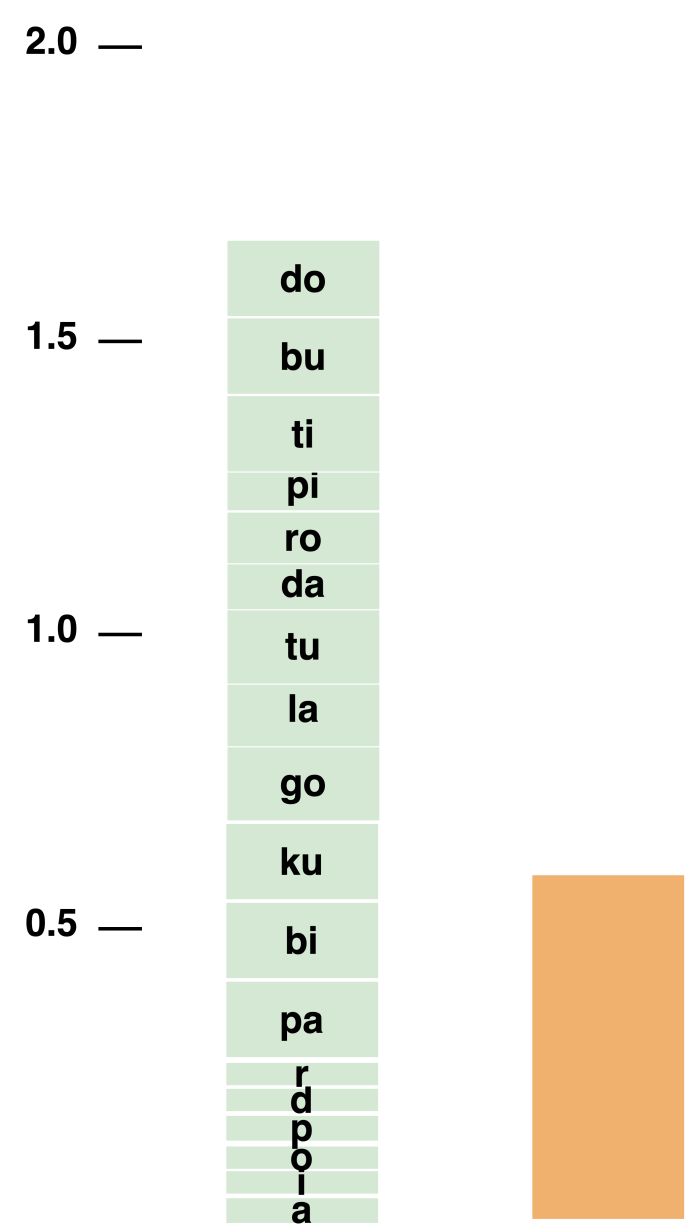

L(S) = 0.14 + 1.19 = 1.33 bits

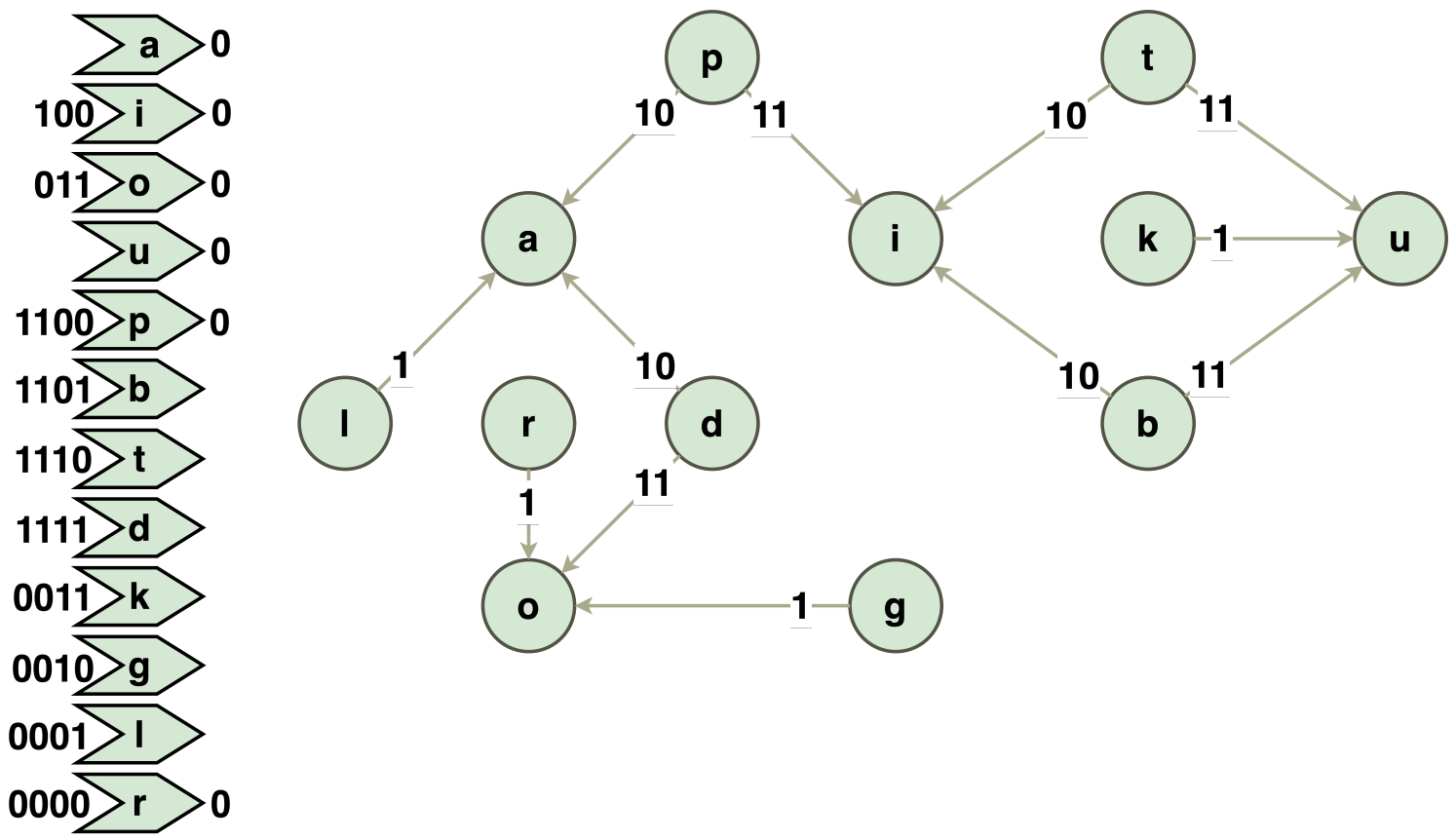

i → 100 0
o → 011 0

p → 1100 0

r → 0000 0

p a → 1100 10 0
b i → 1101 10 0
k u → 0011 1 0
go → 0010 1 0
l a → 0001 1 0
t u → 1110 11 0
d a → 1111 10 0
r o → 0000 1 0
p i → 1100 11 0
t i → 1110 10 0
b u → 1101 11 0
d o → 1111 11 0

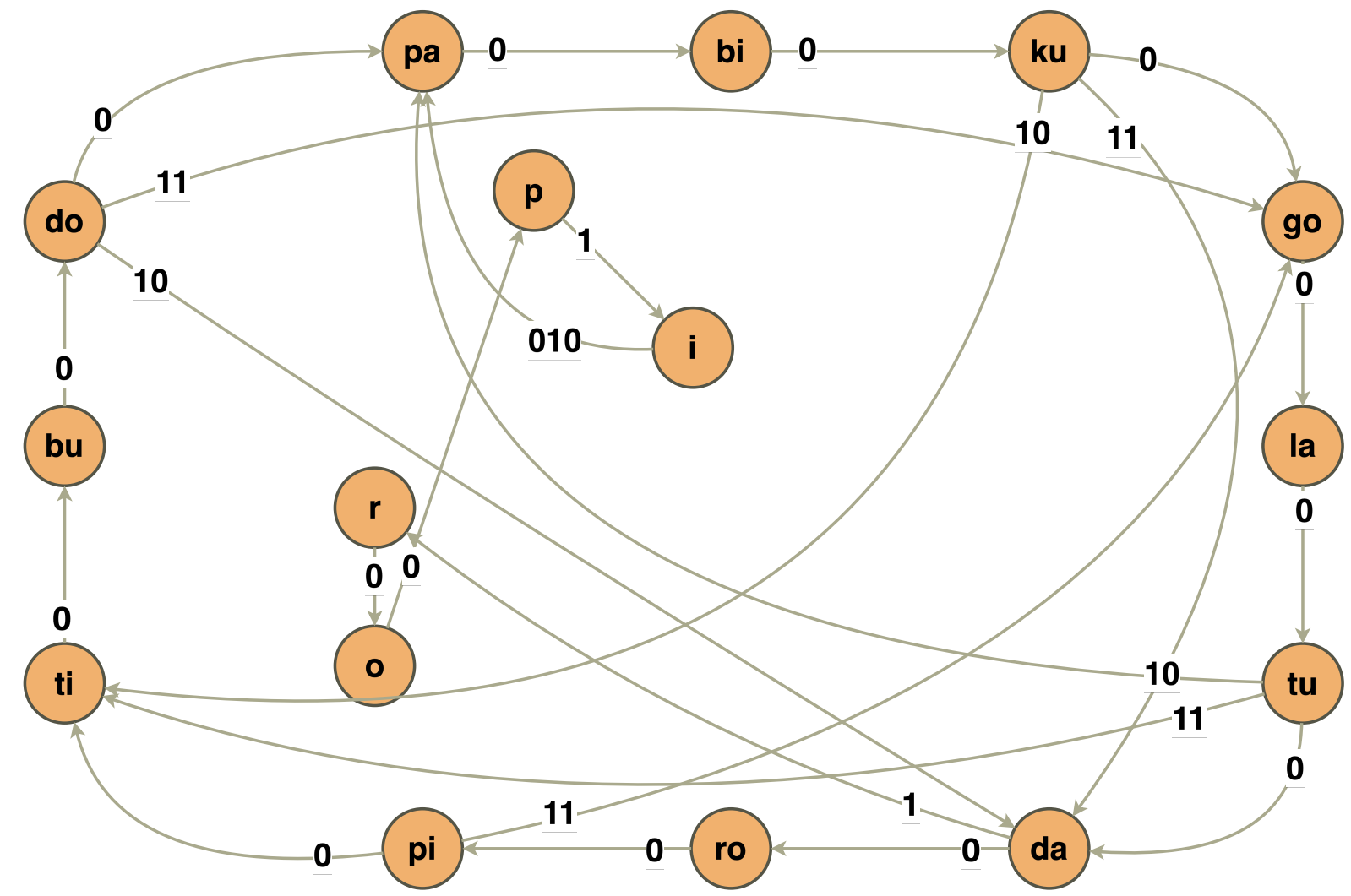

pa bi ku go la tu da ro pi ti bu do pa bi ku da ro pi go la tu ti bu do go la tu pa bi ku ti bu do da r o p i → 0 0 0 0 0 0 0 0 0 0 0 0 0 0 0 11 0 0 11 0 0 11 0 0 11 0 0 10 0 0 10 0 0 10 1 0 0 1 010

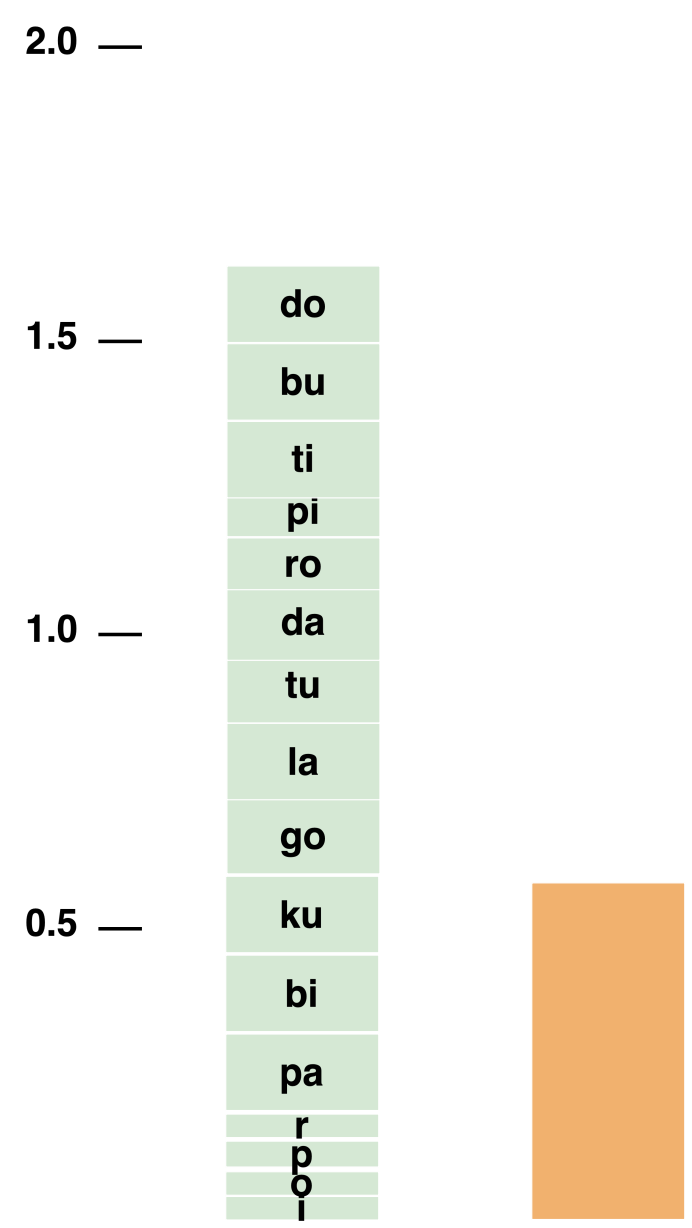

L(S) = 0.18 + 1.14 = 1.32 bits

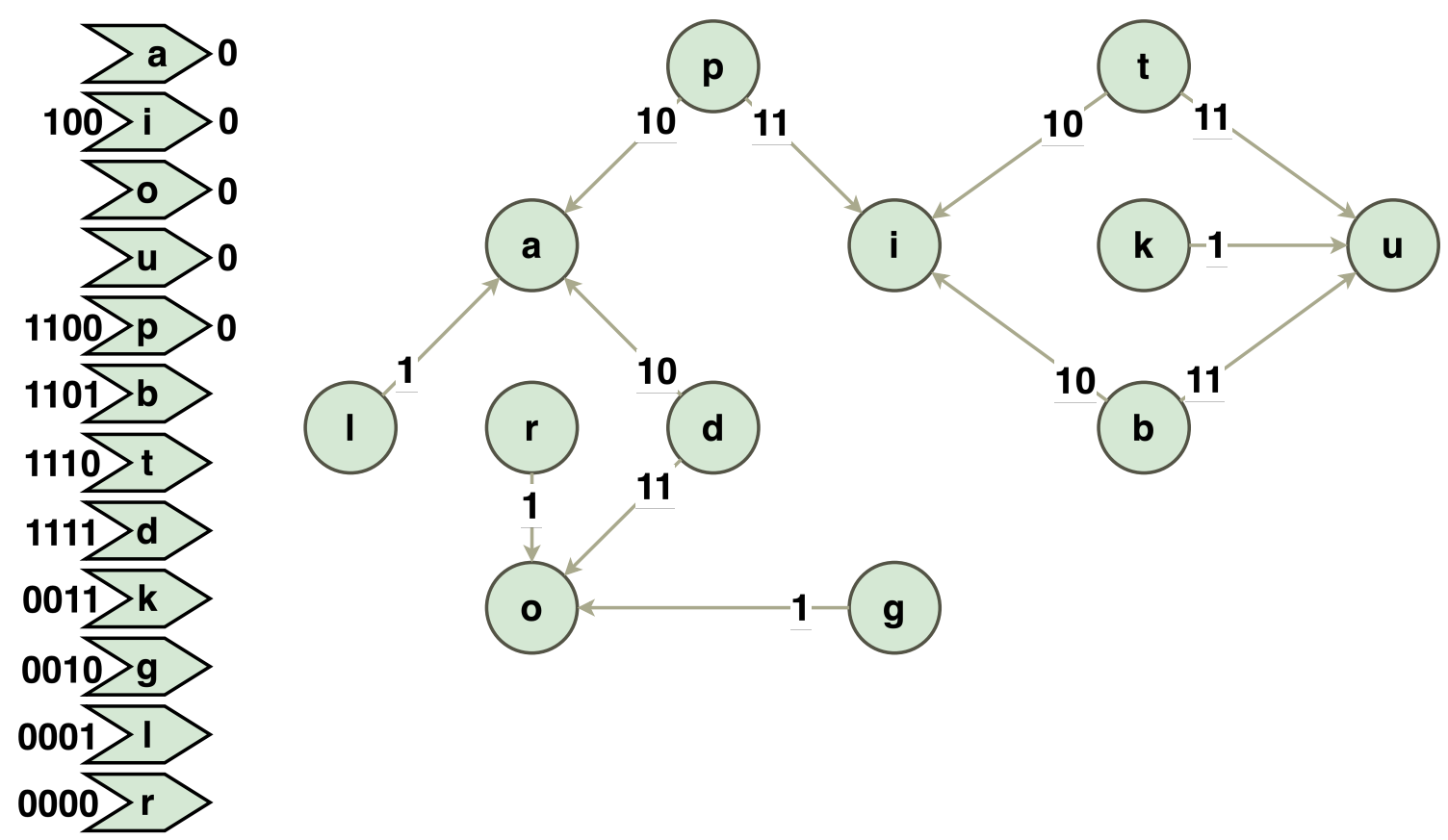

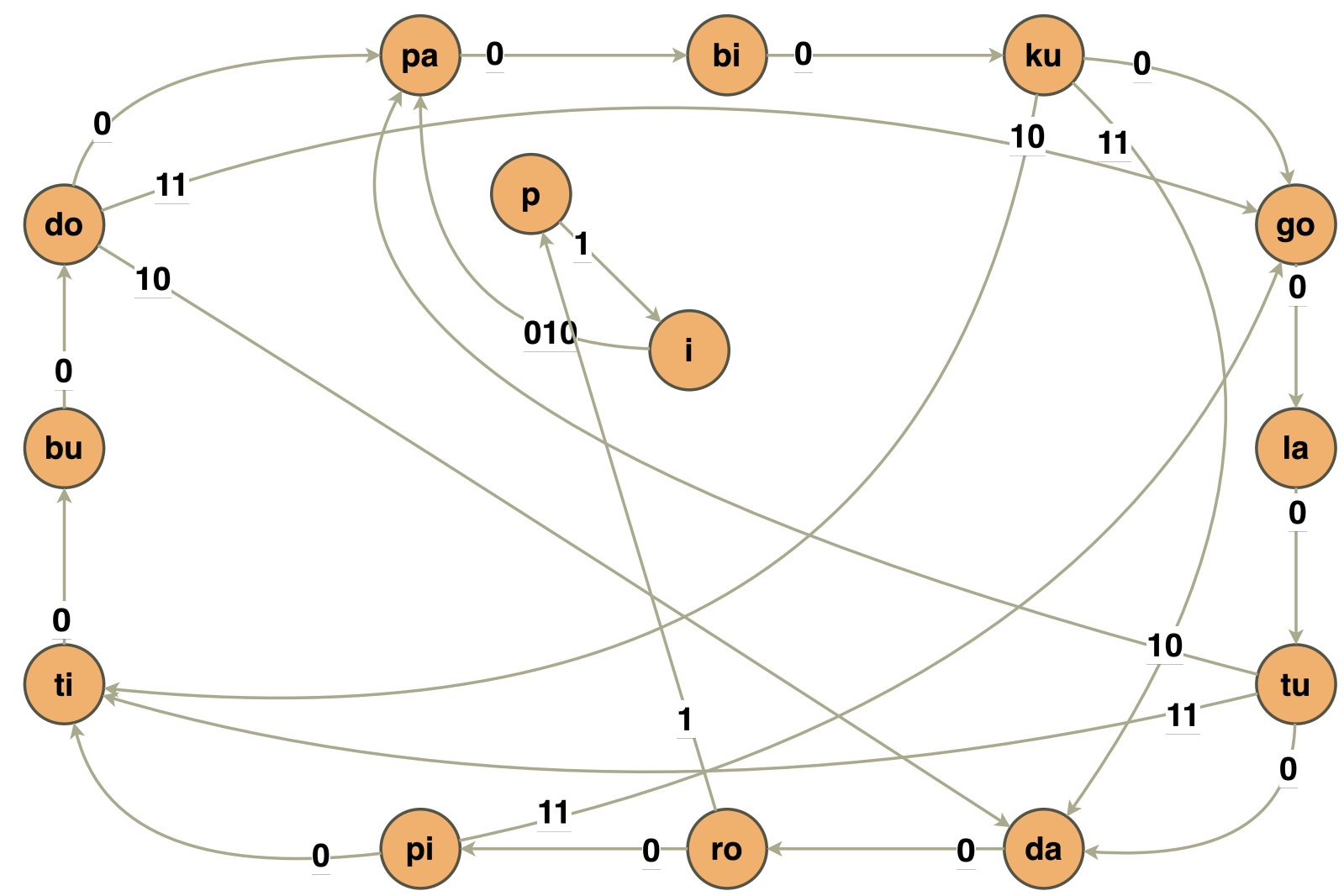

i → 100 0

p → 1100 0

p a → 1100 10 0
b i → 1101 10 0
k u → 0011 1 0
go → 0010 1 0
l a → 0001 1 0
t u → 1110 11 0
d a → 1111 10 0
r o → 0000 1 0
p i → 1100 11 0
t i → 1110 10 0
b u → 1101 11 0
d o → 1111 11 0

pa bi ku go la tu da ro pi ti bu do pa bi ku da ro pi go la tu ti bu do go la tu pa bi ku ti bu do da ro p i → 0 0 0 0 0 0 0 0 0 0 0 0 0 0 11 0 0 11 0 0 11 0 0 11 0 0 10 0 0 10 0 0 10 0 1 1 010

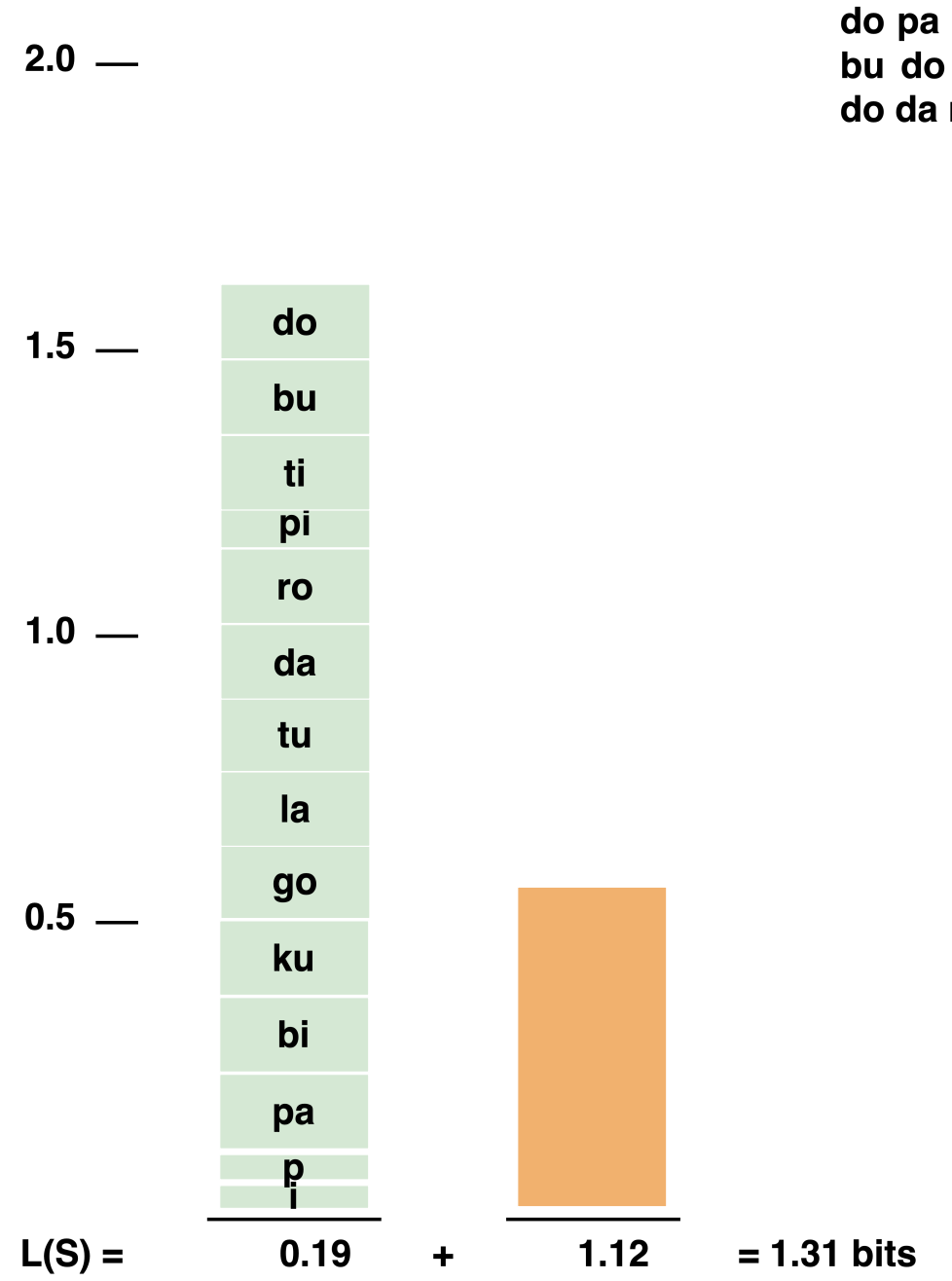

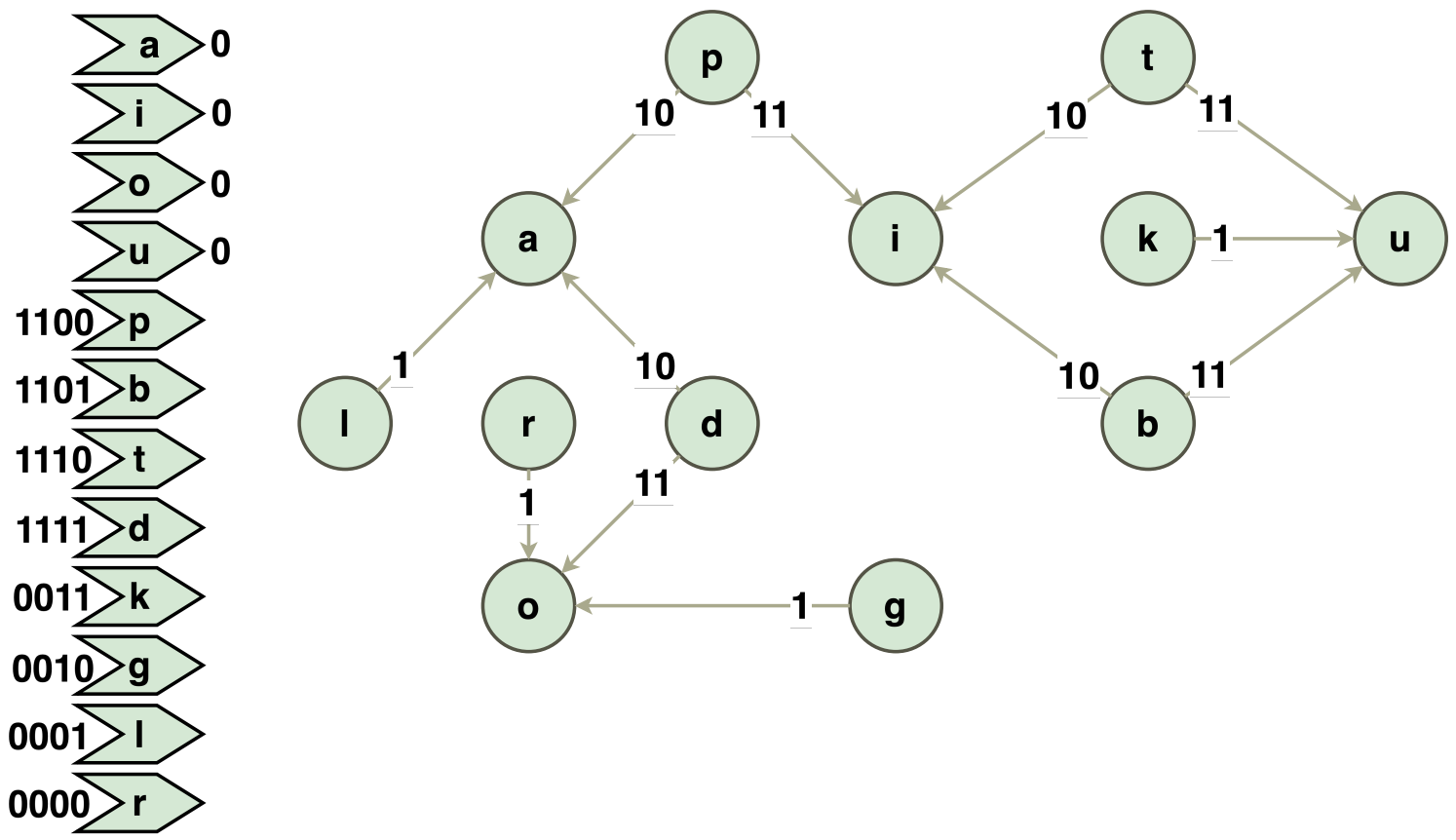

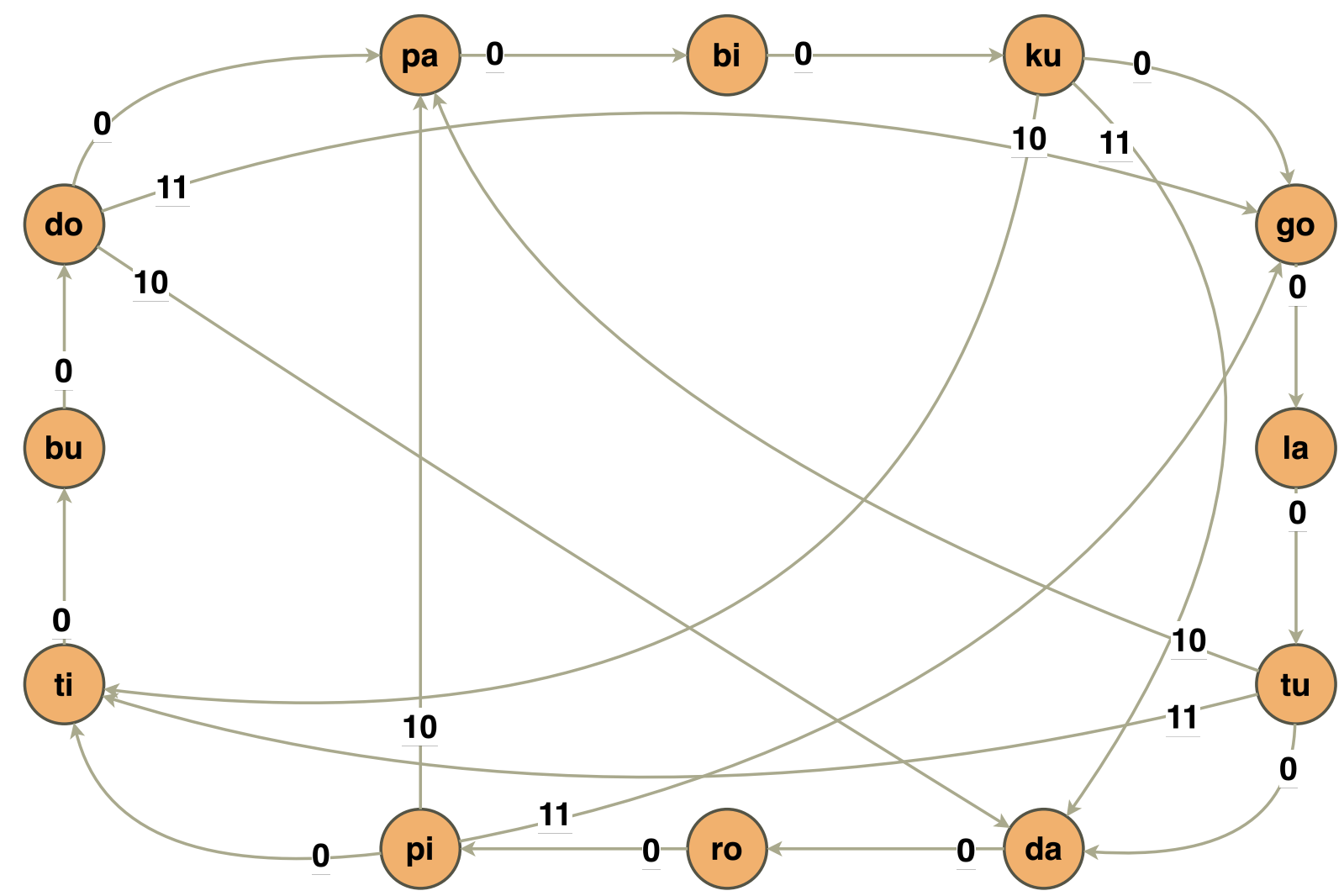

p a → 1100 10 0
b i → 1101 10 0
k u → 0011 1 0
go → 0010 1 0
l a → 0001 1 0
t u → 1110 11 0
d a → 1111 10 0
r o → 0000 1 0
p i → 1100 11 0
t i → 1110 10 0
b u → 1101 11 0
d o → 1111 11 0

pa bi ku go la tu da ro pi ti bu do pa bi ku da ro pi go la tu ti bu do go la tu pa bi ku ti bu do da ro pi → 0 0 0 0 0 0 0 0 0 0 0 0 0 0 11 0 0 11 0 0 11 0 0 11 0 0 10 0 0 10 0 0 10 0 0 10

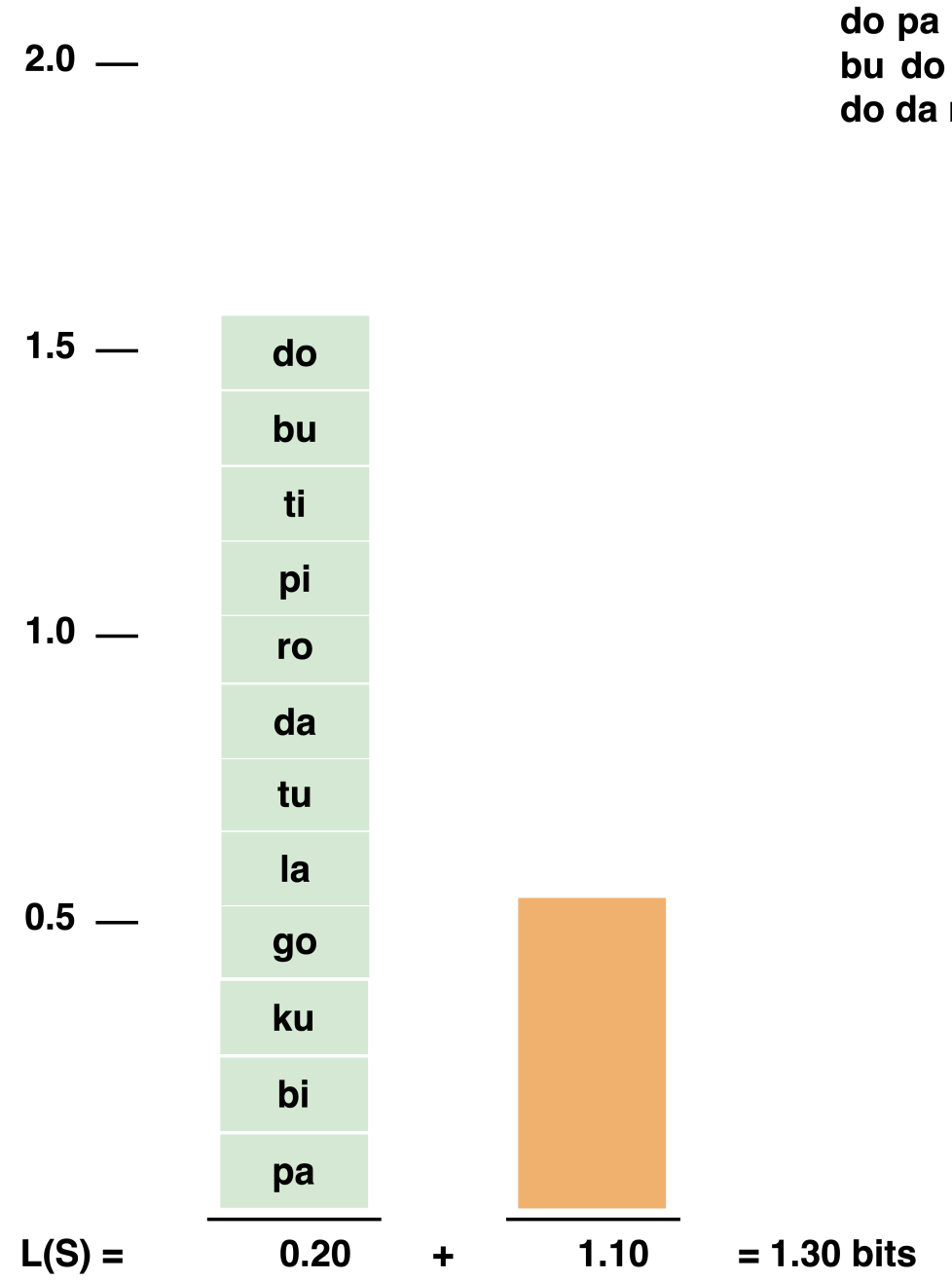

Legend for Dataset
We lowercase all letters before running the program.
We use multiple dots to indicate the levels. For example:
. indicates partition between first and second level
.. indicates partition between second and third level
... indicates partition between third and fourth level
And continues like that.

SI Dataset S1 (pabiku_lullaby_3-level.txt)

pabiku lullaby by Jenny Safran, with three levels of description

pa.bi.ku..go.la.tu..da.ro.pi..ti.bu.do..pa.bi.ku..da.ro.pi..go.la.tu..ti.bu.do..go.la.tu..pa.bi.ku..ti.bu.do..da.ro.pi

SI Dataset S2 (green_eggs_and_ham_4-level.txt)

Green Eggs and Ham by Dr. Seuss with four levels of description

Green.eggs.and.ham...I.am.Sam..I.am.Sam..Sam.I.am...That.Sam.I.am..That.Sam.I.am..I.do.not.like.that..Sam.I.am...Do.you.like.green.eggs.and.ham..I.do.not.like.them..Sam.I.am..I.do.not.like.green.eggs.and.ham...Would.you.like.them.here.or.there..I.would.not.like.them.here.or.there..I.would.not.like.them.any.where..I.do.not.like.green.eggs.and.ham..I.do.not.like.them..Sam.I.am...Would.you.like.them.in.a.house..Would.you.like.them.with.a.mouse...I.do.not.like.them.in.a.house..I.do.not.like.them.with.a.mouse...I.do.not.like.them.here.or.there..I.do.not.like.them.any.where..I.do.not.like.green.eggs.and.ham..I.do.not.like.them..Sam.I.am...Would.you.eat.them.in.a.box..Would.you.eat.them.with.a.fox...Not.in.a.box..Not.with.a.fox..Not.in.a.house..Not.with.a.mouse...I.would.not.eat.them.here.or.there..I.would.not.eat.them.any.where..I.would.not.eat.green.eggs.and.ham..I.do.not.like.them..Sam.I.am...Would.you..Could.you..In.a.car..Eat.them..Eat.them..Here.they.are..I.would.not.could.not.in.a.car...You.may.like.them..You.will.see..You.may.like.them.in.a.tree..I.would.not.could.not.in.a.tree..Not.in.a.car..You.let.me.be...I.do.not.like.them.in.a.box..I.do.not.like.them.with.a.fox..I.do.not.like.them.in.a.house..I.do.not.like.them.with.a.mouse...I.do.not.like.them.here.or.there..I.do.not.like.them.any.where..I.do.not.like.green.eggs.and.ham..I.do.not.like.them..Sam.I.am...A.train..A.train..A.train..A.train..Could.you.would.you.on.a.train..Not.on.train..Not.in.a.tree..Not.in.a.car..Sam.Let.me.be...I.would.not.could.not.in.a.box..I.could.not.would.not.with.a.fox..I.will.not.eat.them.with.a.mouse..I.will.not.eat.them.in.a.house...I.will.not.eat.them.here.or.there..I.will.not.eat.them.any.where..I.do.not.eat.green.eggs.and.ham..I.do.not.like.them..Sam.I.am...Say.In.the.dark..Here.in.the.dark..Would.you.could.you.in.the.dark..I.would.not.could.not.in.the.dark...Would.you.could.you.in.the.rain..I.would.not.could.not.in.the.rain..Not.in.the.dark..Not.on.a.train..Not.in.a.car..Not.in.a.tree..I.do.not.like.them.Sam.you.see...Not.in.a.house..Not.in.a.box..Not.with.a.mouse..Not.with.a.fox..I.will.not.eat.them.here.or.there..I.do.not.like.them.any.where...You.do.not.like.green.eggs.and.ham..I.do.not.like.them..Sam.I.am...Could.you.would.you.with.a.goat..I.would.not.could.not.with.a.goat...Would.you.could.you.on.a.boat..I.could.not.would.not.on.a.boat..I.will.not.will.not.with.a.goat..I.will.not.eat.them.in.the.rain..I.will.not.eat.them.on.a.train...Not.in.the.dark..Not.in.a.tree..Not.in.a.car..You.let.me.be...I.do.not.like.them.in.a.box..I.do.not.like.them.with.a.fox..I.will.not.eat.them.in.a.house..I.do.not.like.them.with.a.mouse..I.do.not.like.them.here.or.there..I.do.not.like.them.any.where...I.do.not.like.green.eggs.and.ham..I.do.not.like.them..Sam.I.am...You.do.not.like.them..So.you.say..Try.them..Try.them..And.you.may..Try.them.and.you.may.I.say...Sam.If.you.will.let.me.be..I.will.try.them..You.will.see...Say.I.like.green.eggs.and.ham..I.do..I.like.them..Sam.I.am..And.I.would.eat.them.in.a.boat..And.I.would.eat.them.with.a.goat...And.I.will.eat.them.in.the.rain..And.in.the.dark..And.on.a.train..And.in.a.car..And.in.a.tree..They.are.so.good.so.good.you.see...So.I.will.eat.them.in.a.box..And.I.will.eat.them.with.a.fox..And.I.will.eat.them.in.a.house..And.I.will.eat.them.with.a.mouse..And.I.will.eat.them.here.and.there..Say.I.will.eat.them.any.where...I.do.so.like.green.eggs.and.ham..Thank.you..Thank.you..Sam.I.am

SI Dataset S3 (the_cat_in_the_hat_4-level.txt)

The Cat in the Hat by Dr. Seuss with four levels of description

the.cat.in.the.hat...The.sun.did.not.shine..It.was.too.wet.to.play..So.we.sat.in.the.house..All.that.cold.cold.wet.day...I.sat.the re.with.Sally..We.sat.there.we.two..And.I.said.How.I.wish..We.had.something.to.do...Too.wet.to.go.out..And.too.cold.to.play .ball..So.we.sat.in.the.house..We.did.nothing.at.all...So.all.we.could.do.was.to..Sit.Sit.Sit.Sit..And.we.did.not.like.it..Not.one. little.bit...And.then.something.went.bump..How.that.bump.made.us.jump...We.looked..Then.we.saw.him.step.in.on.the.mat.. We.looked..And.we.saw.him..The.Cat.in.the.Hat..And.he.said.to.us..Why.do.you.sit.there.like.that...I.know.it.is.wet..And.th e.sun.is.not.sunny..But.we.can.have..Lots.of.good.fun.that.is.funny...I.know.some.good.games.we.could.play..Said.the.cat..I.k now.some.new.tricks..Said.the.Cat.in.the.Hat..A.lot.of.good.tricks..I.will.show.them.to.you..Your.mother..Will.not.mind.at.a ll.if.I.do...Then.Sally.and.I..did.not.know.what.to.say..Our.mother.was.out.of.the.house..for.the.day...But.our.fish.said.No.No ..Make.that.cat.go.away..Tell.that.cat.in.the.hat..You.do.not.want.to.play...He.should.not.be.here..He.should.not.be.about.. He.should.not.be.here..When.your.mother.is.out...Now.Now.Have.no.fear..Have.no.fear.said.the.cat..My.tricks.are.not.bad..S aid.the.Cat.in.the.Hat...Why.we.can.have..Lots.of.good.fun.if.you.wish..With.a.game.that.I.call..Up.Up.Up.with.a.fish...Put. me.down.said.the.fish..This.is.no.fun.at.all..Put.me.down.said.the.fish..I.do.not.wish.to.fall...Have.no.fear.said.the.cat..I.will. not.let.you.fall..I.will.hold.you.up.high..As.I.stand.on.a.ball..With.a.book.on.one.hand..And.a.cup.on.my.hat..But.that.is.not. All.I.can.do..Said.the.cat...Look.at.me..Look.at.me.now..said.the.cat..With.a.cup.and.a.cake..On.the.top.of.my.hat..I.can.hol d.up.Two.books..I.can.hold.up.the.fish...And.a.little.toy.ship..And.some.milk.on.a.dish..And.look...I.can.hop.up.and.down.on. the.ball..But.that.is.not.all..Oh.no..That.is.not.all...Look.at.me..Look.at.me..Look.at.me.Now..It.is.fun.to.have.fun..But.you. have.to.know.how...I.can.hold.up.the.cup..And.the.milk.and.the.cake..I.can.hold.up.these.books..And.the.fish.on.a.rake...I.ca n.hold.the.toy.ship..And.a.little.toy.man..And.look.With.my.tail..I.can.hold.a.red.fan..I.can.fan.with.the.fan..As.I.hop.on.the. ball..But.that.is.not.all..Oh.no.That.is.not.all...That.is.what.the.cat.said..Then.he.fell.on.his.head..He.came.down.with.a.bu mp..From.up.there.on.the.ball..And.Sally.and.I..We.saw.all.the.things.fall...And.our.fish.came.down.too..He.fell.into.a.pot..H e.said.Do.I.like.this..Oh.no.I.do.not..This.is.not.a.good.game..Said.our.fish.as.he.lit..No.I.do.not.like.it..Not.one.little.bit...No w.look.what.you.did..Said.the.fish.to.the.cat..Now.look.at.this.house..Look.at.this.Look.at.that.You.sank.our.toy.ship..Sank.i t.deep.in.the.cake...You.shook.up.our.house..And.you.bent.our.new.rake..You.SHOULD.NOT.be.here..When.our.mother.is.n ot..You.get.out.of.this.house..Said.the.fish.in.the.pot...But.I.like.to.be.here..Oh.I.like.it.a.lot..Said.the.Cat.in.the.Hat..To.the. fish.in.the.pot...I.will.NOT.go.away..I.do.NOT.wish.to.go..And.so.said.the.Cat.in.the.Hat..So.so.so.I.will.show.you..Another. good.game.that.I.know...And.then.he.ran.out..And.then.fast.as.a.fox..The.Cat.in.the.Hat..Came.back.in.with.a.box...A.big.re d.wood.box..It.was.shut.with.a.hook..Now.look.at.this.trick..Said.the.cat..Take.a.look...Then.he.got.up.on.top..With.a.tip.of. his.hat..I.call.this.game.FUN.IN.A.BOX..Said.the.cat...In.this.box.are.two.things..I.will.show.to.you.now..You.will.like.these. two.things..Said.the.cat.with.a.bow...I.will.pick.up.the.hook..You.will.see.something.new..Two.things..And.I.call.them...Thin g.One.and.Thing.Two..These.Things.will.not.bite.you..They.want.to.have.fun...Then.out.of.the.box..Came.Thing.Two.and.T hing.One..And.they.ran.to.us.fast..They.said.How.do.you.do..Would.you.like.to.shake.hands..With.Thing.One.and.Thing.Tw o...And.Sally.and.I..Did.not.know.what.to.do..So.we.had.to.shake.hands..With.Thing.One.and.Thing.Two..We.shook.their.t wo.hands...But.our.fish.said.No.No..Those.Things.should.not.be..In.this.house..Make.them.go...They.should.not.be.here..Wh en.your.mother.is.not..Put.them.out..Put.them.out..Said.the.fish.in.the.pot...Have.no.fear.little.fish..Said.the.Cat.in.the.Hat.. These.Things.are.good.Things..And.he.gave.them.a.pat..They.are.tame.Oh.so.tame..They.have.come.here.to.play..They.will. give.you.some.fun..On.this.wet.wet.wet.day...Now.here.is.a.game.that.they.like..Said.the.cat..They.like.to.fly.kites..Said.the. Cat.in.the.Hat...No.Not.in.the.house..Said.the.fish.in.the.pot..They.should.not.fly.kites..In.a.house.They.should.not..Oh.the.t hings.they.will.bump..Oh.the.things.they.will.hit..Oh.I.do.not.like.it..Not.one.little.bit...Then.Sally.and.I..Saw.them.run.dow n.the.hall..We.saw.those.two.Things..Bump.their.kites.on.the.wall..Bump.Thump.Thump.Bump..Down.the.wall.in.the.hall... Thing.Two.and.Thing.One..They.ran.up..They.ran.down..On.the.string.of.one.kite...We.saw.Mothers.new.gown..Her.gown.w ith.the.dots..That.are.pink.white.and.red.Then.we.saw.one.kite.bump..On.the.head.of.her.bed...Then.those.Things.ran.about. .With.big.bumps.jumps.and.kicks..And.with.hops.and.big.thumps..And.all.kinds.of.bad.tricks..And.I.said..I.do.not.like.the.w ay.that.they.play..If.Mother.could.see.this..Oh.what.would.she.say...Then.our.fish.said.LOOK.LOOK..And.our.fish.shook.wi th.fear..Your.mother.is.on.her.way.home..Do.you.hear..Oh.what.will.she.do.to.us..What.will.she.say..Oh.she.will.not.like.it.. To.find.us.this.way...So.DO.something.Fast..said.the.fish..Do.you.hear..I.saw.her..Your.mother..Your.mother.is.near..So.as.f ast.as.you.can..Think.of.something.to.do..You.will.have.to.get.rid.of..Thing.One.and.Thing.Two...So.as.fast.as.I.could..I.wen t.after.my.net..And.I.said.With.my.net..I.can.get.them.I.bet..I.bet.with.my.net..I.can.get.those.Things.yet...Then.I.let.down. my.net..It.came.down.with.a.PLOP..And.I.had.them.At.last..Those.two.Things.had.to.stop..Then.I.said.to.the.cat..Now.you. do.as.I.say..You.pack.up.those.Things..And.you.take.them.away...Oh.dear.said.the.cat..You.did.not.like.our.game..Oh.dear..

What.a.shame..What.a.shame..What.a.shame...Then.he.shut.up.the.Things..In.the.box.with.the.hook..And.the.cat.went.away..With.a.sad.kind.of.look...That.is.good.said.the.fish..He.has.gone.away.Yes..But.your.mother.will.come..She.will.find.this.big.mess...And.this.mess.is.so.big..And.so.deep.and.so.tall..We.can.not.pick.it.up..There.is.no.way.at.all...And.THEN..Who.was.back.in.the.house..Why.the.cat..Have.no.fear.of.this.mess..Said.the.Cat.in.the.Hat...I.always.pick.up.all.my.play.things..And.so..I.will.show.you.another..Good.trick.that.I.know...Then.we.saw.him.pick.up..All.the.things.that.were.down..He.picked.up.the.cake..And.the.rake.and.the.gown..And.the.milk.and.the.strings..And.the.books.and.the.dish...And.the.fan.and.the.cup..And.the.ship.and.the.fish..And.he.put.them.away..Then.he.said.That.is.that..And.then.he.was.gone..With.a.tip.of.his.hat...Then.our.mother.came.in..And.she.said.to.us.two..Did.you.have.any.fun..Tell.me.What.did.you.do...And.Sally.and.I.did.not.know..What.to.say..Should.we.tell.her..The.things.that.went.on.there.that.day...Should.we.tell.her.about.it..Now.what.SHOULD.we.do..Well.What.would.YOU.do..If.your.mother.asked.YOU...

SI Dataset S4 (midnight_on_the_moon_*level.txt*)

Midnight On the Moon by Mary Pope Osborne with four levels of description

MIDNIGHT.ON.THE.MOON..BY.MOONLIGHT..Jack.whispered.a.voice..Jack.opened.his.eyes..He.saw.a.figure.in.the.moonlight..Wake.up.Get.dressed..It.was.his.sister.Annie..Jack.turned.on.his.lamp..He.rubbed.his.eyes...Annie.was.standing.beside.his.bed..She.wore.jeans.and.a.sweatshirt..Lets.go.to.the.tree.house.she.said..What.time.is.it.asked.Jack..He.put.on.his.glasses..Dont.look.at.your.clock.said.Annie..Jack.looked.at.his.clock..Oh.man.he.said..Its.midnight..Its.too.dark...No.it.isnt..The.moon.makes.it.bright.enough.to.see.said.Annie..Wait.till.morning.said.Jack..No.now.said.Annie..We.have.to.find.the.fourth.M.thing..I.have.a.feeling.that.the.full.moon.might.help.us.Thats.nuts.said.Jack..I.want.to.sleep..You.can.sleep.when.we.come.back.home.said.Annie..No.time.will.have.passed...Jack.sighed.Oh.brother.he.said..But.he.got.out.of.bed..Yay.whispered.Annie..Meet.you.at.the.back.door..She.tiptoed.out.of.Jacks.room..Jack.yawned...He.pulled.on.his.jeans.and.sneakers.and.a.sweatshirt..He.put.his.notebook.and.pencil.into.his.backpack..Then.he.crept.down.the.stairs..Annie.opened.the.back.door..Quietly.they.stepped.outside..Wait.said.Jack.We.need.a.flashlight...No.we.dont.I.told.you..the.moon.will.light.our.way.said.Annie..And.she.took.off..Jack.sighed.then.followed.her..Annie.was.right.thought.Jack..The.moon.was.so.bright.that.he.could.see.his.shadow..Everything.seemed.washed.with.silver...Soon.they.left.their.street..Annie.led.the.way.into.the.Frog.Creek.woods..It.was.much.darker.under.the.shadows.of.the.trees..Jack.looked.up.searching.for.the.tree.house..There.said.Annie..The.magic.tree.house.was.shining.in.the.moonlight..Annie.grabbed.the.rope.ladder.and.started.climbing.up..Careful.go.slowly.said.Jack..He.followed.her.up.the.ladder.and.into.the.tree.house...Moonlight.streamed.through.the.window..It.shone.on.the.letter.M.that.shimmered.on.the.wooden.floor..It.shone.on.the.three.M.things.that.rested.on.the.M..a.moonstone.from.the.time.of.the.ninjas..a.mango.from.the.Amazon.rain.forest..and.a.mammoth.bone.from.the.Ice.Age..We.need.just.one.more.M.thing.said.Annie..to.free.Morgan.from.her.spell...Squeak.Peanut.said.Annie..In.the.dim.light.Jack.saw.a.tiny.mouse..She.sat.on.an.open.book..You.didnt.expect.to.see.us.this.late.did.you.said.Annie..She.picked.up.Peanut..And.Jack.picked.up.the.open.book..So.where.are.we.going.this.time.Annie.asked.him...Jack.held.the.book.up.to.the.moonlight..Uh.oh.he.said..I.knew.we.should.have.brought.a.flashlight..I.cant.read.a.thing..He.could.make.out.diagrams.and.shadowy.pictures..But.he.couldnt.read.a.word..Look.at.the.cover.said.Annie..The.letters.were.bigger.on.the.cover..Jack.squinted.at.them...Its.called.Hello.Moon.he.said..Annie.gasped.Were.going.to.the.moon..Of.course.not.said.Jack..Its.impossible.to.go.to.the.moon.without.tons.of.equipment..Why.Theres.no.air..We.couldnt.breathe..Not.only.that.wed.boil.to.death.if.it.was.day.and.freeze.to.death.if.it.was.night..Yikes.said.Annie.So.where.do.you.think.we.are.going...Maybe.a.place.where.people.train.to.be.astronauts.said.Jack..That.sounds.neat.said.Annie..Yeah.said.Jack..Hed.always.wanted.to.meet.astronauts.and.space.scientists..So.say.the.wish.said.Annie..Jack.opened.the.book.again..He.pointed.to.a.picture.of.a.dome.shaped.structure..I.wish.we.could.go.there.he.said..The.wind.started.to.blow..The.tree.house.started.to.spin..It.spun.faster.and.faster.and.faster..Then.everything.was.silent..Absolutely.silent.As.quiet.and.still.as.silence.could.be...SPACE.MOTEL..Jack.opened.his.eyes..He.looked.out.the.window..The.tree.house.had.landed.inside.a.large.white.room..What.kind.of.training.place.is.this.asked.Annie..I.dont.know.said.Jack..The.room.was.round..It.had.no.windows..It.had.white.floors.and.a.curved.wall.lit.by.bright.lights..Hello.Annie.called..There.was.no.answer..Where.were.all.the.astronauts.and.space.scientists.Jack.wondered..Theres.nobody.here.said.Annie...How.do.you.know.said.Jack..I.just.feel.it.said.Annie..Wed.better.find.out.where.we.are.said.Jack..He.looked.at.the.page.in.the.moon.book..He.read.the.words.below.the.picture.of.the.dome..A.moon.base.was.built.on.the.moon.in.the.year..The.top.of.the.dome.slides.open.to.let.spacecrafts.enter.and.leave...Oh.man.Jack.whispered..What.is.wrong.said.Annie..Jacks.heart.pounded.with.excitement..He.could.hardl

y.speak..Weve.landed.inside.a.moon.base.he.said..So.said.Annie..So.the.moon.base.is.on.the.moon.said.Jack..Annies.eyes.wid ened..Were.on.the.moon.she.asked...Jack.nodded.The.book.says.the.moon.base.was.built.in.the.year.he.said..So.this.book.wa s.written.after.that..Which.means.this.book.is.from.the.future..Oh.wow.said.Annie..Morgan.must.have.gone.forward.in.time. to.borrow.it.from.a.future.library..Right.said.Jack.And.now.were.in.the.future.on.the.moon..Squeak.squeak...Annie.and.Jack. looked.at.Peanut..The.mouse.was.running.around.in.circles..Poor.Peanut.said.Annie..She.tried.to.pick.the.mouse.up..But.Pe anut.hid.behind.the.mango.on.the.letter.M..Maybe.she.is.nervous.about.being.on.the.moon.said.Annie..She.is.not.the.only.on e.said.Jack..He.let.out.a.deep.breath.then.he.pushed.his.glasses.into.place..So.what.is.a.moon.base.asked.Annie...Jack.looked. at.the.book..He.read.aloud..When.scientists.visit.the.moon.for.short.periods.they.eat.and.sleep.in.the.moon.base..A.space.mo tel.said.Annie..I.guess.said.Jack.He.read.more..The.small.base.has.a.landing.chamber.and.a.room.for.storing.spacesuits..Air.a nd.temperature.controls.make.breathing.possible...So.thats.why.we.can.breathe.Jack.said..Lets.explore.said.Annie..We.have. to.find.the.fourth.thing.for.Morgan..No.first.we.should.study.this.map.said.Jack..He.pulled.out.his.notebook..You.study.it.sa id.Annie..Jack.copied.the.map..Then.he.drew.in.the.tree.house...Okay.he.said.He.pointed.at.the.X.in.his.drawing.Were.here.. Jack.looked.up.Annie.was.gone..Oh.brother.Jack.said..As.usual.she.had.left.without.him..Before.they.could.even.make.a.plan ..Jack.put.the.moon.book.and.pencil.into.his.pack..Carrying.his.notebook.and.backpack.he.started.out.the.window..Squeak.S queak.Jack.looked.back.at.Peanut..The.mouse.was.running.back.and.forth.on.the.M...Stay.here.and.be.safe.said.Jack..We.wi ll.be.back.soon..Jack.swung.himself.over.the.window.sill..His.feet.touched.the.floor.of.the.landing.chamber..Annie.he.called.. There.was.no.answer..Jack.looked.at.his.diagram..It.showed.only.one.way.to.go..Jack.walked.along.the.curved.white.wall.to.t he.stairs..He.climbed.the.steps.to.a.hallway...Jack.hurry.Annie.was.at.the.end.of.the.hallway.standing.in.the.airlock..She.pee red.out.a.window.in.a.giant.door.Jack.hurried.toward.her..Annie.stepped.aside.so.he.could.look.out.the.window.too..Oh.man. said.Jack.What.he.saw.took.his.breath.away...He.stared.at.a.rocky.gray.land..The.land.was.filled.with.giant.craters.and.tall. mountains..The.sun.was.shining.But.the.sky.was.ink.black..Say.hi.to.the.moon.Annie.said.softly...OPEN.SESAME..The.four th.M.thing.must.be.out.there.said.Annie..Beside.the.door.was.a.button.with.the.word.OPEN.on.it..Annie.reached.for.the.but ton..Wait.Jack.grabbed.her.hand..Theres.no.air.on.the.moon.Remember..Oh.Right.But.we.have.to.go.out.to.find.the.M.thin g..Lets.see.what.the.book.says.said.Jack..He.pulled.the.book.out.of.his.pack..He.flipped.through.it.until.he.found.a.page.that. showed.the.surface.of.the.moon..He.read.aloud..It.takes.fourteen.Earth.days.to.equal.one.day.on.the.moon..No.air.protects.t he.moon.from.the.suns.rays.so.daytime.heat.reaches.degrees...Jack.looked.at.Annie..I.told.you.our.blood.would.boil.if.we.wen t.out.there.he.said..Yuck.she.said..Jack.read.from.the.book.again..Moon.scientists.wear.spacesuits.which.have.controls.to.kee p.them.from.getting.too.hot.or.too.cold..They.have.tanks.which.provide.air.for.two.hours...Where.do.we.get.spacesuits.asked. Annie..She.looked.around.then.trotted.back.down.the.hall.Maybe.there.Jack.was.studying.his.map..Lets.try.the.spacesuit.sto reroom...Dont.look.at.the.map.said.Annie..Look.at.the.real.room..Jack.glanced.up.Annie.was.peering.through.a.doorway.off.t he.hall..Theres.a.ton.of.space.stuff.in.here.she.said..Jack.went.to.look..Bulky.white.suits.hung.from.hangers..Air.tanks.helme ts.gloves.and.boots.sat.in.neat.rows.on.shelves..Wow.its.like.the.armor.room.in.a.castle.said.Jack.Yeah.with.huge.armor.said. Annie...Lets.pick.out.the.smallest.stuff.said.Jack..The.suits.can.go.over.our.clothes..Annie.found.the.smallest.white.suit..And. Jack.found.the.next.smallest..They.stepped.into.them..Then.Annie.locked.Jacks.air.tank.into.place..Thanks.he.said..And.he. did.the.same.for.her..Thanks.she.said...Gloves.said.Jack..He.and.Annie.pulled.on.white.gloves.Boots.said.Annie..They.each.p ulled.on.a.pair.of.huge.white.boots..Helmets.said.Jack.He.reached.for.a.helmet..Wow.theyre.pretty.light.he.said..I.thought.th ey.would.be.like.knights.helmets...Jack.and.Annie.put.the.helmets.on..They.locked.each.others.into.place..I.cant.move.my.he ad.right.or.left.said.Annie..Me.neither.said.Jack.Lets.try.walking..Jack.and.Annie.moved.clumsily.around.the.room..Jack.felt. like.a.fat.snowman..Close.your.visor.said.Annie..They.both.closed.their.see.through.visors..Cool.air.filled.Jacks.helmet...I.CA N.BREATHE.Annie.yelled..Her.voice.boomed.in.Jacks.ears..Ow.Talk.quietly.Jack.said..We.have.two.way.radios.inside.our.h elmets..Sorry.whispered.Annie..Jack.put.the.moon.book.back.in.his.pack..Then.he.slung.the.pack.over.his.shoulder...Okay.he. said.Remember.we.only.have.two.hours.of.air.in.our.tanks..So.we.need.to.find.the.fourth.M.thing.really.fast..I.hope.we.can.fi nd.it.said.Annie..Me.too.said.Jack..He.knew.they.could.not.go.home.until.they.did..Lets.go.said.Annie.She.gave.Jack.a.little. push..Watch.it.No.goofing.off.he.said..We.dont.want.to.fall.over.in.these.suits...Just.go.go.said.Annie..She.pushed.him.out.of. the.room..They.walked.back.to.the.airlock..Ready.said.Annie.Open.sesame..She.pressed.the.OPEN.button..A.door.slowly.sli d.closed.behind.them..A.door.opened.in.front.of.them..And.Jack.and.Annie.stepped.out.onto.the.moon...MOON.RABBITS.. Oh.wow.said.Annie..She.took.a.step.forward..But.Jack.stood.frozen..He.wanted.to.get.a.good.look.at.everything.first..He.star ed.at.the.ground..He.was.standing.in.a.layer.of.gray.dust.as.fine.as.powder..Footprints.were.everywhere..Jack.wondered.who. had.made.them...He.reached.into.his.pack.for.the.moon.book..To.his.surprise.it.was.as.light.as.a.feather..He.found.a.picture. of.footprints.on.the.moon.He.read..The.moon.has.no.rain.or.wind.to.blow.the.dust.around..So.footprints.will.never.wear.awa y.naturally.not.even.in.a.billion.years...Oh.man.Jack.said.The.moon.was.the.stillest.place.he.had.ever.ever.been..It.was.as.sti ll.as.a.picture..And.its.stillness.would.never.ever.end..Jack.stared.at.the.ink.black.sky..A.lovely.blue.and.white.ball.glowed.fa

r.away.Earth...For.the.first.time.it.really.hit.Jack..They.were.in.outer.space..Look.Annie.cried.laughing..She.bounded.past.J
ack.almost.flying.through.the.air..She.landed.on.her.feet..Then.she.jumped.again..Im.a.moon.rabbit.she.called...Jack.laughed.
How.does.she.do.that.he.wondered..He.turned.a.page.and.read..A.person.weighs.less.on.the.moon.because.of.the.moons.low.g
ravity.and.lack.of.air..If.you.weigh.pounds.on.Earth.you.would.only.weigh.pounds.on.the.moon..Dont.just.stand.there.readin
g.said.Annie..grabbing.the.book.from.Jacks.gloved.hand..She.tossed.it.into.space..It.flew.far.away...Jack.started.after.it..He.
bounded.up.and.down..Boing.Boing.Boing.Now.he.felt.as.light.as.a.feather..Look.he.called.to.Annie..Im.a.moon.rabbit.too..
Where.Jacks.boots.hit.the.ground.moondust.gracefully.sprayed.into.space..The.book.had.landed.at.the.edge.of.a.shallow.crat
er..When.Jack.reached.it.he.tried.to.stop..But.his.feet.slipped..He.fell.right.over.and.lay.on.his.side..He.tried.to.stand.But.he.
was.off.balance...He.tried.again.But.the.dust.was.just.too.deep..And.his.spacesuit.was.too.clumsy..You.okay.asked.Annie..I.c
ant.get.up.said.Jack..You.shouldnt.have.been.goofing.off.said.Annie.wisely..You.goofed.off.first.said.Jack..Now.help.me.up.pl
ease...Annie.started.toward.him..Dont.fall.too.warned.Jack..I.wont.Annie.moved.very.slowly..She.half.floated.half.walked..G
ive.me.your.hand.she.said..Annie.grabbed.Jacks.hand..She.pressed.her.boot.against.his.and.pulled.him.up..Thanks.he.said..N
o.problem.she.said..It.was.easy.You.were.really.light...Thank.goodness.said.Jack..It.is.impossible.to.get.up.alone..He.picked.
up.the.moon.book..It.was.covered.with.dust..He.brushed.it.off..Oh.wow.Look.said.Annie..She.stood.at.the.edge.of.the.crater..
What.is.it.said.Jack..A.moon.buggy.said.Annie...The.buggy.was.parked.in.the.crater..It.had.four.huge.wheels..Let.us.go.for.a.
ride.said.Annie..We.cant.said.Jack..We.just.have.two.hours.of.air.in.our.tanks.Remember..I.bet.we.will.find.the.M.thing.fast
er..if.we.take.the.moon.buggy.Annie.bounded.into.the.crater..But.we.cant.drive.said.Jack..I.bet.I.can.drive.this.said.Annie..I
t.looks.easy.Come.on...She.jumped.into.the.drivers.seat..But.you.dont.have.a.license.said.Jack..Who.cares.said.Annie..There.
arent.any.roads.on.the.moon.or.stoplights.or.policemen.either..She.was.right.Jack.thought..Well.go.slow.he.said..And.he.clim
bed.in.beside.her..Annie.pushed.a.button.labeled.ON...The.moon.buggy.lurched.backward..Yikes.said.Annie..Step.on.the.bra
ke.said.Jack..Annie.pressed.a.pedal.on.the.floor..The.buggy.stopped.with.a.jerk..Whew.she.said..It.must.be.in.reverse.said.Ja
ck.Let.me.study.this..But.before.he.could.study.anything.Annie.pushed.another.button...The.buggy.tilted.back..Its.front.wh
eels.started.to.rise.into.the.air..Let.me.out.of.here.said.Jack..Annie.pushed.more.buttons..The.buggys.front.wheels.landed.ba
ck.on.the.ground..And.the.buggy.leaped.forward...Slower.said.Jack..I.cant.said.Annie.I.dont.know.how..Annie.steered.the.bu
ggy.over.the.tracks.on.the.ground..The.wide.wheels.kept.it.from.sinking.into.the.deep.dust..Careful.said.Jack..The.buggy.zoo
med.out.of.the.crater..Gray.clouds.of.dust.rose.behind.them.as.they.took.off.across.the.moon...HANG.ON..Annie.drove.the.
moon.buggy.over.bumps.and.hollows..It.bucked.like.a.bronco..Im.going.through.there..She.pointed.to.an.opening.between.tw
o.mountains..Jack.held.on.to.the.dashboard..The.buggy.bumped.toward.the.opening.and.shot.through..On.the.other.side.the.
ground.was.even.rockier..Look.for.the.fourth.M.thing.said.Annie.bouncing.up.and.down..Jack.groaned.Looking.for.anything.
on.this.wild.ride.was.impossible...Slow.down.he.said.How..Try.pressing.on.the.brake.pedal..On.the.floor.slowly..Annie.presse
d.on.the.brake..The.buggy.slowed.down..Jack.sighed.with.relief..The.ride.was.still.bumpy..But.now.at.least.he.could.take.a.g
ood.look.at.the.moon..He.had.never.been.to.such.a.colorless.barren.place..There.was.no.green.no.blue.no.red..No.water.no.tre
es.no.clouds..Only.giant.gray.rocks.and.craters.and.an.American.flag...Oh.man.said.Jack..That.is.from.the.first.astronauts.w
ho.landed.on.the.moon..And.look.a.telescope.said.Annie..She.drove.near.the.flag.and.telescope..Then.she.put.her.foot.on.the.
brake.until.the.buggy.stopped..She.pressed.a.button.that.said.OFF..Then.she.and.Jack.hopped.out..They.took.slow.giant.ste
ps.to.the.site.of.the.first.moon.landing..Beside.the.flag.was.a.sign.Annie.read.it.aloud..HERE.MEN.FROM.THE.PLANET.E
ARTH.FIRST.SET.FOOT.UPON.THE.MOON..WE.CAME.IN.PEACE.FOR.ALL.MANKIND...That.is.a.good.message.sai
d.Jack..He.handed.the.moon.book.to.Annie..Then.he.took.out.his.notebook.and.pencil.to.copy.the.sign..Let.us.leave.our.own.
message.said.Annie..What.should.we.say.said.Jack..The.same.thing.said.Annie..But.say.we.are.the.first.kids..Jack.turned.to.
a.new.page.in.his.notebook..In.big.letters.he.wrote.their.message..Now.we.have.to.sign.it.Annie.said..Jack.signed.his.name...
Then.he.passed.the.notebook.and.pencil.to.Annie..She.signed.her.name.and.passed.the.notebook.back..Jack.tore.out.the.piec
e.of.paper..He.put.it.by.the.flag..No.wind.would.ever.blow.the.message.away..No.rain.would.ever.fall.on.it..It.would.be.there.
forever.unless.someone.moved.it..Thinking.of.forever.made.Jack.feel.dizzy..He.shook.his.head.to.clear.his.thoughts..Then.he.
remembered.the.time.Had.two.hours.passed.yet..I.wish.I.had.a.watch.he.said.standing.up..We.might.be.running.out.of.time..
Oh.wow.A.moon.man.said.Annie...What.Jack.turned.to.look.at.her..She.was.staring.through.the.telescope..Jack.walked.over.
to.the.telescope..Annie.stepped.aside.so.he.could.look.too..Jack.gasped..In.the.distance.something.was.flying.above.the.groun
d..It.looked.like.a.giant.man.in.a.spacesuit...HIGH.JUMP..Who.is.that.said.Jack..I.dont.know.said.Annie..But.we.will.soon.fi
nd.out..She.started.waving..No.said.Jack..He.grabbed.her.arm..Lets.go.back.to.the.base.before.he.gets.here..Why.said.Annie..
We.dont.know.who.he.is.said.Jack..We.dont.know.if.he.is.friendly.or.mean.or.what..But.we.cant.go.back.said.Annie..We.hav
ent.found.the.fourth.M.thing.yet..We.wont.be.able.to.go.home...It.does.not.matter..We.can.lock.the.door.at.the.moon.base.u
ntil.he.goes.away.said.Jack..Then.we.can.get.new.air.tanks..Jack.hurried.to.the.moon.buggy..Come.on.He.jumped.into.the.dr
ivers.seat..Annie.gave.a.little.wave.to.the.dot.in.the.sky..Then.she.climbed.into.the.moon.buggy..The.buggy.took.off..Careful.

said.Annie...They.bumped.over.the.rocks.as.Jack.turned.the.buggy.around..Then.they.zoomed.toward.the.pass..Jack.steered.around.craters.and.rocks..More.than.once.the.buggy.nearly.tipped.over..Whoa.Slow.down.said.Annie..They.were.almost.at.the.mountain.pass..Suddenly.a.cloud.of.dust.flew.up.in.front.of.them..The.ground.trembled..Watch.it.cried.Annie...Jack.couldnt.see.a.thing..He.stepped.on.the.brake..The.buggy.jerked.to.a.stop..The.dust.settled..A.giant.rock.had.fallen.into.the.narrow.pass..It.was.stuck.between.two.walls.of.rock..They.were.trapped..Jack.quickly.found.a.picture.of.a.giant.rock.in.the.moon.book..He.read.aloud...Rocks.of.all.sizes.crash.into.the.moon.from.outer.space..These.rocks.are.called.meteorites..Were.lucky.that.meteorite.didnt.land.on.us.said.Jack..Yeah.and.I.guess.its.too.big.to.be.the.M.thing.said.Annie..She.had.climbed.out.of.the.moon.buggy.and.was.standing.by.the.meteorite..It.was.more.than.twice.as.tall.as.she.was..Jack.looked.at.the.black.sky..The.flying.thing.was.nowhere.in.sight.yet...We.will.have.to.jump.over.it.Annie.said..Jump.I.dont.think.so.said.Jack.Its.too.high..Im.going.to.try.anyway.said.Annie..Wait.Lets.think.first.said.Jack..But.Annie.was.already.backing.up..One.two.three.go.she.shouted.and.took.giant.leaping.steps.toward.the.meteorite...When.Annie.got.close.to.the.rock.she.pushed.off.the.ground..Then.she.flew.through.space.and.disappeared.behind.the.meteorite..Annie.Jack.called..There.was.no.answer..Oh.brother.Jack.said..He.backed.up.and.took.off.toward.the.rock..He.jumped.as.high.as.he.could..Then.he.was.flying.through.space..Jack.hit.the.ground.and.fell.facedown.into.the.dust...Jack.tried.to.stand..But.his.suit.was.too.bulky..He.tried.to.roll.over..But.his.suit.made.even.that.impossible..Oh.no.he.groaned.Not.again..Are.you.here.asked.Annie.Did.you.make.it..Yes.Jack.was.relieved.to.hear.her.voice..But.he.could.not.turn.his.head.to.see.her..He.could.only.hear.her.over.the.radio...Can.you.help.me.up.he.asked..Nope.said.Annie.Why.not..I.fell.down.too.she.said..Oh.brother.Jack.sighed..Now.we.are.really.in.trouble..He.tried.to.stand.again.And.failed..Can.you.see.anything.he.asked..Just.the.sky.said.Annie.Wow.is.it.weird..Im.worried.about.our.air.tanks.said.Jack..I.feel.like.its.been.two.hours...Jack.said.Annie..And.what.about.that.moon.man.said.Jack..Where.did.he.go.to..Jack.whispered.Annie..What.He.is.here.she.said..The.moon.man.is.here..What.He.is.standing.above.me...THE.MOON.MAN..Jacks.heart.nearly.stopped..He.could.hear.Annie.talking..Hi.she.said.We.come.in.peace..There.was.silence..Then.Jack.heard.Annie.say..Thank.you.I.have.to.help.my.brother.up.now..A.moment.later.Annie.rolled.Jack.onto.his.back..She.grabbed.his.hand.and.pulled.him.up...Thanks.said.Jack.once.he.was.standing..The.moon.man.was.a.few.feet.away..His.face.was.hidden.by.a.metal.visor..He.looked.like.a.spaceman..A.huge.spaceman.with.a.giant.tank.on.his.back..It.was.as.big.as.a.refrigerator...That.is.a.jet.pack.said.Jack..Ive.seen.pictures.of.future.astronauts.flying.with.those.things..It.is.like.a.mini.spaceship.Right..The.moon.man.didnt.answer..I.dont.think.he.can.hear.us.said.Annie..He.is.not.hooked.up.to.our.radio...Oh.right.said.Jack.I.will.write.him.a.message..Good.idea.said.Annie..Jack.pulled.out.his.notebook.and.pencil.He.wrote.Were.Jack.and.Annie..We.come.in.peace.from.America.Who.are.you..Jack.handed.the.notebook.and.his.pencil.to.the.moon.man..They.looked.tiny.in.his.big.hands...The.moon.man.looked.down.at.the.message..He.looked.at.the.tiny.pencil..Then.he.turned.the.notebook.over..Jack.and.Annie.watched.as.the.moon.man.put.the.pencil.to.the.paper..He.was.writing.something.very.carefully...Finally.he.gave.the.notebook.back.to.Jack..Jack.and.Annie.stared.at.the.marks..Stars.said.Annie.He.drew.stars..Maybe.it.is.a.space.map.said.Jack..Space.map.said.Annie..Hey.Jack.map.starts.with.M...Oh.wow.said.Jack..This.must.be.the.fourth.M.thing..Lets.ask.him.what.his.map.means.said.Annie..She.turned.around..We.will.never.know.now.she.said..Why.Jack.looked.up.from.the.map..That.is.why.Annie.pointed..The.moon.man.was.flying.over.the.mountains..Thanks.Annie.cried...ONE.STAR.TO.ANOTHER..Who.was.that.guy.said.Jack..What.does.his.map.mean..I.dont.know.said.Annie..But.lets.see.if.it.works..Jack.took.a.deep.breath..Yeah.wed.better.hurry.back..I.think.Im.running.out.of.air..It.feels.harder.to.breathe..For.me.too.said.Annie..Go.slow.Dont.breathe.too.much.said.Jack..He.and.Annie.took.long.floating.steps.toward.the.moon.base..Jack.held.his.breath.as.if.he.were.underwater.By.the.time.they.got.to.the.white.dome.he.was.ready.to.burst...Annie.pushed.a.button.beside.the.huge.door..It.slid.open.They.hurried.into.the.airlock..The.door.closed.behind.them.and.the.door.to.the.hallway.opened..Jack.opened.the.visor.of.his.helmet..He.took.a.long.deep.breath.and.let.it.out..Let.us.get.out.of.these.suits.said.Annie..Good.idea.Jack.was.dying.to.free.his.arms.and.legs...As.they.moved.clumsily.into.the.spacesuit.storeroom..Jack.felt.heavy.again..He.and.Annie.unlocked.each.others.helmets.gloves..and.boots.and.pulled.everything.off..Then.they.stepped.out.of.their.bulky.suits..Whew.Jack.said.He.took.off.his.glasses.and.rubbed.his.eyes..It.was.great.to.be.free..even.if.he.no.longer.felt.as.light.as.a.feather..Hurry.Peanuts.waiting.said.Annie...She.led.the.way.down.the.steps.to.the.bright.landing.chamber..Yay.she.said.softly..Jack.was.relieved.to.see.the.tree.house.still.there..Soon.theyd.be.heading.home..He.couldnt.wait..Jack.and.Annie.crawled.through.the.tree.house.window...Were.back.Peanut.said.Annie..Squeak.Peanut.ran.to.the.letter.M..We.missed.you.said.Annie..She.patted.the.mouses.head..We.met.a.moon.man..Sorry.Peanut.but.you.have.to.move.said.Jack..We.have.to.put.the.map.on.the.M..Annie.gently.lifted.the.mouse.off.the.M..Jack.tore.the.star.map.out.of.his.notebook..He.placed.it.on.the.M.next.to.the.mammoth.bone.the.mango.and.the.moonstone...He.sighed.then.sat.back.on.his.heels.Hand.me.the.Pennsylvania.book.he.said..They.needed.the.Pennsylvania.book.to.get.back.home..There.was.silence..Jack.turned.and.looked.at.Annie..It.is.not.working.Jack.she.said..The.book.is.not.here..What.Was.the.map.the.wrong.thing..They.looked.around.the.tree.house..Its.definitely.not.here.said.Annie...Oh.no.Jacks.heart.sank..He.picked.up.the.star.map.and.stared.at.it..Squeak.squeak.Peanut..jumped.out.of.Annies.ar

ms.and.scurried.back.to.the.letter.M...Ive.got.an.idea.said.Jack..He.reached.into.his.pack.and.took.out.his.pencil..What.are.y ou.doing.said.Annie..You.know.how.you.draw.a.constellation.said.Jack..You.connect.all.the.stars.What.happens.if.we.try.th at...He.drew.a.line.from.one.star.to.another..He.kept.drawing.until.all.the.stars.were.connected..Let.me.see.said.Annie..Jack. held.the.paper.out.so.they.could.both.study.it..It.looks.like.a.mouse.said.Annie..Yeah.said.Jack...Is.there.such.a.thing.as.a.m ouse.constellation.said.Annie..I.dont.think.so.said.Jack..Squeak.Annie.and.Jack.looked.at.Peanut..She.was.standing.on.the. M..Oh.wow.Jack.Annie.whispered..I.think.I.know.what.the.fourth.thing.is...Jack.grinned..Me.too.he.said..It.is.a.Mouse.they. said.together..Squeak.Squeak..Maybe.the.spell.is.Moonstone..mango.mammoth.bone.mouse.said.Annie..Jack.touched.each.M. thing.in.turn.as.he.whispered..Moonstone.mango.mammoth.bone.mouse..Lets.say.it.over.and.over.and.see.what.happens.said .Annie..Together.they.chanted...Moonstone.mango.mammoth.bone.mouse..Moonstone.mango.mammoth.bone.mouse..Sudde nly.a.bright.light.filled.the.tree.house..The.light.got.brighter.and.brighter.and.brighter..The.brightness.was.blinding.and.whi rling..The.air.spun.with.brightness..Then.everything.was.clear..Peanut.the.mouse.was.gone..And.Morgan.le.Fay.stood.before. Jack.and.Annie...MORGAN..Thank.you.Morgan.said.softly..You.have.freed.me.from.the.magicians.spell..Jack.just.stared.at. her..You.were.Peanut.Annie.said..Morgan.nodded.and.smiled..Really.You.were.with.us.all.the.time.said.Jack..On.all.our.mis sions..Morgan.nodded.again..Why.did.we.have.to.go.on.this.mission.to.find.a.mouse.said.Jack..If.you.were.always.with.us...T o.break.the.spell.we.had.to.be.on.the.moon.said.Morgan..You.could.have.broken.it.the.minute.we.arrived..Oh.thats.what.Pe anut.I.mean.you.were.trying.to.say.said.Annie..We.didnt.have.to.leave.the.moon.base.at.all..Morgan.nodded.smiling...But.th e.moon.man.came.along.to.help.us.said.Annie..He.drew.a.constellation.of.a.mouse..Is.he.a.friend.of.yours..Morgan.shrugged.L ets.just.say.we.had.a.little.talk..He.stopped.by.the.moon.base.while.you.were.out..The.same.way.you.had.a.talk.with.the.ninj a.master.right.said.Jack..And.the.monkey.and.the.sorcerer..Morgan.nodded.I.always.squeaked.to.the.ones.who.helped.you... But.how.did.they.understand.you.a.mouse.said.Jack..Morgan.smiled.again..Certain.wise.ones.understand.the.language.of.litt le.creatures.she.said..I.bet.it.was.you.who.turned.the.pages.of.the.books.said.Annie..To.show.us.where.to.go.next..Morgan.no dded..But.who.turned.you.into.a.mouse.said.Annie...Morgan.frowned..A.certain.person.who.likes.to.play.tricks.on.me.she.sai d..His.name.is.Merlin..Merlin.said.Jack..The.greatest.magician.who.ever.lived..Morgan.sniffed.he.is.not.that.great.she.said..H e.does.not.even.know..I.have.two.brave.friends.who.help.me..Us.said.Annie.shyly..Morgan.nodded..And.I.thank.you.both.wit h.all.my.heart...You.are.welcome.said.Jack.and.Annie..Morgan.handed.Annie.the.Pennsylvania.book..Are.you.ready.to.go.ho me.now.she.asked..Yes.said.Jack.and.Annie..Annie.pointed.to.a.picture.of.the.Frog.Creek.woods..I.wish.we.could.go.there.sh e.said..The.tree.house.started.to.spin..It.spun.faster.and.faster.and.faster..Then.everything.was.still..Absolutely.still..But.onl y.for.a.moment...EARTH.LIFE..The.midnight.woods.woke.up..A.breeze.rustled.the.leaves..An.owl.hooted..The.sounds.were. soft.but.very.alive..Jack.opened.his.eyes..He.pushed.his.glasses.into.place..He.smiled.Morgan.was.still.with.them..He.could.se e.her.in.the.moonlight..Her.long.white.hair.was.shining..Morgan.can.you.and.the.tree.house.stay.here.said.Annie.In.Frog.Cre ek..No.I.must.leave.again..Im.afraid.said.Morgan..Ive.been.gone.from.Camelot.for.a.long.time...She.handed.Jack.his.pack..Sh e.brushed.his.cheek..Her.hand.felt.soft.and.cool..A.bit.of.moondust.still.on.you.she.said..Thank.you.Jack.for.your.great.love. of.knowledge..Youre.welcome.said.Jack..Morgan.tugged.on.one.of.Annies.braids..And.thank.you.Annie..for.your.belief.in.the. impossible..Youre.welcome.said.Annie..Go.home.now.said.Morgan...Jack.smiled..Home.was.Earth.that.bright.colorful.world.. where.everything.was.alive.and.always.changing..Bye.Morgan.said.Annie..She.started.out.of.the.tree.house..Jack.looked.back .at.Morgan..Will.you.come.back.soon.he.said..Anything.can.happen.said.Morgan..The.universe.is.filled.with.wonders.Isnt.it. Jack..He.smiled.and.nodded...Go.now.Morgan.said.softly..Jack.followed.Annie.down.the.rope.ladder..He.stepped.onto.the.gr ound..The.wind.started.to.blow..The.tree.started.to.shake..A.loud.roar.filled.Jacks.ears..He.squeezed.his.eyes.shut..He.covere d.his.ears..Then.everything.was.silent.and.still...Jack.opened.his.eyes..The.ladder.was.gone..He.looked.through.the.leaves.an d.branches.of.the.giant.oak.tree..Where.the.tree.house.had.been.was.only.moonlight.now..Bye.Morgan.he.whispered.sadly..B ye.Peanut.said.Annie..Jack.and.Annie.stared.at.the.top.of.the.tree.for.a.long.moment..Ready.said.Annie..Jack.nodded..They. started.for.home...The.midnight.air.felt.cool.and.moist..It.was.filled.with.the.soft.sounds.of.earth.life..Jack.and.Annie.left.the. Frog.Creek.woods..They.started.down.their.street..Annie.glanced.up.at.the.sky..The.moon.looks.really.far.away.does.not.it..I t.did.thought.Jack.It.was...I.wonder.how.the.moon.man.can.be.up.there.all.alone.said.Annie..What.do.you.mean.said.Jack..I. mean.who.helps.him.put.on.his.spacesuit.said.Annie..Who.helps.him.get.up.when.he.falls.down..And.who.is.he.added.Jack.. Who.do.you.think.he.was.said.Annie..He.must.be.a.scientist.or.an.astronaut.from.Earth.said.Jack..No.I.think.he.is.an.alien.s aid.Annie.from.another.galaxy...Jack.scoffed..What.makes.you.say.that..I.just.feel.it.said.Annie..Wrong.said.Jack..Theres.no. proof.that.aliens.exist..Maybe.not.now.said.Annie..But.dont.forget.we.were.in.the.future..Oh.brother.said.Jack..They.crossed .their.yard.and.climbed.their.back.steps..Annie.tiptoed.inside.the.house...Jack.followed.her..Before.he.shut.the.door.he.glanc ed.up.at.the.moon..Was.Annie.right.he.wondered..Could.the.moon.man.have.come.from.another.galaxy..Morgans.words.cam e.back.to.him..The.universe.is.filled.with.wonders..Isnt.it.Jack..Goodnight.moon.man.Jack.whispered..Then.he.closed.the.do or...

SI Dataset S5 (insulin_2-level.txt)

Human preproinsulin, with two levels of description
Level 1: 330 nucleotide sequence
Level 2: 110 amino acids
source: https://www.uniprot.org/uniprotkb/P01308/entrysequences

atg.gcc.ctg.tgg.atg.cgc.ctc.ctg.ccc.ctg.ctg.gcg.ctg.ctg.gcc.ctc.tgg.gga.cct.gac.cca.gcc.gca.gcc.ttt.gtg.aac.caa.cac.ctg.tgc.ggc.tca.cac.ctg.gtg.gaa.gct.ctc.tac.cta.gtg.tgc.ggg.gaa.cga.ggc.ttc.ttc.tac.aca.ccc.aag.acc.cgc.cgg.gag.gca.gag.gac.ctg.cag.gtg.ggg.cag.gtg.gag.ctg.ggc.ggg.ggc.cct.ggt.gca.ggc.agc.ctg.cag.ccc.ttg.gcc.ctg.gag.ggg.tcc.ctg.cag.aag.cgt.ggc.att.gtg.gaa.caa.tgc.tgt.acc.agc.atc.tgc.tcc.ctc.tac.cag.ctg.gag.aac.tac.tgc.aac

SI Dataset S6 (lysozyme_2-level.txt)

Human lysozyme, with two levels of description
Level 1: 444 nucleotide sequence
Level 2: 148 amino acids
source: https://www.uniprot.org/uniprotkb/P61626/entrysequences

atg.aag.gct.ctc.att.gtt.ctg.ggg.ctt.gtc.ctc.ctt.tct.gtt.acg.gtc.cag.ggc.aag.gtc.ttt.gaa.agg.tgt.gag.ttg.gcc.aga.act.ctg.aaa.aga.ttg.gga.atg.gat.ggc.tac.agg.gga.atg.agc.cta.gca.aac.tgg.atg.tgt.ttg.gcc.aaa.tgg.gag.agt.ggt.tac.aac.aca.cga.gct.aca.aac.tac.aat.gct.gga.gac.aga.agc.act.gat.tat.ggg.ata.ttt.cag.atc.aat.agc.cgc.tac.tgg.tgt.aat.gat.ggc.aaa.acc.cca.gga.gca.gtt.aat.gcc.tgt.cat.tta.tcc.tgc.agt.gct.ttg.ctg.caa.gat.aac.atc.gct.gat.gct.gta.gct.tgt.gca.aag.agg.gtt.gtc.cgt.gat.cca.caa.ggc.att.aga.gca.tgg.gtg.gca.tgg.aga.aat.cgt.tgt.caa.aac.aga.gat.gtc.cgt.cag.tat.gtt.caa.ggt.tgt.gga.gtg

SI Dataset S7 (myoglobin_2-level.txt)

Human myoglobin, with two levels of description
Level 1: 462 nucleotide sequence
Level 2: 154 amino acids
source: https://www.uniprot.org/uniprotkb/P02144/entrysequences

atg.ggg.ctc.agc.gac.ggg.gaa.tgg.cag.ttg.gtg.ctg.aac.gtc.tgg.ggg.aag.gtg.gag.gct.gac.atc.cca.ggc.cat.ggg.cag.gaa.gtc.ctc.atc.agg.ctc.ttt.aag.ggt.cac.cca.gag.act.ctg.gag.aag.ttt.gac.aag.ttc.aag.cac.ctg.aag.tca.gag.gac.gag.atg.aag.gca.tct.gag.gac.tta.aag.aag.cat.ggt.gcc.act.gtg.ctc.acc.gcc.ctg.ggt.ggc.atc.ctt.aag.aag.aag.ggg.cat.cat.gag.gca.gag.att.aag.ccc.ctg.gca.cag.tcg.cat.gcc.acc.aag.cac.aag.atc.ccc.gtg.aag.tac.ctg.gag.ttc.atc.tcg.gaa.tgc.atc.atc.cag.gtt.ctg.cag.agc.aag.cat.ccc.ggg.gac.ttt.ggt.gct.gat.gcc.cag.ggg.gcc.atg.aac.aag.gcc.ctg.gag.ctg.ttc.cgg.aag.gac.atg.gcc.tcc.aac.tac.aag.gag.ctg.ggc.ttc.cag.ggc
